# Ligand-enabled Ni-catalyzed hydroarylation and hydroalkenylation of internal alkenes with organoborons

**DOI:** 10.1038/s41467-022-34675-x

**Published:** 2022-11-12

**Authors:** Dao-Ming Wang, Li-Qin She, Yichen Wu, Chunyin Zhu, Peng Wang

**Affiliations:** 1 State Key Laboratory of Organometallic Chemistry, Shanghai Institute of Organic Chemistry, University of Chinese Academy of Sciences, CAS, 345 Lingling Road, Shanghai, 200032 PR China; 2 School of Chemistry and Chemical Engineering, Jiangsu University, Zhenjiang, 212013 PR China; 3 CAS Key Laboratory of Energy Regulation Materials, Shanghai Institute of Organic Chemistry, CAS, 345 Lingling Road, Shanghai, 200032 PR China; 4 School of Chemistry and Materials Science, Hangzhou Institute for Advanced Study, University of Chinese Academy of Sciences, 1 Sub-lane Xiangshan, Hangzhou, 310024 PR China

**Keywords:** Synthetic chemistry methodology, Homogeneous catalysis

## Abstract

The transition metal-catalyzed hydrofunctionalization of alkenes offers an efficient solution for the rapid construction of complex functional molecules, and significant progress has been made during last decades. However, the hydrofunctionalization of internal alkenes remains a significant challenge due to low reactivity and the difficulties of controlling the regioselectivity. Here, we report the hydroarylation and hydroalkenylation of internal alkenes lacking a directing group with aryl and alkenyl boronic acids in the presence of a nickel catalyst, featuring a broad substrate scope and wide functional group tolerance under redox-neutral conditions. The key to achieving this reaction is the identification of a bulky 1-adamantyl *β*-diketone ligand, which is capable of overcoming the low reactivity of internal 1,2-disubstituted alkenes. Preliminary mechanistic studies unveiled that this reaction undergoes an Ar-Ni(II)-H initiated hydroarylation process, which is generated by the oxidative addition of alcoholic solvent with Ni(0) species and sequential transmetalation. In addition, the oxidative addition of the alcoholic solvent proves to be the turnover-limiting step.

## Abstract (web summary)

The hydrocarbofunctionalization of alkenes represents one of the most efficient methods for generating complex products from simple starting materials. Here, the authors report a bulky β-diketone-ligand-enabled hydroarylation and hydroalkenylation of internal alkenes.

## Introduction

**Fig. 1:**
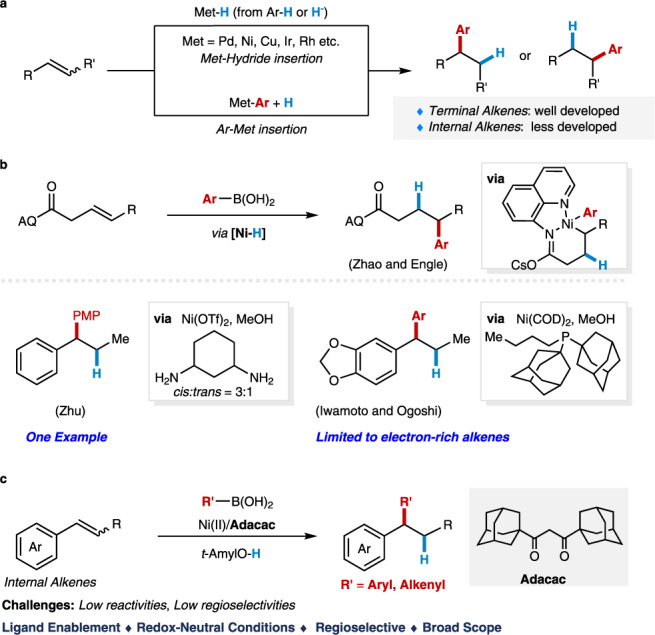
Synopsis for Ni-catalyzed hydroarylation and hydroalkenylation of internal alkenes. **a** Transition-metal catalyzed hydroarylation of alkenes. **b** Ni-catalyzed hydroarylation of internal alkenes with arylborons. **c** Ligand enabled Ni-catalyzed hydroarylation and hydroalkenylation of internal alkenes with organoborons. Ar aryl, PMP *p*-methoxyphenyl.

The transition metal-catalyzed hydrofunctionalization of alkene represents one of the most efficient methods for generating structurally complex products from commercially available alkenes, which has significantly accelerated the advances in synthetic chemistry in the last decades^1–7^. Among those transformations, the hydroarylation of alkene with various arylating reagents has attracted much attention for direct construction of carbon-carbon bond (Fig. 1a)^8–11^. To date, various strategies have been developed to render the reactivity and control regioselectivity for the transition-metal catalyzed hydroarylation, and the major progress in this area has been made for both conjugated alkenes and non-conjugated terminal alkenes^12–42^. However, the direct hydroarylation of sterically more congested internal alkenes, such as 1,2-disubstituted alkene and trisubstituted alkenes, is comparatively rare and remains a major challenge due to the steric hindrance and the difficulties of controlling the regioselectivity. Early successes for hydroarylation of internal alkenes relied on the high reactivity of constrained cyclic alkenes and polar conjugated alkenes developed by Catellani^12^ and Cacchi^13,14^. Recently elegant developments have built up on the rapid emergence of metal-hydride chemistry^19,20,27^, and the directing group approach^31–42^. For instance, Zhu has developed a Ni–H initiated hydroarylation of 1,2-disubstituted alkenes with aryl iodides as the arylating reagents in the presence of stoichiometric silanes^30^. Engle^32,33,36,37^, Loh^34,38–40^, Chen^35^, and others^41,42^ have demonstrated the hydroarylation of internal alkenes via a directing group approach which provides the precise control of the regioselectivity via cyclometallation. Despite the noteworthy advances, the development of a general method under redox-neutral conditions without extra reductants for the hydroarylation of undirected 1,2-substituted internal alkenes is still lacking and highly desired.

Recently, the nickel catalyzed hydroarylation under redox-neutral conditions has emerged as an appealing approach for the functionalization of alkenes pioneered by Zhou^43,44^ in 2018. Although the Ni(0)/phosphine ligand system showed to be incompatible with internal alkenes and unactivated alkenes previously, Zhao and Engle mentioned the success of hydroarylation of the internal alkenes with the strong bidentate directing group^45,46^, in which the extra manipulations for the installation and removal of the directing groups are required. Very recently, Zhu^47^, Iwamoto and Ogoshi^48^ indicated the hydroarylation of internal 1,2-disubsituted alkene could indeed happen during their studies of migratory hydroarylation of terminal alkenes. However, only one case of the *β*-substituted styrene was mentioned in Zhu’s protocol, and the process developed by Iwamoto and Ogoshi was largely limited to electron-rich substrates with moderate yields (ave. 40–60% yields) in the presence of Ni(0) and a bulky mono phosphine ligand PAd_2_(*n*-Bu) (Fig. 1b). Moreover, the Ni-catalyzed hydroalkenylation of internal alkenes with alkenylborons has not been disclosed yet^44,45,49–54^.

In this work, we reported the discovery of a bulky *β*-diketone ligand which is capable of enabling the hydroarylation and hydroalkenylation of internal alkenes with broad substrate scope and functional group tolerance (Fig. 1c), thus providing a practical access to 1,1-diarylalkanes, a biologically active structure common in both pharmaceuticals and natural products^55–57^. Moreover, this study led to the development of a nickel(II) precursor, Ni(Adacac)_2_, which shows superior reactivity for the hydroarylation of internal alkenes in comparison with the commercially available Ni(acac)_2_. Notably, the potential and the role of the *β*-diketone ligands in nickel catalysis are not fully established, despite the Ni(acac)_2_ has been used as a precursor for a long time in nickel catalysis, and *β*-diketone derivatives bonded nickel complexes have been utilized in Ni-catalyzed radical coupling reaction^58,59^.

## Results and discussion

### Reaction optimization

**Fig. 2:**
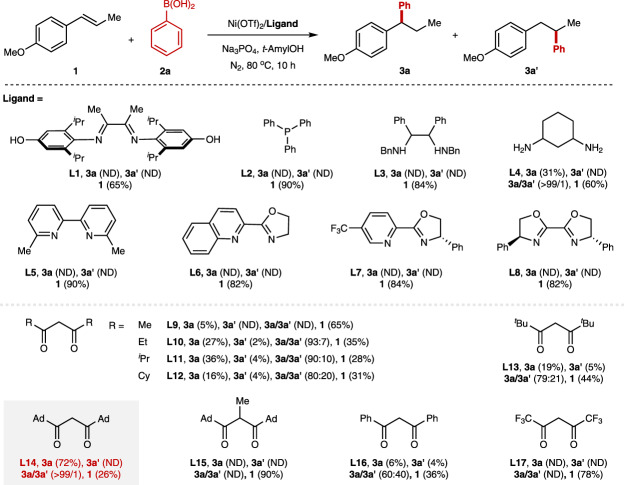
Ligand evaluation for Ni-catalyzed hydroarylation of internal alkenes. Reaction conditions: **1** (0.1 mmol, 1.0 equiv), **2a** (0.2 mmol, 2.0 equiv), Ni(OTf)_2_ (10 mol%), Ligand (10 mol%), Na_3_PO_4_ (32.8 mg, 2.0 equiv), *t*-AmylOH (0.5 mL), 80 °C, N_2_, 10 h; Ad 1-adamantyl, ND not detected. The yield was determined by ^1^H NMR using dibromomethane as the internal standard.

In the context of our recent efforts on Ni-catalyzed hydroarylation of unactivated alkenes, we found the redox-neutral Ni(II)-catalysis is capable of the hydroarylation of cyclohexene and 1,1-disubsituted alkenes, albeit with lower efficiency^60^. Hence, we envisioned that the more challenging hydroarylation of 1,2-disubsituted internal alkenes could be realized via a ligand acceleration approach using nickel as the catalyst under redox-neutral conditions. Unfortunately, the hydroarylation for the 1,2-disubstituted alkene didn’t proceed in the presence of Ni(acac)_2_ and electron-rich diamine ligand **L1**, which is superior in the redox-neutral Ni(II)-catalyzed hydroarylation of unactivated alkene. We next checked the ligand effects employing the Ni(OTf)_2_ as the catalyst precursor (Fig. 2). To our great surprise, phosphine ligand (**L2**), diamine ligand (**L3**), bipyridine ligand (**L5**), pyridine-oxazoline ligand (**L6**, **L7**) and bis-oxazoline ligand (**L8**), which are privileged in nickel catalysis, all resulted in no reaction. 1,3-cyclohexanediamine (**L4**), the optimal ligand in Zhu’s migratory hydroarylation reaction, resulted in 31% yield with excellent regioselectivity (**3a**/**3a’** > 99/1). Those results further indicate the challenge of the hydroarylation of internal alkenes. Given the fact that 5% yield of desired product **3a** could be observed by the addition of 10 mol% of acac ligand, we next systematically evaluated the effects of *β*-diketone ligands using Ni(OTf)_2_ as the catalyst precursor. To our delight, the switch of the acac ligand to ethyl group led to 27% yield of the desired product with a regioselectivity ratio of 93/7. Further increase the steric hindrance of the substituents contributed inferior regioselectivities (**L11**-**13**). Gratifyingly, the 1-adamantyl substituted *β*-diketone ligand **L14** resulted in 72% yield of desired hydroarylated product **3a** with excellent regioselectivity (**3a**/**3a’** > 99/1). Installation of a methyl group in the methylene position on ligand **L14** led to no reaction (**L15**). Dibenzoylmethane (**L16**) is also inefficient for this reaction, and hexafluoroacetylacetone (**L17**) resulted in no reaction. The yield was further optimized to 96% ^1^H NMR yields (90% isolated yield) with excellent regioselectivity employing Ni(2-NH_2_−5-MeC_6_H_3_SO_3_)_2_ as the nickel source and 3.0 equivalent of phenylboronic acid. Notably, both the *trans*-anethol and *cis*-anethol are compatible with the standard conditions, providing the desired product in high yield with excellent regioselectivity (For details, see Supplementary Methods). Control experiments indicate all parameters are crucial for this reaction, and the reaction doesn’t proceed in the absence of *β*-diketone ligand **L14** or base.

### Substrate scope

**Fig. 3:**
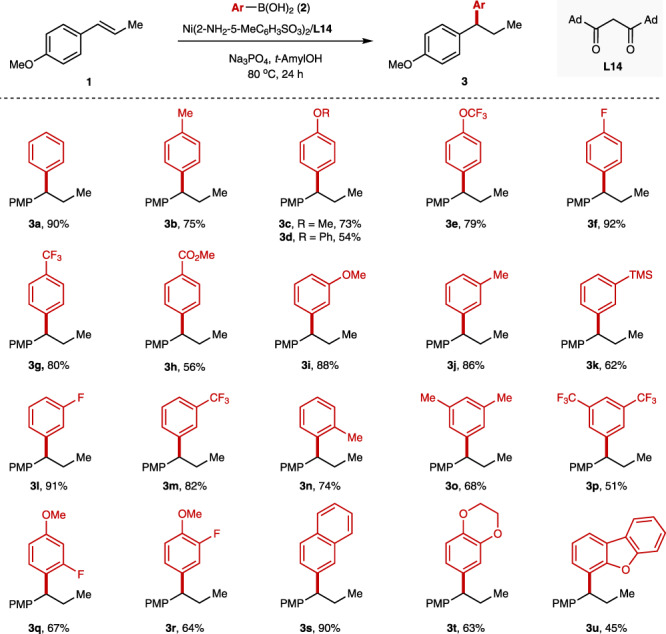
The scope of arylboronic acids. The values under each structure indicate isolated yields. The regioselectivity was determined by analysis of the crude ^1^H NMR (rr > 99/1 for all substrates). Reaction conditions: **1** (0.2 mmol, 1.0 equiv), **2** (0.6 mmol, 3.0 equiv), Ni(2-NH_2_−5-MeC_6_H_3_SO_3_)_2_ (10 mol%), **L14** (10 mol%), Na_3_PO_4_ (0.6 mmol, 3.0 equiv), *t*-AmylOH (1.0 ml), 80 °C, N_2_, 24 h. For **3h** the reactions were conducted with Ni(OTf)_2_ instead of Ni(2-NH_2_-5-MeC_6_H_3_SO_3_)_2_.

With internal alkene **1** (*trans*-anethol) as the model substrate, the breadth of this *β*-diketone ligand enabled Ni-catalyzed hydroaryaltion was evaluated. A wide range of functional groups are well tolerated, delivering the desired hydroarylated products in moderate to excellent yields and excellent regioselectivities. As listed in Fig. 3, both electron-deficient and electron-rich aryl boronic acids at different positions on the arenes are suitable arylating reagents for this hydroarylation reaction. A wide range of functional groups, including methyl (**2b**, **2j**, **2n**), methoxy (**2c**, **2i**), trifluoromethoxy (**2e**), fluoro (**2f**, **2l**), trifluoromethyl (**2g**, **2m**), ester (**2h**), trimethylsilyl (**2k**) etc., are all tolerated, providing the corresponding products in moderate to excellent yields. The substituents at *ortho-*, *meta-*, *para-* position are all compatible, although the *ortho*-substituent normally led to a slightly lower yield probably due to the steric hindrance (**2n** vs. **2b**, **2j**). Multisubstituted aryl boronic acids (**2o**–**r**) and 2-naphthyl boronic acid (**2s**) gave the hydroarylated products in 51–90% yields. Notably, the process is also suitable for electron-rich heteroaryl boronic acid, such as 1,4-benzodioxane-6-boronic acid (**2t**) and 4-(dibenzofuranyl)boronic acid (**2u**).

**Fig. 4:**
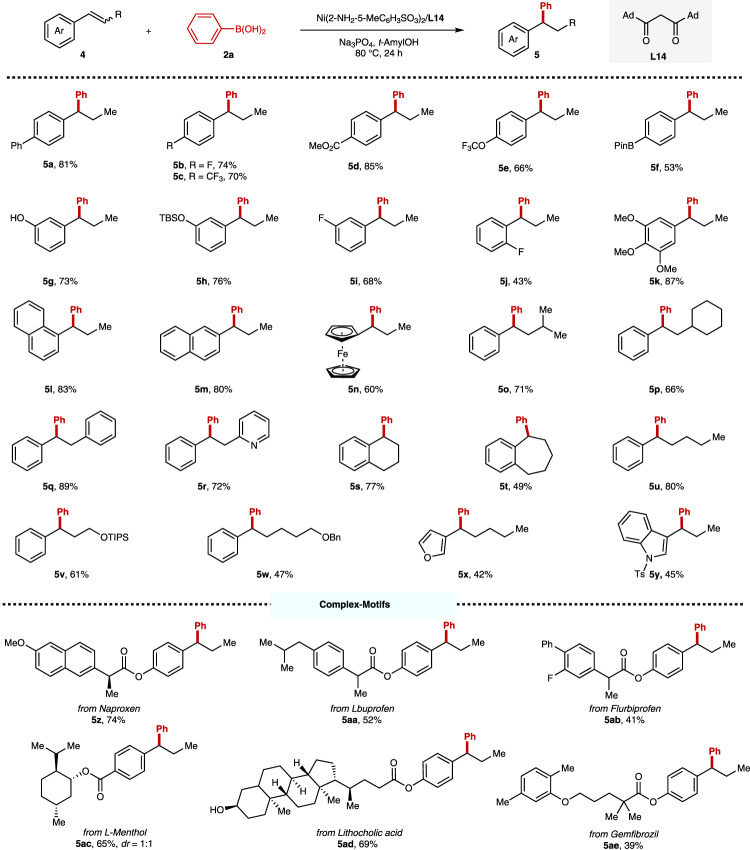
The scope of internal alkenes. The values under each structure indicate isolated yields. The regioselectivity was determined by analysis of the crude ^1^H NMR (rr > 99/1 for all substrates) and the dr was determined by GC. Reaction conditions: **4** (0.2 mmol, 1.0 equiv), **2a** (0.6 mmol, 3.0 equiv), Ni(2-NH_2_-5-MeC_6_H_3_SO_3_)_2_ (10 mol%), **L14** (10 mol%), Na_3_PO_4_ (0.6 mmol, 3.0 equiv), *t*-AmylOH (1.0 mL), 80 °C, N_2_, 24 h. The *Z/E* mixtures were used for most of substrates. For **5q** and **5v**, (*E*)-**4q** and (*E*)-**4v** were used; For **5s**, (*Z*)-**4s** was used.

We next investigated the scope of the internal 1,2-disubstituted alkenes with phenylboronic acid (**2a**) as the aryl sources (Fig. 4). Preliminary control experiments demonstrated that both *cis*- and *trans*- internal alkenes showed similar reactivities under our optimal conditions, and preliminary kinetic experiments unveiled a similar reaction rate for both *cis*- and *trans*- internal alkenes during the hydroarylation event (see Supplementary Methods for details). Accordingly, a broad range of internal alkenes containing both *cis*- and *trans*- isomers are evaluated directly. To our delight, this protocol presents high level of compatibility of a large range of *β*-substituted styrenes, affording good yields and excellent regioselectivities. The substrates bearing both the electron-rich and the electron-deficient substituents showed similar reactivities for this protocol (**4a**–**m**). It is noteworthy that the BPin group (**4f**), and naked phenoxy group (**4g**) are tolerated with this hydrodarylation process which underscores the generality of this reaction. This feature provides the possibility of facile sequential functionalizations of the corresponding hydroarylated products. The *ortho*-substituent led to a lower yield (**4j** vs. **4b**, **4i**), and the regioselectivity remains. Moreover, ferrocene-based internal alkene **4n** is also suitable for this protocol, giving the corresponding product in 60% yield. Replacement of the *β*-methyl group with bulky *n*-propyl (**4u**), isopropyl (**4o**), cyclohexyl (**4p**), phenyl group (**4q**), and other functionalized alkyl groups (**4v**, **w**) did not significantly affect the reactivities. The cyclic alkenes (**4s**, **t**) are also tolerated to provide the hydroarylated products in moderate to good yields. Notably, this process also showed high level of compatibilities with heterocyclic substrates (**4r**, **4x**, **y**), like pyridine, furan, and indole, giving the desired products in moderate yields. The tolerance with complex motifs derived from natural products and marketed drugs was also evaluated. A series of derivatives of bioactive molecules, including naproxen, lbuprofen, flurbiprofen, menthol, lithochiolic acid, and gemfibrozil, proved to be compatible with our protocol.

**Fig. 5:**
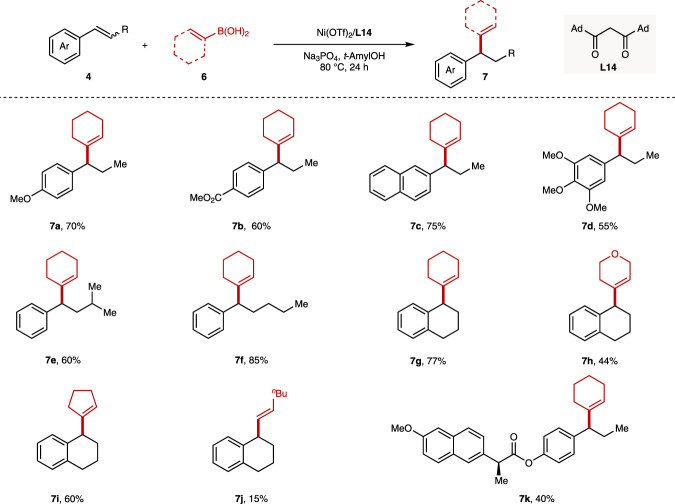
Hydroalkenylation of internal alkenes with alkenyl boronic acids. The values under each structure indicate isolated yields. The regioselectivity was determined by analysis of the crude ^1^H NMR (rr > 99/1 for all substrates). Reaction conditions: **4** (0.2 mmol, 1.0 equiv), **6** (0.6 mmol, 3.0 equiv), Ni(OTf)_2_ (10 mol%), **L14** (10 mol%), Na_3_PO_4_ (0.6 mmol, 3.0 equiv), *t*-AmylOH (1.0 mL), 80 °C, N_2_, 24 h. The *Z*/*E* mixtures were used for most of substrates. For **7a** and **7d**, (*E*)-**1** and (*E*)-**4k** were used; For **7g**–**j**, (*Z*)-**4s** was used.

Encouraged by the success of the hydroarylation of internal alkenes, we envisioned that the hydroakenylation of the 1,2-disubstituted alkenes might be feasible with our developed *β*-diketone ligands. To our delight, the hydroalkenylation of internal alkenes with alkenylboronic acids has been realized in high efficiency with ligand **L14**, as shown in Fig. 5. Employing the cyclohexenyl boronic acid as the model substrate, this protocol could compatible with electron-rich *β*-substituted styrenes (**1**, **4k**), electron-deficient *β*-substituted styrene (**4d**), bulky *β*-substituted styrene derivatives (**4u**, **4o**) and naproxen-derived substrate (**4z**), providing corresponding alkenylated products **7a**–**f** in 40–85% yields. In addition, the cyclic alkene **4s** gave the desired product **7g** in 77% yield. The scope of alkenyl bronoic acids for the hydroalkenylation was next examined. The cyclic alkenyl bronoic acids showed better yields than the acyclic alkenyl bronoic acid (**7i**, **k** vs. **7j**), probably due to the rapid hydrolysis of the acyclic alkenyl boronic acids.

### Mechanistic insights

**Fig. 6:**
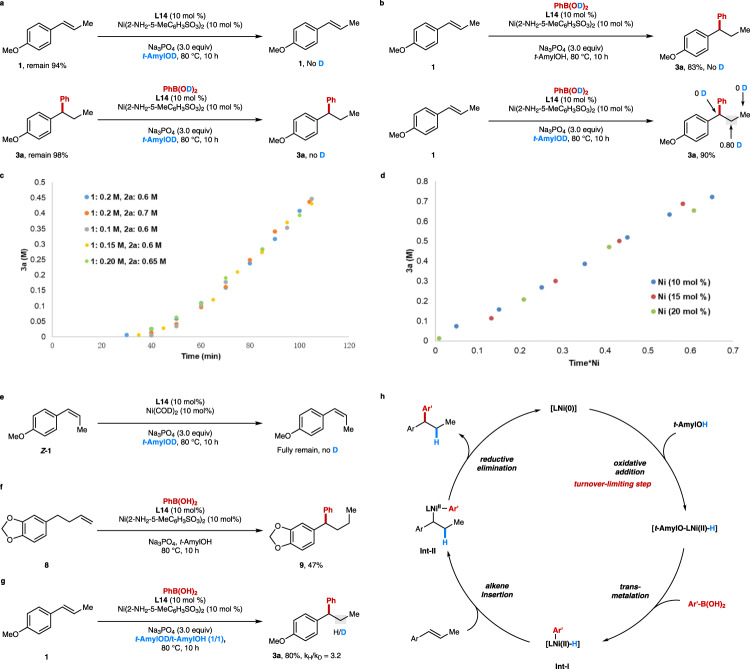
Mechanistic studies. **a** Deuterium scrambling experiments. **b** Deuteration experiments. **c** “Different excess” experiments for determining the substrates orders. **d** [Ni] order determination based on the variable time normalization analysis, [Ni] = 10, 15, 20 mM. **e** Isomerization of *Z*-alkene under optimal conditions. **f** Ni-catalyzed migratory hydroarylation. **g** KIE study with *tert*-amyl alcohol. **h** Proposed catalytic cycles.

To further understand this bulky 1-adamantyl *β*-diketone ligand enabled hydroarylation reaction of internal alkenes, detailed mechanistic studies were carried out. No H/D exchange happened when treating the alkene **1** and product **3** under the standard conditions with deuterium-labeled *tert*-amyl alcohol and arylboronic acid **D-2a** (Fig. 6a). The control experiments with deuterium-labeled *tert*-amyl alcohol and arylboronic acid **D-2a** disclosed that the H in the product came from the solvent, and no deuterium detected in other positions except *β*-position (Fig. 6b). Moreover, the Ni(COD)_2_ is also an effective catalyst for this hydroarylation reaction, unlike our previous Ar-Ni(II) initiated reaction where the Ni(COD)_2_ is inactive^60^. The kinetic studies are next conducted by the reaction progress kinetic analysis, revealing that this reaction is zero order to alkene and arylboronic acid and first order with regard to Ni(2-NH_2_-5-MeC_6_H_3_SO_3_)_2_/**L14** catalyst (Fig. 6c, d). Moreover, the induction period in the kinetic curves might be elucidated by the slow formation of Ni(0) species under our conditions. Although a concerted hydronickelation mechanism was proposed via ligand-to-ligand hydrogen transfer (LLHT) process in the Ni(0)/phosphine ligand catalyzed alkene hydroarylation reaction^50,52,61,62^, the zero order to alkene under our conditions rules out this concerted pathway. We thus hypothesized that a stepwise Ni(II)-H initiated process generated from the oxidative addition of *t*-amyl alcohol with in-situ generated Ni(0) species might be more possible in current reaction^43,46^.

To identify the active Ni(II)-H species [*t*-AmylO-Ni(II)-H or Ar-Ni(II)-H)], a control experiment with Ni(COD)_2_ was conducted with *cis*-anethol in the absence of arylboronic acid (Fig. 6e). No deuterium scrambling and *cis*/*trans* isomerization happened under those conditions, which might rule out the alkene insertion with *t*-AmylO-Ni(II)-H species, as the alkene insertion into nickel hydride complexes is often fast and reversible^46,47^. In addition, hydroarylation of unactivated alkene **8** under our optimal conditions gave the migratory product **9** with high regioselectivity, which further confirms the presence of Ar-Ni(II)-H species, similar to Zhu’s protocol^47^ (Fig. 6f). Based on the aforementioned mechanistic insights, we believed that the Ar-Ni(II)-H species might be responsible for this efficient hydroarylation reaction, which could be generated by the oxidative addition of *tert*-amyl alcohol and sequential transmetalation with arylboronic acid. Moreover, those outcomes are consistent with the excellent regioselectivity observed in the reaction, which might originate from the formation of the most stable alkyl metal complex after alkene insertion to the Ar-Ni(II)-H species.

We also conducted the KIE study (Fig. 6g) with *tert*-amyl alcohol, which indicates the O–H cleavage might be involved in the turnover-limiting step (*k*_H_/*k*_D_ = 3.2/1.0). The first order with the Ni(II)/**L14** catalyst, along with this KIE experiments indicated the oxidative addition of the alcohol with Ni(0) might be the turnover-limiting step. According to the aforementioned mechanistic experiments, a proposed reaction pathway was depicted in Fig. 6h. The Ar-Ni(II)-H (**Int-I**) species was generated via oxidative addition of *t*-amyl alcohol and subsequent transmetalation with aryl boronic acid. The following migratory insertion to internal alkene leads to the regioselective formation of intermediate **Int-II**, which delivered the product and regenerated the Ni(0) species via reductive elimination. Based on our mechanism studies, we hypothesize that the *β*-diketone ligand might be very important to stabilize the unstable nickel intermediates, such as *t*-AmylO-Ni^(II)^-H or Ar-Ni^(II)^-H etc., as a neutral bidentate dicarbonyl ligand (acacH)^63^. In particular, the Ar-Ni^(II)^-H could result in reduction of arylbronic acids via rapid reductive elimination, which has been confirmed by the detection of corresponding arenes with GC-MS. The longer life time of Ar-Ni^(II)^-H species might be responsible for the high efficiency. Moreover, the neutral bidentate coordination might also accelerate the reductive elimination of **Int-II** to afford the desired product, as the reductive elimination step has been proven as the turnover-limiting step in the directed Ni-catalyzed hydroarylation of unactivated alkenes^46^.

**Fig. 7:**
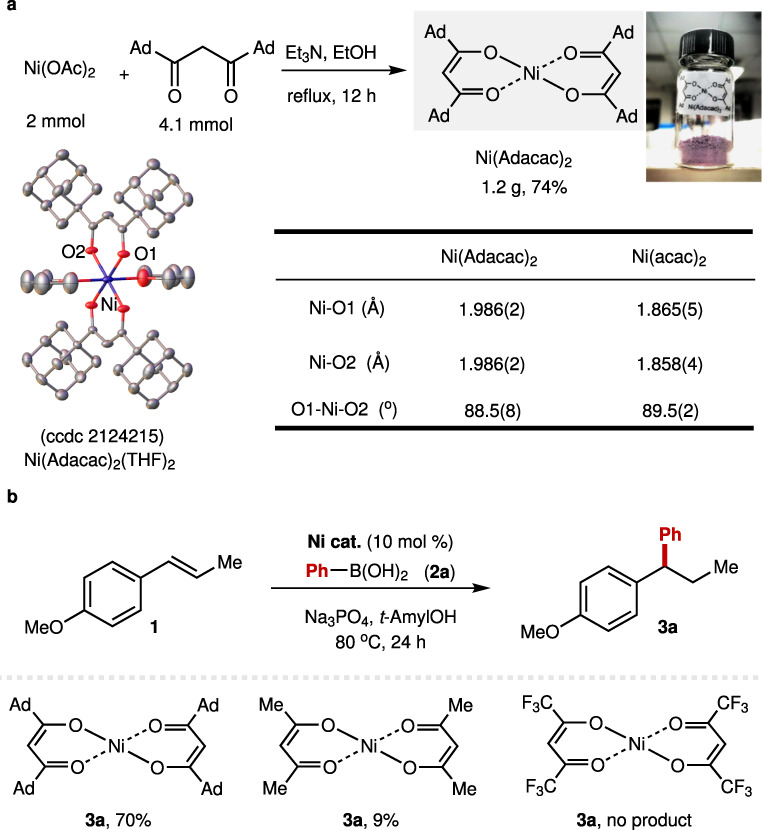
Synthesis of Ni(Adacac)_2_ complex and its reactivity. **a** Synthesis of Ni(Adacac)_2_ complex. **b** Reactivity of Ni(Adacac)_2_ in Ni-catalyzed hydroarylation reaction.

Inspired by the superior behavior of the 1-adamantyl *β*-diketone ligand (**L14**), we envisioned that the Ni(Adacac)_2_ might be an attractive nickel precursor for nickel catalysis as commercially available Ni(acac)_2_. Accordingly, the Ni(Adacac)_2_ was synthesized in 74% yield via the reaction of Ni(OAc)_2_ and 1-adamantyl *β*-diketone ligand (**L14**) in the presence of triethylamine (Fig. 7a). The structure of this complex was determined by the X-ray crystallography. The length of the Ni-O is longer than that in Ni(acac)_2_ (1.986 Å vs. 1.865 Å)^64^, which indicate a weaker coordinating ability in comparison with acac ligand. We next checked the efficiency of the Ni(Adacac)_2_ in the Ni-catalyzed hydroarylation of internal alkenes (Fig. 7b), and this Ni(II) precursor showed better reactivity than commercially available Ni(acac)_2_ and Ni(Hfacac)_2_.

In summary, the Ni-catalyzed hydroarylation and hydroalkenylation of internal alkenes with aryl or alkenyl boronic acids have been demonstrated using a *β*-diketone ligand under redox-neutral conditions. With the developed bulky 1-adamantly *β*-diketone ligand, a broad range of 1,2-disubstituted alkenes are converted to corresponding diarylated motifs. Further application of our Ni/*β*-diketone catalytic system in the functionalization of alkenes is underway in our laboratory.

## Methods

### General procedure for Ni-catalyzed hydroarylation and hydroalkenylation

In a nitrogen-filled glovebox, substrate **1** or **4** (0.2 mmol, 1.0 equiv), **2** or **6** (0.6 mmol, 3.0 equiv), Ni(2-NH_2_-5-MeC_6_H_3_SO_3_)_2_ or Ni(OTf)_2_ (10 mol%), **L14** (6.8 mg, 10 mol%), Na_3_PO_4_ (98.4 mg, 0.6 mmol, 3.0 equiv) were charged in a 10-mL tube. The tube was sealed using an open-top cap with PTFE cap liner, and moved outside of the glovebox, followed by the addition of *tert*-amyl alcohol (1.0 mL). The tube was sealed again with parafilm and heated to 80 °C for 24 h. After cooling to room temperature, the mixture was passed through a pad of silica gel with EtOAc as the eluent to remove the nickel and the insoluble precipitate. The resulting solution was concentrated, and the residue was purified by column chromatography on silica gel or preparative thin-layer chromatography as mentioned. Full experimental details and characterization of new compounds can be found in the Supplementary Methods.

## Supplementary information

Supplementary Information

## Data Availability

X-ray structural data of compound Ni(Adacac)_2_ (ccdc 2124215) is available free of charge from the Cambridge Crystallographic Data Center via www.ccdc.cam.ac.uk/data_request/cif.
