# Supplementary material for: Ligand-enabled Ni-catalyzed hydroarylation and hydroalkenylation of internal alkenes with organoborons
Source: Nat Commun. 2022 Nov 12;13:6878. doi: 10.1038/s41467-022-34675-x (PMC9653468; doi:10.1038/s41467-022-34675-x)
Supplement: Supplementary file 1 — Supplementary Information [file 41467_2022_34675_MOESM1_ESM.pdf]

Supplementary Information

for

Ligand-Enabled Ni-Catalyzed Hydroarylation and Hydroalkenylation of Internal Alkenes with Organoborons

Dao-Ming Wang,[1] Li-Qin She,[1] Yichen Wu[1], Chunyin Zhu[2], and Peng Wang*[1,3,4]

[1]*State Key Laboratory of Organometallic Chemistry, Shanghai Institute of Organic Chemistry, CAS 345 Lingling Road, Shanghai 200032, P.R. China*

[2]*School of Chemistry and Chemical Engineering, Jiangsu University, Zhenjiang 212013, P.R. China*

[3]*CAS Key Laboratory of Energy Regulation Materials, Shanghai Institute of Organic Chemistry, CAS 345 Lingling Road, Shanghai 200032, P.R. China*

[4]*School of Chemistry and Materials Science, Hangzhou Institute for Advanced Study, University of Chinese Academy of Sciences, 1 Sub-lane Xiangshan, Hangzhou 310024, P.R. China*

* Email: pengwang@sioc.ac.cn

Supplementary Methods

1. General Information

Ni(2-NH_2-5-Me$C_6H_3SO_3$)$_2$ were purchased from Alfa Aesar. Other reagents were purchased from TCI, Sigma-Aldrich, Acros, Adamas-beta, J&K, 9-Ding, Bidepharm and Energy Chemical of the highest purity grade and used without further purification, unless otherwise indicated. Tetrahydrofuran (THF), acetonitrile (CH_3CN), dichloromethane (CH_2Cl_2) and *N*, *N*-dimethylformamide (DMF) were dried using the solvent purification system, and stored in the glovebox before use. 2-Methyl-2-butanol (*t*-AmylOH) was dried using sodium. Other anhydrous solvents were purchased from J&K. The extent of reaction was monitored by thin–layer chromatography (TLC), performed on 0.25 mm silica gel HSGF254. The TLC plates were visualized by ultraviolet light (254 nm) or treatment with potassium permanganate stain followed by gentle heating.

NMR spectra were recorded on Varian 400, Bruker 400 and Agilent 400 (400 MHz for ^{1}H; 375 MHz for ^{19}F; 100 MHz for ^{13}C) spectrometer. The chemical shifts (δ) were quoted in parts per million (ppm) referenced to TMS (0.0 ppm for ^{1}H NMR) and $CDCl_3$ (77.0 ppm for ^{13}C NMR). The following abbreviations were used to explain multiplicities: s = singlet, d = doublet, t = triplet, q = quartet, m = multiplet and br = broad. Coupling constants, *J*, were reported in Hertz unit (Hz). High-resolution mass spectra (HRMS) were recorded on an Agilent Mass spectrometer and Thermo Fisher Scientific LTQ FTICR-MS using ESI–TOF, DART or EI，The selectivity of the products was recorded on a Thermo Fisher gas chromatography-mass spectrometer (GC).

2. Experimental Section

2.1 Preparation of Substrates

Substrates **4a**[1], **4b**[1], **4c**[1], **4d**[13], **4e**[2], **4f**[3], **4g**[4], **4i**[5], **4k**[1], **4l**[6], **4m**[1], **4n**, **4o**[13], **4p**[7], **4r**[8], **4u**[9] were prepared according to the following Wittig-olefination procedure.

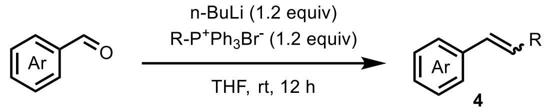

General Procedure A for the Synthesis of Internal Alkenes A suspension of the alkyltriphenylphosphonium bromide (1.1 equiv) and *t*-BuOK (2.0 equiv) in dry THF (0.5 M) was stirred at 0 °C under nitrogen for 30 min. Then, the solution of aldehyde (1.0 equiv) in THF (5.0 mL) was added dropwise over 5 min. The reaction mixture was stirred for 30 min at 0 °C, then allowed to warm up to room temperature overnight. The reaction mixture was quenched with water and extracted with Et_2O. The combined organic layers were dried over anhydrous Na_2SO_4 and concentrated under vacuum. The crude reaction mixture was purified by flash chromatography on silica gel to afford the corresponding internal alkene.

General Procedure B for the Synthesis of Internal Alkenes: To a suspension of the corresponding alkyltriphenylphosphonium bromide (1.2 equiv) in dry THF (0.5 M) at 0 °C under nitrogen was added *n*-butyllithium (1.2 equiv, 2.5 M in hexane) dropwise. After stirring at the same temperature for 30 min, the solution of the corresponding aldehyde (1.0 equiv) in THF (5.0 mL) was added dropwise over 5 min. The reaction mixture was stirred for 30 min at 0 °C, and was allowed to warm up to room temperature overnight. Upon completion, the reaction mixture was quenched with water and extracted with Et_2O. The combined organic layers were dried over anhydrous Na_2SO_4 and concentrated under vacuum. The crude reaction mixture was purified by flash chromatography on silica gel to afford the corresponding internal alkene

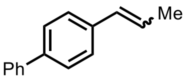

4a, ***E/Z*** **= 97/3**

4a was prepared according to general procedure A using 4-biphenylcarboxaldehyde (1.82 g, 10.0 mmol, 1.0 equiv), ethyltriphenylphosphonium bromide (4.08 g, 11 mmol, 1.1 equiv), *t*-BuOK (2.24 g, 20 mmol, 2.0 equiv) in dry THF (20 mL). Purification by flash chromatography on silica gel (PE/EA = 10/1) gave a mixture of alkenes as a white solid (760.9 mg, 40% yield, *E/Z* = 97/3). ^1H NMR (400 MHz, $CDCl_3$) δ 7.64–7.56 (m, 2H), 7.56–7.49 (m, 2H), 7.46–7.36 (m, 4H), 7.36–7.28 (m, 1H), 6.44 (dd, *J* = 15.6, 1.8 Hz, 1H), 6.29 (dq, *J* = 15.8, 6.4 Hz, 0.97H), 5.90–5.74 (m, 0.03H), 1.95 (dd, *J* = 7.2, 1.6 Hz, 0.13H), 1.91 (dd, *J* = 6.6, 1.6 Hz, 2.9H).

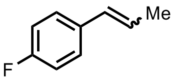

4b, ***E/Z*** **= 79/21**

4b was prepared according to general procedure B using 4-fluorobenzaldehyde (1.24 g, 10.0 mmol, 1.0 equiv),

ethyltriphenylphosphonium bromide (4.5 g, 12 mmol, 1.2 equiv), *n*-butyllithium (4.8 mL, 2.5 M in hexane, 12 mmol, 1.2 equiv) in dry THF (20 mL). Purification by flash chromatography on silica gel (PE) gave a mixture of alkenes as a colourless oil (671 mg, 49% yield, *E/Z* = 79/21). ^{1}H NMR (400 MHz, $CDCl_3$) δ 7.30–7.26 (m, 2H), 7.07–6.84 (m, 2H), 6.42–6.28 (m, 1H), 6.23–6.05 (m, 0.79H), 5.82–5.71 (dm, 0.21H), 1.89–1.82 (m, 3H).

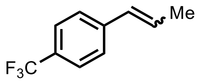

4c, ***E/Z* = 99/1**

4c was prepared according to general procedure A using 4-(trifluoromethyl)benzaldehyde (1.74 g, 10.0 mmol, 1.0 equiv), ethyltriphenylphosphonium bromide (4.08 g, 11 mmol, 1.1 equiv), *t*-BuOK (2.24 g, 20 mmol, 2.0 equiv) in dry THF (20 mL). Purification by flash chromatography on silica gel (PE) gave a mixture of alkenes as a colourless oil (583.3 mg, 31% yield, *E/Z* = 99/1). ^{1}H NMR (400 MHz, $CDCl_3$) δ 7.53 (d, *J* = 8.2 Hz, 2H), 7.40 (d, *J* = 8.2 Hz, 2H), 6.51–6.22 (m, 2H), 1.91 (dd, J = 6.4, 1.2 Hz, 3H).

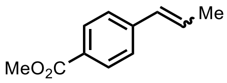

4d, ***E/Z* = 33/67**

4d was prepared according to general procedure B using methyl 4-formylbenzoate (1.64 g, 10.0 mmol, 1.0 equiv), ethyltriphenylphosphonium bromide (3.9 g, 10.5 mmol, 1.05 equiv), *n*-butyllithium (4.2 mL, 2.5 M in hexane, 10.5 mmol, 1.05 equiv) in dry THF (20 mL). Purification by flash chromatography on silica gel (PE) gave a mixture of alkenes as a yellow liquid (1.14 g, 65% yield, *E/Z* = 33/67).^{1}H NMR (400 MHz, $CDCl_3$) δ 8.04–7.96 (m, 1.29H), 7.99–7.91 (m, 0.61H), 7.39–7.33 (m, 2H), 6.53–6.23 (m, 1.33H), 5.90 (dq, J = 11.6, 7.2 Hz, 0.67H), 3.91 (s, 1.94H), 3.90 (s, 1.02H), 1.93–1.88 (m, 2H).

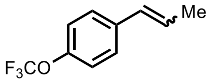

4e, ***E/Z* = 52/48**

4e was prepared according to general procedure B using 4-(trifluoromethoxy)benzaldehyde (1.64 g, 10.0 mmol, 1.0 equiv), ethyltriphenylphosphonium bromide (4.5 g, 12 mmol, 1.2 equiv), *n*-butyllithium (4.8 mL, 2.5 M in hexane, 12 mmol, 1.2 equiv) in dry THF (20 mL). Purification by flash chromatography on silica gel (PE) gave a mixture of alkenes as a colourless oil (1.76 g, 87% yield, *E/Z* = 52/48).^{1}H NMR (400 MHz, $CDCl_3$) δ 7.33–7.25 (m, 2H), 7.18–7.14 (m, 1H), 7.13–7.09 (m, 1H), 6.44–6.30 (m, 1H), 6.20 (dq, *J* = 15.8, 6.4 Hz, 0.52H), 5.81 (dq, *J* = 11.6, 7.2 Hz, 0.48H), 1.86 (dd, J = 7.0, 1.8 Hz, 3H).

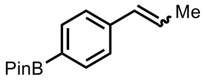

4f, ***E/Z* = 62/38**

4f was prepared according to general procedure B using 4-formylphenylboronic acid pinacol cyclic ester (2.32 g, 10.0 mmol, 1.0 equiv), ethyltriphenylphosphonium bromide (4.5 g, 12 mmol, 1.2 equiv), *n*-butyllithium (4.8 mL, 2.5 M in hexane, 12 mmol, 1.2 equiv) in dry THF (20 mL). Purification by flash chromatography on silica gel (PE/EA = 20/1) gave a mixture of alkenes as a colourless oil (2.15 g, 88% yield, *E/Z* = 62/38).^{1}H NMR (400 MHz, $CDCl_3$) δ

7.78 (d, J = 7.6 Hz, 0.8H), 7.73 (d, J = 7.6 Hz, 1.13H), 7.35–7.27 (m, 2H), 6.51–6.36 (m, 1H), 6.31 (dq, J = 15.8, 6.4 Hz, 1H), 5.82 (dq, J = 11.4, 7.2 Hz, 0H), 1.93–1.82 (m, 3H), 1.36–1.30 (m, 12H).

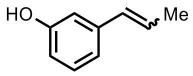

4g, ***E/Z*** **= 69/31**

4g was prepared according to general procedure B using 3-hydroxybenzaldehyde (3.66 g, 30.0 mmol, 1.0 equiv), ethyltriphenylphosphonium bromide (22.3 g, 60 mmol, 2.0 equiv), *n*-butyllithium (24 mL, 2.5 M in hexane, 60 mmol, 2.0 equiv) in dry THF (60 mL). Purification by flash chromatography on silica gel (PE/EA = 10/1) gave a mixture of alkenes as a colourless oil (3.44 g, 86% yield, *E/Z* = 69/31). ^{1}H NMR (400 MHz, $CDCl_3$) δ 7.27–7.08 (m, 1H), 6.93–6.84 (m, 1H), 6.82–6.76 (m, 1H), 6.73–6.62 (m, 1H), 6.40–6.29 (m, 1H), 6.20 (dq, J = 15.8, 6.4 Hz, 0.69H), 5.78 (dq, J = 11.6, 7.2 Hz, 0.31H), 5.29 (s, 1H), 1.92–1.83 (t, J = 8.0 Hz, 3H).

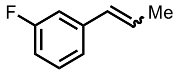

4i, ***E/Z*** **= 55/45**

4i was prepared according to general procedure B using 3-fluorobenzaldehyde (1.24 g, 10.0 mmol, 1.0 equiv), ethyltriphenylphosphonium bromide (4.5 g, 12 mmol, 1.2 equiv), *n*-butyllithium (4.8 mL, 2.5 M in hexane, 12 mmol, 1.2 equiv) in dry THF (20 mL). Purification by flash chromatography on silica gel (PE) gave a mixture of alkenes as a colourless oil (1.0 g, 76% yield, *E/Z* = 55/45). ^{1}H NMR (400 MHz, $CDCl_3$) δ 7.36–7.16 (m, 1H), 7.10–6.97 (m, 2H), 6.94–6.84 (m, 1H), 6.44–6.32 (m, 1H), 6.25 (dq, J = 15.8, 6.4 Hz, 0.55H), 5.83 (dq, J = 11.4, 7.2 Hz, 0.45H), 1.92–1.85 (m, 3H).

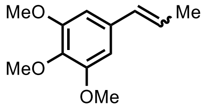

4k, ***E/Z*** **= 60/40**

4k was prepared according to general procedure A using 3,4,5-trimethoxybenzaldehyde (1.96 g, 10.0 mmol, 1.0 equiv), ethyltriphenylphosphonium bromide (4.08 g, 11 mmol, 1.1 equiv), *t*-BuOK (2.24 g, 20 mmol, 2.0 equiv) in dry THF (20 mL). Purification by flash chromatography on silica gel (PE/EA = 10/1) gave a mixture of alkenes as a colourless oil (1.38 g, 66 % yield, *E/Z* = 60/40). ^{1}H NMR (400 MHz, $CDCl_3$) δ 6.56 (s, 1.18H), 6.52 (s, 0.82H), 6.41–6.29 (m, 1H), 6.15 (dq, J = 15.8, 6.6 Hz, 0.60H), 5.76 (dq, J = 11.6, 7.2 Hz, 0.40H), 3.89–3.81 (m, 9H), 1.92 (dd, J = 7.2, 2.0 Hz, 1.24H), 1.88 (dd, J = 6.4, 1.6 Hz, 1.76H).

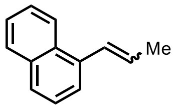

4l, ***E/Z*** **= 90/10**

4l was prepared according to general procedure A using 1-naphthaldehyde (1.56 g, 10.0 mmol, 1.0 equiv), ethyltriphenylphosphonium bromide (4.08 g, 11 mmol, 1.1 equiv), *t*-BuOK (2.24 g, 20 mmol, 2.0 equiv) in dry THF (20 mL). Purification by flash chromatography on silica gel (PE) gave a mixture of alkenes as a colourless oil (1.17

g, 70 % yield, *E/Z* = 90/10). ^{1}H NMR (400 MHz, $CDCl_3$) δ 8.12 (d, *J* = 7.6 Hz, 1H), 8.01–7.97 (m, 0.12H), 7.87–7.79 (m, 1.12H), 7.77–7.70 (m, 1.12H), 7.56–7.30 (m, 4.5H), 7.12 (d, *J* = 15.6 Hz, 1H), 6.90 (d, *J* = 11.4 Hz, 0.12H), 6.24 (dq, *J* = 15.6, 6.6 Hz, 1H), 6.04 (dq, *J* = 11.6, 6.8 Hz, 0.12H), 2.00 (dd, *J* = 6.8, 1.6 Hz, 3.0H), 1.75 (dd, *J* = 7.0, 1.6 Hz, 0.36H).

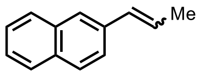

4m, ***E/Z* = 98/2**

4m was prepared according to general procedure A using 2-naphthaldehyde (1.56 g, 10.0 mmol, 1.0 equiv), ethyltriphenylphosphonium bromide (4.08 g, 11 mmol, 1.1 equiv), *t*-BuOK (2.24 g, 20 mmol, 2.0 equiv) in dry THF (20 mL). Purification by flash chromatography on silica gel (PE) gave a mixture of alkenes as a colourless oil (1.28 g, 76 % yield, *E/Z* = 98/2). ^{1}H NMR (400 MHz, $CDCl_3$) δ 7.81– 7.74 (m, 3H), 7.65 (s, 1H), 7.59–7. 53 (m, 1H), 7.48–7.34 (m, 2H), 6.56 (dd, *J* = 15.8, 2.0 Hz, 1H), 6.37 (dq, *J* = 15.8, 6.6 Hz, 0.98H), 5.98 – 5.79 (m, 0.02H), 2.00–1.96 (m, 0.6H), 1.94 (dd, *J* = 6.6, 1.6 Hz, 3H).

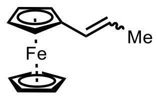

4n (*E/Z* = 18:82)

4n was prepared according to general procedure B using ferrocenecarboxaldehyde (1.5 g, 10.0 mmol, 1.0 equiv), ethyltriphenylphosphonium bromide (4.08 g, 11 mmol, 1.1 equiv), *t*-BuOK (2.24 g, 20 mmol, 2.0 equiv) in dry THF (20 mL). Purification by flash chromatography on silica gel (PE) gave a mixture of alkenes as a yellow liquid (1.94 g, 86% yield, *E/Z* = 18/82). ^{1}H NMR (400 MHz, $CDCl_3$) δ 6.09 (dd, *J* = 11.2, 1.6 Hz, 1.22H), 5.80 (dq, *J* = 15.6, 6.6 Hz, 0.22H), 5.56 (dq, *J* = 11.4, 7.2 Hz, 1H), 4.33 (t, *J* = 2.0 Hz, 2H), 4.26 (t, *J* = 2.0 Hz, 0.46H), 4.19 (t, *J* = 1.8 Hz, 2H), 4.14 (t, *J* = 2.0 Hz, 0.48H), 4.10 (s, 5H), 4.09 (s, 1.2H), 1.82 (dd, *J* = 7.2, 1.6 Hz, 3H), 1.73 (dd, *J* = 6.4, 1.6 Hz, 0.67H); ^{13}C NMR (100 MHz, $CDCl_3$) δ 127.61, 126.83, 123.49, 122.89, 82.33, 69.03, 68.98, 68.94, 68.84, 68.18, 68.03, 66.14, 18.47, 14.80; HRMS (ESI-TOF) *m/z* Calcd for $C_{13}H_{14}Fe$ $[M]^+$: 226.0439, found: 226.0438.

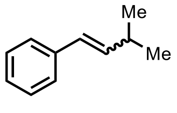

4o,*E/Z* = 28/72

4o was prepared according to general procedure B using benzaldehyde (530.6 mg, 5.0 mmol, 1.0 equiv), isopropyltriphenylphosphonium bromide (2.2 g, 5.5 mmol, 1.1 equiv), *n*-butyllithium (2.2 mL, 2.5 M in hexane, 5.5 mmol, 1.1 equiv) in dry THF (10 mL). Purification by flash chromatography on silica gel (PE) gave a mixture of alkenes as a colourless oil (570.3 mg, 78% yield, *E/Z* = 28/72). ^{1}H NMR (400 MHz, $CDCl_3$) δ 7.48–7.10 (m, 5H), 6.38–6.26 (m, 1H), 6.19 (ddd, *J* = 15.8, 6.6, 2.0 Hz, 0.28H), 5.47 (ddd, *J* = 11.8, 10.4, 2.2 Hz, 0.72H), 2.97–2.82 (m, 0.72H), 2.57–2.28 (m, 0.28H), 1.12–1.07 (m, 1.68H), 1.06–1.02 (m, 4.32H).

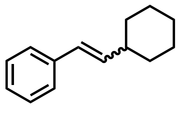

4p, ***E/Z* = 70/30**

4p was prepared according to general procedure B using cyclohexanecarboxaldehyde (560.8 mg, 5.0 mmol, 1.0 equiv), benzyltriphenylphosphonium bromide (2.4 g, 5.5 mmol, 1.1 equiv), *n*-butyllithium (2.2 mL, 2.5 M in hexane, 5.5 mmol, 1.1 equiv) in dry THF (10 mL). Purification by flash chromatography on silica gel (PE) gave a mixture of alkenes as a colourless oil (902.5 mg, 97% yield, *E/Z* = 70/30). ^{1}H NMR (400 MHz, $CDCl_3$) δ 7.37–7.09 (m, 5H), 6.39–6.27 (m, 1H), 6.17 (dd, *J* = 16.0, 6.8 Hz, 0.7H), 5.48 (dd, *J* = 11.6, 10.4 Hz, 0.30H), 2.64–2.51 (m, 0.30H), 2.20–2.03 (m, 0.70H), 1.83–1.58 (m, 5H), 1.40–1.08 (m, 5H).

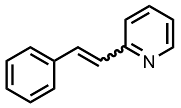

4r, ***E/Z* = 60/40**

4r was prepared according to general procedure B using 2-pyridinecarboxaldehyde (1.07 g, 10.0 mmol, 1.0 equiv), benzyltriphenylphosphonium bromide (5.2 g, 12 mmol, 1.2 equiv), *n*-butyllithium (4.8 mL, 2.5 M in hexane, 12 mmol, 1.2 equiv) in dry THF (20 mL). Purification by flash chromatography on silica gel (PE/EA = 10/1) gave a mixture of alkenes as a yellow solid (1.67 g, 92% yield, *E/Z* = 60/40). ^{1}H NMR (400 MHz, $CDCl_3$) δ 8.62–8.55 (m, 1H), 7.67–7.61 (m, 1.3H), 7.60–7.55 (m, 1.30H), 7.45–7.33 (m, 2.50H), 7.31–7.21 (m, 2.8H), 7.17–7.10 (m, 1.30H), 7.09–7.04 (m, 0.40H), 6.82 (d, *J* = 12.4 Hz, 0.40H), 6.69 (d, *J* = 12.4 Hz, 0.40H).

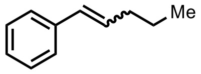

4u, ***E/Z* = 56/44**

4u was prepared according to general procedure B using benzaldehyde (1.07 g, 10.0 mmol, 1.0 equiv), *n*-butyltriphenylphosphonium bromide (4.2 g, 10.5 mmol, 1.05 equiv), *n*-butyllithium (4.2 mL, 2.5 M in hexane, 10.5 mmol, 1.05 equiv) in dry THF (20 mL). Purification by flash chromatography on silica gel (PE) gave a mixture of alkenes as a colourless oil (1.12 g, 77% yield, *E/Z* = 56/44). ^{1}H NMR (400 MHz, $CDCl_3$) δ 7.37–7.26 (m, 4H), 7.24–7.14 (m, 1H), 6.49–6.33 (m, 1H), 6.23 (dt, *J* = 15.8, 6.8 Hz, 0.56H), 5.67 (dt, *J* = 11.8, 7.2 Hz, 0.44H), 2.36–2.27 (m, 0.88H), 2.19 (q, *J* = 7.2 Hz, 1.12H), 1.59–1.44 (m, 2H), 1.06–0.88 (m, 3H).

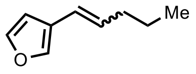

4x (*E/Z* = 36/64)

4x was prepared according to general procedure B using 3-furaldehyde (0.48 g, 5.0 mmol, 1.0 equiv), *n*-butyltriphenylphosphonium bromide (2.2 g, 5.5 mmol, 1.1 equiv), *n*-butyllithium (2.2 mL, 2.5 M in hexane, 5.5 mmol, 1.1 equiv) in dry THF (10 mL). Purification by flash chromatography on silica gel (PE) gave a mixture of alkenes as a colourless oil (0.43 g, 64% yield, *E/Z* = 36/64). ^{1}H NMR (400 MHz, $CDCl_3$) δ 7.43 (s, 0.51H), 7.40–7.36 (m, 0.50H), 7.34 (s, 0.59H), 6.50 (s, 0.31H), 6.46 (s, 0.53H), 6.22 (d, *J* = 15.8 Hz, 0.32H), 6.15 (d, *J* = 11.4 Hz, 0.55H), 5.94 (dt, *J* = 15.6, 7.0 Hz, 0.33H), 5.57 (dt, *J* = 11.4, 7.2 Hz, 0.56H), 2.25 (qd, *J* = 7.4, 1.8 Hz, 1.2H), 2.13 (q, *J* = 7.2 Hz, 0.7H), 1.48 (tq, *J* = 12.4, 6.9 Hz, 2H), 0.95 (dt, *J* = 10.2, 7.4 Hz, 3H); ^{13}C NMR (100 MHz, $CDCl_3$) δ 143.25, 142.58, 140.75, 139.29, 131.99, 130.59, 124.53, 122.64, 119.36, 118.71, 110.98, 107.53, 34.98, 31.25, 22.72, 22.52, 13.88, 13.70; HRMS (EI) *m/z* Calcd for $C_9H_{12}O$ $[M]^+$: 136.0883, found: 136.0884.

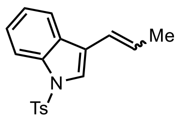

4y (*E/Z* = 50:50)

4y was prepared according to general procedure B using 1-tosyl-1*H*-indole-3-carbaldehyde (1.5 g, 5.0 mmol, 1.0 equiv), ethyltriphenylphosphonium bromide (2.2 g, 6.0 mmol, 1.2 equiv), *n*-butyllithium (2.4 mL, 2.5 M in hexane, 6.0 mmol, 1.2 equiv) in dry THF (20 mL). Purification by flash chromatography on silica gel (PE/EA = 10/1) gave a mixture of alkenes as a white solid (1.47 g, 94% yield, *E/Z* = 50/50). ^{1}H NMR (400 MHz, $CDCl_3$) δ 8.00–7.94 (m, 1H), 7.79–7.71 (m, 2H), 7.68 (d, *J* = 8.0 Hz, 0.5H), 7.54 (s, 1H), 7.53–7.48 (m, 1H), 7.35–7.27 (m, 1H), 7.26–7.21 (m, 1H), 7.20–7.15 (m, 2H), 6.53–6.36 (m, 1H), 6.34–6.20 (m, 0.5H), 5.93 (dq, *J* = 11.4, 7.2 Hz, 0.5H), 2.31–2.27 (m, 3H), 1.94–1.88 (m, 3H); ^{13}C NMR (100 MHz, $CDCl_3$) δ 144.87, 144.82, 135.45, 135.14, 134.53, 130.79, 129.81, 129.79, 129.30, 128.44, 127.37, 126.74, 126.73, 124.81, 124.71, 123.52, 123.29, 123.19, 122.52, 121.32, 121.10, 120.27, 119.53, 119.09, 118.70, 113.70, 113.53, 21.49, 18.91, 15.63; HRMS (EI) *m/z* Calcd for $C_{18}H_{17}NO_2S$ $[M]^+$: 311.0975, found: 311.0983.

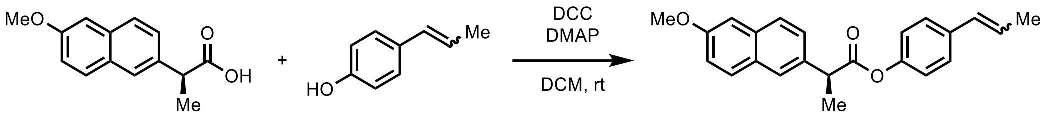

A Schlenk flask was charged with the naproxen (268.2 mg, 2.0 mmol, 1.0 equiv), DCC (618.9 mg, 3.0 mmol, 1.5 equiv), DMAP (244.3 mg, 2.0 mmol, 1.0 equiv) and anhydrous DCM (10 mL) under N_2, followed by addition of **S1** (506.7 mg, 2.2 mmol, 1.1 equiv, *E/Z* = 93/7). The reaction mixture was stirred at room temperature for 12 h. Upon completion, the solvent was removed under vacuum, and the crude residue was purified by silica gel column chromatography (PE/EA = 20/1 to 10/1) to afford the product **4z** as a white solid (307.9 mg, 44% yield, *E/Z* = 95/5). ^{1}H NMR (400 MHz, $CDCl_3$) δ 7.80–7.68 (m, 3H), 7.49 (dt, *J* = 8.4, 2.0 Hz, 1H), 7.27–7.22 (m, 2H), 7.18–7.09 (m, 2H), 7.00–6.82 (m, 2H), 6.34 (dt, *J* = 15.8, 1.8 Hz, 1H), 6.21–6.08 (m, 0.95H), 5.83–5.69 (m, 0.05H), 4.08 (qd, *J* = 7.2, 2.0 Hz, 1H), 3.92–3.91 (m, 3H), 1.84 (dt, *J* = 6.4, 1.8 Hz, 3H), 1.71–1.63 (m, 3H); ^{13}C NMR (100 MHz, $CDCl_3$) δ 173.20, 157.70, 149.52, 135.64, 135.15, 133.77, 130.02, 129.66, 129.30, 128.96, 127.33, 126.55, 126.11, 125.85, 121.32, 120.98, 119.07, 55.29, 45.54, 18.51, 18.41; HRMS (ESI-TOF) *m/z* Calcd for $C_{23}H_{22}O_3Na$ $[M+Na]^+$: 369.1461, found: 369.1454.

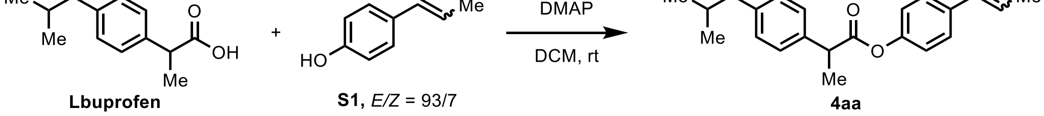

A Schlenk flask was charged with the lbuprofen (412.6 mg, 2.0 mmol, 1.0 equiv), EDCI (460.1 mg, 2.4 mmol, 1.2 equiv), DMAP (48.9 mg, 0.4 mmol, 0.2 equiv) and anhydrous DCM (10 mL) under N_2, followed by addition of **S1** (506.7 mg, 2.2 mmol, 1.1 equiv, *E/Z* = 93/7). The reaction mixture was stirred at room temperature for 12 h. Upon

completion, the solvent was removed under vacuum, and the crude residue was purified by silica gel column chromatography (PE/EA = 40/1) to afford the product **4aa** as a colourless oil (365.7 mg, 57% yield, *E/Z* = 92/8). ^{1}H NMR (400 MHz, $CDCl_3$) δ 7.32–7.21 (m, 4H), 7.16–7.08 (m, 2H), 6.96 (d, *J* = 8.6 Hz, 0.17H), 6.93–6.87 (m, 1.76H), 6.47–6.29 (m, 1H), 6.15 (dq, *J* = 15.8, 6.6 Hz, 0.92H), 5.76 (dq, *J* = 11.6, 7.2 Hz, 0.08H), 3.92 (q, *J* = 7.2 Hz, 1H), 2.46 (d, *J* = 7.2 Hz, 2H), 1.93–1.77 (m, 4H), 1.51–1.58 (m, 3H); ^{13}C NMR (100 MHz, $CDCl_3$) δ 173.23, 149.56, 140.75, 137.23, 135.58, 130.05, 129.64, 129.46, 127.18, 126.54, 125.80, 121.32, 120.99, 45.22, 45.01, 30.16, 22.37, 18.52, 18.42; HRMS (ESI-TOF) *m/z* Calcd for $C_{22}H_{26}O_2Na$ [M+Na]$^+$: 345.1825, found: 345.1820.

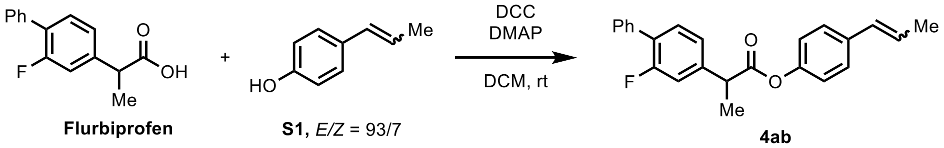

A Schlenk flask was charged with the flurbiprofen (488.5 mg, 2.0 mmol, 1.0 equiv), DCC (618.9 mg, 3.0 mmol, 1.5 equiv), DMAP (293.3 mg, 2.4 mmol, 1.2 equiv) and anhydrous DCM (10 mL) under N_2, followed by addition of **S1** (618.9 mg, 2.4 mmol, 1.2 equiv, *E/Z* = 93/7). The reaction mixture was stirred at room temperature for 12 h. Upon completion, the solvent was removed under vacuum, and the crude residue was purified by silica gel column chromatography (PE/EA = 20/1 to 10/1) to afford the product **4ab** as a colourless oil (278.9 mg, 39% yield, *E/Z* = 95/5). ^{1}H NMR (400 MHz, $CDCl_3$) δ 7.55 (d, *J* = 7.6 Hz, 2H), 7.46–7.40 (m, 3H), 7.37 (t, *J* = 7.4 Hz, 1H), 7.29 (d, *J* = 8.4 Hz, 2H), 7.26–7.18 (m, 2H), 7.00 (d, *J* = 8.2 Hz, 0.2H), 6.97–6.92 (m, 1.8H), 6.36 (d, *J* = 15.6 Hz, 1H), 6.25–6.08 (m, 0.95H), 5.84–5.67 (m, 0.05H), 3.98 (q, *J* = 7.2 Hz, 1H), 1.86 (dd, *J* = 6.8, 1.6 Hz, 3H), 1.64 (d, *J* = 7.2 Hz, 3H); ^{13}C NMR (100 MHz, $CDCl_3$) δ 172.49, 159.74 (d, *J* = 248.7 Hz), 149.38, 141.27 (d, *J* = 7.9 Hz), 135.81, 135.38, 130.97 (d, *J* = 4.0 Hz), 129.98, 128.93 (d, *J* = 3.0 Hz), 128.44, 127.70, 126.62, 126.02, 123.57 (d, *J* = 3.3 Hz), 121.26, 115.30 (d, *J* = 23.8 Hz), 45.11, 34.90, 18.44, 18.41; ^{19}F NMR (375 MHz, $CDCl_3$) δ -117.31; HRMS (ESI-TOF) *m/z* Calcd for $C_{24}H_{21}O_2FNa$ [M+Na]$^+$: 383.1418, found: 383.1424.

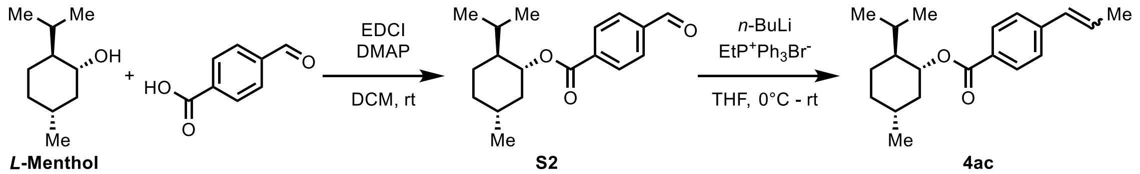

A Schlenk flask was charged with the *L*-menthol (781.5 mg, 5.0 mmol, 1.0 equiv), EDCI (1.15 g, 6.0 mmol, 1.2 equiv), DMAP (122.2 mg, 1.0 mmol, 0.2 equiv) and anhydrous DCM (20 mL) under N_2, followed by addition of *p*-hydroxybenzaldehyde (825.6 mg, 5.5 mmol, 1.1 equiv). The reaction mixture was stirred at room temperature for 12 h. Upon completion, the solvent was removed in vacuum, and the crude residue was purified by silica gel column chromatography (PE/EA = 20/1) to afford the product **S2** as a colourless oil (620.1 mg, 43% yield).

To a suspension of the ethyltriphenylphosphonium bromide (913.4 mg, 2.46 mmol, 1.2 equiv) in dry THF (10 mL) at 0 °C under nitrogen was added *n*-butyllithium (2.46 mmol, 2.5 M in hexane, 1.2 equiv) dropwise. After stirring at

the same temperature for 30 min, the solution of the corresponding aldehyde (590.1 mg, 2.05 mmol, 1.0 equiv) in THF (5.0 mL) was added dropwise over 5 min. The reaction mixture was stirred for 30 min at 0 °C, and was allowed to warm up to room temperature overnight. Upon completion, the reaction mixture was quenched with water and extracted with Et_2O. The combined organic layers were dried over anhydrous Na_2SO_4 and concentrated in vacuum. The crude reaction mixture was purified by flash chromatography on silica gel (PE/EA = 40/1) to afford the product **4ac** as a colourless oil (539.6 mg, 88% yield, *E/Z* = 23/77). ^{1}H NMR (400 MHz, $CDCl_3$) δ 8.04–7.99 (m, 1.51H), 7.96 (dd, *J* = 8.4, 1.6 Hz, 0.43H), 7.39–7.31 (m, 2H), 6.50–6.29 (m, 1.19H), 5.89 (dq, *J* = 11.7, 7.2 Hz, 0.73H), 4.92 (tt, *J* = 10.4, 5.0 Hz, 1H), 2.17–2.09 (m, 1H), 2.04–1.87 (m, 4H), 1.76–1.68 (m, 2H), 1.61–1.48 (m, 2H), 1.22–1.03 (m, 2H), 0.99–0.85 (m, 7H), 0.82–0.76 (m, 3H); ^{13}C NMR (100 MHz, $CDCl_3$) δ 165.96, 142.21, 142.07, 130.39, 129.83, 129.40, 129.16, 128.86, 128.66, 128.63, 128.53, 125.56, 74.68, 47.27, 40.97, 34.32, 31.42, 26.48, 23.63, 22.03, 20.75, 18.61, 16.51, 14.72; HRMS (ESI-TOF) *m/z* Calcd for $C_{20}H_{28}O_2Na$ $[M+Na]^+$: 323.1981, found: 323.1974.

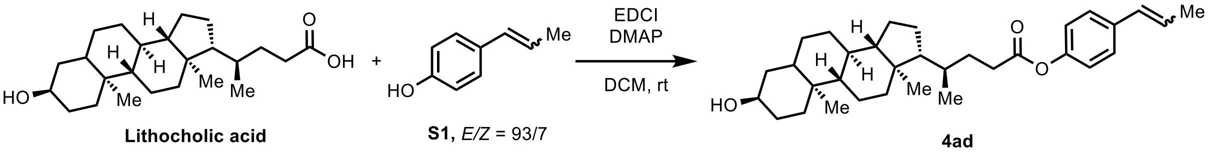

A Schlenk flask was charged with the lithochlic acid (753.2 mg, 2.0 mmol, 1.0 equiv), EDCI (460.1 mg, 2.4 mmol, 1.2 equiv), DMAP (48.9 mg, 0.4 mmol, 0.2 equiv) and anhydrous DCM (10 mL) under N_2, followed by addition of **S1** (483.6 mg, 2.1 mmol, 1.05 equiv, *E/Z* = 93/7). The reaction mixture was stirred at room temperature for 12 h. Upon completion, the solvent was removed in vacuum, and the crude residue was purified by silica gel column chromatography (PE/EA = 3/1) to afford the product **4ad** as a white solid (845.3 mg, 86% yield, *E/Z* = 94/6). ^{1}H NMR (400 MHz, $CDCl_3$) δ 7.31 (d, *J* = 8.2 Hz, 2H), 7.04 (d, *J* = 8.4 Hz, 0.12H), 6.99 (d, *J* = 8.6 Hz, 1.88H), 6.42–6.31 (m, 1H), 6.18 (dq, *J* = 15.4, 6.4 Hz, 0.94H), 5.87–5.71 (m, 0.06H), 3.67–3.55 (m, 1H), 2.59 (ddd, *J* = 14.8, 9.8, 4.8 Hz, 1H), 2.45 (ddd, *J* = 15.6, 8.8, 6.8 Hz, 1H), 2.01–1.70 (m, 9H), 1.72–1.54 (m, 3H), 1.54–1.21 (m, 12H), 1.19–1.02 (m, 5H), 1.01–0.97 (m, 4H), 0.92 (s, 3H), 0.66 (s, 3H); ^{13}C NMR (100 MHz, $CDCl_3$) δ 172.76, 149.45, 135.57, 130.05, 129.71, 126.61, 125.79, 121.46, 121.14, 71.77, 56.46, 55.92, 42.72, 42.04, 40.38, 40.13, 36.38, 35.80, 35.33, 35.31, 34.52, 31.35, 30.92, 30.48, 28.19, 27.15, 26.37, 24.16, 23.33, 20.78, 18.41, 18.28, 12.02; HRMS (ESI-TOF) *m/z* Calcd for $C_{33}H_{48}O_3Na$ $[M+Na]^+$: 515.3496, found: 515.3502.

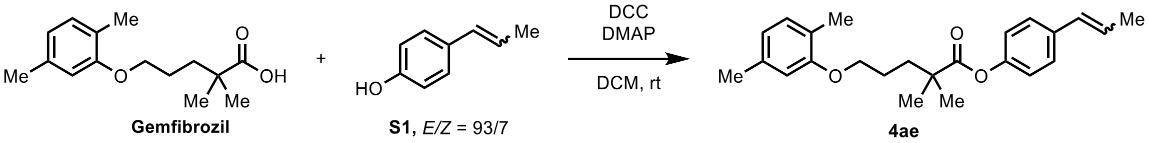

A Schlenk flask was charged with the gemfibrozil (250.3 mg, 1.0 mmol, 1.0 equiv), DCC (309.5 mg, 1.5 mmol, 1.5 equiv), DMAP (122.2 mg, 1.0 mmol, 1.0 equiv) and anhydrous DCM (10 mL) under N_2, followed by addition of **S1**

(161.1 mg, 1.2 mmol, 1.2 equiv, *E/Z* = 93/7). The reaction mixture was stirred at room temperature for 12 h. Upon completion, the solvent was removed in vacuum, and the crude residue was purified by silica gel column chromatography (PE/EA = 20/1) to afford the product **4ae** as a colourless oil (237.4 mg, 65% yield, *E/Z* = 98/2). ^{1}H NMR (400 MHz, $CDCl_3$) δ 7.34–7.28 (m, 2H), 7.00 (d, *J* = 7.4 Hz, 1H), 6.97–6.91 (m, 2H), 6.66 (d, *J* = 7.4 Hz, 1H), 6.62 (d, *J* = 1.8 Hz, 1H), 6.38 (dd, *J* = 15.8, 1.8 Hz, 1H), 6.18 (dq, *J* = 15.8, 6.6 Hz, 0.98H), 5.83–5.71 (m, 0.02H), 3.98 (p, *J* = 2.8 Hz, 2H), 2.30 (s, 3H), 2.17 (s, 3H), 1.90–1.84 (m, 7H), 1.36 (s, 6H); ^{13}C NMR (100 MHz, $CDCl_3$) δ 176.40, 156.85, 149.70, 136.46, 135.56, 130.31, 130.08, 126.62, 125.82, 123.60, 121.46, 120.70, 111.90, 67.75, 42.40, 37.13, 25.24, 25.14, 21.39, 18.45, 15.79; HRMS (ESI-TOF) *m/z* Calcd for $C_{24}H_{30}O_3Na$ $[M+Na]^+$: 389.2087, found: 389.2080.

2.2 Experimental Optimization

2.2.1 Base Effect[a]

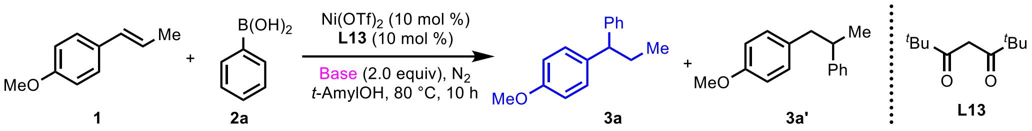

Entry	Base	3a (%)	3a' (%)	1 (%)	3a/3a'	Entry	Base	3a (%)	3a' (%)	1 (%)	3a/3a'
1	Li_2CO_3	--	--	76	--	11	Na_2HPO_4	--	--	74	--
2	Na_2CO_3	16	--	52	--	12	CsF	--	--	84	--
3	K_2CO_3	2	6	52	25/75	13	KF	--	--	76	--
4	Cs_2CO_3	--	--	64	--	14	CsOAc	--	--	84	--
5	$NaHCO_3$	--	--	82	--	15	NaOAc	--	--	86	--
6	$KHCO_3$	11	2	63	85/15	16	KOMe	--	--	84	--
7	$CsHCO_3$	3	6	64	33/67	17	KOtBu	--	--	84	--
8	K_3PO_4	3	4	66	43/57	18	CsOPiv	--	--	90	--
9	Na_3PO_4	31	2	36	94/6	19	KOH	--	--	80	--
10	NaH_2PO_4	--	--	82	--						

[a]Reaction conditions: **1** (0.1 mmol, 1.0 equiv), **2a** (0.2 mmol, 2.0 equiv), Base (0.2 mmol, 2.0 equiv), $Ni(OTf)_2$ (10 mol %), **L** (10 mol %), N_2, 80 °C, 10 h, *t*-AmylOH (0.5 mL). Yield was determined by ^{1}H NMR using CH_2Br_2 as an internal standard.

2.2.2 Evaluation of Ligands[a]

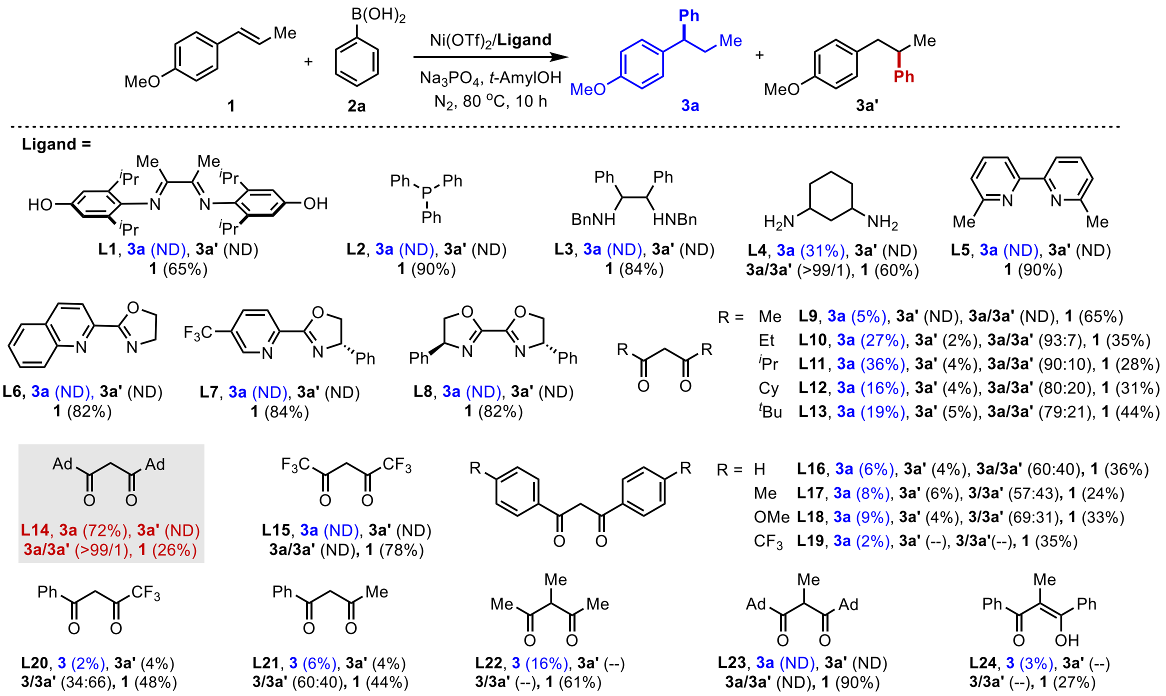

[a] Reaction conditions: 1 (0.1 mmol, 1.0 equiv), 2 (0.2 mmol, 2.0 equiv), $Ni(OTf)_2$ (10 mol%), Ligand (10 mol%), Na_3PO_4 (32.8 mg, 2.0 equiv), *t*-AmylOH (0.5 mL), 80 °C, N_2, 10 h. [b] Yield was determined by ^{1}H NMR using CH_2Br_2 as an internal standard.

2.2.3 Evaluation of Nickel Sources[a]

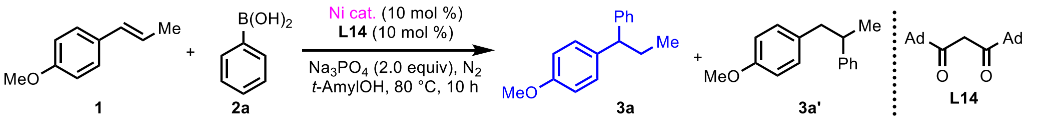

Entry	Ni	3a (%)	3a' (%)	1 (%)	3a/3a'	Entry	Ni	3a (%)	3a' (%)	1 (%)	3a/3a'
1	NiF_2	--	--	96	--	11	$Ni(HFacac)_2$	--	4	71	--
2	$NiCl_2$	--	--	99	--	12	$Ni(ClO_4)_2$•$6H_2O$	32	--	8	--
3	$NiBr_2$	60	2	31	97/3	13[b]	$Ni(ClO_4)_2$•$6H_2O$	30	--	63	--
4	NiI_2	--	--	100	--	14	$Ni(PhSO_3)_2$•$6H_2O$	64	2	29	97/3
5	$NiCl_2$•DME	--	--	97	--	15[b]	$Ni(PhSO_3)_2$•$6H_2O$	36	--	59	--
6	$NiBr_2$•DME	30	2	55	94/6	16	$Ni(TsO)_2$•$6H_2O$	66	--	29	--
7	$NiCl_2$•diglyme	26	--	63	--	17[b]	$Ni(TsO)_2$•$6H_2O$	32	--	53	--
8	$Ni(OAc)_2$	--	--	100	--	18	$Ni(BF_4)_2$•$6H_2O$	30	--	61	--
9	$Ni(COD)_2$	70	--	10	--	19	$Ni(2\text{-}NH_2\text{-}5\text{-}MeC_6H_3SO_3)_2$	80	--	9	--
10	$Ni(acac)_2$	58	2	33	97/3	20	$Ni(OTf)_2$	72	--	26	--

[a] Reaction conditions: **1** (0.1 mmol, 1.0 equiv), **2a** (0.2 mmol, 2.0 equiv), **Ni cat.** (10 mol %), **L14** (10 mol %), Na_3PO_4 (2.0 equiv), *t*-AmylOH (0.5 mL), 80 °C, N_2, 10 h. Yield was determined by ^{1}H NMR using CH_2Br_2 as an internal standard. [b] 20 mg 4Å MS was added.

2.2.4 Solvent Effect[a]

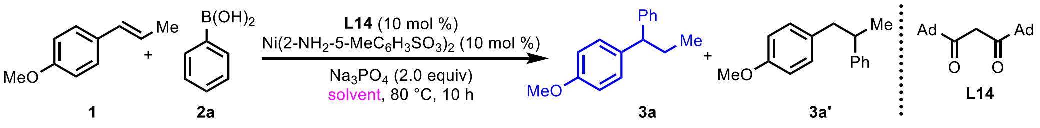

Entry	Solvent	3a (%)	3a' (%)	1 (%)	3a/3a'	Entry	Solvent	3a (%)	3a' (%)	1 (%)	3a/3a'
1	THF	29	--	64	--	7	EtOH	10	--	84	--
2	dioxane	62	--	38	--	8	*i*-PrOH	20	--	34	--
3	Toluene	30	--	52	--	9	*t*-BuOH	70	--	20	--
4	DMF	--	--	16	--	10	*t*-AmylOH	80	--	14	--
5	DMSO	--	--	24	--	11[b]	*t*-AmylOH	80	--	18	--
6	MeOH	--	--	100	--						

[a]Reaction conditions: **1** (0.1 mmol, 1.0 equiv), **2a** (0.2 mmol, 2.0 equiv), Na_3PO_4 (0.2 mmol, 2 equiv), $Ni(2\text{-}NH_2\text{-}5\text{-}MeC_6H_3SO_3)_2$ (10 mol %), **L14** (10 mol %), N_2, 80 °C, 10 h, solvent (0.5 mL). Yield was determined by ^{1}H NMR using CH_2Br_2 as an internal standard. [b] 24 h.

2.2.5 Loading of Arylboronic Acid and Base[a]

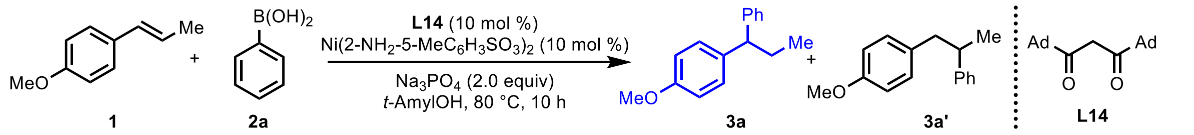

Entry	Condition	3a (%)	3a' (%)	1 (%)	3a/3a'
1	None	80	--	18	--
2	**2a** (1.2 equiv)	44	--	30	--
3	**2a** (1.5 equiv)	58	--	22	--
4	**2a** (2.0 equiv)	80		14	
5	**2a** (2.5 equiv)	76	--	14	--
6	**2a** (3.0 equiv)	76	--	6	--
7	**2a** (3.0 equiv), Na_3PO_4 (2.5 equiv)	84	--	8	--
8	**2a** (3.0 equiv), Na_3PO_4 (3.0 equiv)	92	--	--	--
9[b]	**2a** (3.0 equiv), Na_3PO_4 (3.0 equiv)	96 (91)[c]	--	--	--

[a] Reaction conditions: **1** (0.1 mmol, 1.0 equiv), **2a** (0.2 mmol, 2.0 equiv), Na_3PO_4 (0.2 mmol, 2 equiv), Ni(2-NH_2-5-$MeC_6H_3SO_3$)$_2$ (10 mol %), **L14** (10 mol %), N_2, 80 °C, 10 h, *t*-AmylOH (0.5 mL). Yield was determined by ^{1}H NMR using CH_2Br_2 as an internal standard. [b] **1** (0.2 mmol, 1.0 equiv), **2a** (0.6 mmol, 3.0 equiv), Na_3PO_4 (0.6 mmol, 3.0 equiv), Ni(2-NH_2-5-$MeC_6H_3SO_3$)$_2$ (10 mol %), **L14** (10 mol %), N_2, 80 °C, 10 h, *t*-AmylOH (1.0 mL). [c] Isolated yield.

2.2.6 Control Experiments[a]

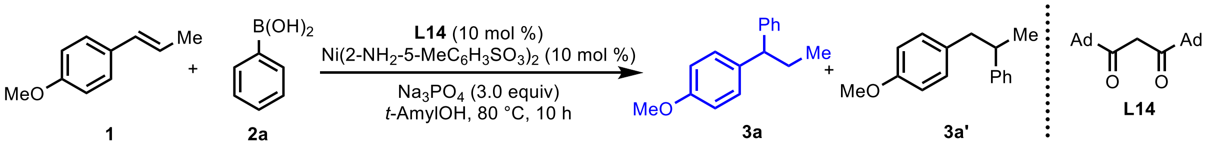

Entry	Condition	3a (%)	3a' (%)	1 (%)	3a/3a'
1	None	92	--	--	--
2	No **L14**	--	--	80	--
3	No **Base**	--	--	92	--
4	No **Ni**	--	--	86	--

[a]Reaction conditions: **1** (0.1 mmol, 1.0 equiv), **2a** (0.3 mmol, 3.0 equiv), Na_3PO_4 (0.3 mmol, 3 equiv), Ni(2-NH_2-5-$MeC_6H_3SO_3$)$_2$ (10 mol %), **L14** (10 mol %), N_2, 80 °C, 10 h, *t*-AmylOH (0.5 mL). Yield was determined by ^{1}H NMR using CH_2Br_2 as an internal standard.

2.2.7 The Reactivities of *cis*- and *trans*-Alkenes

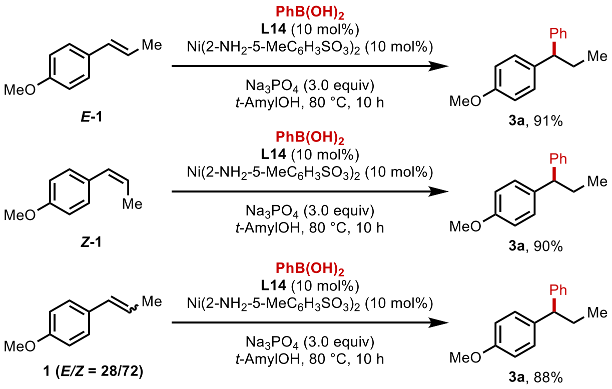

The experimental results unveiled that the configuration of the alkenes didn't affect the reactivity of this Ni-catalyzed hydroarylation reaction. The *E*-**1**, *Z*-**1**, and the mixture with a ratio of 28/72 (*E/Z*) all resulted in similar yields. Accordingly, we evaluated our reaction scope using the alkenes with the configurational mixtures.

2.2.8 The Reaction Rates of *Z*-Alkene and *E*-Alkene

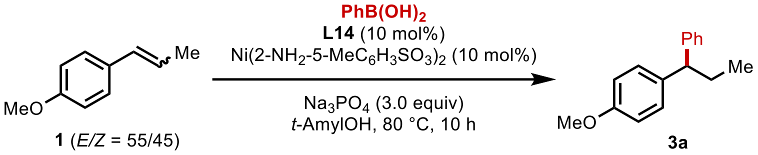

Experimental Procedure: In a nitrogen-filled glovebox, substrate **1** (Z/E = 55/45), **2a**, Ni(2-NH_2-5-$MeC_6H_3SO_3$)$_2$, **L14**, Na_3PO_4 were charged in a 10-mL tube, and added 3.0 μL dodecane as internal standard. The tube was sealed using an open-top cap with PTFE cap liner, and moved outside of the glovebox, followed by the addition of *tert*-amyl alcohol (1.0 mL). The tube was sealed again with parafilm and heated to 80 °C. The reaction progress was monitored by removing aliquots (~10 μL) from the reaction mixture via syringe under N_2. Each aliquot was quenched by EA (4.0 mL) in an 8.0 mL tube. The mixture was filtered with a filter head into 2.0 mL GC vial and analyzed by gas chromatography. The kinetic experiments with respect to the consumption of *E*-**1** and *Z*-**1** were investigated, indicating a similar reaction rate for *cis*- and *trans*- internal alkenes in our reaction.

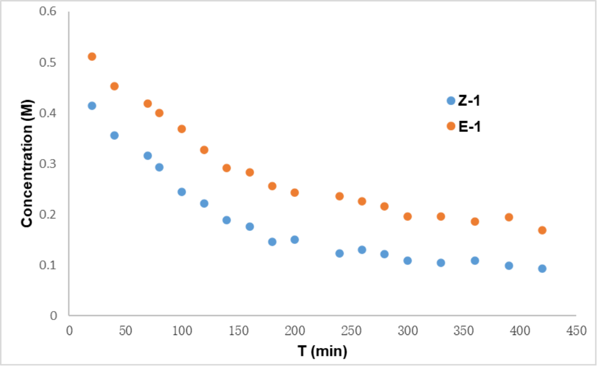

Supplementary Figure 1. Time-course profile for the reaction using the mixed **1** (*Z/E*=45:55)

2.3 General Procedure for Ni-Catalyzed Hydroarylation of Internal Alkenes

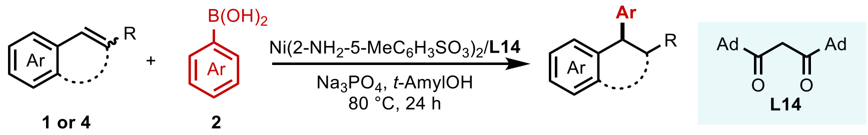

General Procedure: In a nitrogen-filled glovebox, substrate **1** or **4** (0.2 mmol, 1.0 equiv), **2** (0.6 mmol, 3.0 equiv), Ni(2-NH_2-5-Me$C_6H_3SO_3$)$_2$ (8.6 mg, 10 mol %), **L14** (6.8 mg, 10 mol %), Na_3PO_4 (98.4 mg, 0.6 mmol, 3.0 equiv) were charged in a 10-mL tube. The tube was sealed using an open-top cap with PTFE cap liner, and moved outside of the glovebox, followed by the addition of *tert*-amyl alcohol (1.0 mL). The tube was sealed again with parafilm and heated to 80 °C for 24 h. After cooling to room temperature, the mixture was passed through a pad of silica gel with EtOAc as the eluent to remove the nickel and the insoluble precipitate. The resulting solution was concentrated. The residue was then purified by silica gel chromatography or preparative thin-layer chromatography to afford the hydroarylated product **3** or **5**.

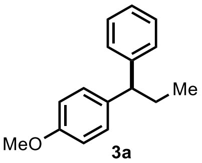

3a

1-Methoxy-4-(1-phenylpropyl)benzene[10]

3a was synthesized following the general procedure with *E*-**1**. After purification by preparative thin-layer chromatography using PE as the eluent, **3a** was obtained in 90% yield (40.6 mg) as a colourless liquid.

^{1}H NMR (400 MHz, $CDCl_3$) δ 7.34–7.17 (m, 4H), 7.17–7.10 (m, 3H), 6.81 (d, *J* = 8.8 Hz, 2H), 3.75 (s, 3H), 3.72 (t, *J* = 8.0 Hz, 1H), 2.0 –1.99 (m, 2H), 0.88 (t, *J* = 7.4 Hz, 3H); ^{13}C NMR (100 MHz, $CDCl_3$) δ 157.78, 145.55, 137.32, 128.75, 128.30, 127.79, 125.88, 113.70, 55.16, 52.35, 28.72, 12.77.

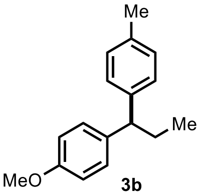

3b

1-Methoxy-4-(1-(*p*-tolyl)propyl)benzene[11]

3b was synthesized following the general procedure with *E*-**1**. After purification by preparative thin-layer chromatography using PE as the eluent, **3b** was obtained in 75% yield (35.8 mg) as a colourless liquid.

^{1}H NMR (400 MHz, $CDCl_3$) δ 7.16–7.03 (m, 6H), 6.84–6.77 (m, 2H), 3.74 (s, 3H), 3.70 (t, *J* = 7.8 Hz, 1H), 2.28 (s, 3H), 2.05–1.96 (m, 2H), 0.88 (t, *J* = 7.4 Hz, 3H); ^{13}C NMR (100 MHz, $CDCl_3$) δ 157.71, 142.54, 137.58, 135.30, 129.00, 128.68, 127.63, 113.68, 55.15, 51.93, 28.74, 20.93, 12.79.

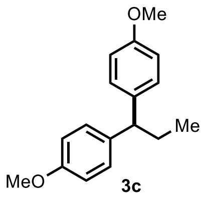

3c

4,4'-(Propane-1,1-diyl)bis(methoxybenzene)[12]

3c was synthesized following the general procedure with *E*-**1**. After purification by preparative thin-layer chromatography using PE as the eluent, **3c** was obtained in 73% yield (37.5 mg) as a colourless liquid.

^{1}H NMR (400 MHz, $CDCl_3$) δ 7.12 (d, *J* = 8.6 Hz, 4H), 6.81 (d, *J* = 8.8 Hz, 4H), 3.75 (s, 6H), 3.68 (t, *J* = 7.8 Hz, 1H), 2.04–1.95 (m, 2H), 0.87 (t, *J* = 7.2 Hz, 3H); ^{13}C NMR (100 MHz, $CDCl_3$) δ 157.70, 137.71, 128.64, 113.67, 55.15, 51.46, 28.87, 12.77.

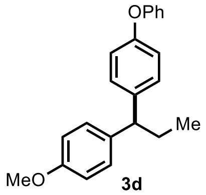

3d

4,4'-(Propane-1,1-diyl)bis(methoxybenzene)

3d was synthesized following the general procedure with *E*-**1**. After purification by preparative thin-layer chromatography using PE/EA (50/1) as the eluent, **3d** was obtained in 54% yield (34.4 mg) as a colourless liquid.

^{1}H NMR (400 MHz, $CDCl_3$) δ 7.34–7.26 (m, 2H), 7.15 (t, *J* = 8.4 Hz, 4H), 7.06 (t, *J* = 7.3 Hz, 1H), 6.97 (d, *J* = 8.0 Hz, 2H), 6.91 (d, *J* = 8.4 Hz, 2H), 6.83 (d, *J* = 8.8 Hz, 2H), 3.77 (s, 3H), 3.72 (t, *J* = 7.8 Hz, 1H), 2.05–1.98 (m, 2H), 0.89 (t, *J* = 7.2 Hz, 3H); ^{13}C NMR (100 MHz, $CDCl_3$) δ 157.80, 157.44, 155.12, 140.56, 137.31, 129.61, 128.92, 128.71, 122.93, 118.80, 118.61, 113.72, 55.17, 51.67, 28.88, 12.77; HRMS (EI) *m/z* Calcd for $C_{22}H_{22}O_2$ $[M]^+$: 318.1614, found: 318.1619.

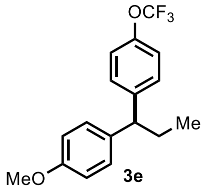

3e

1-Methoxy-4-(1-(4-(trifluoromethoxy)phenyl)propyl)benzene

3e was synthesized following the general procedure with *E*-**1**. After purification by preparative thin-layer chromatography using PE as the eluent, **3e** was obtained in 79% yield (48.9 mg) as a colourless liquid.

^{1}H NMR (400 MHz, $CDCl_3$) δ 7.26–7.18 (m, 2H), 7.14–7.05 (m, 4H), 6.83 (d, *J* = 8.8 Hz, 2H), 3.76 (s, 3H), 3.73 (t, *J* = 7.8 Hz, 1H), 2.05–1.96 (m, 1H), 0.88 (t, *J* = 7.4 Hz, 3H); ^{13}C NMR (100 MHz, $CDCl_3$) δ 157.99, 147.33 (d, *J* = 1.9 Hz), 144.33, 136.60, 128.97, 128.71, 120.82, 120.49 (d, *J* = 255.2 Hz), 113.84, 55.17, 51.70, 28.74, 12.67; ^{19}F NMR (375 MHz, $CDCl_3$) δ -57.90; HRMS (EI) *m/z* Calcd for $C_{17}H_{17}F_3O_2$ $[M]^+$: 310.1175, found: 310.1182.

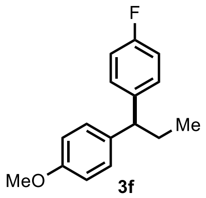

1-Fluoro-4-(1-(4-methoxyphenyl)propyl)benzene[11]

3f was synthesized following the general procedure with *E*-**1**. After purification by preparative thin-layer chromatography using PE as the eluent, **3f** was obtained in 92% yield (45.1 mg) as a colourless liquid.

^{1}H NMR (400 MHz, $CDCl_3$) δ 7.15 (dd, *J* = 8.4, 5.6 Hz, 2H), 7.11 (d, *J* = 8.6 Hz, 2H), 6.94 (t, *J* = 8.6 Hz, 2H), 6.82 (d, *J* = 8.8 Hz, 2H), 3.76 (s, 3H), 3.72 (t, *J* = 7.8 Hz, 1H), 2.06–1.93 (m, 2H), 0.87 (t, *J* = 7.4 Hz, 3H); ^{13}C NMR (100 MHz, $CDCl_3$) δ 161.17 (d, *J* = 243.8 Hz), 157.87, 141.23 (d, *J* = 3.2 Hz), 137.10, 129.10 (d, *J* = 7.6 Hz), 128.65, 115.02 (d, *J* = 21.2 Hz), 113.77, 55.17, 51.54, 28.84, 12.69; ^{19}F NMR (375 MHz, $CDCl_3$) δ -117.67.

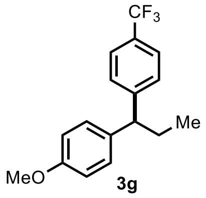

1-Methoxy-4-(1-(4-(trifluoromethyl)phenyl)propyl)benzene[13]

3g was synthesized following the general procedure with *E*-**1**. After purification by preparative thin-layer chromatography using PE as the eluent, **3g** was obtained in 80% yield (47.1 mg) as a colourless liquid.

^{1}H NMR (400 MHz, $CDCl_3$) δ 7.51 (d, *J* = 8.0 Hz, 2H), 7.32 (d, *J* = 8.0 Hz, 2H), 7.12 (d, *J* = 8.2 Hz, 2H), 6.83 (d, *J* = 9.2 Hz, 2H), 3.80 (t, *J* = 7.8 Hz, 1H), 3.77 (s, 3H), 2.09–1.99 (m, 1H)., 0.89 (t, *J* = 7.4 Hz, 3H); ^{13}C NMR (100 MHz, $CDCl_3$) δ 158.07, 149.67, 136.18, 128.74, 128.08, 127.00 (q, *J* = 271.0 Hz), 125.28 (q, *J* = 3.8 Hz), 113.90, 55.18, 52.19, 28.51, 12.63; ^{19}F NMR (375 MHz, $CDCl_3$) δ -62.32.

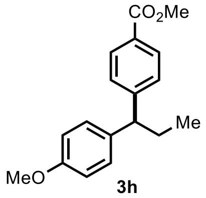

Methyl-4-(1-(4-methoxyphenyl)propyl)benzoate

3h was synthesized following the general procedure with *E*-**1**. $Ni(OTf)_2$ (10 mol%) instead of $Ni(2\text{-}NH_2\text{-}5\text{-}MeC_6H_3SO_3)_2$ as catalyst. After purification by preparative thin-layer chromatography using PE as the eluent, **3h** was obtained in 56% yield (32.0 mg) as a colourless liquid.

^{1}H NMR (400 MHz, $CDCl_3$) δ 7.97–7.91 (m, 2H), 7.31–7.25 (m, 2H), 7.16–7.09 (m, 2H), 6.86–6.79 (m, 2H), 3.88 (s, 3H), 3.79 (t, *J* = 8.0 Hz, 1H), 3.76 (s, 3H), 2.09–2.00 (m, 2H), 0.88 (t, *J* = 7.2 Hz, 3H); ^{13}C NMR (100 MHz, $CDCl_3$) δ 167.04, 157.99, 150.96, 136.30, 129.70, 128.73, 127.86, 127.81, 113.83, 55.15, 52.33, 51.91, 28.47, 12.63; HRMS (EI) *m/z* Calcd for $C_{18}H_{20}O_3$ $[M]^+$: 284.1407, found: 284.1414.

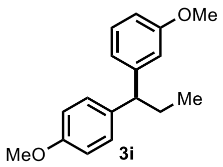

1-Methoxy-3-(1-(4-methoxyphenyl)propyl)benzene

3i was synthesized following the general procedure with *E*-**1**. After purification by preparative thin-layer chromatography using PE/EA (50/1) as the eluent, **3i** was obtained in 88% yield (45.0 mg) as a colourless liquid.

^{1}H NMR (400 MHz, $CDCl_3$) δ 7.22–7.08 (m, 3H), 6.85–6.77 (m, 3H), 6.77–6.76 (m, 1H), 6.69 (dd, *J* = 8.2, 2.4 Hz, 1H), 3.75 (s, 3H), 3.70 (t, *J* = 7.8 Hz, 1H), 2.07–1.96 (m, 2H), 0.88 (t, *J* = 7.4 Hz, 3H); ^{13}C NMR (100 MHz, $CDCl_3$) δ 159.53, 157.79, 147.24, 137.12, 129.21, 128.69, 120.26, 113.92, 113.69, 110.71, 55.14, 55.04, 52.36, 28.64, 12.76; HRMS (EI) *m/z* Calcd for $C_{17}H_{20}O_2$ $[M]^+$: 256.1461, found: 256.1460.

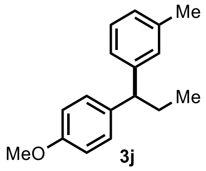

1-(1-(4-Methoxyphenyl)propyl)-3-methylbenzene

3j was synthesized following the general procedure with *E*-**1**. After purification by preparative thin-layer chromatography using PE/EA (50/1) as the eluent, **3j** was obtained in 86% yield (41.5 mg) as a colourless liquid.

^{1}H NMR (400 MHz, $CDCl_3$) δ 7.17–7.12 (m, 3H), 7.03–7.00 (m, 2H), 6.96 (d, *J* = 7.6 Hz, 1H), 6.86–6.76 (m, 2H), 3.75 (s, 3H), 3.69 (t, *J* = 8.0 Hz, 1H), 2.29 (s, 3H), 2.06–1.97 (m, 2H), 0.88 (t, *J* = 7.2 Hz, 3H); ^{13}C NMR (100 MHz, $CDCl_3$) δ 157.73, 145.47, 137.76, 137.42, 128.72, 128.61, 128.17, 126.66, 124.72, 113.67, 55.14, 52.32, 28.73, 21.49, 12.81; HRMS (EI) *m/z* Calcd for $C_{17}H_{20}O$ $[M]^+$: 240.1509, found: 240.1513.

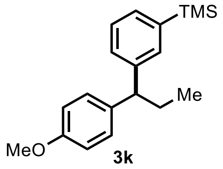

(3-(1-(4-Methoxyphenyl)propyl)phenyl)trimethylsilane

3k was synthesized following the general procedure with *E*-**1**. After purification by preparative thin-layer chromatography using PE/EA (50/1) as the eluent, **3k** was obtained in 62% yield (37.0 mg) as a colourless liquid.

^{1}H NMR (400 MHz, $CDCl_3$) δ 7.37 (s, 1H), 7.32 (dt, *J* = 7.2, 1.2 Hz, 1H), 7.24 (t, *J* = 4.0 Hz, 1H), 7.21–7.11 (m, 3H), 6.82 (d, *J* = 8.8 Hz, 2H), 3.76 (s, 3H), 3.74 (t, *J* = 7.6 Hz, 1H), 2.08–1.99 (m, 2H), 0.89 (t, *J* = 7.4 Hz, 3H), 0.24 (s, 9H); ^{13}C NMR (100 MHz, $CDCl_3$) δ 157.75, 144.61, 140.26, 137.27, 132.82, 130.94, 128.78, 128.13, 127.71, 113.69, 55.16, 52.54, 28.92, 12.82, -1.09; HRMS (EI) *m/z* Calcd for $C_{19}H_{26}OSi$ $[M]^+$: 298.1747, found: 298.1753.

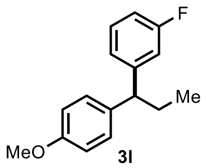

3l

1-Fluoro-3-(1-(4-methoxyphenyl)propyl)benzene

3l was synthesized following the general procedure with *E*-**1**. After purification by preparative thin-layer chromatography using PE/EA (50/1) as the eluent, **3l** was obtained in 91% yield (44.5 mg) as a colourless liquid.

^{1}H NMR (400 MHz, $CDCl_3$) δ 7.26–7.17 (m, 1H), 7.16–7.08 (m, 2H), 6.99 (dt, *J* = 7.6, 1.6 Hz, 1H), 6.91 (dt, *J* = 10.4, 2.0 Hz, 1H), 6.89–6.77 (m, 3H), 3.76 (s, 3H), 3.73 (t, *J* = 8.0 Hz, 1H), 2.05–1.97 (m, 2H), 0.88 (t, *J* = 7.2 Hz, 3H); ^{13}C NMR (100 MHz, $CDCl_3$) δ 162.90 (d, *J* = 245.1 Hz), 157.95, 148.28 (d, *J* = 6.7 Hz), 136.53, 129.66 (d, *J* = 8.3 Hz), 128.70, 123.48 (d, *J* = 2.7 Hz), 114.53 (d, *J* = 21.1 Hz), 113.80, 112.74 (d, *J* = 21.1 Hz), 55.16, 52.06 (d, *J* = 1.6 Hz), 28.57, 12.66; ^{19}F NMR (375 MHz, $CDCl_3$) δ -113.56; HRMS (EI) *m/z* Calcd for $C_{16}H_{17}OF$ $[M]^+$: 244.1258, found: 244.1264.

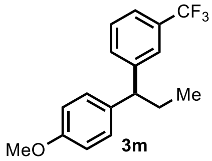

3m

1-(1-(4-Methoxyphenyl)propyl)-3-(trifluoromethyl)benzene[10]

3m was synthesized following the general procedure with *E*-**1**. After purification by preparative thin-layer chromatography using PE as the eluent, **3m** was obtained in 82% yield (48.0 mg) as a colourless liquid.

^{1}H NMR (400 MHz, $CDCl_3$) δ 7.48 (s, 1H), 7.45–7.32 (m, 3H), 7.16–7.08 (m, 2H), 6.88–6.79 (m, 2H), 3.79 (t, *J* = 8.0 Hz, 1H), 3.76 (s, 3H), 2.11–1.97 (m, 2H), 0.89 (t, *J* = 7.2 Hz, 3H); ^{13}C NMR (100 MHz, $CDCl_3$) δ 158.06, 146.55, 136.21, 131.23, 130.56 (q, *J* = 31.9 Hz), 128.76, 128.74, 124.42 (q, *J* = 3.8 Hz), 124.27 (q, *J* = 270.6 Hz), 122.85 (q, *J* = 3.9 Hz), 113.91, 55.17, 52.18, 28.64, 12.63; ^{19}F NMR (375 MHz, $CDCl_3$) δ -62.41.

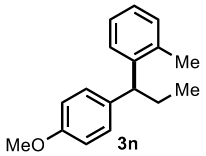

3n

1-(1-(4-Methoxyphenyl)propyl)-2-methylbenzene

3n was synthesized following the general procedure with *E*-**1**. After purification by preparative thin-layer chromatography using PE as the eluent, **3n** was obtained in 74% yield (35.8 mg) as a colourless liquid.

^{1}H NMR (400 MHz, $CDCl_3$) δ 7.32–7.26 (m, 1H), 7.23–7.13 (m, 1H), 7.12–7.06 (m, 4H), 6.82–6.75 (m, 2H), 3.92 (t, *J* = 7.6 Hz, 1H), 3.74 (s, 3H), 2.25 (s, 3H), 2.05–1.94 (m, 2H), 0.90 (t, *J* = 7.4 Hz, 3H); ^{13}C NMR (100 MHz, $CDCl_3$) δ 157.62, 143.19, 136.81, 136.28, 130.39, 129.08, 126.41, 125.91, 125.79, 113.55, 55.13, 47.87, 29.16, 19.86, 12.78; HRMS (EI) *m/z* Calcd for $C_{17}H_{20}O$ $[M]^+$: 240.1509, found: 240.1502.

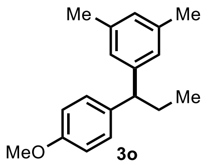

1-(1-(4-Methoxyphenyl)propyl)-3,5-dimethylbenzene[11]

3o was synthesized following the general procedure with *E*-**1**. After purification by preparative thin-layer chromatography using PE as the eluent, **3o** was obtained in 68% yield (34.7 mg) as a colourless liquid.

^{1}H NMR (400 MHz, $CDCl_3$) δ 7.18–7.11 (m, 2H), 6.85–6.78 (m, 5H), 3.76 (s, 3H), 2.26 (s, 6H), 2.05–1.95 (m, 2H), 0.87 (t, *J* = 7.2 Hz, 3H); ^{13}C NMR (100 MHz, $CDCl_3$) δ 157.70, 145.45, 137.64, 137.53, 128.71, 127.60, 125.58, 113.66, 55.16, 52.30, 28.75, 21.37, 12.85.

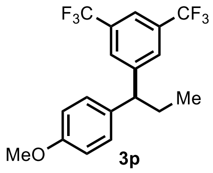

1-(1-(4-Methoxyphenyl)propyl)-3,5-bis(trifluoromethyl)benzene

3p was synthesized following the general procedure with *E*-**1**. After purification by preparative thin-layer chromatography using PE as the eluent, **3p** was obtained in 51% yield (37.0 mg) as a colourless liquid.

^{1}H NMR (400 MHz, $CDCl_3$) δ 7.69 (s, 1H), 7.65 (s, 2H), 7.15–7.09 (m, 2H), 6.89–6.83 (m, 2H), 3.87 (t, *J* = 7.8 Hz, 1H), 3.79 (s, 3H), 2.14–1.98 (m, 2H), 0.90 (t, *J* = 7.2 Hz, 3H); ^{13}C NMR (100 MHz, $CDCl_3$) δ 158.40, 148.23, 134.96, 131.54 (q, *J* = 33.0 Hz), 128.74, 127.88 (d, *J* = 3.2 Hz), 126.86, 123.43 (q, *J* = 272.6 Hz), 120.12 (p, *J* = 4.0 Hz), 114.19, 55.20, 52.09, 28.59, 12.52; ^{19}F NMR (375 MHz, $CDCl_3$) δ -62.76; HRMS (EI) *m/z* Calcd for $C_{18}H_{16}F_6O$ $[M]^+$: 362.1100, found: 362.1111.

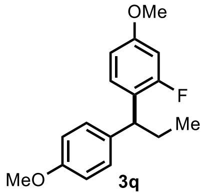

Fluoro-4-methoxy-1-(1-(4-methoxyphenyl)propyl)benzene

3q was synthesized following the general procedure with *E*-**1**. After purification by preparative thin-layer chromatography using PE/EA (50/1) as the eluent, **3q** was obtained in 67% yield (36.9 mg) as a colourless liquid.

^{1}H NMR (400 MHz, $CDCl_3$) δ 7.15–7.06 (m, 2H), 6.97–6.89 (m, 2H), 6.86 (d, *J* = 8.3 Hz, 1H), 6.84–6.79 (m, 2H), 3.83 (s, 3H), 3.76 (s, 3H), 3.66 (t, *J* = 7.8 Hz, 1H), 2.02–1.93 (m, 2H), 0.87 (t, *J* = 7.2 Hz, 3H); ^{13}C NMR (100 MHz, $CDCl_3$) δ 157.87, 152.25 (d, *J* = 245.0 Hz), 145.59 (d, *J* = 10.9 Hz), 138.87 (d, *J* = 5.5 Hz), 136.95, 128.59, 123.19 (d, *J* = 3.3 Hz), 115.33 (d, *J* = 18.1 Hz), 113.76, 113.22 (d, *J* = 2.2 Hz), 56.23, 55.15, 51.36 (d, *J* = 1.3 Hz), 28.69, 12.65; ^{19}F NMR (375 MHz, $CDCl_3$) δ -135.36 (dd, *J* = 12.6, 8.5 Hz); HRMS (EI) *m/z* Calcd for $C_{17}H_{19}FO_2$ $[M]^+$: 274.1364, found: 274.1363.

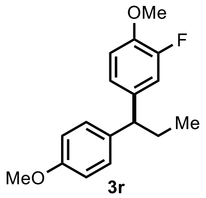

3r

2-Fluoro-1-methoxy-4-(1-(4-methoxyphenyl)propyl)benzene

3r was synthesized following the general procedure with *E*-**1**. After purification by preparative thin-layer chromatography using PE/EA (50/1) as the eluent, **3r** was obtained in 64% yield (35.0 mg) as a colourless liquid.

^{1}H NMR (400 MHz, $CDCl_3$) δ 7.15–7.07 (m, 2H), 6.96–6.89 (m, 2H), 6.86 (d, *J* = 8.4 Hz, 1H), 6.85 – 6.79 (m, 2H), 3.84 (s, 3H), 3.77 (s, 3H), 3.66 (t, *J* = 7.8 Hz, 1H), 2.02–1.93 (m, 2H), 0.87 (t, *J* = 7.2 Hz, 3H); ^{13}C NMR (100 MHz, $CDCl_3$) δ 157.89, 152.27 (d, *J* = 245.1 Hz), 145.61 (d, *J* = 10.8 Hz), 138.89 (d, *J* = 5.4 Hz), 136.97, 128.61, 123.20 (d, *J* = 3.3 Hz), 115.35 (d, *J* = 18.1 Hz), 113.78, 113.25 (d, *J* = 2.2 Hz), 56.28, 55.19, 51.38 (d, *J* = 1.3 Hz), 28.71, 12.67; ^{19}F NMR (375 MHz, $CDCl_3$) δ -135.37; HRMS (EI) *m/z* Calcd for $C_{17}H_{19}FO_2$ $[M]^+$: 274.1364, found: 274.1369.

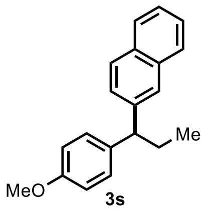

3s

2-(1-(4-Methoxyphenyl)propyl)naphthalene[11]

3s was synthesized following the general procedure with *E*-**1**. After purification by preparative thin-layer chromatography using PE/EA (20/1) as the eluent, **3s** was obtained in 90% yield (49.7 mg) as a colourless liquid.

^{1}H NMR (400 MHz, $CDCl_3$) δ 7.77 (t, *J* = 8.0 Hz, 2H), 7.72 (d, *J* = 8.4 Hz, 1H), 7.68 (s, 1H), 7.46 – 7.36 (m, 2H), 7.31 (dd, *J* = 8.4, 1.8 Hz, 1H), 7.18 (d, *J* = 8.4 Hz, 2H), 6.81 (d, *J* = 8.4 Hz, 2H), 3.90 (t, *J* = 7.6 Hz, 1H), 3.75 (s, 3H), 2.22–2.02 (m, 2H), 0.92 (t, *J* = 7.2 Hz, 3H); ^{13}C NMR (100 MHz, $CDCl_3$) δ 157.83, 142.95, 137.15, 133.51, 132.06, 128.89, 127.93, 127.66, 127.52, 126.79, 125.83, 125.74, 125.22, 113.73, 55.17, 52.35, 28.49, 12.80.

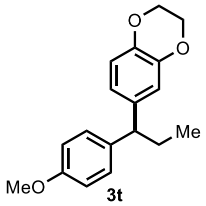

3t

6-(1-(4-Methoxyphenyl)propyl)-2,3-dihydrobenzo[*b*][1,4]dioxine

3t was synthesized following the general procedure with *E*-**1**. After purification by preparative thin-layer chromatography using PE/EA (50/1) as the eluent, **3t** was obtained in 63% yield (35.8 mg) as a colourless liquid.

^{1}H NMR (400 MHz, $CDCl_3$) δ 7.16–7.08 (m, 2H), 6.83–6.78 (m, 2H), 6.75 (d, *J* = 8.2 Hz, 1H), 6.72 (d, *J* = 2.0 Hz, 1H), 6.68 (dd, *J* = 8.0, 2.0 Hz, 1H), 4.19 (s, 4H), 3.75 (s, 3H), 3.62 (t, *J* = 7.6 Hz, 1H), 2.01–1.97 (m, 2H), 0.87 (t, *J* = 7.2 Hz, 3H); ^{13}C NMR (100 MHz, $CDCl_3$) δ 157.73, 143.16, 141.58, 139.06, 137.45, 128.60, 120.70, 116.91,

116.27, 113.68, 64.33, 64.24, 55.15, 51.63, 28.75, 12.76; HRMS (EI) *m/z* Calcd for $C_{18}H_{20}O_3$ $[M]^+$: 284.1407, found: 284.1409.

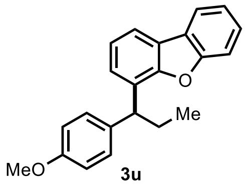

3u

4-(1-(4-Methoxyphenyl)propyl)dibenzo[*b,d*]furan

3u was synthesized following the general procedure with *E*-**1**. After purification by preparative thin-layer chromatography using PE/EA (50/1) as the eluent, **3u** was obtained in 45% yield (28.2 mg) as a colourless liquid.

^{1}H NMR (400 MHz, $CDCl_3$) δ 7.91 (dd, *J* = 7.6, 1.2 Hz, 1H), 7.77 (dd, *J* = 7.4, 1.6 Hz, 1H), 7.58 (d, *J* = 8.0 Hz, 1H), 7.43 (ddd, *J* = 8.4, 7.4, 1.2 Hz, 1H), 7.39–7.23 (m, 5H), 6.84–6.78 (m, 2H), 4.46 (t, *J* = 8.0 Hz, 1H), 3.74 (s, 3H), 2.34–2.11 (m, 2H), 0.95 (t, *J* = 7.2 Hz, 3H); ^{13}C NMR (100 MHz, $CDCl_3$) δ 157.88, 155.98, 154.34, 136.24, 129.72, 128.94, 126.85, 125.34, 124.58, 123.99, 122.86, 122.49, 120.57, 118.22, 113.68, 111.69, 55.16, 46.15, 27.94, 12.82; HRMS (EI) *m/z* Calcd for $C_{22}H_{20}O_2$ $[M]^+$: 316.1470, found: 316.1458.

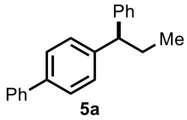

5a

4-(1-Phenylpropyl)-1,1'-biphenyl

5a was synthesized following the general procedure with **4a** (*E*/*Z* = 97/3). After purification by preparative thin-layer chromatography using PE as the eluent, **5a** was obtained in 81% yield (44.0 mg) as a colourless liquid.

^{1}H NMR (400 MHz, $CDCl_3$) δ 7.59–7.52 (m, 2H), 7.52–7.47 (m, 2H), 7.39 (t, *J* = 7.8 Hz, 2H), 7.33–7.23 (m, 6H), 7.19–7.14 (m, 1H), 3.82 (t, *J* = 7.8 Hz, 1H), 2.14–2.04 (m, 2H), 0.92 (t, *J* = 7.2 Hz, 3H); ^{13}C NMR (100 MHz, $CDCl_3$) δ 145.02, 144.28, 140.96, 138.87, 128.66, 128.40, 128.26, 127.90, 127.07, 126.99, 126.96, 126.07, 52.90, 28.58, 12.81; HRMS (EI) *m/z* Calcd for $C_{21}H_{20}$ $[M]^+$: 272.1561, found: 272.1560.

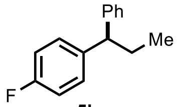

5b

1-Fluoro-4-(1-phenylpropyl)benzene [13]

5b was synthesized following the general procedure with **4b** (*E*/*Z* = 79/21). After purification by preparative thin-layer chromatography using PE as the eluent, **5b** was obtained in 74% yield (31.8 mg) as a colourless liquid.

^{1}H NMR (400 MHz, $CDCl_3$) δ 7.32–7.24 (m, 2H), 7.23–7.12 (m, 5H), 6.99–6.88 (m, 2H), 3.76 (t, *J* = 7.8 Hz, 1H), 2.09–1.97 (m, 2H), 0.88 (t, *J* = 7.2 Hz, 3H); ^{13}C NMR (100 MHz, $CDCl_3$) δ 161.23 (d, *J* = 243.9 Hz), 144.94, 140.81 (d, *J* = 3.2 Hz), 129.21 (d, *J* = 7.8 Hz), 128.40, 127.77, 126.12, 115.06 (d, *J* = 21.0 Hz), 52.40, 28.67, 12.69; ^{19}F NMR (375 MHz, $CDCl_3$) δ -117.45.

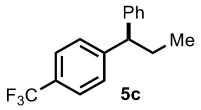

5c

1-(1-Phenylpropyl)-4-(trifluoromethyl)benzene [13]

5c was synthesized following the general procedure with **4c** (*E*/*Z* = 99/1). After purification by preparative thin-layer chromatography using PE as the eluent, **5c** was obtained in 70% yield (37.1 mg) as a colourless liquid.

^{1}H NMR (400 MHz, $CDCl_3$) δ 7.52 (d, *J* = 8.0 Hz, 2H), 7.33 (d, *J* = 8.0 Hz, 2H), 7.30–7.26 (m, 2H), 7.24–7.14 (m, 3H), 3.84 (t, *J* = 7.8 Hz, 1H), 2.14–2.08 (m, 2H), 0.90 (t, *J* = 7.2 Hz, 3H); ^{13}C NMR (100 MHz, $CDCl_3$) δ 149.27, 149.25, 144.06, 128.55, 128.20, 127.85, 126.41, 125.31 (q, *J* = 3.8 Hz), 124.30 (q, J = 270 Hz), 53.06, 28.37, 12.62; ^{19}F NMR (375 MHz, $CDCl_3$) δ -62.34.

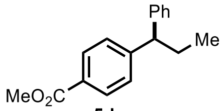

5d

Methyl-4-(1-phenylpropyl)benzoate[13]

5d was synthesized following the general procedure with **4d** (*E*/*Z* = 33/67). After purification by preparative thin-layer chromatography using PE as the eluent, **5d** was obtained in 85% yield (43.0 mg) as a colourless liquid.

^{1}H NMR (400 MHz, $CDCl_3$) δ 8.01–7.88 (m, 2H), 7.34–7.24 (m, 4H), 7.23–7.10 (m, 3H), 3.87 (s, 3H), 3.84 (t, *J* = 7.8 Hz, 1H), 2.14–2.01 (m, 2H), 0.89 (t, *J* = 7.2 Hz, 3H); ^{13}C NMR (100 MHz, $CDCl_3$) δ 167.02, 150.52, 144.16, 129.71, 128.46, 127.95, 127.91, 127.83, 126.28, 53.17, 51.93, 28.31, 12.62.

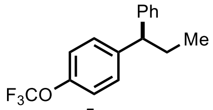

5e

1-(1-phenylpropyl)-4-(trifluoromethoxy)benzene[13]

5e was synthesized following the general procedure with **4e** (*E*/*Z* = 52/48). After purification by preparative thin-layer chromatography using PE as the eluent, **5e** was obtained in 66% yield (36.8 mg) as a colourless liquid.

^{1}H NMR (400 MHz, $CDCl_3$) δ 7.33–7.16 (m, 7H), 7.11 (d, *J* = 8.2 Hz, 2H), 3.79 (t, *J* = 7.8 Hz, 1H), 2.13–1.97 (m, 2H), 0.89 (t, *J* = 7.2 Hz, 3H); ^{13}C NMR (100 MHz, $CDCl_3$) δ 147.41, 144.47, 143.90, 129.08, 128.49, 127.83, 126.29, 120.85, 120.48 (q, *J* = 255 Hz), 52.56, 28.59, 12.67; ^{19}F NMR (375 MHz, $CDCl_3$) δ -57.86.

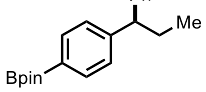

5f

4,4,5,5-Tetramethyl-2-(4-(1-phenylpropyl)phenyl)-1,3-dioxolane[13]

5f was synthesized following the general procedure with **4f** (*E*/*Z* = 62/38). After purification by preparative thin-layer chromatography using PE/Aetone (50/1) as the eluent, **5f** was obtained in 53% yield (34.1 mg) as a colourless liquid.

^{1}H NMR (400 MHz, $CDCl_3$) δ 7.73 (d, *J* = 8.0 Hz, 2H), 7.25–7.19 (m, 5H), 7.19–7.12 (m, 2H), 3.80 (t, *J* = 7.8 Hz, 1H), 2.11–2.03 (m, 2H), 1.32 (s, 12H), 0.89 (t, *J* = 7.2 Hz, 3H); ^{13}C NMR (100 MHz, $CDCl_3$) δ 148.42, 144.89, 134.91, 128.33, 127.87, 127.74, 127.41, 126.01, 83.62, 53.37, 28.36, 24.82, 12.73.

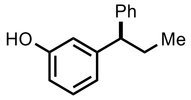

5g

3-(1-Phenylpropyl)phenol

5g was synthesized following the general procedure with **4g** (*E*/*Z* = 69/31). After purification by preparative thin-layer chromatography using PE/EA (20/1) as the eluent, **5g** was obtained in 73% yield (30.8 mg) as a colourless liquid.

^{1}H NMR (400 MHz, $CDCl_3$) δ 7.31–7.20 (m, 4H), 7.19–7.07 (m, 2H), 6.82 (d, *J* = 7.6 Hz, 1H), 6.68 (s, 1H), 6.61 (dd, *J* = 8.2, 2.6 Hz, 1H), 4.80 (s, 1H), 3.73 (t, *J* = 7.8 Hz, 1H), 2.08–1.97 (m, 2H), 0.88 (t, *J* = 7.2 Hz, 3H); ^{13}C NMR (100 MHz, $CDCl_3$) δ 155.38, 147.18, 144.83, 129.47, 128.35, 127.85, 126.07, 120.50, 114.80, 112.93, 53.01, 28.41, 12.73; HRMS (EI) *m/z* Calcd for $C_{15}H_{16}O$ $[M]^+$: 212.1193, found: 212.1196.

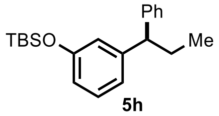

5h

***tert*-Butyldimethyl(3-(1-phenylpropyl)phenoxy)silane**

5h was synthesized following the general procedure with **4h** (*E*/*Z* = 69/31). After purification by preparative thin-layer chromatography using PE/EA (20/1) as the eluent, **5h** was obtained in 76% yield (49.8 mg) as a colourless liquid.

^{1}H NMR (400 MHz, $CDCl_3$) δ 7.29–7.18 (m, 4H), 7.18–7.04 (m, 2H), 6.81 (dt, *J* = 7.6, 1.2 Hz, 1H), 6.71 (t, *J* = 2.0 Hz, 1H), 6.64 (dt, *J* = 7.8, 2.0 Hz, 1H), 3.72 (t, *J* = 7.8 Hz, 1H), 2.08–1.98 (m, 2H), 0.95 (s, 9H), 0.89 (t, *J* = 7.2 Hz, 3H), 0.15 (s, 6H); ^{13}C NMR (100 MHz, $CDCl_3$) δ 155.57, 146.69, 145.07, 129.13, 128.28, 127.85, 125.96, 121.05, 119.71, 117.64, 53.02, 28.48, 25.71, 18.21, 12.75, -4.43; HRMS (EI) *m/z* Calcd for $C_{21}H_{30}OSi$ $[M]^+$: 326.2067, found: 326.2060.

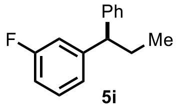

5i

1-Fluoro-3-(1-phenylpropyl)benzene

5i was synthesized following the general procedure with **4i** (*E*/*Z* = 55/45). After purification by preparative thin-layer chromatography using PE as the eluent, **5i** was obtained in 68% yield (29.0 mg) as a colourless liquid.

^{1}H NMR (400 MHz, $CDCl_3$) δ 7.28 (t, *J* = 7.6 Hz, 2H), 7.25–7.14 (m, 4H), 7.01 (d, *J* = 7.8 Hz, 1H), 6.93 (dd, *J* = 10.4, 2.4 Hz, 1H), 6.85 (td, *J* = 8.4, 2.4 Hz, 1H), 3.78 (t, *J* = 7.8 Hz, 1H), 2.10–1.99 (m, 2H), 0.89 (t, *J* = 7.2 Hz, 3H); ^{13}C NMR (100 MHz, $CDCl_3$) δ 162.92 (d, *J* = 245.1 Hz), 147.84 (d, *J* = 6.7 Hz), 144.40, 129.69 (d, *J* = 8.3 Hz), 128.46, 127.82, 126.26, 123.61 (d, *J* = 2.7 Hz), 114.66 (d, *J* = 21.2 Hz), 112.86 (d, *J* = 21.1 Hz), 52.94 (d, *J* = 1.7

Hz), 28.43, 12.66; ^{19}F NMR (375 MHz, $CDCl_3$) δ -113.52 (td, *J* = 9.7, 6.3 Hz); HRMS (EI) *m/z* Calcd for $C_{15}H_{15}F$ $[M]^+$: 214.1147, found: 214.1152.

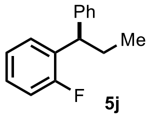

5j

1-Fluoro-2-(1-phenylpropyl)benzene

5j was synthesized following the general procedure with **4j** (*E*/*Z* = 58/42). After purification by preparative thin-layer chromatography using PE as the eluent, **5j** was obtained in 43% yield (18.3 mg) as a colourless liquid.

^{1}H NMR (400 MHz, $CDCl_3$) δ 7.31–7.22 (m, 5H), 7.21–7.11 (m, 2H), 7.07 (td, *J* = 7.4, 1.4 Hz, 1H), 6.98 (ddd, *J* = 9.8, 8.0, 1.4 Hz, 1H), 4.16 (t, *J* = 7.8 Hz, 1H), 2.13–1.98 (m, 2H), 0.91 (t, *J* = 7.2 Hz, 3H); ^{13}C NMR (100 MHz, $CDCl_3$) δ 160.79 (d, *J* = 244.8 Hz), 143.94, 132.01 (d, *J* = 14.4 Hz), 128.51 (d, *J* = 4.5 Hz), 128.33, 127.96, 127.49 (d, *J* = 8.3 Hz), 126.16, 124.02 (d, *J* = 3.6 Hz), 115.34 (d, *J* = 22.8 Hz), 45.26 (d, *J* = 2.2 Hz), 27.69, 12.61. ^{19}F NMR (375 MHz, $CDCl_3$) δ -117.94; HRMS (EI) *m/z* Calcd for $C_{15}H_{15}F$ $[M]^+$: 214.1152, found: 214.1151.

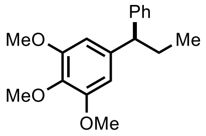

5k

1,2,3-Trimethoxy-5-(1-phenylpropyl)benzene [13]

5k was synthesized following the general procedure with **4k** (*E*/*Z* = 60/40). After purification by preparative thin-layer chromatography using PE/EA (20/1) as the eluent, **5k** was obtained in 87% yield (50.1 mg) as a colourless liquid.

^{1}H NMR (400 MHz, $CDCl_3$) δ 7.36–7.22 (m, 4H), 7.21–7.14 (m, 1H), 6.45 (s, 2H), 3.82 (s, 6H), 3.81 (s, 3H), 3.72 (t, *J* = 7.6 Hz, 1H), 2.10–1.97 (m, 2H), 0.91 (t, *J* = 7.2 Hz, 3H); ^{13}C NMR (100 MHz, $CDCl_3$) δ 152.98, 144.87, 140.82, 136.18, 128.32, 127.69, 126.07, 104.86, 60.73, 55.99, 53.46, 28.69, 12.75.

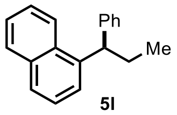

5l

1-(1-Phenylpropyl)naphthalene[14]

5l was synthesized following the general procedure with **4l** (*E*/*Z* = 90/10). After purification by preparative thin-layer chromatography using PE as the eluent, **5l** was obtained in 83% yield (40.8 mg) as a colourless liquid.

^{1}H NMR (400 MHz, $CDCl_3$) δ 8.14–8.08 (m, 1H), 7.85–7.77 (m, 1H), 7.70 (dd, *J* = 7.4, 2.0 Hz, 1H), 7.52–7.34 (m, 4H), 7.29–7.18 (m, 4H), 7.15–7.06 (m, 1H), 4.59 (t, *J* = 7.4 Hz, 1H), 2.30–2.08 (m, 2H), 0.98 (t, *J* = 7.2 Hz, 3H); ^{13}C NMR (100 MHz, $CDCl_3$) δ 144.98, 140.49, 134.03, 132.04, 128.79, 128.29, 128.10, 126.82, 125.96, 125.80, 125.38, 125.23, 124.23, 123.73, 48.15, 29.27, 12.98.

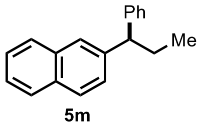

5m

2-(1-Phenylpropyl)naphthalene[11]

5m was synthesized following the general procedure with **4m** (*E*/*Z* = 98/2). After purification by preparative thin-layer chromatography using PE as the eluent, **5m** was obtained in 80% yield (39.2 mg) as a colourless liquid.

^{1}H NMR (400 MHz, $CDCl_3$) δ 7.82–7.65 (m, 4H), 7.41 (p, *J* = 7.0 Hz, 2H), 7.32 (d, *J* = 8.4 Hz, 1H), 7.28–7.22 (m, 4H), 7.16 (dt, *J* = 9.2, 5.4 Hz, 1H), 3.94 (t, *J* = 7.8 Hz, 1H), 2.23–2.08 (m, 2H), 0.93 (t, *J* = 7.2 Hz, 3H); ^{13}C NMR (100 MHz, $CDCl_3$) δ 144.99, 142.54, 133.50, 132.10, 128.36, 128.01, 127.96, 127.68, 127.53, 126.81, 126.06, 125.90, 125.86, 125.28, 53.22, 28.34, 12.80.

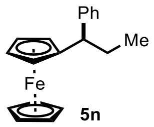

5n

(1-Phenylpropyl)ferrocene

5n was synthesized following the general procedure with **4n** (*E*/*Z* = 18/82). After purification by preparative thin-layer chromatography using PE as the eluent, **5n** was obtained in 60% yield (36.4 mg) as a yellow solid.

^{1}H NMR (400 MHz, $CDCl_3$) δ 7.26 (t, *J* = 7.4 Hz, 2H), 7.20–7.12 (m, 3H), 4.17 (s, 1H), 4.08 (s, 1H), 4.05–4.01 (m, 6H), 3.93 (s, 1H), 3.46 (dd, *J* = 10.8, 4.4 Hz, 1H), 2.17–2.04 (m, 1H), 1.87–1.73 (m, 1H), 0.83 (t, *J* = 7.4 Hz, 3H); ^{13}C NMR (100 MHz, $CDCl_3$) δ 145.37, 128.07, 127.95, 125.98, 94.45, 68.54, 67.49, 67.40, 66.93, 66.78, 47.89, 29.85, 12.71; HRMS (EI) *m/z* Calcd for $C_{19}H_{20}Fe$ $[M]^+$: 304.0909, found: 304.0913.

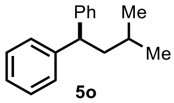

5o

(3-Methylbutane-1,1-diyl)dibenzene[15]

5o was synthesized following the general procedure with **4o** (*E*/*Z* = 28/72). After purification by preparative thin-layer chromatography using PE as the eluent, **5o** was obtained in 71% yield (32.0 mg) as a colourless liquid.

^{1}H NMR (400 MHz, $CDCl_3$) δ 7.30–7.20 (m, 8H), 7.17–7.12 (m, 2H), 4.01 (t, *J* = 8.0 Hz, 1H), 1.94–1.88 (m, 2H), 1.48–1.39 (m, 1H), 0.91 (dd, *J* = 6.6, 1.2 Hz, 6H); ^{13}C NMR (100 MHz, $CDCl_3$) δ 145.27, 128.36, 127.86, 125.97, 48.84, 45.00, 25.49, 22.62.

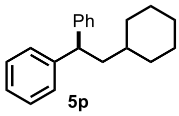

5p

(2-Cyclohexylethane-1,1-diyl)dibenzene[15]

5p was synthesized following the general procedure with **4p** (*E*/*Z* = 70/30). After purification by preparative thin-layer chromatography using PE as the eluent, **5p** was obtained in 66% yield (34.8 mg) as a colourless liquid.

^{1}H NMR (400 MHz, $CDCl_3$) δ 7.28–7.19 (m, 8H), 7.17–7.10 (m, 2H), 4.06 (t, *J* = 8.0 Hz, 1H), 1.91 (t, *J* = 7.2 Hz, 2H), 1.80–1.72 (m, 2H), 1.68–1.56 (m, 3H), 1.22–1.03 (m, 4H), 0.99–0.88 (m, 2H); ^{13}C NMR (100 MHz, $CDCl_3$) δ 145.43, 128.34, 127.87, 125.92, 47.93, 43.59, 34.83, 33.40, 26.62, 26.12.

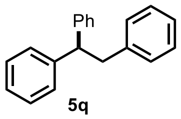

5q

Ethane-1,1,2-triyltribenzene[16]

5q was synthesized following the general procedure with *E*-**4q**. After purification by preparative thin-layer chromatography using PE as the eluent, **5q** was obtained in 89% yield (46.2 mg) as a colourless liquid.

^{1}H NMR (400 MHz, $CDCl_3$) δ 7.27–7.17 (m, 8H), 7.16–7.06 (m, 5H), 7.01–6.95 (m, 2H), 4.22 (t, *J* = 7.8 Hz, 1H), 3.35 (d, *J* = 7.8 Hz, 2H); ^{13}C NMR (100 MHz, $CDCl_3$) δ 144.42, 140.23, 129.04, 129.02, 128.31, 128.29, 128.02, 128.00, 126.15, 126.14, 125.85, 53.06, 42.07.

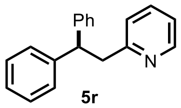

5r

2-(2,2-Diphenylethyl)pyridine[17]

5r was synthesized following the general procedure with **4r** (*E*/*Z* = 60/40). After purification by preparative thin-layer chromatography using PE/EA (10/1) as the eluent, **5r** was obtained in 72% yield (37.2 mg) as a colourless liquid.

^{1}H NMR (400 MHz, $CDCl_3$) δ 8.54–8.47 (m, 1H), 7.39 (td, *J* = 7.8, 2.0 Hz, 1H), 7.25–7.19 (m, 8H), 7.16–7.19 (m, 2H), 7.01 (ddd, *J* = 7.6, 4.8, 1.2 Hz, 1H), 6.86 (d, *J* = 7.8 Hz, 1H), 4.61 (t, *J* = 8.0 Hz, 1H), 3.52 (d, *J* = 8.0 Hz, 2H); ^{13}C NMR (100 MHz, $CDCl_3$) δ 159.85, 149.15, 144.17, 135.92, 128.29, 127.98, 126.13, 123.72, 121.06, 51.00, 44.28.

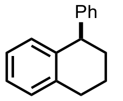

5s

1-Phenyl-1,2,3,4-tetrahydronaphthalene[18]

5s was synthesized following the general procedure with **4s**. After purification by preparative thin-layer chromatography using PE as the eluent, **5s** was obtained in 77% yield (31.9 mg) as a colourless liquid.

^{1}H NMR (400 MHz, $CDCl_3$) δ 7.27 (t, *J* = 7.4 Hz, 2H), 7.18 (t, *J* = 7.2 Hz, 1H), 7.13–7.06 (m, 4H), 7.05–6.97 (m, 1H), 6.83 (d, *J* = 7.8 Hz, 1H), 4.11 (t, *J* = 6.8 Hz, 1H), 2.95–2.79 (m, 2H), 2.19–2.12 (m, 1H), 1.97–1.81 (m, 2H), 1.80–1.67 (m, 1H); ^{13}C NMR (100 MHz, $CDCl_3$) δ 147.49, 139.35, 137.55, 130.15, 128.93, 128.82, 128.19, 125.90, 125.86, 125.61, 45.59, 33.23, 29.76, 20.93.

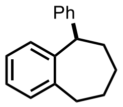

5t

5-Phenyl-6,7,8,9-tetrahydro-5*H*-benzo[7]annulene

5t was synthesized following the general procedure with **4t**. After purification by preparative thin-layer chromatography using PE as the eluent, **5t** was obtained in 49% yield (21.9 mg) as a colourless liquid.

^{1}H NMR (400 MHz, $CDCl_3$) δ 7.33 (t, *J* = 7.4 Hz, 2H), 7.26–7.21 (m, 1H), 7.18 (d, *J* = 7.6 Hz, 2H), 7.15–7.05 (m, 2H), 7.01 (td, *J* = 7.4, 1.6 Hz, 1H), 6.65 (d, *J* = 7.6 Hz, 1H), 4.27 (dd, *J* = 8.2, 2.8 Hz, 1H), 3.02–2.87 (m, 1H), 2.81–2.72 (m, 1H), 2.19–2.06 (m, 2H), 2.02–1.94 (m, 1H), 1.86–1.76 (m, 2H), 1.59–1.45 (m, 1H); ^{13}C NMR (100 MHz, $CDCl_3$) δ 145.26, 144.67, 142.85, 129.56, 128.46, 128.35, 126.05, 125.92, 49.76, 36.42, 33.88, 29.96, 27.75; HRMS (EI) *m/z* Calcd for $C_{17}H_{18}$ $[M]^+$: 222.1403, found: 222.1403.

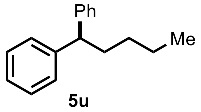

5u

Pentane-1,1-diyldibenzene[19]

5u was synthesized following the general procedure with **4u** (*E*/*Z* = 56/44). After purification by preparative thin-layer chromatography using PE as the eluent, **5u** was obtained in 80% yield (35.7 mg) as a colourless liquid.

^{1}H NMR (400 MHz, $CDCl_3$) δ 7.30–7.21 (m, 8H), 7.17–7.11 (m, 2H), 3.87 (t, *J* = 7.8 Hz, 1H), 2.03 (q, *J* = 7.8 Hz, 2H), 1.38–1.29 (m, 2H), 1.28–1.18 (m, 2H), 0.86 (t, *J* = 7.2 Hz, 3H); ^{13}C NMR (100 MHz, $CDCl_3$) δ 145.33, 128.33, 127.83, 125.95, 51.34, 35.44, 30.23, 22.69, 13.98.

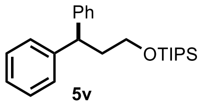

5v

(3,3-Diphenylpropoxy)triisopropylsilane

5v was synthesized following the general procedure with *E*-**4v**. After purification by preparative thin-layer chromatography using PE/EA (20/1) as the eluent, **5v** was obtained in 61% yield (45.3 mg) as a colourless liquid.

^{1}H NMR (400 MHz, $CDCl_3$) δ 7.35–7.23 (m, 8H), 7.18–7.14 (m, 2H), 4.20 (t, *J* = 7.8 Hz, 1H), 3.61 (t, *J* = 6.4 Hz, 2H), 2.31–2.24 (m, 2H), 1.08–0.97 (m, 21H); ^{13}C NMR (100 MHz, $CDCl_3$) δ 144.83, 128.35, 128.00, 126.04, 61.12, 46.87, 38.66, 18.03, 18.00, 11.95; HRMS (DAST) *m/z* Calcd for $C_{24}H_{37}OSi$ $[M+H]^+$: 369.2604, found: 369.2608.

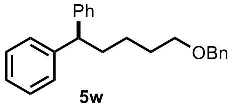

5w

(5-(Benzyloxy)pentane-1,1-diyl)dibenzene

5w was synthesized following the general procedure with **4w** (*E*/*Z* = 93/7). After purification by preparative thin-layer chromatography using PE/EA (20/1) as the eluent, **5w** was obtained in 47% yield (31.1 mg) as a colourless liquid.

^{1}H NMR (400 MHz, $CDCl_3$) δ 7.36–7.20 (m, 13H), 7.19–7.06 (m, 2H), 4.45 (s, 2H), 3.88 (t, *J* = 7.8 Hz, 1H), 3.42 (t, *J* = 6.6 Hz, 2H), 2.11–2.00 (m, 2H), 1.65 (p, *J* = 6.8 Hz, 2H), 1.40–1.26 (m, 2H); ^{13}C NMR (100 MHz, $CDCl_3$) δ 145.10, 138.56, 128.36, 128.30, 127.82, 127.60, 127.45, 126.01, 72.82, 70.19, 51.29, 35.48, 29.66, 24.63; HRMS (DAST) *m/z* Calcd for $C_{24}H_{27}O$ $[M+H]^+$: 331.2056, found: 331.2056.

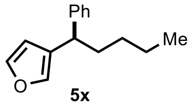

5x

3-(1-Phenylpentyl)furan

5x was synthesized following the general procedure with **4x** (*E*/*Z* = 36/64). After purification by preparative thin-layer chromatography using PE as the eluent, **5x** was obtained in 42% yield (18.1 mg) as a colourless liquid.

^{1}H NMR (400 MHz, $CDCl_3$) δ 7.39–7.15 (m, 7H), 6.22 (d, *J* = 1.8 Hz, 1H), 3.73 (t, *J* = 7.6 Hz, 1H), 2.02–1.80 (m, 2H), 1.39–1.12 (m, 4H), 0.86 (t, *J* = 7.2 Hz, 3H); ^{13}C NMR (100 MHz, $CDCl_3$) δ 144.81, 142.78, 138.76, 129.39, 128.33, 127.75, 126.16, 110.39, 42.34, 35.70, 29.96, 22.61, 13.98; HRMS (EI) *m/z* Calcd for $C_{15}H_{18}O$ $[M]^+$: 214.1352, found: 214.1353.

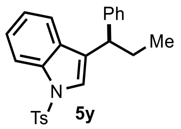

5y

3-(1-Phenylpropyl)-1-tosyl-1*H*-indole

5y was synthesized following the general procedure with **4y** (*E*/*Z* = 50/50). After purification by preparative thin-layer chromatography using PE/EA (20/1) as the eluent, **5y** was obtained in 45% yield (35.2 mg) as a colourless liquid.

^{1}H NMR (400 MHz, $CDCl_3$) δ 7.97–7.90 (m, 1H), 7.78–7.69 (m, 2H), 7.45 (d, *J* = 1.2 Hz, 1H), 7.27–7.11 (m, 9H), 7.11–7.03 (m, 1H), 3.90 (t, *J* = 7.6 Hz, 1H), 2.32 (s, 3H), 2.22–2.10 (m, 1H), 2.05–1.90 (m, 1H), 0.92 (t, *J* = 7.2 Hz, 3H); ^{13}C NMR (100 MHz, $CDCl_3$) δ 144.69, 143.30, 135.58, 135.16, 130.70, 129.73, 128.35, 127.85, 126.79, 126.68, 126.35, 124.54, 122.99, 122.67, 120.12, 113.72, 44.43, 28.50, 21.51, 12.51; HRMS (EI) *m/z* Calcd for $C_{24}H_{23}NO_2S$ $[M]^+$: 389.1444, found: 389.1454.

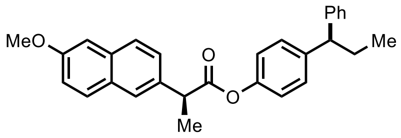

5z

4-(1-Phenylpropyl)phenyl (*S*)-2-(6-methoxynaphthalen-2-yl)propanoate

5z was synthesized following the general procedure with **4z** (*E*/*Z* = 95/5). After purification by preparative thin-layer chromatography using PE/EA (20/1) as the eluent, **5z** was obtained in 74% yield (63.1 mg) as a colourless liquid.

^{1}H NMR (400 MHz, $CDCl_3$) δ 7.76–7.67 (m, 3H), 7.47 (dd, *J* = 8.5, 1.9 Hz, 1H), 7.28–7.19 (m, 2H), 7.19–7.09 (m, 7H), 6.92–6.80 (m, 2H), 4.06 (q, *J* = 7.2 Hz, 1H), 3.89 (s, 3H), 3.74 (t, *J* = 7.8 Hz, 1H), 2.06–1.94 (m, 2H), 1.66 (d, *J* = 7.2 Hz, 3H), 0.85 (t, *J* = 7.2 Hz, 3H); ^{13}C NMR (100 MHz, $CDCl_3$) δ 173.20, 157.67, 148.93, 144.75, 142.57, 135.15, 133.74, 129.27, 128.93, 128.66, 128.32, 127.81, 127.28, 126.10, 126.07, 126.04, 121.06, 119.04, 105.53, 55.26, 52.52, 45.51, 28.55, 18.47, 12.67; HRMS (EI) *m/z* Calcd for $C_{29}H_{28}O_3$ $[M]^+$: 424.2041, found: 424.2033.

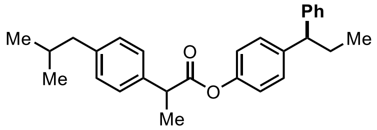

5aa

4-(1-Phenylpropyl)phenyl (2*S*)-2-(4-isobutylphenyl)propanoate

5aa was synthesized following the general procedure with **4aa** (*E*/*Z* = 92/8). After purification by preparative thin-layer chromatography using PE/EA (20/1) as the eluent, **5aa** was obtained in 52% yield (41.7 mg) as a colourless liquid.

^{1}H NMR (400 MHz, $CDCl_3$) δ 7.31–7.22 (m, 4H), 7.20–7.14 (m, 5H), 7.12 (d, *J* = 8.0 Hz, 2H), 6.93–6.86 (m, 2H), 3.90 (q, *J* = 7.2 Hz, 1H), 3.76 (t, *J* = 7.8 Hz, 1H), 2.45 (d, *J* = 7.2 Hz, 2H), 2.08–1.95 (m, 2H), 1.91–1.79 (m, 1H), 1.58 (d, *J* = 7.2 Hz, 3H), 0.93–0.83 (m, 9H); ^{13}C NMR (100 MHz, $CDCl_3$) δ 173.26, 148.97, 144.80, 142.53, 140.73, 137.24, 129.43, 128.66, 128.34, 127.83, 127.17, 126.06, 121.08, 52.55, 45.21, 45.01, 30.16, 28.57, 22.37, 18.46, 12.69; HRMS (EI) *m/z* Calcd for $C_{28}H_{32}O_2$ $[M]^+$: 400.2388, found: 400.2397.

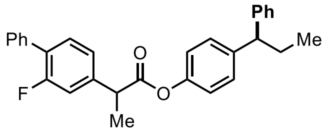

5ab

4-(1-Phenylpropyl)phenyl 2-(2-fluoro-[1,1'-biphenyl]-4-yl)propanoate

5ab was synthesized following the general procedure with **4ab** (*E*/*Z* = 95/5). After purification by preparative thin-layer chromatography using PE/EA (20/1) as the eluent, **5ab** was obtained in 41% yield (35.8 mg) as a colourless liquid.

^{1}H NMR (400 MHz, $CDCl_3$) δ 7.54 (d, *J* = 7.6 Hz, 2H), 7.46–7.34 (m, 4H), 7.27–7.17 (m, 9H), 6.94 (d, *J* = 8.0 Hz, 2H), 3.96 (q, *J* = 7.2 Hz, 1H), 3.77 (t, *J* = 7.8 Hz, 1H), 2.09–1.95 (m, 2H), 1.63 (d, *J* = 7.2 Hz, 3H), 0.87 (t, *J* = 7.2 Hz, 3H); ^{13}C NMR (100 MHz, $CDCl_3$) δ 172.50, 159.72 (d, *J* = 248.6 Hz), 148.82, 144.74, 142.79, 141.29 (d, *J* = 7.6 Hz), 135.38, 130.94 (d, *J* = 3.9 Hz), 128.94, 128.92, 128.75, 128.44, 128.36, 127.84, 127.69, 126.10, 123.56 (d, *J* = 3.4 Hz), 121.01, 115.28 (d, *J* = 23.7 Hz), 52.56, 45.10, 28.58, 18.36, 12.70; ^{19}F NMR (375 MHz, $CDCl_3$) δ -117.35; HRMS (ESI-TOF) *m/z* Calcd for $C_{30}H_{28}FO_2$ $[M+H]^+$: 439.2073, found: 439.2068.

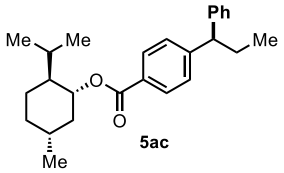

5ac

(1*R*,2*S*,5*R*)-2-isopropyl-5-methylcyclohexyl 4-(1-phenylpropyl)benzoate

5ac was synthesized following the general procedure with **4ac** (*E*/*Z* = 23/77). After purification by preparative thin-layer chromatography using PE/EA (40/1) as the eluent, **5ac** was obtained in 65% yield (49.1 mg) as a colourless liquid. The *dr* (1:1) was determined by GC.

^{1}H NMR (400 MHz, $CDCl_3$) δ 7.95 (d, *J* = 8.2 Hz, 2H), 7.33–7.25 (m, 4H), 7.24–7.15 (m, 3H), 4.90 (td, *J* = 10.8, 4.4 Hz, 1H), 3.85 (t, *J* = 7.8 Hz, 1H), 2.14–2.03 (m, 3H), 1.98–1.89 (m, 1H), 1.75–1.67 (m, 2H), 1.60–1.46 (m, 2H), 1.19–1.00 (m, 2H), 0.90 (t, *J* = 6.8 Hz, 10H), 0.77 (d, *J* = 7.0 Hz, 3H); ^{13}C NMR (100 MHz, $CDCl_3$) δ 166.02, 150.33, 150.30, 144.28, 144.25, 129.72, 128.71, 128.48, 127.86, 127.83, 126.29, 74.58, 53.22, 47.26, 40.96, 34.31, 31.41, 28.33, 28.29, 26.43, 26.41, 23.59, 23.57, 22.03, 20.76, 16.47, 16.46, 12.66; HRMS (DAST) *m/z* Calcd for $C_{26}H_{35}O_2$ $[M+H]^+$: 379.2629, found: 379.2632.

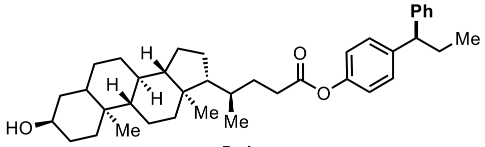

5ad

4-(1-phenylpropyl)phenyl (4*R*)-4-((3*R*, 8*R*, 9*S*, 10*S*, 13*R*, 14*S*, 17*R*)-3-hydroxy-10,13-dimethylhexadecahydro-1*H*-cyclopenta[*a*]phenanthren-17-yl)pentanoate

5ad was synthesized following the general procedure with **4ad** (*E*/*Z* = 94/6). After purification by preparative thin-layer chromatography using PE/Acetone (10/1) as the eluent, **5ad** was obtained in 69% yield (78.8 mg) as a colourless liquid.

^{1}H NMR (400 MHz, $CDCl_3$) δ 7.30–7.24 (m, 2H), 7.23–7.19 (m, 4H), 7.17 (td, *J* = 7.0, 1.6 Hz, 1H), 7.02–6.92 (m, 2H), 3.78 (t, *J* = 7.8 Hz, 1H), 3.67–3.57 (m, 1H), 2.62–2.52 (m, 1H), 2.50–2.39 (m, 1H), 2.10–2.01 (m, 2H), 1.99–1.94 (m, 1H), 1.93–1.70 (m, 5H), 1.69–1.63 (m, 1H), 1.61–1.00 (m, 20H), 0.97 (d, *J* = 6.4 Hz, 3H), 0.92 (s, 3H), 0.89 (t, *J* = 7.2 Hz, 3H), 0.65 (s, 3H); ^{13}C NMR (100 MHz, $CDCl_3$) δ 172.84, 148.87, 144.79, 142.54, 128.73, 128.37, 127.89, 126.09, 121.27, 71.86, 56.47, 55.95, 52.60, 42.74, 42.05, 40.39, 40.15, 36.42, 35.82, 35.34, 35.31, 34.55, 31.36, 30.94, 30.52, 28.62, 28.21, 27.16, 26.39, 24.19, 23.35, 20.80, 18.28, 12.73, 12.05; HRMS (ESI-TOF) *m/z* Calcd for $C_{39}H_{54}O_3Na$ [M+Na]$^+$: 593.3965, found: 593.3965.

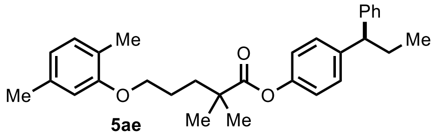

5ae

4-(1-Phenylpropyl)phenyl 5-(2,5-dimethylphenoxy)-2,2-dimethylpentanoate

5ae was synthesized following the general procedure with **4ae** (*E*/*Z* = 98/2). After purification by preparative thin-layer chromatography using PE/Acetone (50/1) as the eluent, **5ac** was obtained in 39% yield (34.6 mg) as a colourless liquid.

^{1}H NMR (400 MHz, $CDCl_3$) δ 7.31–7.23 (m, 2H), 7.23 – 7.11 (m, 5H), 6.99 (d, *J* = 7.4 Hz, 1H), 6.96–6.91 (m, 2H), 6.65 (d, *J* = 7.4 Hz, 1H), 6.61 (s, 1H), 4.00–3.92 (m, 2H), 3.78 (t, *J* = 7.8 Hz, 1H), 2.29 (s, 3H), 2.16 (s, 3H), 2.09–2.01 (m, 2H), 1.86–1.84 (m, 4H), 1.34 (s, 6H), 0.89 (t, *J* = 7.2 Hz, 3H); ^{13}C NMR (100 MHz, $CDCl_3$) δ 176.38, 156.84, 149.08, 144.84, 142.47, 136.44, 130.29, 128.74, 128.35, 127.85, 126.07, 123.58, 121.21, 120.69, 111.89, 67.75, 52.56, 42.36, 37.13, 28.60, 25.23, 25.13, 21.38, 15.78, 12.71; HRMS (ESI-TOF) *m/z* Calcd for $C_{30}H_{37}O_3$ [M+H]$^+$: 445.2745, found: 445.2737.

Other Substrates[a,b]

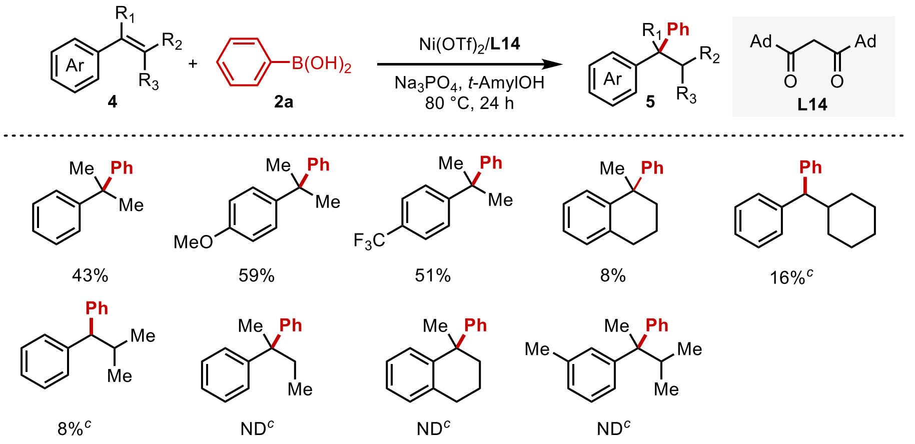

[a] Reaction conditions: **4** (0.2 mmol, 1.0 equiv), **2a** (0.6 mmol, 3.0 equiv), $Ni(OTf)_2$ (10 mol %), **L14** (10 mol%), Na_3PO_4 (0.6 mmol, 3.0 equiv), *t*-AmylOH (1.0 mL), 80 °C, N_2, 24 h. [b] Isolated yield, and the regioselectivity was determined by analysis of the crude ^1H NMR. [c] Ni(2-$NH_{2\text{-}5}$-$MeC_6H_3SO_3)_2$ instead of $Ni(OTf)_2$.

2.4 General Procedure for Ni-Catalyzed Hydroalkenylation of Internal Alkenes

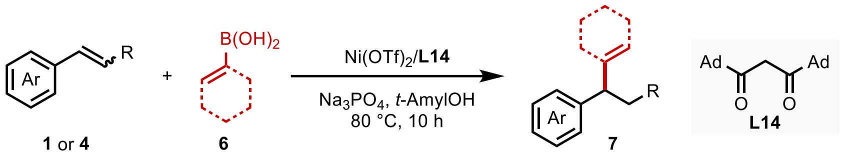

General Procedure: In a nitrogen-filled glovebox, substrate **4** (0.2 mmol, 1.0 equiv), **6** (0.6 mmol, 3.0 equiv), $Ni(OTf)_2$ (7.1 mg, 10 mol %), **L14** (6.8 mg, 10 mol %), Na_3PO_4 (98.4 mg, 0.6 mmol, 3.0 equiv) were charged in a 10-mL tube. The tube was sealed using an open-top cap with PTFE cap liner, and moved outside of the glovebox, followed by the addition of *tert*-amyl alcohol (1.0 mL). The tube was sealed again with parafilm and heated to 80 °C for 24 h. After cooling to room temperature, the mixture was passed through a pad of silica gel with EtOAc as the eluent to remove the nickel and the insoluble precipitate. The resulting solution was concentrated. The residue was then purified by silica gel chromatography or preparative thin-layer chromatography to afford the hydroarylated product **7**.

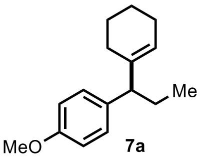

7a

1-(1-(Cyclohex-1-en-1-yl)propyl)-4-methoxybenzene

7a was synthesized following the general procedure with *E*-**1**. After purification by preparative thin-layer chromatography using *n*-hexane as the eluent, **7a** was obtained in 69% yield (31.6 mg) as a colourless liquid.

^{1}H NMR (400 MHz, $CDCl_3$) δ 7.13–7.06 (m, 2H), 6.86–6.78 (m, 2H), 5.60–5.56 (m, 1H), 3.78 (s, 3H), 2.93 (t, J = 7.6 Hz, 1H), 2.04 (q, J = 3.4 Hz, 2H), 1.87–1.55 (m, 4H), 1.51 (p, J = 3.2 Hz, 4H), 0.82 (t, J = 7.2 Hz, 3H); ^{13}C NMR (100 MHz, $CDCl_3$) δ 157.66, 140.31, 136.67, 128.72, 120.97, 113.32, 55.16, 54.00, 26.45, 25.59, 25.34, 23.03, 22.68, 12.58; HRMS (EI) *m/z* Calcd for $C_{16}H_{22}O$ $[M]^+$: 230.1671, found: 230.1663.

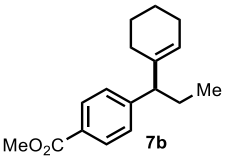

7b

Methyl-4-(1-(cyclohex-1-en-1-yl)propyl)benzoate

7b was synthesized following the general procedure with **4d** (*E*/*Z* = 33/67). After purification by preparative thin-layer chromatography using PE/EA (40/1) as the eluent, **7b** was obtained in 60% yield (30.9 mg) as a colourless liquid.

^{1}H NMR (400 MHz, $CDCl_3$) δ 7.98–7.91 (m, 2H), 7.29–7.22 (m, 2H), 5.65–5.61 (m, 1H), 3.90 (s, 3H), 3.04 (t, J = 7.6 Hz, 1H), 2.07–2.02 (m, 2H), 1.93–1.79 (m, 1H), 1.74–1.60 (m, 3H), 1.51 (p, J = 3.0 Hz, 4H), 0.83 (t, J = 7.2 Hz, 3H); ^{13}C NMR (100 MHz, $CDCl_3$) δ 167.21, 150.24, 139.30, 129.38, 127.93, 127.79, 122.00, 54.90, 51.93, 26.59, 25.32, 25.27, 22.93, 22.53, 12.45; HRMS (EI) *m/z* Calcd for $C_{17}H_{22}O_2$ $[M]^+$: 258.1620, found: 258.1615.

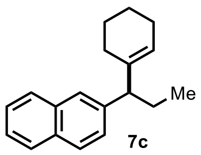

7c

2-(1-(Cyclohex-1-en-1-yl)propyl)naphthalene

7c was synthesized following the general procedure with **4m** (*E*/*Z* = 98/2). After purification by preparative thin-layer chromatography using PE as the eluent, **7c** was obtained in 75% yield (37.6 mg) as a colourless liquid.

^{1}H NMR (400 MHz, $CDCl_3$) δ 7.79 (dd, *J* = 7.6, 1.8 Hz, 2H), 7.74 (d, *J* = 8.4 Hz, 1H), 7.61 (d, *J* = 1.6 Hz, 1H), 7.48–7.37 (m, 2H), 7.34 (dd, *J* = 8.4, 1.8 Hz, 1H), 5.70–5.66 (m, 1H), 3.14 (t, *J* = 7.6 Hz, 1H), 2.07 (p, *J* = 3.6 Hz, 2H), 2.00–1.84 (m, 1H), 1.87–1.70 (m, 3H), 1.54–1.46 (m, 4H), 0.86 (t, *J* = 7.2 Hz, 3H); ^{13}C NMR (100 MHz, $CDCl_3$) δ 142.04, 139.94, 133.41, 132.14, 127.59, 127.51, 126.75, 126.07, 125.67, 125.03, 121.51, 54.95, 26.64, 25.39, 25.30, 23.01, 22.65, 12.61; HRMS (EI) *m/z* Calcd for C_{19} $H_{22}NO_2S$ $[M]^+$: 250.1722, found: 250.1718.

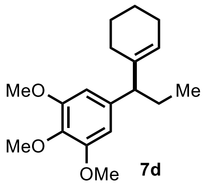

7d

5-(1-(Cyclohex-1-en-1-yl)propyl)-1,2,3-trimethoxybenzene

7d was synthesized following the general procedure with **4k** (*E*/*Z* = 60/40). After purification by preparative thin-layer chromatography using PE/EA (40/1) as the eluent, **7d** was obtained in 55% yield (32.0 mg) as a colourless liquid.

^{1}H NMR (400 MHz, $CDCl_3$) δ 6.40 (s, 2H), 5.60 (q, *J* = 3.4 Hz, 1H), 3.84 (s, 6H), 3.83 (s, 3H), 2.91 (t, *J* = 7.6 Hz, 1H), 2.06 (s, 2H), 1.89–1.38 (m, 8H), 0.85 (t, *J* = 7.3 Hz, 3H); ^{13}C NMR (100 MHz, $CDCl_3$) δ 152.76, 140.46, 139.83, 136.03, 121.41, 104.83, 60.82, 56.04, 55.12, 26.58, 25.62, 25.37, 23.07, 22.65, 12.62; HRMS (EI) *m/z* Calcd for $C_{18}H_{26}O_3$ $[M]^+$: 290.1882, found: 290.1882.

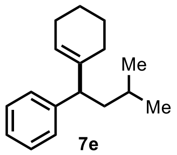

7e

(1-(Cyclohex-1-en-1-yl)-3-methylbutyl)benzene

7e was synthesized following the general procedure with **4o** (*E*/*Z* = 28/72). After purification by preparative thin-layer chromatography using PE as the eluent, **7e** was obtained in 60% yield (27.6 mg) as a colourless liquid.

^{1}H NMR (400 MHz, $CDCl_3$) δ 7.30–7.22 (m, 2H), 7.22–7.12 (m, 3H), 5.61 (d, *J* = 4.0 Hz, 1H), 3.22 (t, *J* = 8.0 Hz, 1H), 2.09–1.93 (m, 2H), 1.86–1.70 (m, 2H), 1.70–1.48 (m, 6H), 1.47–1.37 (m, 1H), 0.90 (d, *J* = 6.6 Hz, 3H), 0.86 (d, *J* = 6.6 Hz, 3H); ^{13}C NMR (100 MHz, $CDCl_3$) δ 144.75, 140.27, 127.99, 127.88, 125.74, 121.14, 50.33, 41.86, 26.58, 25.37, 25.32, 23.04, 22.92, 22.63, 22.61; HRMS (EI) *m/z* Calcd for $C_{17}H_{24}$ $[M]^+$: 228.1878, found: 228.1871.

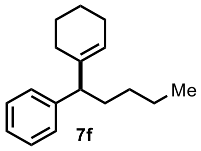

7f

(1-(Cyclohex-1-en-1-yl)pentyl)benzene

7f was synthesized following the general procedure with **4u** (*E*/*Z* = 56/44). After purification by preparative thin-layer chromatography using *n*-hexane as the eluent, **7f** was obtained in 85% yield (39.0 mg) as a colourless liquid.

^{1}H NMR (400 MHz, $CDCl_3$) δ 7.30–7.22 (m, 2H), 7.21–7.12 (m, 3H), 5.61 (t, *J* = 4.0 Hz, 1H), 3.07 (t, *J* = 7.6 Hz, 1H), 2.06–2.01 (m, 2H), 1.87–1.60 (m, 4H), 1.55–1.46 (m, 4H), 1.37–1.06 (m, 3H), 0.86 (t, *J* = 7.2 Hz, 3H); ^{13}C NMR (100 MHz, $CDCl_3$) δ 144.81, 140.27, 127.98, 127.87, 125.74, 121.10, 52.96, 32.37, 30.13, 26.58, 25.35, 23.03, 22.80, 22.64, 14.07; HRMS (EI) *m/z* Calcd for $C_{17}H_{24}$ $[M]^+$: 228.1878, found: 228.1873.

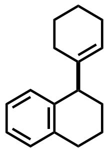
7g

1-(Cyclohex-1-en-1-yl)-1,2,3,4-tetrahydronaphthalene

7g was synthesized following the general procedure with **4s**. After purification by preparative thin-layer chromatography using *n*-hexane as the eluent, **7g** was obtained in 77% yield (32.7 mg) as a colourless liquid.

^{1}H NMR (400 MHz, $CDCl_3$) δ 7.14–7.01 (m, 4H), 5.39 (tt, *J* = 3.6, 1.6 Hz, 1H), 3.44–3.36 (m, 1H), 2.90–2.53 (m, 2H), 2.19–1.97 (m, 2H), 1.96–1.68 (m, 5H), 1.65–1.49 (m, 5H); ^{13}C NMR (100 MHz, $CDCl_3$) δ 141.13, 138.85, 137.65, 128.92, 128.85, 125.50, 125.41, 124.44, 47.79, 29.88, 28.52, 25.66, 25.44, 23.14, 22.70, 21.61; HRMS (EI) *m/z* Calcd for $C_{16}H_{20}$ $[M]^+$: 212.1565, found: 212.1560.

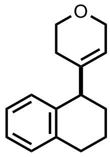

7h

4-(1,2,3,4-Tetrahydronaphthalen-1-yl)-3,6-dihydro-2*H*-pyran

7h was synthesized following the general procedure with **4s**. After purification by preparative thin-layer chromatography using PE as the eluent, **7h** was obtained in 44% yield (19.0 mg) as a colourless liquid.

^{1}H NMR (400 MHz, $CDCl_3$) δ 7.15–7.04 (m, 4H), 5.35 (t, *J* = 2.4 Hz, 1H), 4.23– 4.10 (m, 2H), 3.81 (dt, *J* = 10.6, 5.2 Hz, 1H), 3.72 (ddd, *J* = 11.2, 6.6, 4.6 Hz, 1H), 3.51–3.43 (m, 1H), 2.85–2.68 (m, 2H), 2.10–2.00 (m, 1H), 1.99–1.83 (m, 3H), 1.82–1.65 (m, 2H); ^{13}C NMR (101 MHz, $CDCl_3$) δ 139.14, 137.71, 137.60, 129.10, 129.03, 125.87, 125.53, 122.99, 65.63, 64.50, 46.73, 29.68, 28.02, 26.04, 21.12; HRMS (EI) *m/z* Calcd for $C_{15}H_{18}O$ $[M]^+$: 214.1358, found: 214.1351.

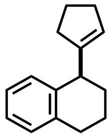

7i

1-(Cyclopent-1-en-1-yl)-1,2,3,4-tetrahydronaphthalene

7i was synthesized following the general procedure with **4s**. After purification by preparative thin-layer chromatography using PE as the eluent, **7i** was obtained in 60% yield (25.6 mg) as a colourless liquid.

^{1}H NMR (400 MHz, $CDCl_3$) δ 7.10–7.04 (m, 4H), 5.25 (t, *J* = 2.4 Hz, 1H), 3.65 (t, *J* = 5.8 Hz, 1H), 2.82–2.70 (m, 2H), 2.40–2.27 (m, 2H), 2.22–2.12 (m, 2H), 1.94–1.77 (m, 5H), 1.80–1.67 (m, 1H); ^{13}C NMR (100 MHz, $CDCl_3$) δ 148.29, 138.67, 137.09, 129.25, 128.91, 126.88, 125.61, 125.30, 41.22, 32.45, 32.23, 29.64, 28.21, 23.73, 20.91.; HRMS (EI) *m/z* Calcd for $C_{15}H_{18}$ $[M]^+$: 198.1409, found: 198.1402.

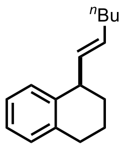

7j

(*E*)-1-(Hex-1-en-1-yl)-1,2,3,4-tetrahydronaphthalene

7j was synthesized following the general procedure with **4s**. After purification by preparative thin-layer chromatography using PE as the eluent, **7j** was obtained in 15% yield (6.3 mg) as a colourless liquid.

^{1}H NMR (400 MHz, $CDCl_3$) δ 7.19–7.14 (m, 1H), 7.13–7.02 (m, 3H), 5.57–5.36 (m, 2H), 3.38 (q, *J* = 6.4 Hz, 1H), 2.84–2.72 (m, 2H), 2.10–1.98 (m, 2H), 1.99–1.82 (m, 2H), 1.80–1.58 (m, 2H), 1.48–1.22 (m, 4H), 0.90 (t, *J* = 6.8 Hz, 3H); ^{13}C NMR (100 MHz, $CDCl_3$) δ 139.21, 136.93, 134.66, 131.24, 129.42, 129.00, 125.69, 125.40, 42.52, 32.19, 31.74, 30.58, 29.72, 22.22, 20.92, 13.97; HRMS (EI) *m/z* Calcd for $C_{16}H_{22}$ $[M]^+$: 214.1722, found: 214.1712.

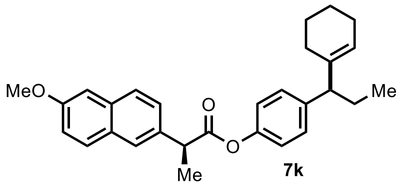

7k

4-(1-(Cyclohex-1-en-1-yl)propyl)phenyl (2*S*)-2-(6-methoxynaphthalen-2-yl)propanoate

7k was synthesized following the general procedure with **4z** (*E*/*Z* = 95/5). After purification by preparative thin-layer chromatography using PE/EA (20/1) as the eluent, **7k** was obtained in 40% yield (34.5 mg) as a colourless liquid.

^{1}H NMR (400 MHz, $CDCl_3$) δ 7.85–7.62 (m, 3H), 7.50 (dd, *J* = 8.4, 2.0 Hz, 1H), 7.20–7.02 (m, 4H), 6.96–6.76 (m, 2H), 5.71–5.37 (m, 1H), 4.08 (q, *J* = 7.2 Hz, 1H), 3.91 (s, 3H), 2.94 (t, *J* = 7.6 Hz, 1H), 2.04–1.96 (m, 2H), 1.87–1.58 (m, 7H), 1.52–1.44 (m, 4H), 0.79 (t, *J* = 7.2 Hz, 3H); ^{13}C NMR (100 MHz, $CDCl_3$) δ 173.29, 157.67, 148.84, 142.00, 139.76, 135.21, 133.75, 129.29, 128.95, 128.60, 127.28, 126.14, 126.09, 121.44, 120.68, 119.03, 105.54, 55.27, 54.22, 45.54, 26.39, 25.43, 25.30, 22.93, 22.57, 18.48, 12.48; HRMS (EI) *m/z* Calcd for $C_{29}H_{32}O_3$ $[M]^+$: 428.2351, found: 428.2364.

3. Mechanistic Study

3.1 Kinetics Data

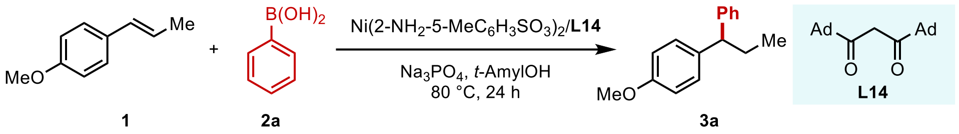

(S1)

General Kinetics Experimental Procedure: In a nitrogen-filled glovebox, substrate **1**, **2a**, Ni(2-NH_2-5-$MeC_6H_3SO_3)_2$, **L14**, Na_3PO_4 were charged in a 10-mL tube, and added 5.0 µL dodecane as internal standard. The tube was sealed using an open-top cap with PTFE cap liner, and moved outside of the glovebox, followed by the addition of *tert*-amyl alcohol (1.0 mL). The tube was sealed again with parafilm and heated to 80 °C. The reaction progress was monitored by removing aliquots (~10 µL) from the reaction mixture via syringe under N_2. Each aliquot was quenched by EA (4.0 mL) in an 8.0 mL tube. The mixture was filtered with a filter head into 2.0 mL GC vial and analyzed by gas chromatography.

3.1.1 Kinetic Plots for "Different Excess" Experiment

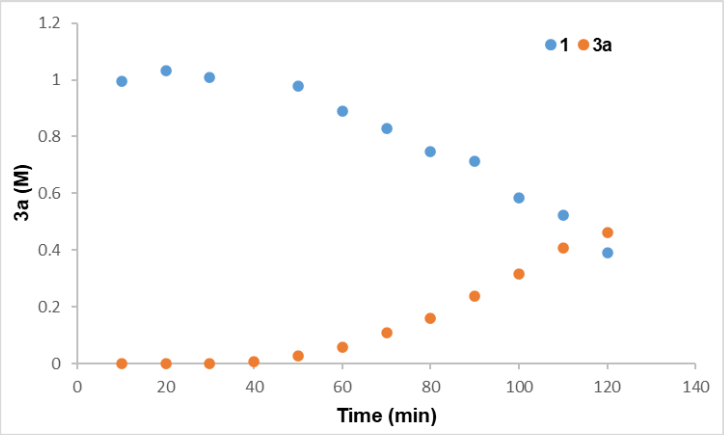

Supplementary Figure 2. Time-course for the reaction shown in eq **S1**. Reaction conditions: [Ni(2-NH_2-5-$MeC_6H_3SO_3)_2$] = 0.02 M, [**L14**] = 0.02 M, Na_3PO_4 = 0.6 mmol, solvent = *t*-AmylOH. [**1**]$_0$ = 0.20 M; [**2a**]$_0$ = 0.60 M.

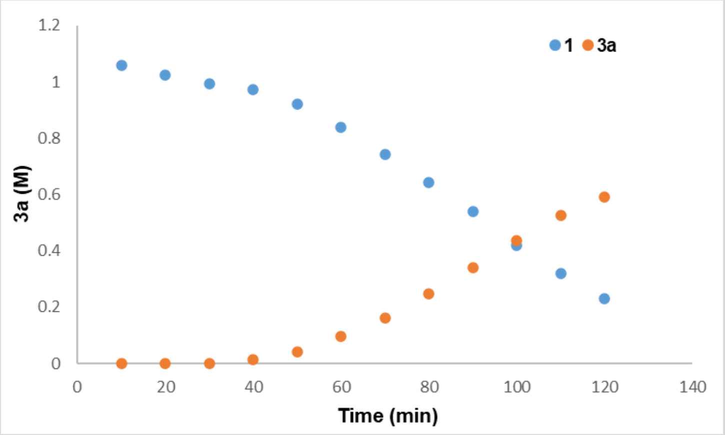

Supplementary Figure 3. Time-course for the reaction shown in eq **S1**. Reaction conditions: [Ni(2-NH_2-5-$MeC_6H_3SO_3)_2$] = 0.02 M, [**L14**] = 0.02 M, Na_3PO_4 = 0.6 mmol, solvent = *t*-AmylOH. [**1**]$_0$ = 0.20 M; [**2a**]$_0$ = 0.70 M.

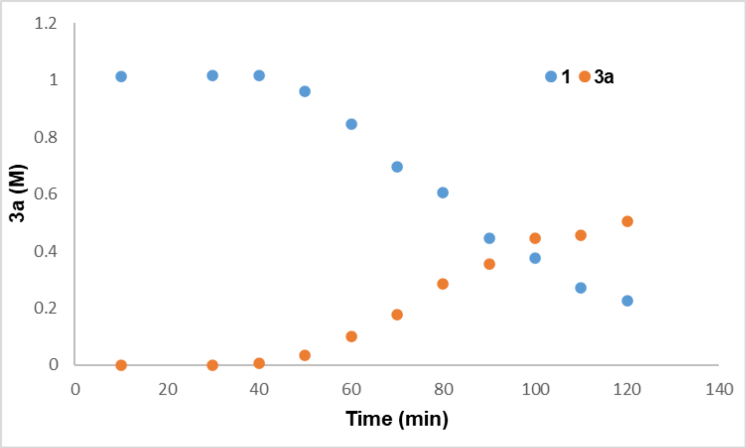

Supplementary Figure 4. Time-course for the reaction shown in eq **S1**. Reaction conditions: [Ni(2-NH_2-5-$MeC_6H_3SO_3)_2$] = 0.02 M, [**L14**] = 0.02 M, Na_3PO_4 = 0.6 mmol, solvent = *t*-AmylOH. [**1**]$_0$ = 0.10 M; [**2a**]$_0$ = 0.60 M.

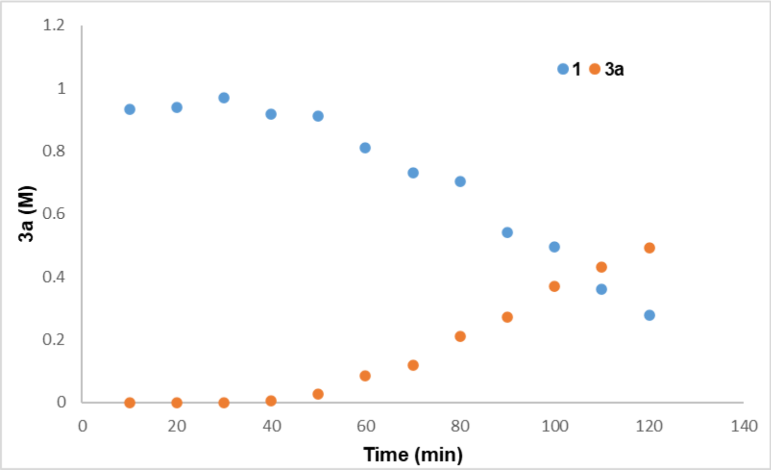

Supplementary Figure 5. Time-course for the reaction shown in eq **S1**. Reaction conditions: [Ni(2-NH_2-5-$MeC_6H_3SO_3)_2$] = 0.02 M, [**L14**] = 0.02 M, Na_3PO_4 = 0.6 mmol, solvent = *t*-AmylOH. [**1**]$_0$ = 0.15 M; [**2a**]$_0$ = 0.60 M.

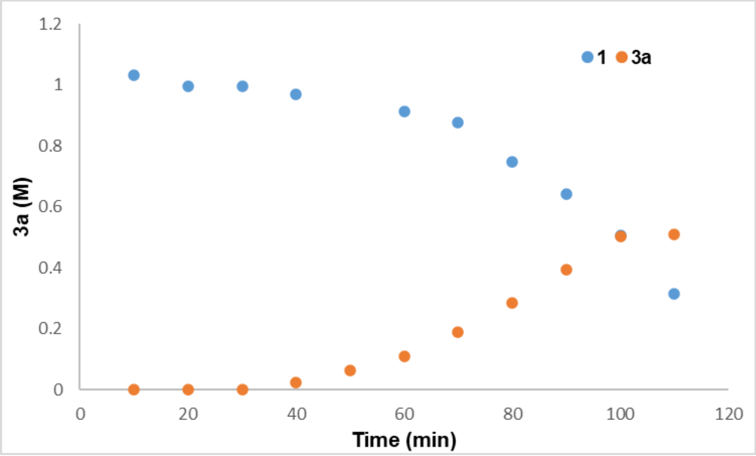

Supplementary Figure 6. Time-course for the reaction shown in eq **S1**. Reaction conditions: [Ni(2-NH_2-5-$MeC_6H_3SO_3)_2$] = 0.02 M, [**L14**] = 0.02 M, Na_3PO_4 = 0.6 mmol, solvent = *t*-AmylOH. [**1**]$_0$ = 0.20 M; [**2a**]$_0$ = 0.65 M.

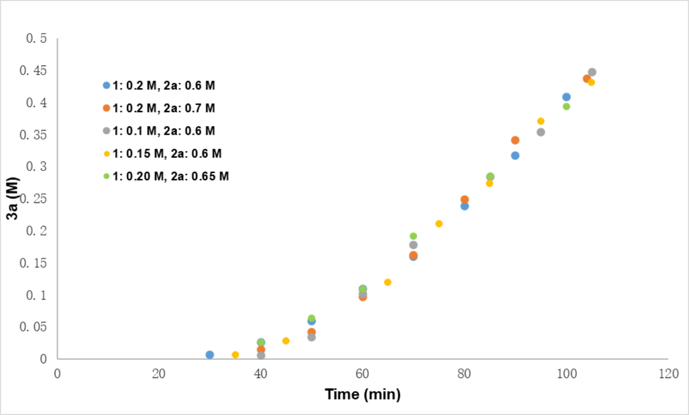

Supplementary Figure 7. "Different excess" experiments reveal that this reaction is zero-order dependence on alkene concentration and arylboronic acid concentration.

3.1.2 Kinetic Plots with Different [Ni] loading

The study of kinetic orders in the catalyst was completed following the general procedure described above.

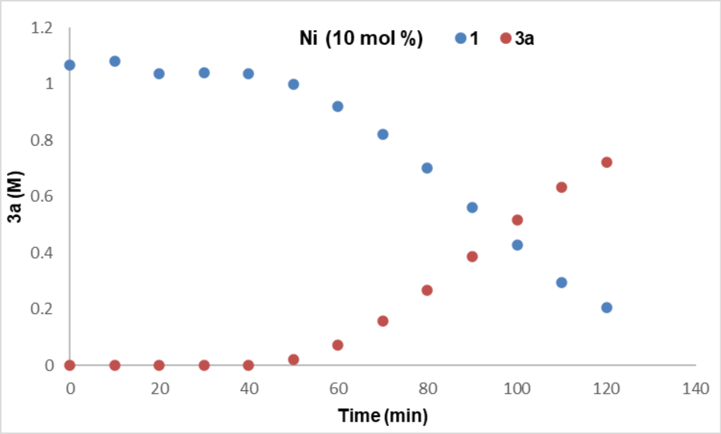

Supplementary Figure 8. Time-course for the reaction shown in eq **S1**. Reaction conditions: [Ni(2-NH_2-5-$MeC_6H_3SO_3)_2$] = 0.02 M, [**L14**] = 0.02 M, Na_3PO_4 = 0.6 mmol, solvent = *t*-AmylOH. [**1**]$_0$ = 0.20 M; [**2a**]$_0$ = 0.60 M.

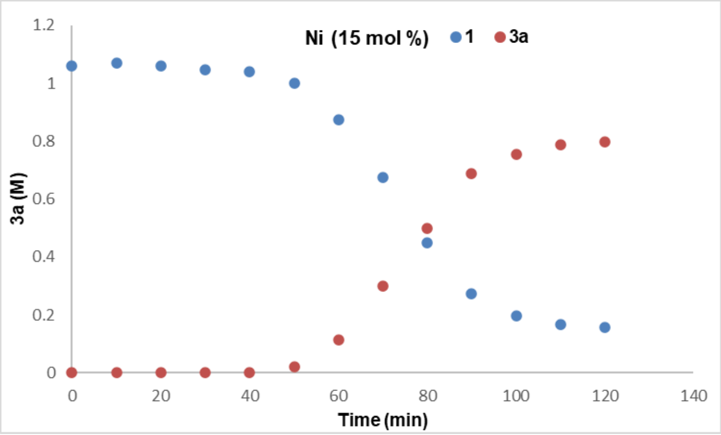

Supplementary Figure 9. Time-course for the reaction shown in eq **S1**. Reaction conditions: [Ni(2-NH_2-5-$MeC_6H_3SO_3)_2$] = 0.03 M, [**L14**] = 0.03 M, Na_3PO_4 = 0.6 mmol, solvent = *t*-AmylOH. [**1**]$_0$ = 0.20 M; [**2a**]$_0$ = 0.60 M.

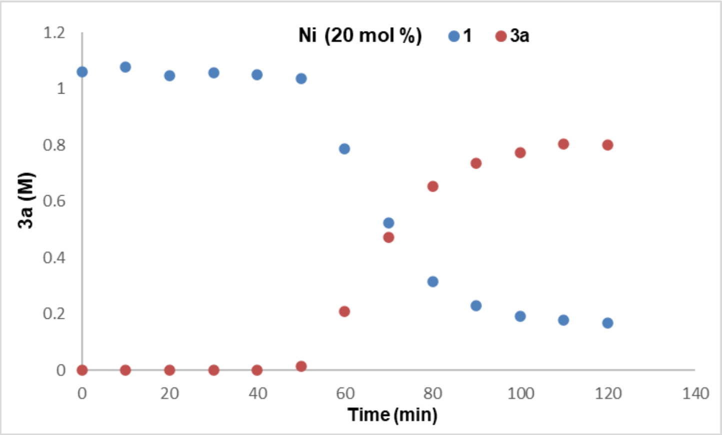

Supplementary Figure 10. Time-course for the reaction shown in eq **S1**. Reaction conditions: [Ni(2-NH_2-5-$MeC_6H_3SO_3$)$_2$] = 0.04 M, [**L14**] = 0.04 M, Na_3PO_4 = 0.6 mmol, solvent = *t*-AmylOH. [**1**]$_0$ = 0.20 M; [**2a**]$_0$ = 0.60 M.

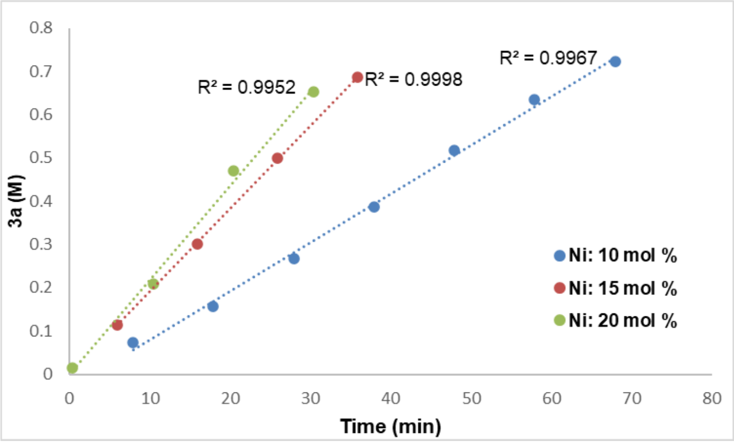

Supplementary Figure 11. Various normalization analysis of product vs.Time.

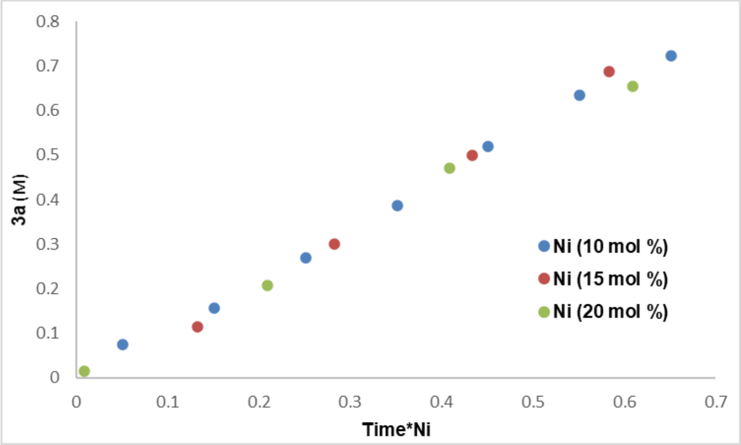

Supplementary Figure 12. Various normalization analysis of product vs.[catalyst.]*(time).

The experiments with different concentration of [Ni] were performed to evaluate the order of [Ni] catalyst. When normalizing the rates as a function of [Ni] catalyst, good overlay of the curves indicates the first order with regard to [Ni] catalyst (Supplementary Figure 12).

3.2 Deuteration Experiments

3.2.1 Deuterium-Scrambling Reactions

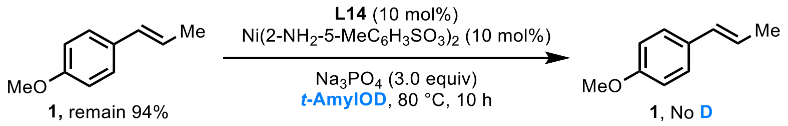

Following the general procedure, in a nitrogen-filled glovebox, substrate **1** (0.1 mmol, 1.0 equiv), Ni(2-NH_2-5-$MeC_6H_3SO_3)_2$ (4.3 mg, 10 mol %), **L14** (3.4 mg, 10 mol %), Na_3PO_4 (49.2 mg, 3.0 equiv) were charged in a 10-mL tube. The tube was sealed using an open-top cap with PTFE cap liner, and moved outside of the glovebox, followed by the addition of *tert*-amyl alcohol (1.0 mL). The tube was sealed again with parafilm and heated to 80 ºC for 10 h. After cooling to room temperature, the mixture was passed through a pad of silica gel with DCM as the eluent to remove the nickel and the insoluble precipitate. The resulting solution was concentrated. The residue was then purified by preparative thin-layer chromatography to give **1** as a colourless liquid in 94% yield.

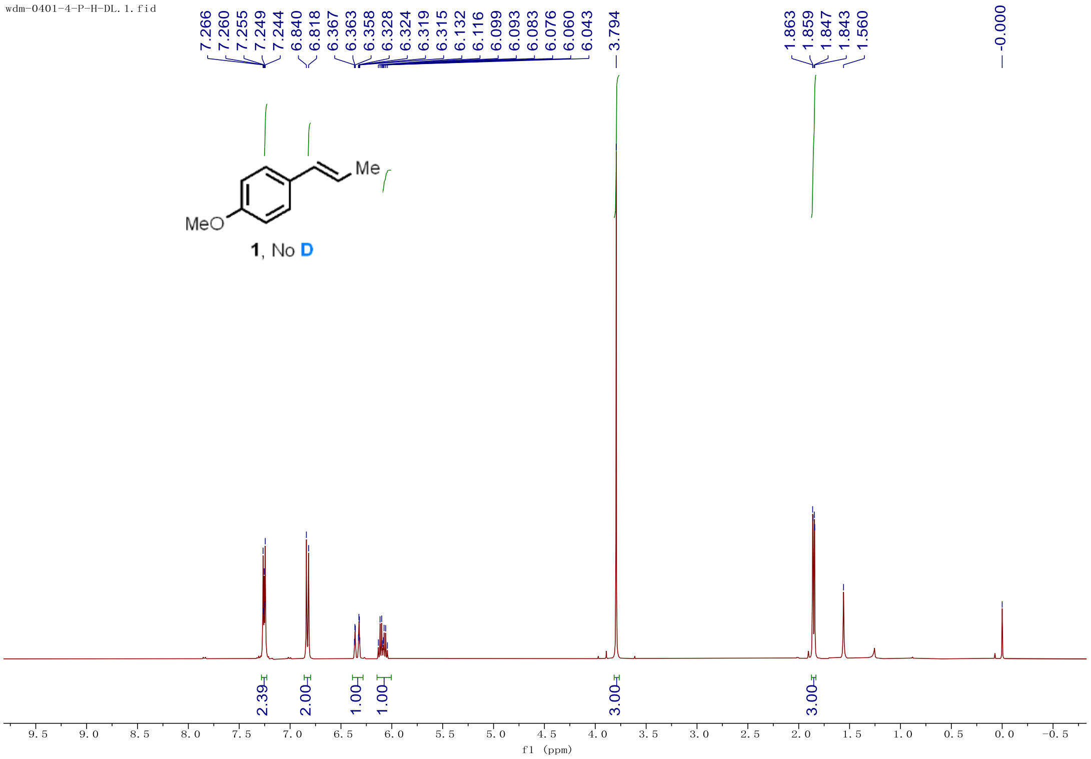

Supplementary Figure 13. ^{1}H NMR (400 MHz, $CDCl_3$) spectrum of 1

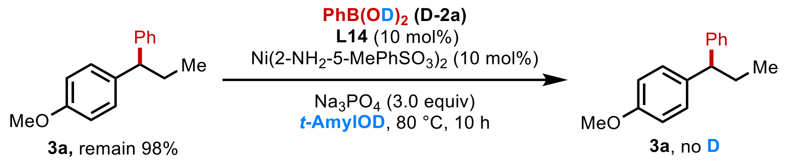

Following the general procedure, in a nitrogen-filled glovebox, substrate **3a** (0.1 mmol, 1.0 equiv), **D-2a** (0.3 mmol, 3.0 equiv), Ni(2-NH_2-5-$MeC_6H_3SO_3)_2$ (4.3 mg, 10 mol %), **L14** (3.4 mg, 10 mol %), Na_3PO_4 (49.2 mg, 3.0 equiv) were charged in a 10-mL tube. The tube was sealed using an open-top cap with PTFE cap liner, and moved outside

of the glovebox, followed by the addition of *tert*-amyl alcohol (0.5 mL). The tube was sealed again with parafilm and heated to 80 °C for 10 h. After cooling to room temperature, the mixture was passed through a pad of silica gel with DCM as the eluent to remove the nickel and the insoluble precipitate. The resulting solution was concentrated. The residue was then purified by preparative thin-layer chromatography as a colourless liquid in 98% yield.

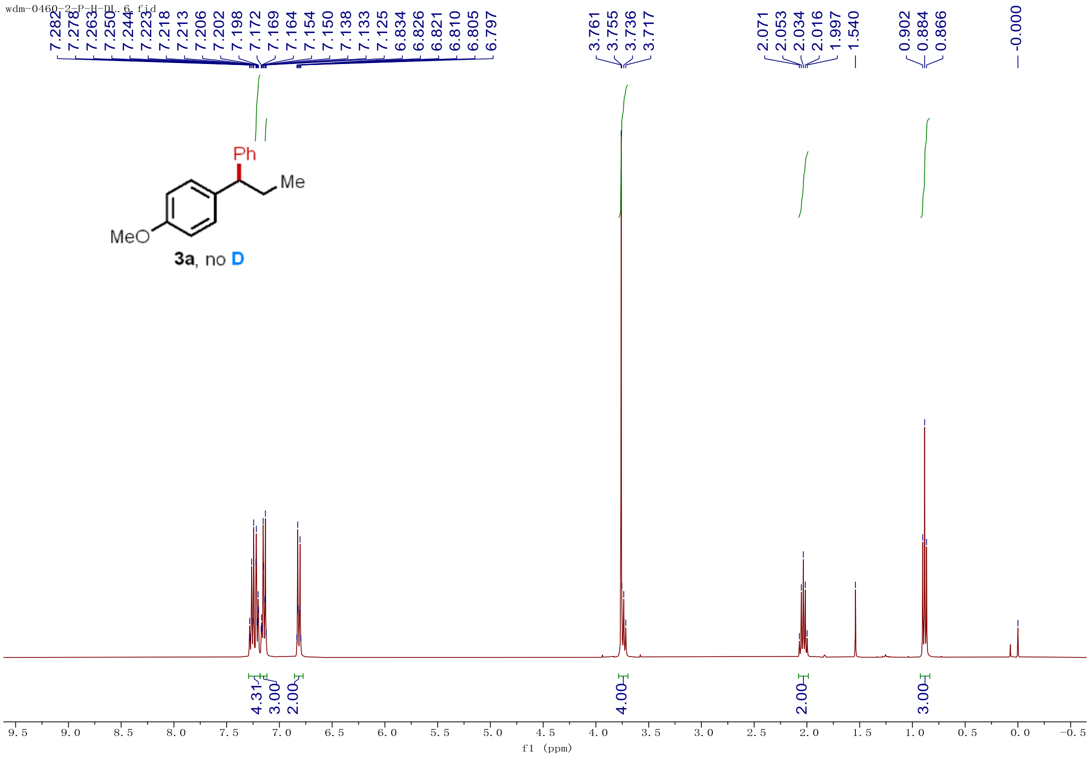

Supplementary Figure 14. ^{1}H NMR (400 MHz, $CDCl_3$) spectrum of 3a

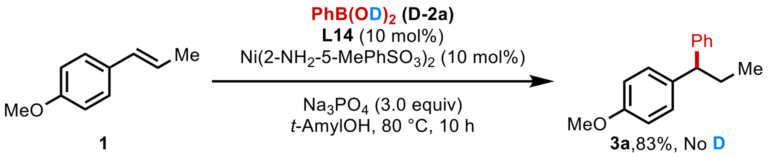

Following the general procedure, in a nitrogen-filled glovebox, substrate **1** (0.1 mmol, 1.0 equiv), **D-2a** (0.3 mmol, 3.0 equiv), Ni(2-NH_2-5-$MeC_6H_3SO_3$)$_2$ (4.3 mg, 10 mol %), **L14** (3.4 mg, 10 mol %), Na_3PO_4 (49.2 mg, 3.0 equiv) were charged in a 10-mL tube. The tube was sealed using an open-top cap with PTFE cap liner, and moved outside of the glovebox, followed by the addition of *tert*-amyl alcohol (0.5 mL). The tube was sealed again with parafilm and heated to 80 °C for 10 h. After cooling to room temperature, the mixture was passed through a pad of silica gel with DCM as the eluent to remove the nickel and the insoluble precipitate. The resulting solution was concentrated. The residue was then purified by preparative thin-layer chromatography as a colourless liquid in 83% yield.

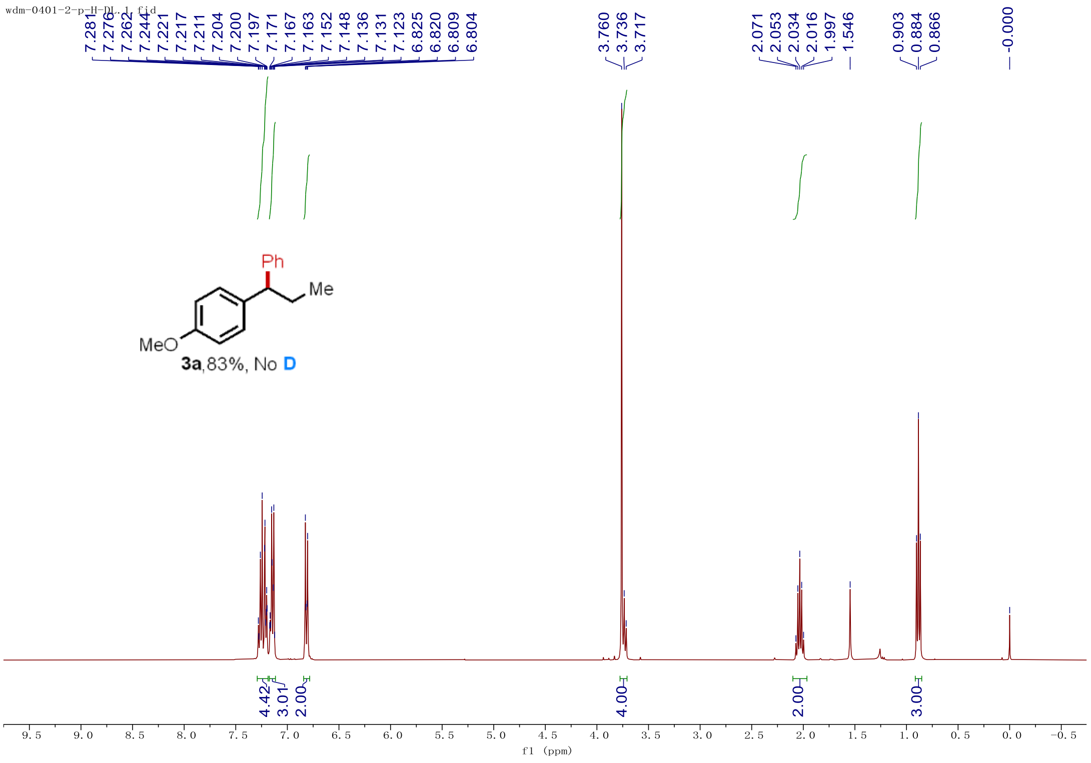

Supplementary Figure 15. ^{1}H NMR (400 MHz, $CDCl_3$) spectrum of 3a

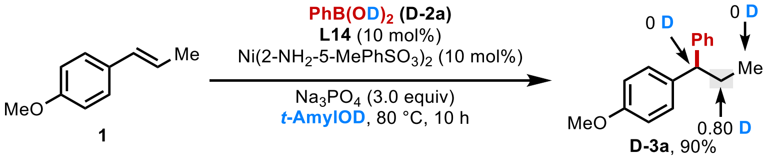

Following the general procedure, in a nitrogen-filled glovebox, substrate **1** (0.1 mmol, 1.0 equiv), **D-2a** (0.3 mmol, 3.0 equiv), Ni(2-NH_2-5-$MeC_6H_3SO_3$)$_2$ (4.3 mg, 10 mol %), **L14** (3.4 mg, 10 mol %), Na_3PO_4 (49.2 mg, 3.0 equiv) were charged in a 10-mL tube. The tube was sealed using an open-top cap with PTFE cap liner, and moved outside of the glovebox, followed by the addition of *tert*-amyl alcohol (0.5 mL). The tube was sealed again with parafilm and heated to 80 ^{o}C for 10 h. After cooling to room temperature, the mixture was passed through a pad of silica gel with DCM as the eluent to remove the nickel and the insoluble precipitate. The resulting solution was concentrated. The residue was then purified by preparative thin-layer chromatography as a colourless liquid in 83% yield.

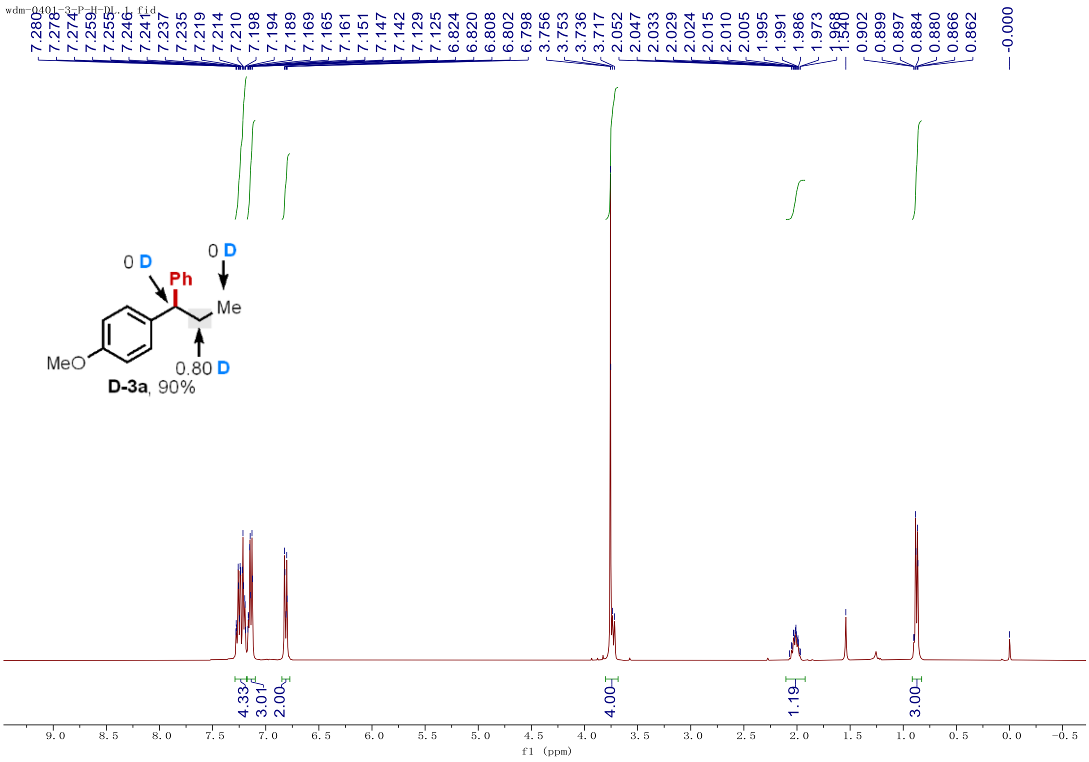

Supplementary Figure 16. ^{1}H NMR (400 MHz, $CDCl_3$) spectrum of D-3a

3.2.2 KIE Study

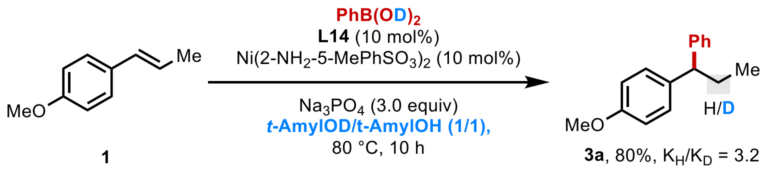

Following the general procedure, in a nitrogen-filled glovebox, substrate **1** (0.1 mmol, 1.0 equiv), **2a** (0.3 mmol, 3.0 equiv), Ni(2-NH_2-5-Me$C_6H_3SO_3$)$_2$ (4.3 mg, 10 mol %), **L14** (3.4 mg, 10 mol %), Na_3PO_4 (49.2 mg, 3.0 equiv) were charged in a 10-mL tube. The tube was sealed using an open-top cap with PTFE cap liner, and moved outside of the glovebox, followed by the addition of *tert*-amyl alcohol (0.5 mL). The tube was sealed again with parafilm and heated to 80 °C for 10 h. After cooling to room temperature, the mixture was passed through a pad of silica gel with DCM as the eluent to remove the nickel and the insoluble precipitate. The resulting solution was concentrated. The residue was then purified by preparative thin-layer chromatography as a colourless liquid in 80% yield.

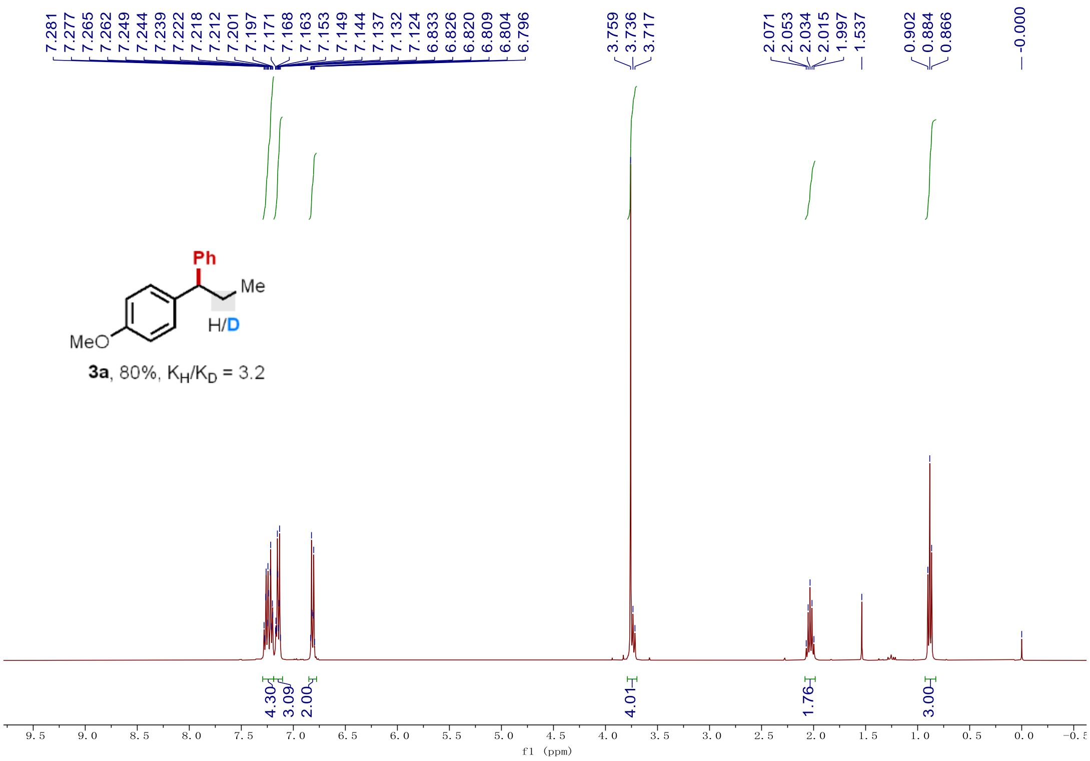

Supplementary Figure 17. ^{1}**H NMR (400 MHz,** $CDCl_3$**) spectrum of 3a**

3.2.3 Identification of Active Ar-Ni(II)-H

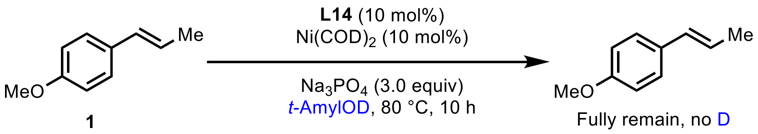

Following the general procedure, in a nitrogen-filled glovebox, substrate **1** (0.1 mmol, 1.0 equiv), $Ni(COD)_2$ (2.8 mg, 10 mol %), **L14** (3.4 mg, 10 mol %), Na_3PO_4 (49.2 mg, 3.0 equiv) were charged in a 10-mL tube. The tube was sealed using an open-top cap with PTFE cap liner, and moved outside of the glovebox, followed by the addition of *tert*-amyl alcohol (0.5 mL). The tube was sealed again with parafilm and heated to 80 °C for 10 h. After cooling to room temperature, the mixture was passed through a pad of silica gel with DCM as the eluent to remove the nickel and the insoluble precipitate. The resulting solution was concentrated. The residue was then purified by preparative thin-layer chromatography.

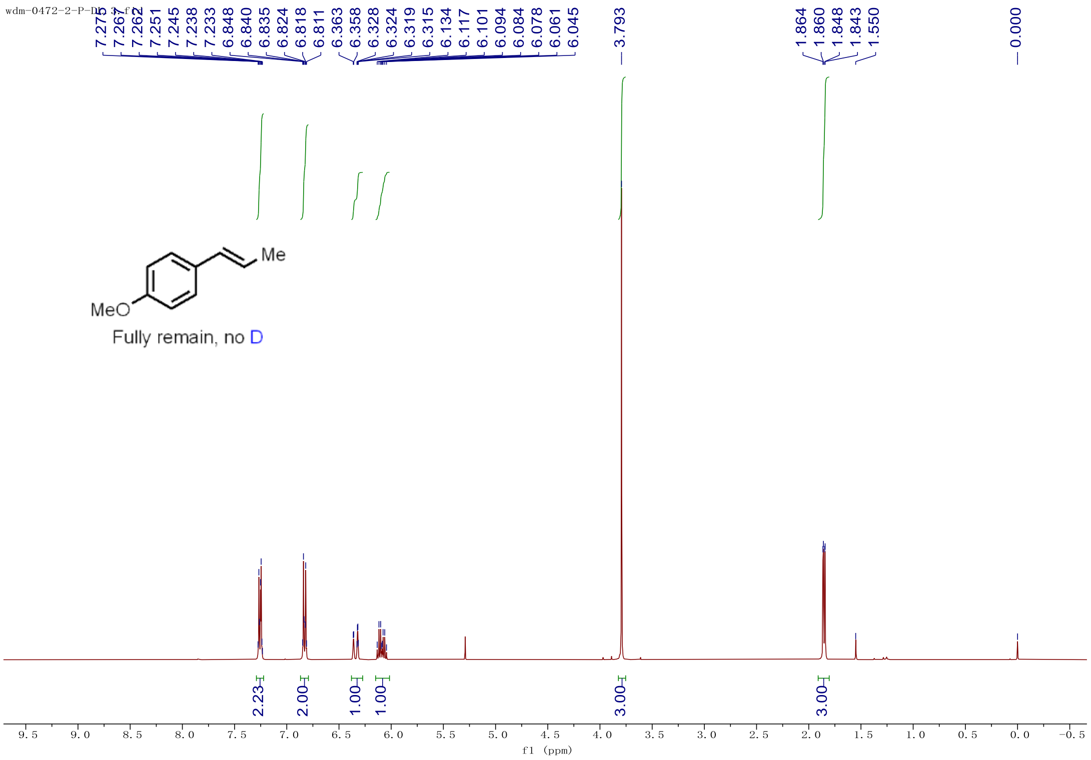

Supplementary Figure 18. **^{1}H NMR (400 MHz, $CDCl_3$) spectrum of 1**

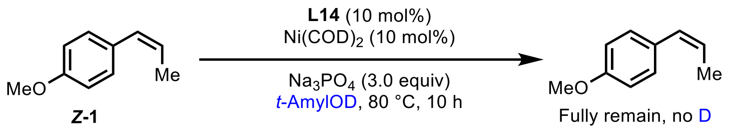

Following the general procedure, in a nitrogen-filled glovebox, substrate (*Z*)-**1** (0.1 mmol, 1.0 equiv), $Ni(COD)_2$ (2.8 mg, 10 mol %), **L14** (3.4 mg, 10 mol %), Na_3PO_4 (49.2 mg, 3.0 equiv) were charged in a 10-mL tube. The tube was sealed using an open-top cap with PTFE cap liner, and moved outside of the glovebox, followed by the addition of *tert*-amyl alcohol (0.5 mL). The tube was sealed again with parafilm and heated to 80 °C for 10 h. After cooling to room temperature, the mixture was passed through a pad of silica gel with DCM as the eluent to remove the nickel and the insoluble precipitate. The resulting solution was concentrated. The residue was then purified by preparative thin-layer chromatography.

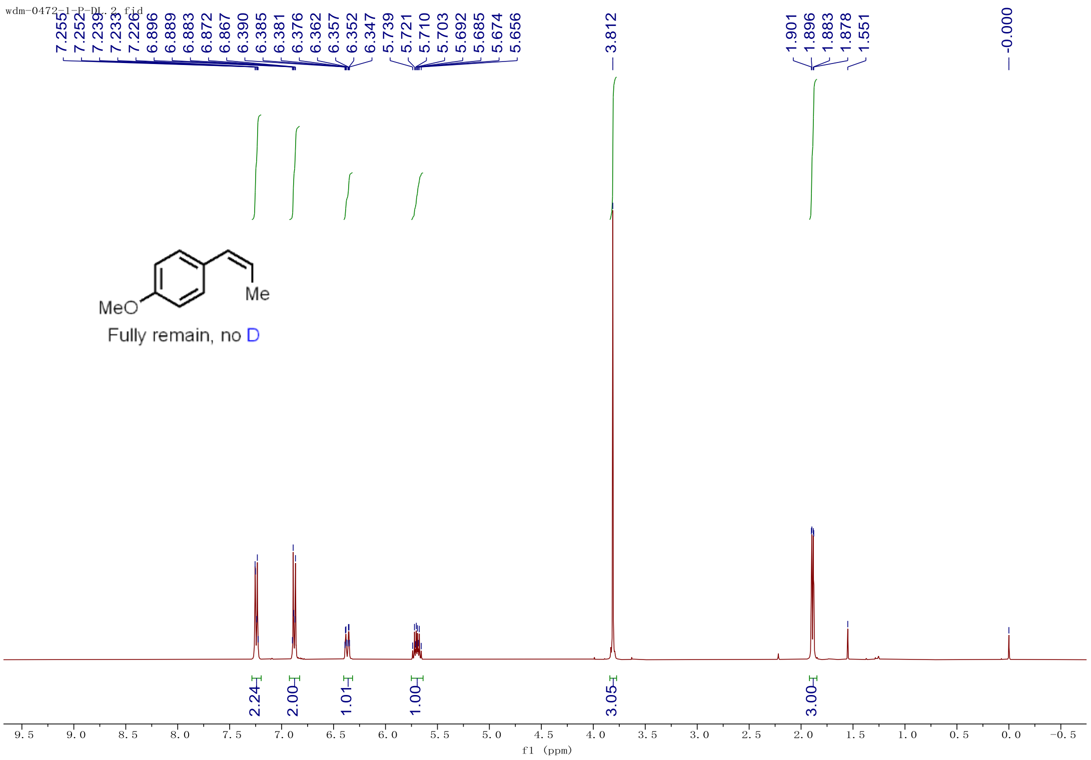

Supplementary Figure 19. ^{1}H NMR (400 MHz, CDCl$_3$) spectrum of (*Z*)-1

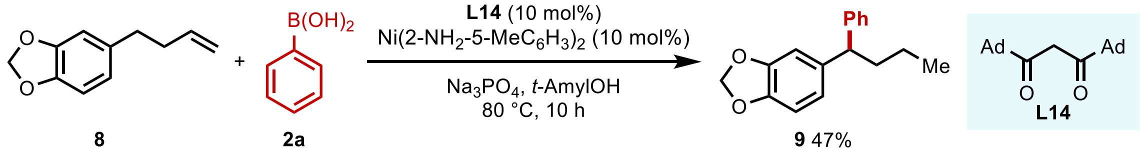

General Procedure: In a nitrogen-filled glovebox, substrate **8** (0.1 mmol, 1.0 equiv), **2a** (0.3 mmol, 3.0 equiv), Ni(2-NH$_2$-5-MeC$_6$H$_3$SO$_3$)$_2$ (4.3 mg, 10 mol %), **L14** (3.4 mg, 10 mol %), Na$_3$PO$_4$ (49.2 mg, 0.6 mmol, 3.0 equiv) were charged in a 10-mL tube. The tube was sealed using an open-top cap with PTFE cap liner, and moved outside of the glovebox, followed by the addition of *tert*-amyl alcohol (0.5 mL). The tube was sealed again with parafilm and heated to 80 ^{o}C for 10 h. After cooling to room temperature, the mixture was passed through a pad of silica gel with EtOAc as the eluent to remove the nickel and the insoluble precipitate. The resulting solution was concentrated. The residue was then purified by silica gel chromatography or preparative thin-layer chromatography to afford the hydroarylated product **9** as a colourless oil (11.9 mg, 47 % yield). ^{1}H NMR (400 MHz, CDCl$_3$) δ 7.30–7.19 (m, 4H), 7.19–7.13 (m, 1H), 6.71 (s, 3H), 5.89 (d, *J* = 1.4 Hz, 2H), 3.82 (t, *J* = 7.8 Hz, 1H), 1.96 (q, *J* = 7.6 Hz, 2H), 1.34–1.20 (m, *J* = 7.1 Hz, 2H), 0.91 (t, *J* = 7.4 Hz, 3H); ^{13}C NMR (100 MHz, CDCl$_3$) δ 147.59, 145.64, 145.40, 139.37, 128.36, 127.66, 126.00, 120.72, 108.21, 108.00, 100.76, 50.67, 37.96, 21.09, 14.04; HRMS (EI) *m/z* Calcd for $C_{17}H_{18}O_2$ [M]$^+$: 254.1301, found: 254.1302.

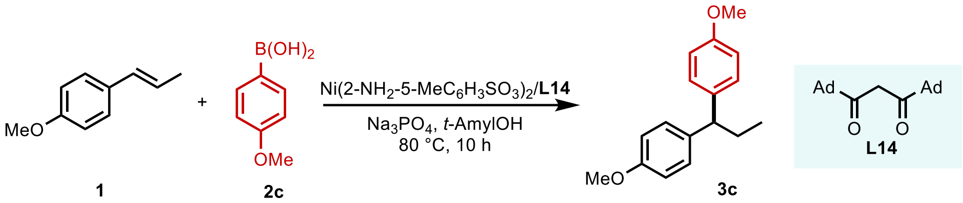

General Experimental Procedure: In a nitrogen-filled glovebox, substrate **1** (0.1 mmol, 1.0 equiv), **2c** (0.3 mmol, 3.0 equiv), $Ni(2\text{-}NH_2\text{-}5\text{-}MeC_6H_3SO_3)_2$ (4.3 mg, 10 mol %), **L14** (3.4 mg, 10 mol %), Na_3PO_4 (49.2 mg, 3.0 equiv) were charged in a 10-mL tube, and added 5.0 μL dodecane as internal standard. The tube was sealed using an open-top cap with PTFE cap liner, and moved outside of the glovebox, followed by the addition of *tert*-amyl alcohol (1.0 mL). The tube was sealed again with parafilm and heated to 80 °C for 10 h. After cooling to room temperature, the mixture was passed through a pad of silica gel with EtOAc as the eluent to remove the nickel and the insoluble precipitate. The mixture was filtered with a filter head into 2.0 mL GC vial and analyzed by gas chromatography.

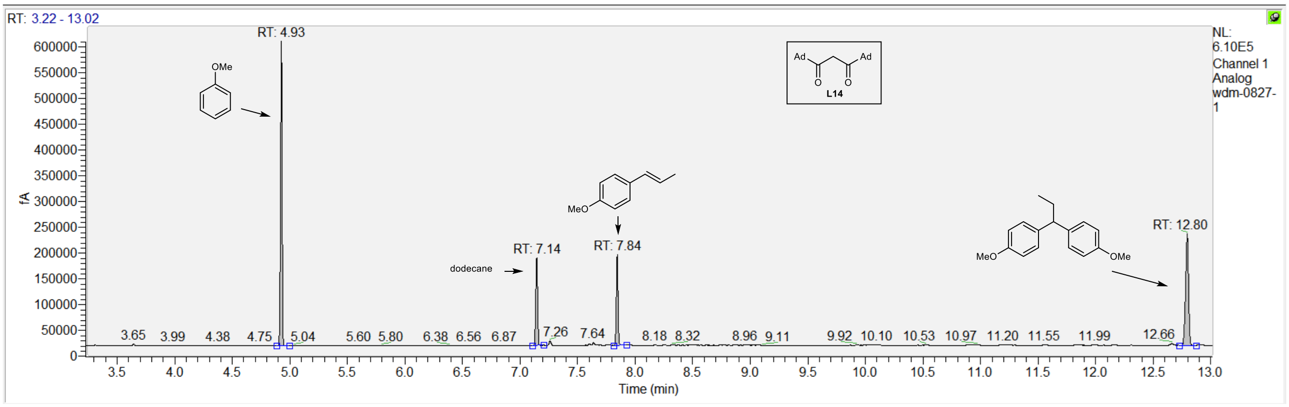

Apex RT	Start RT	End RT	Area	%Area	Height	%Height
4.93	4.88	4.99	597894.435	44.05	589603.5	51.21
7.14	7.11	7.2	185072.453	13.64	169447.5	14.72
7.84	7.81	7.92	186404.691	13.73	175421.7	15.23
12.8	12.73	12.88	387815.496	28.57	216975.8	18.84

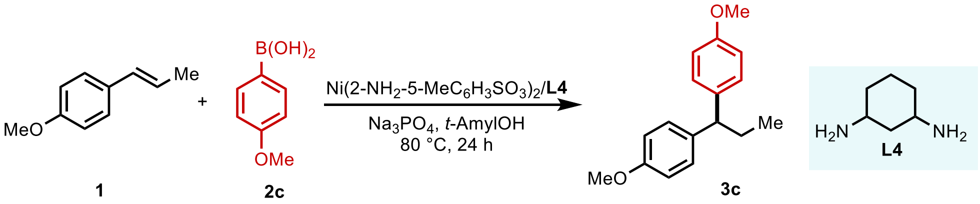

General Experimental Procedure: In a nitrogen-filled glovebox, substrate **1** (0.1 mmol, 1.0 equiv), **2c** (0.3 mmol, 3.0 equiv), $Ni(2\text{-}NH_2\text{-}5\text{-}MeC_6H_3SO_3)_2$ (4.3 mg, 10 mol %), **L4** (1.1 mg, 10 mol %), Na_3PO_4 (49.2 mg, 3.0 equiv) were charged in a 10-mL tube, and added 5.0 μL dodecane as internal standard. The tube was sealed using an open-top cap with PTFE cap liner, and moved outside of the glovebox, followed by the addition of *tert*-amyl alcohol (1.0 mL). The tube was sealed again with parafilm and heated to 80 °C for 10 h. After cooling to room temperature, the mixture was passed through a pad of silica gel with EtOAc as the eluent to remove the nickel and the insoluble precipitate. The mixture was filtered with a filter head into 2.0 mL GC vial and analyzed by gas chromatography.

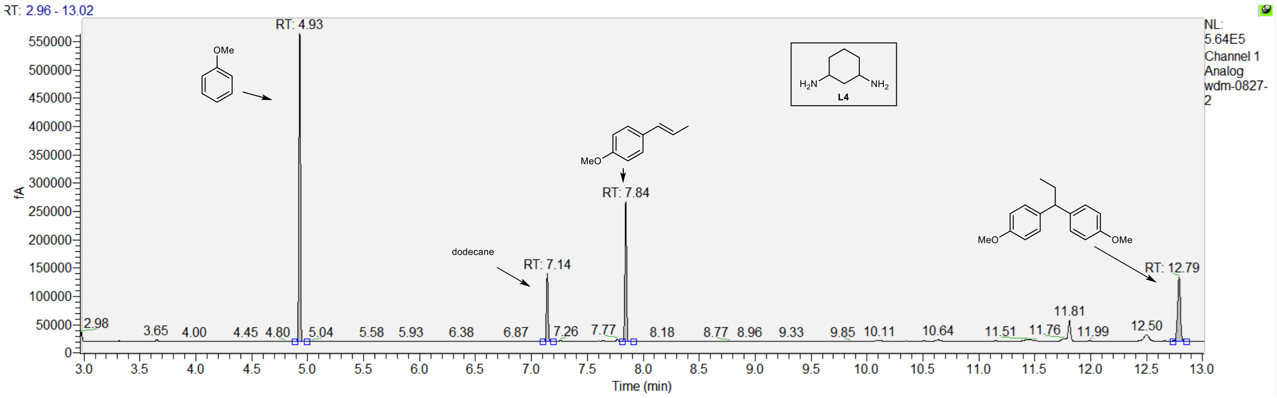

Apex RT	Start RT	End RT	Area	%Area	Height	%Height
4.93	4.88	4.99	554988.598	48.3	543134.9	53.1
7.14	7.1	7.19	130559.184	11.36	119386	11.67
7.84	7.81	7.91	263104.017	22.9	246500.4	24.1
12.79	12.73	12.85	200414.322	17.44	113853.9	11.13

To demonstrate the ligand effect on stabilizing the Ar-Ni(II)-H intermediate, we carried out the experiments with ligand **L4** and **L14**, as other ligands resulted in no reaction. The control experiments unveiled that our ligand **L14** could inhibit the formation of protonation byproduct from arylboronic acid, which supports our hypothesis.

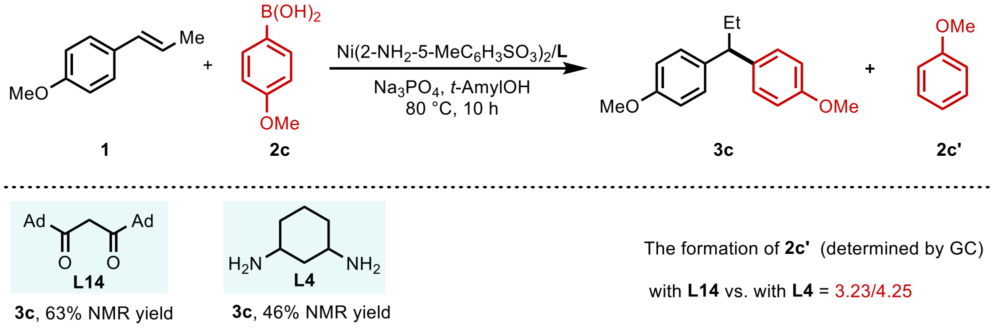

3.3 Synthesis of Ni(Adacac)$_2$[20]

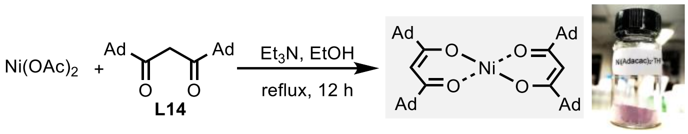

To a dry Schlenk flask was subsequentially added the ligand **L14** (1.4 g, 4.1 mmol, 2.05 equiv), Ni(OAc)$_2$ (353.6 mg, 2.0 mmol, 1.0 equiv), and dry EtOH (10 mL). Then Et$_3$N (0.84 mL, 6.0 mmol, 3.0 equiv) was added. The reaction mixture was heated to 80 ^{o}C and stirred for 12 h. After cooling to room temperature, the mixture was filtered and the precipitate was subsequentially washed with H_2O, EtOH and THF to afford a green solid. After remove the THF under vacuum at 80 ^{o}C, a purple solid [Ni(Adacac)$_2$(THF)] was obtained in 74% yield (1.2 g). The green solid was assigned to the Ni(Adacac)$_2$(THF)$_2$. Anal. calcd for [Ni(Adacac)$_2$(THF)] $C_{50}H_{70}NiO_5$ (808.46): C, 74.16; H, 8.71. Found: C, 73.92; H, 8.52.

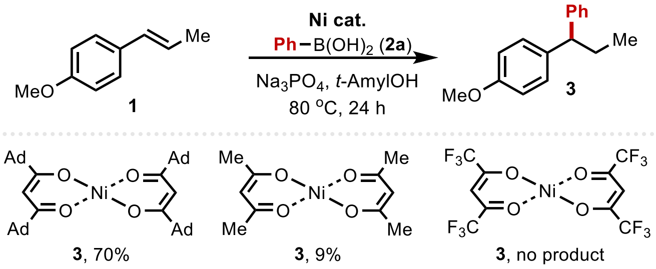

We also checked the efficiency of the Ni(Adacac)$_2$ in the Ni-catalyzed hydroarylation of internal alkenes, and this new Ni(II) precursor showed better reactivity than commercially available Ni(acac)$_2$ and Ni(Hfacac)$_2$.

4. X-Ray Structure of $Ni(Adacac)_2(THF)_2$

Single crystals of $Ni(Adacac)_2(THF)_2$ (green) were obtained by recrystallization from THF. The molecular structure and X-ray diffractional data/refinement of $Ni(Adacac)_2(THF)_2$ were shown below.

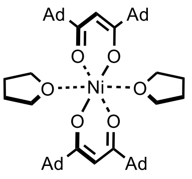

$Ni(Adacac)_2(THF)_2$

≡

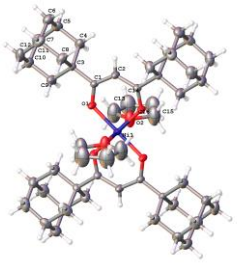

Bond precision: C-C = 0.0082 A Wavelength=1.34139

Cell: a=26.6950(6) b=9.1351(2) c=19.8265(5)

alpha=90 beta=90 gamma=90

Temperature: 214 K

	Calculated	Reported
Volume	4834.92(19)	4834.92(19)
Space group	C m c a	C m c e
Hall group	-C 2bc 2	-C 2bc 2
Moiety formula	C54 H78 Ni O6	C54 H78 Ni O6
Sum formula	C54 H78 Ni O6	C54 H78 Ni O6
Mr	881.85	881.87
Dx,g cm-3	1.212	1.212
Z	4	4
Mu (mm-1)	2.426	2.426
F000	1912.0	1912.0
F000'	1910.36	
h,k,lmax	32,11,24	32,11,24
Nref	2362	2346
Tmin,Tmax	0.844,0.886	0.522,0.751
Tmin'	0.844	

Correction method= # Reported T Limits: Tmin=0.522 Tmax=0.751 AbsCorr = MULTI-SCAN

Data completeness= 0.993 Theta(max)= 54.956

R(reflections)= 0.0734(1965) wR2(reflections)= 0.2423(2346)

S = 1.093 Npar= 148

5. References

[1] Liu, H.; Xu, M.; Cai, C.; Chen, J.; Gu, Y.; Xia, Y. *Org. Lett.* **2020**, *22*, 1193−1198.

[2] Jiang, X.; Han, B.; Xue, Y.; Duan, M.; Gui, Z.; Wang, Y.; Zhu, S. *Nat. Commun.* **2021**, *12*, 3792.

[3] Li, Y.-H.; Wang, C.-H.; Gao, S.-Q.; Qia, F.-M.; Yang, S.-D. *Chem. Commun*., **2019**, *55*, 11888−11891.

[4] Shi, Y.; Kamer, P. C. J.; Cole-Hamilton, D. J. *Green Chem*. **2019**, *21*, 1043–1053.

[5] Logan, K.M.; Brown, M. K. *Angew. Chem. Int. Ed*. **2017**, *56*, 851–855.

[6] Colomer, I.; Batchelor-McAuley, C.; Odell, B.; Donohoe, T. J.; Compton, R. G. *J. Am. Chem. Soc*. **2016**, *138*, 8855−8861.

[7] Yasir, M.; Liu, P.; Tennie, I. K.; Kilbinger, A. F. M. *Nat. Chem*. **2019**, *11*, 488–494.

[8] Zhou, B.; Hu, Y.; Wang, C. *Angew. Chem. Int. Ed.* **2015**, *54*, 13659 –13663.

[9] Yu, W.-L.; Chen, J.-Q.; Wei, Y.-L.; Wang, Z.-Y.; Xu, P.-F. *Chem. Commun*., **2018**, *54*, 1948–1951.

[10] Chen, F.; Chen, K.; Zhang, Y.; He, Y.; Wang, Y.-M.; Zhu, S. *J. Am. Chem. Soc*. **2017**, *139*, 13929−13935.

[11] Do, H.-Q.; Chandrashekar, E. R. R.; Fu, G. C. *J. Am. Chem. Soc*. **2013**, *135*, 16288−16291.

[12] Podder, S.; Choudhury, J.; Roy, U. K.; Roy, S. *J. Org. Chem.* **2007**, *72*, 3100–3103.

[13] He, Y.; Liu, C.; Yu, L.; Zhu, S. *Angew. Chem. Int. Ed.* **2020**, *59*, 21530 –21534.

[14] Zhang, W.; Wu, L.; Chen, P.; Liu, G. *Angew. Chem. Int. Ed*. **2019**, *58*, 6425 –6429.

[15] Gu, F.; Huang, W.; Liu, X.; Chen, W. Cheng, X. *Adv. Synth. Catal*. **2018**, *360*, 925 – 931.

[16] Fessard, T. C.; Motoyoshi, H.; Carreira, E. M. *Angew. Chem. Int. Ed.* **2007**, *46*, 2078 –2081.

[17] Li, J.; He, L.; Liu, X.; Cheng, X.; Li, G. *Angew. Chem. Int. Ed*. **2019**, *58*, 1759 –1763.

[18] Friedfeld, M. R.; Shevlin, M.; Margulieux, G. W.; Campeau, L.-C.; Chirik, P. J. *J. Am. Chem. Soc*. **2016**, *138*, 3314−3324.

[19] Peng, L.; Li, Z.; Yin, G. *Org. Lett*. **2018**, *20*, 1880−1883.

[20] Kołodziejski, M.; Walczak, A.; Hnatejko, Z.; Harrowfield, J.; Stefankiewicz, A. R. *Polyhedron* **2017**, *137*, 270–277.

6. NMR Spectra

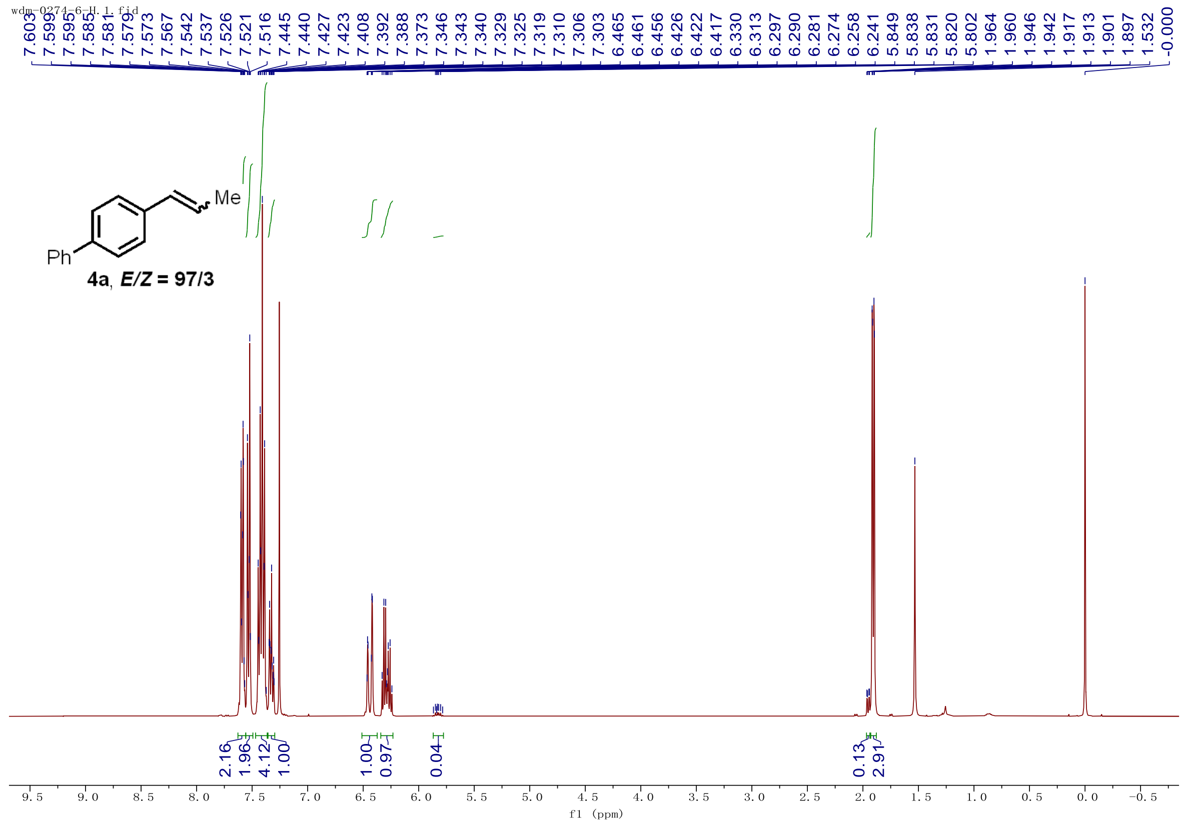

Supplementary Figure 20. ^{1}H NMR (400 MHz, $CDCl_3$) spectrum of 4a

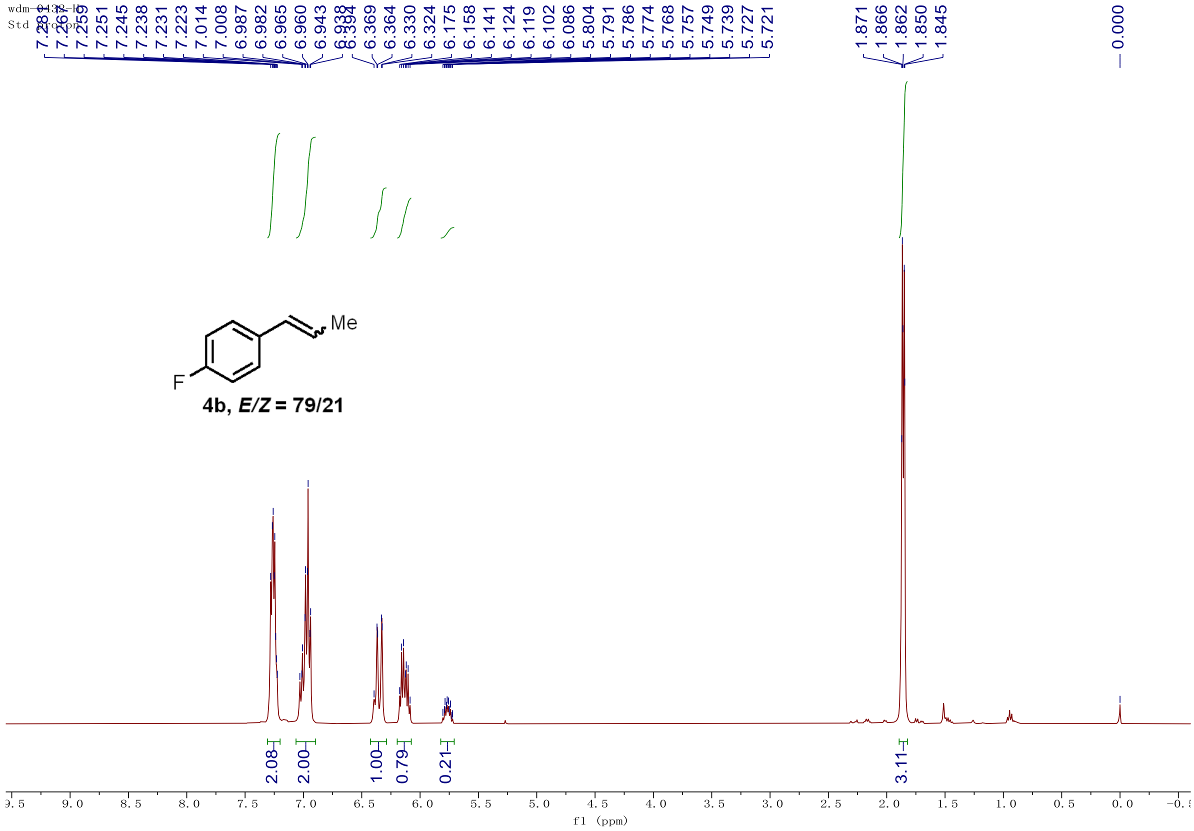

Supplementary Figure 21. ^{1}H NMR (400 MHz, $CDCl_3$) spectrum of 4b

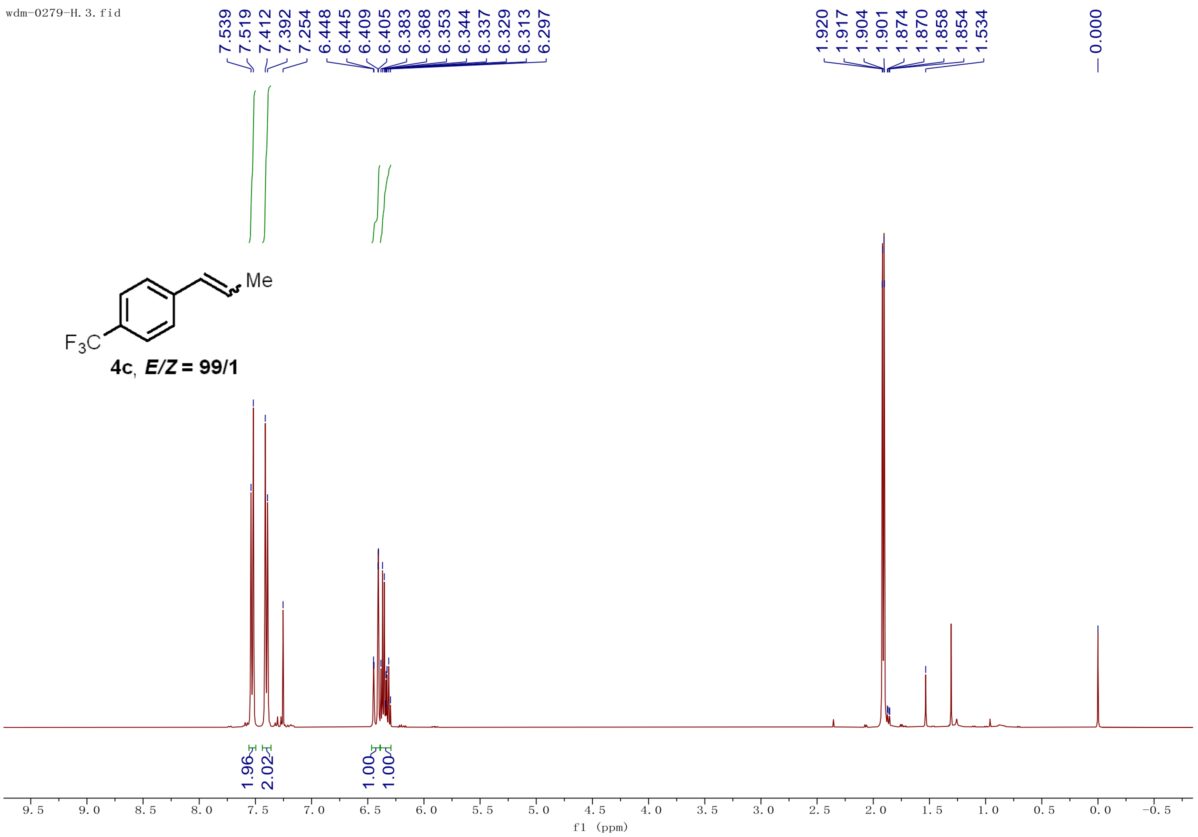

Supplementary Figure 22. ^{1}**H NMR (400 MHz,** $CDCl_3$**) spectrum of 4c**

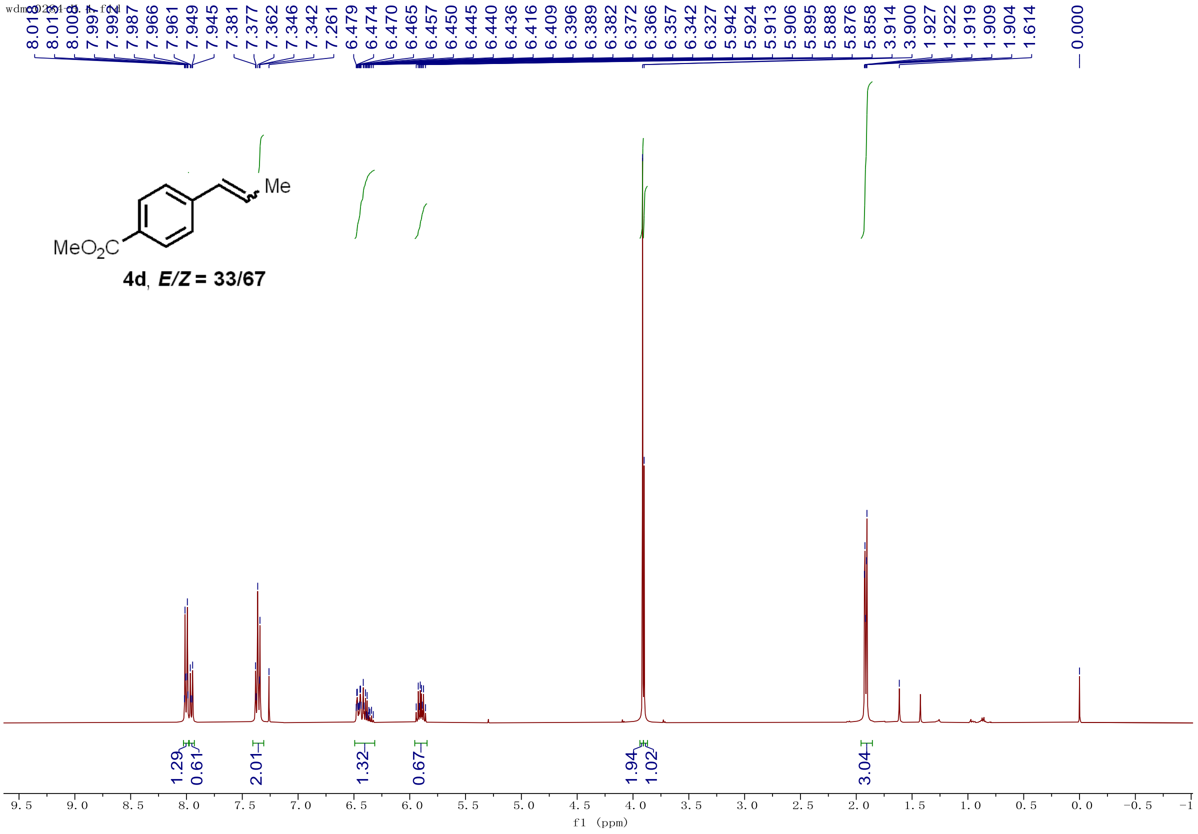

Supplementary Figure 23. ^{1}**H NMR (400 MHz,** $CDCl_3$**) spectrum of 4d**

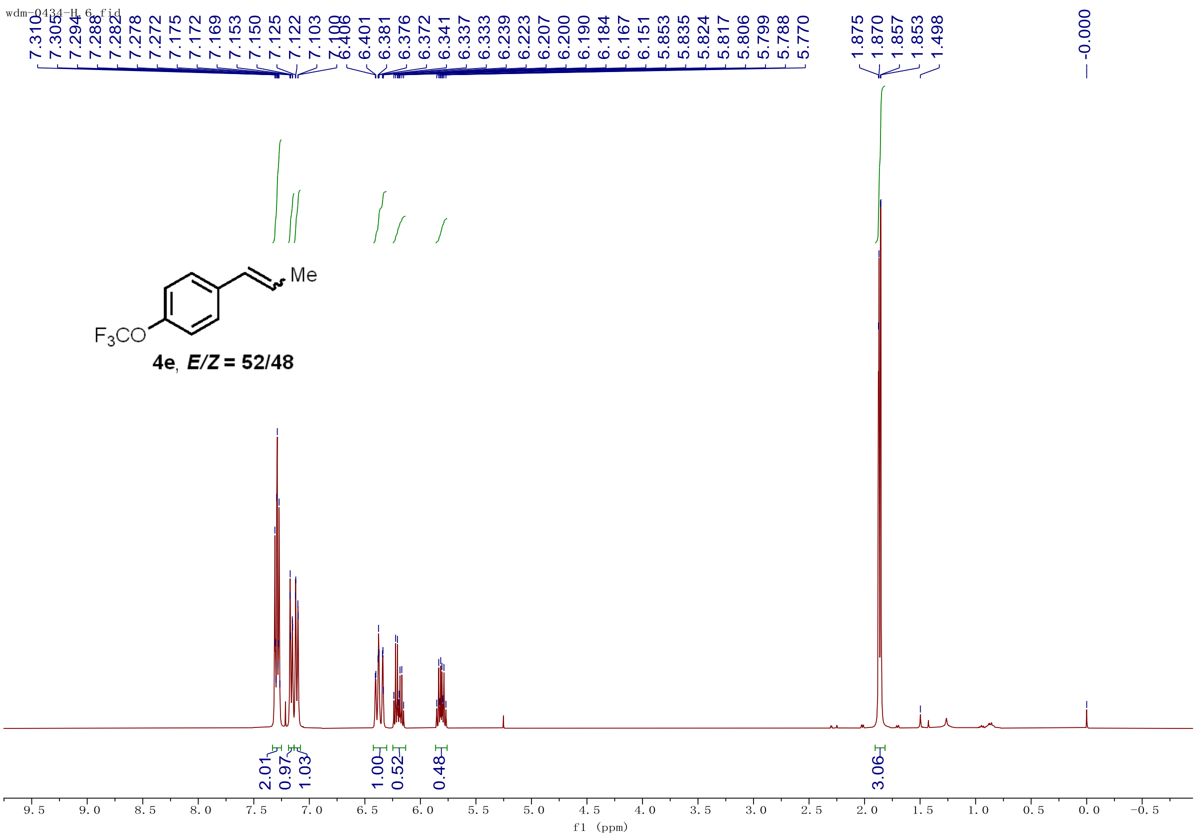

Supplementary Figure 24. ^{1}**H NMR (400 MHz,** $CDCl_3$**) spectrum of 4e**

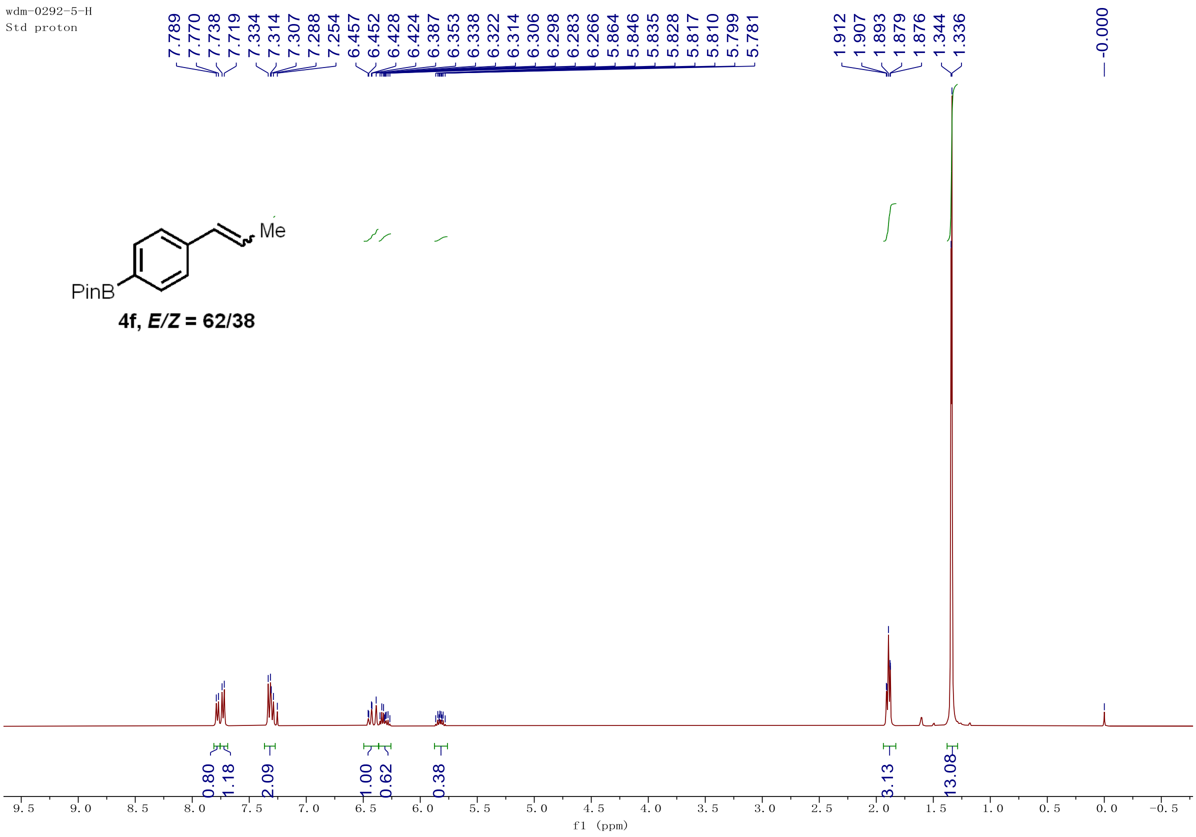

Supplementary Figure 25. ^{1}**H NMR (400 MHz,** $CDCl_3$**) spectrum of 4f**

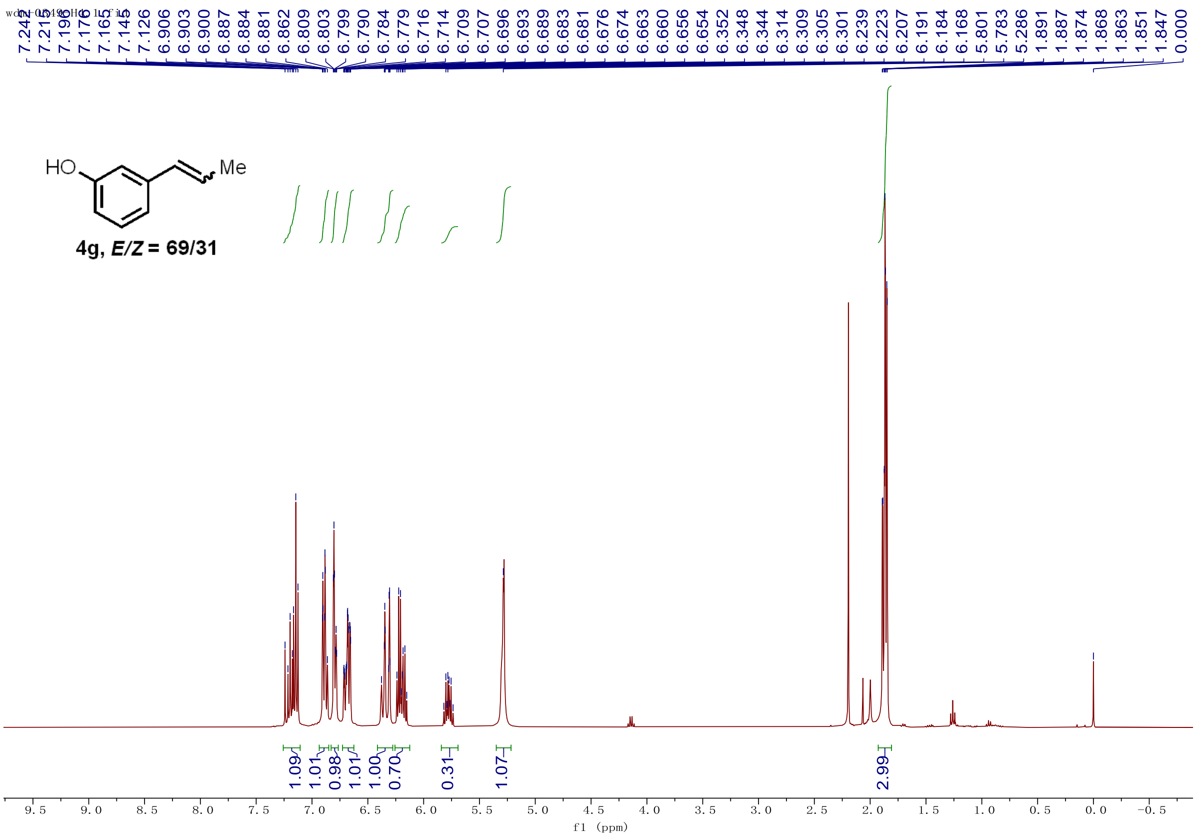

Supplementary Figure 26. ^{1}H NMR (400 MHz, $CDCl_3$) spectrum of 4g

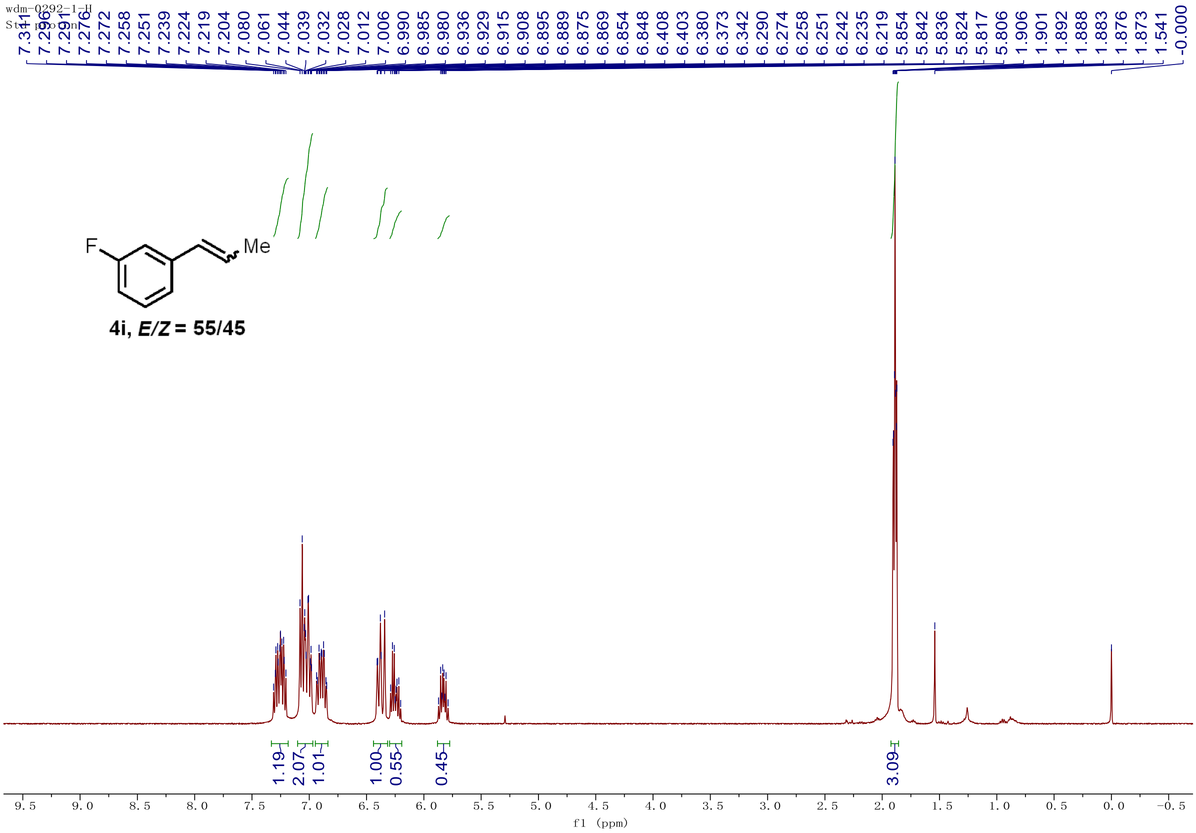

Supplementary Figure 28. ^{1}H NMR (400 MHz, $CDCl_3$) spectrum of 4i

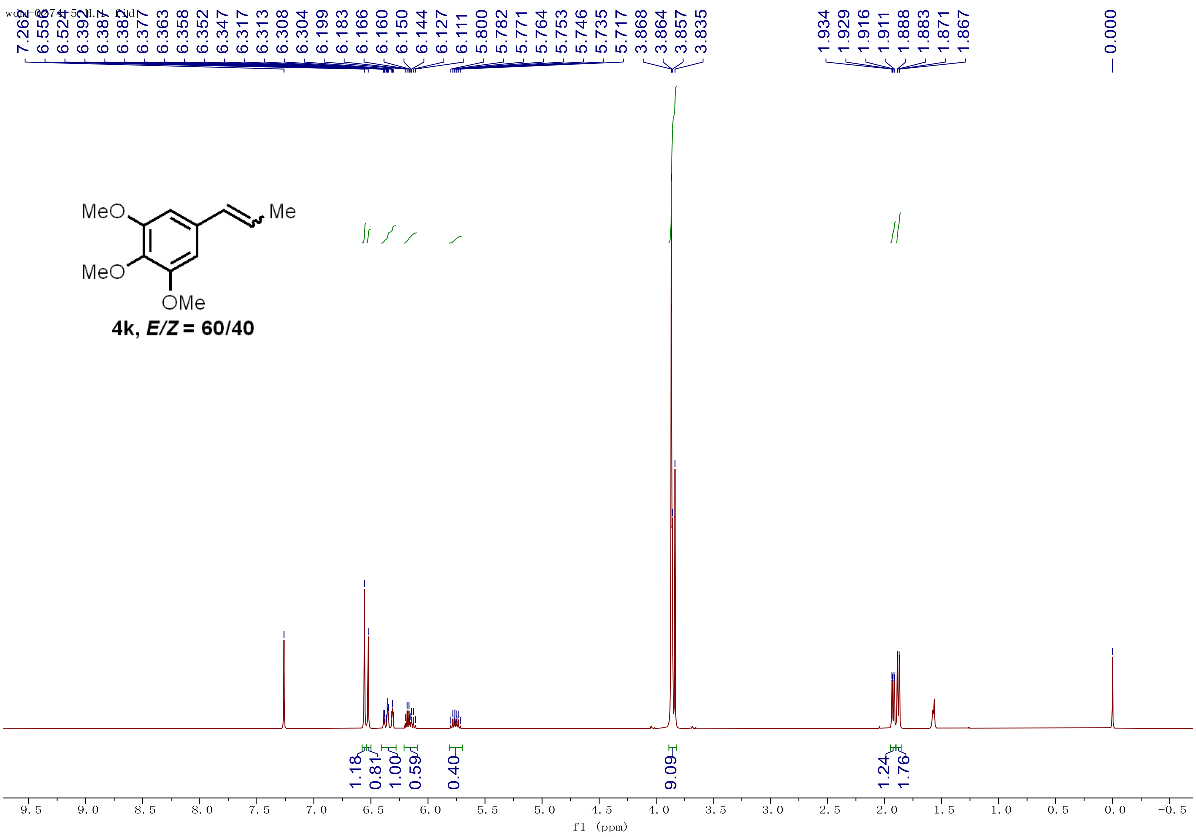

Supplementary Figure 29. ^{1}H NMR (400 MHz, $CDCl_3$) spectrum of 4k

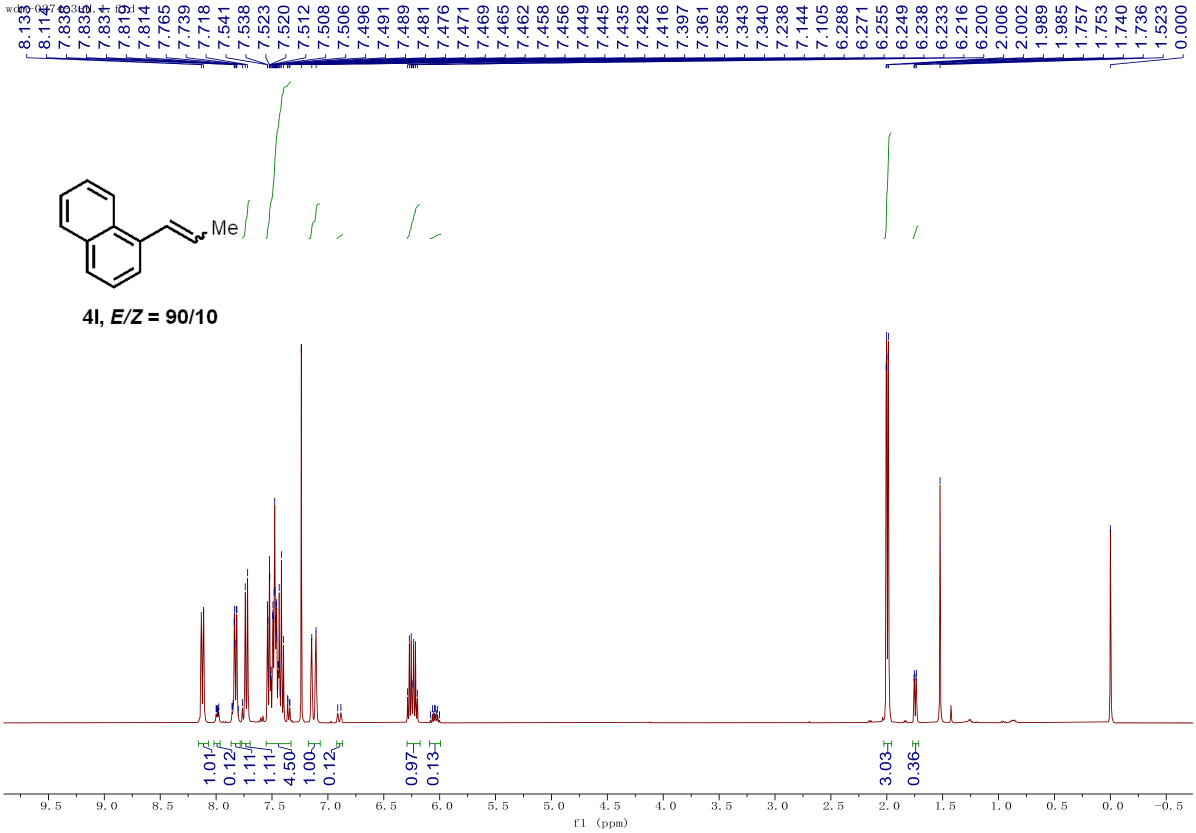

Supplementary Figure 30. ^{1}H NMR (400 MHz, $CDCl_3$) spectrum of 4l

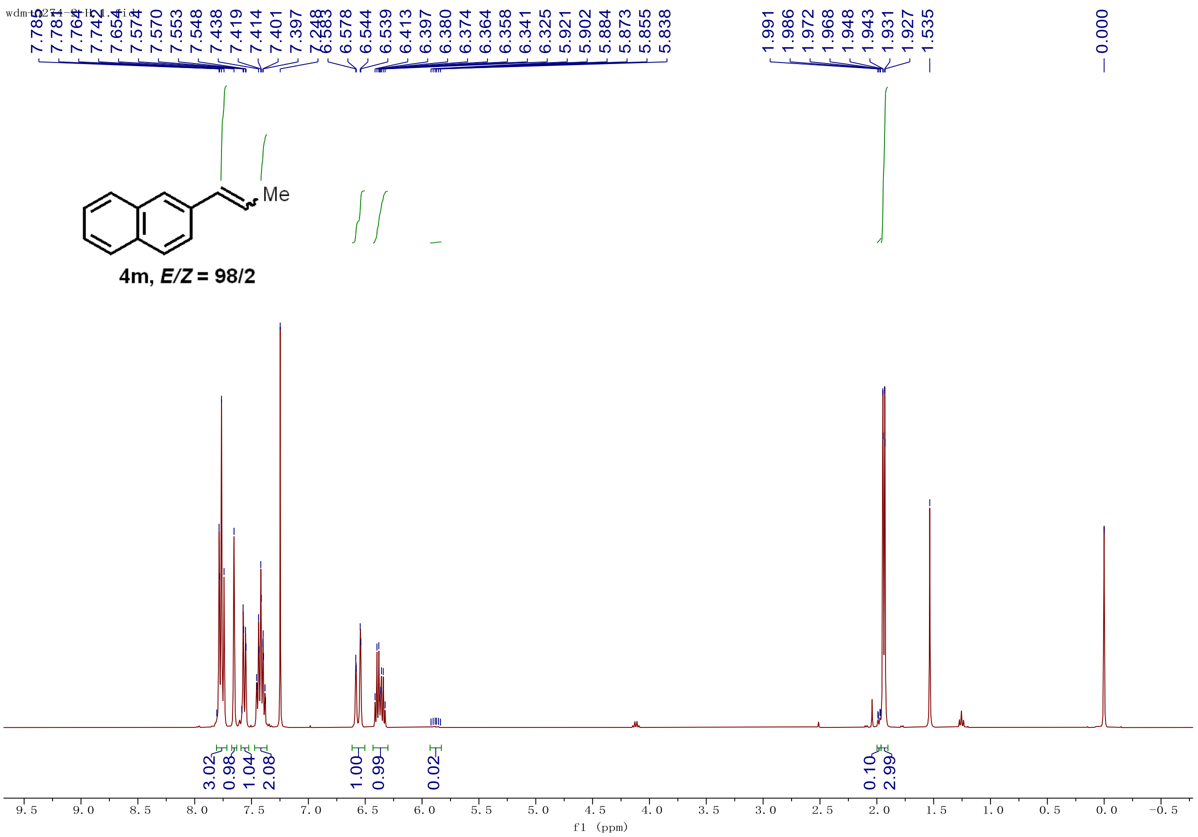

Supplementary Figure 31. ^{1}**H NMR (400 MHz, CDCl**$_3$**) spectrum of 4m**

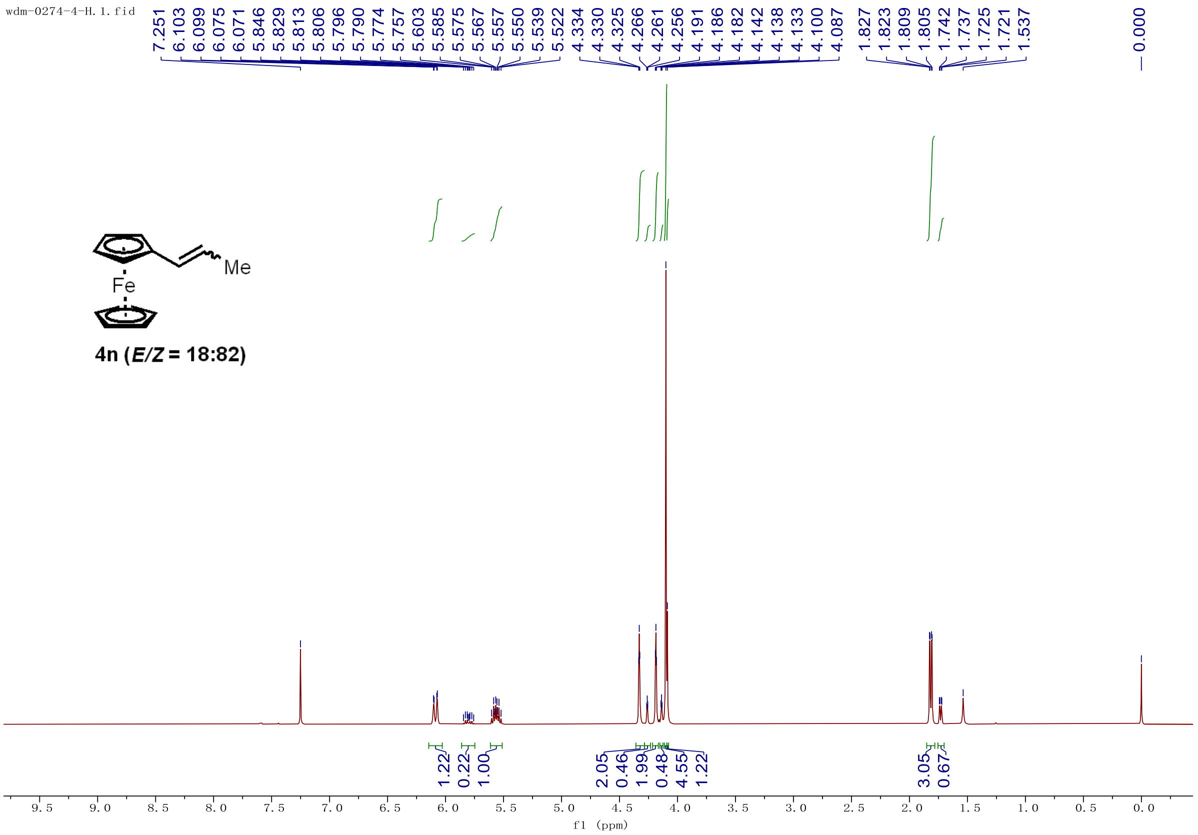

Supplementary Figure 32. ^{1}**H NMR (400 MHz, CDCl**$_3$**) spectrum of 4n**

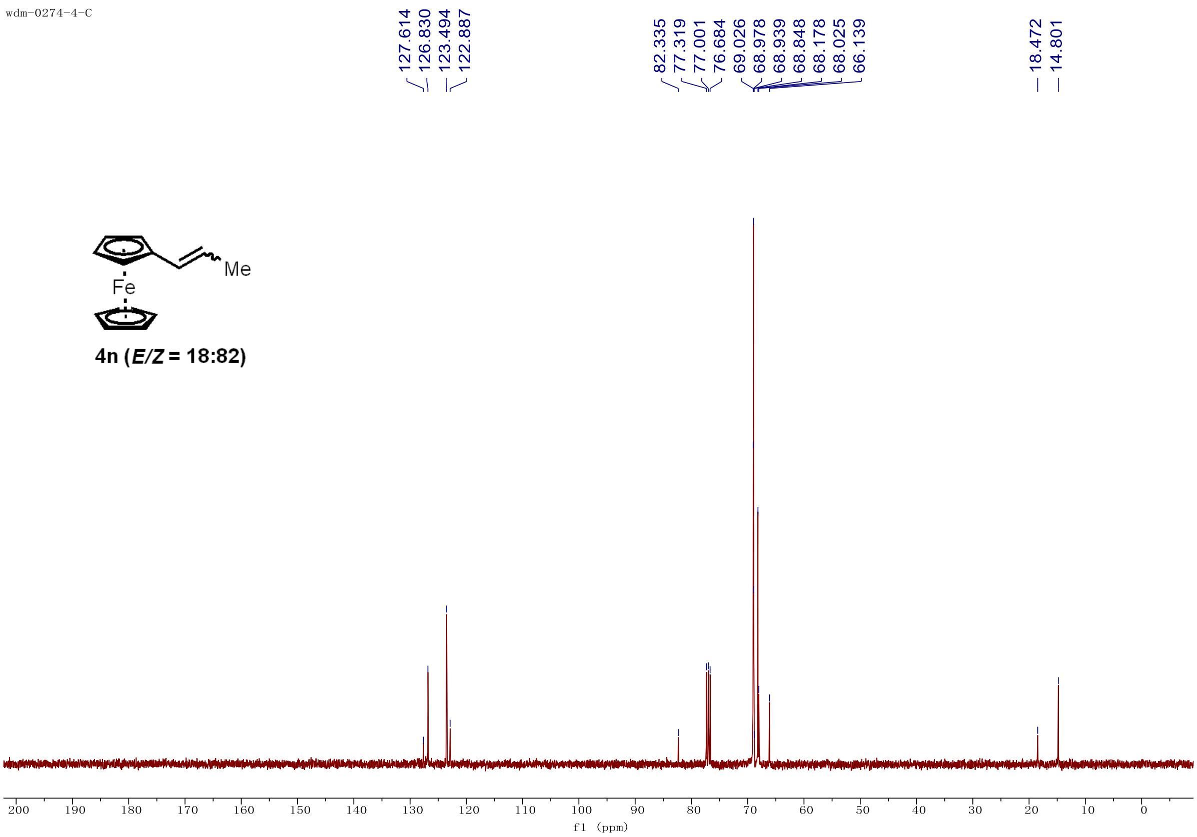

Supplementary Figure 33. ^{13}C NMR (100 MHz, CDCl$_3$) spectrum of 4n

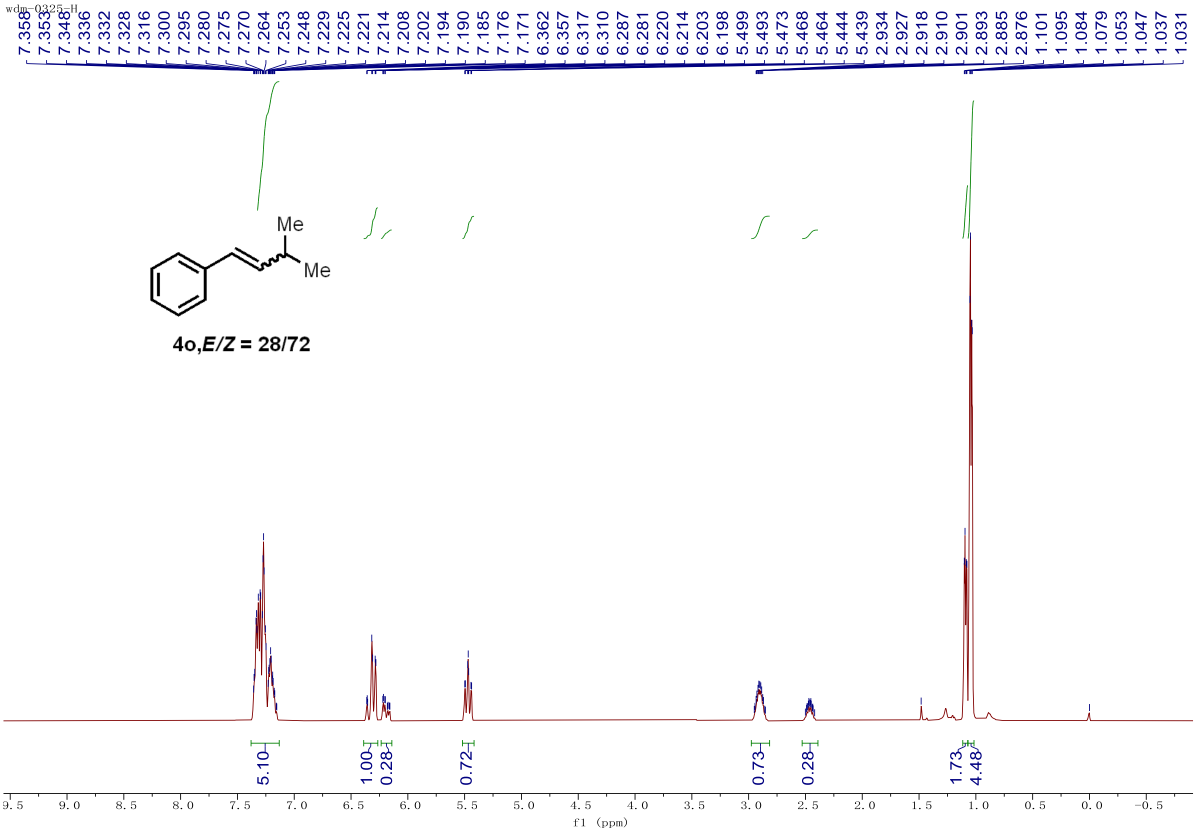

Supplementary Figure 34. ^{1}H NMR (400 MHz, CDCl$_3$) spectrum of 4o

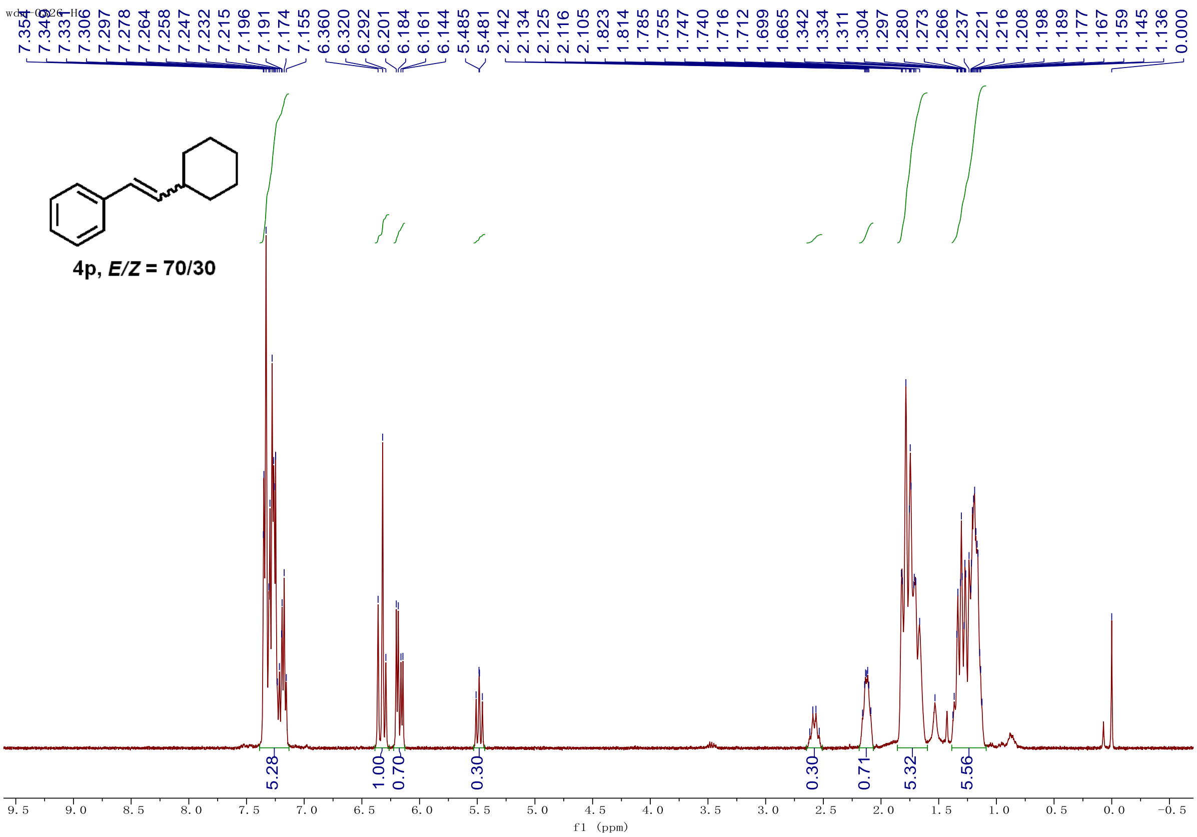

Supplementary Figure 35. ^{1}**H NMR (400 MHz, CDCl**$_3$**) spectrum of 4p**

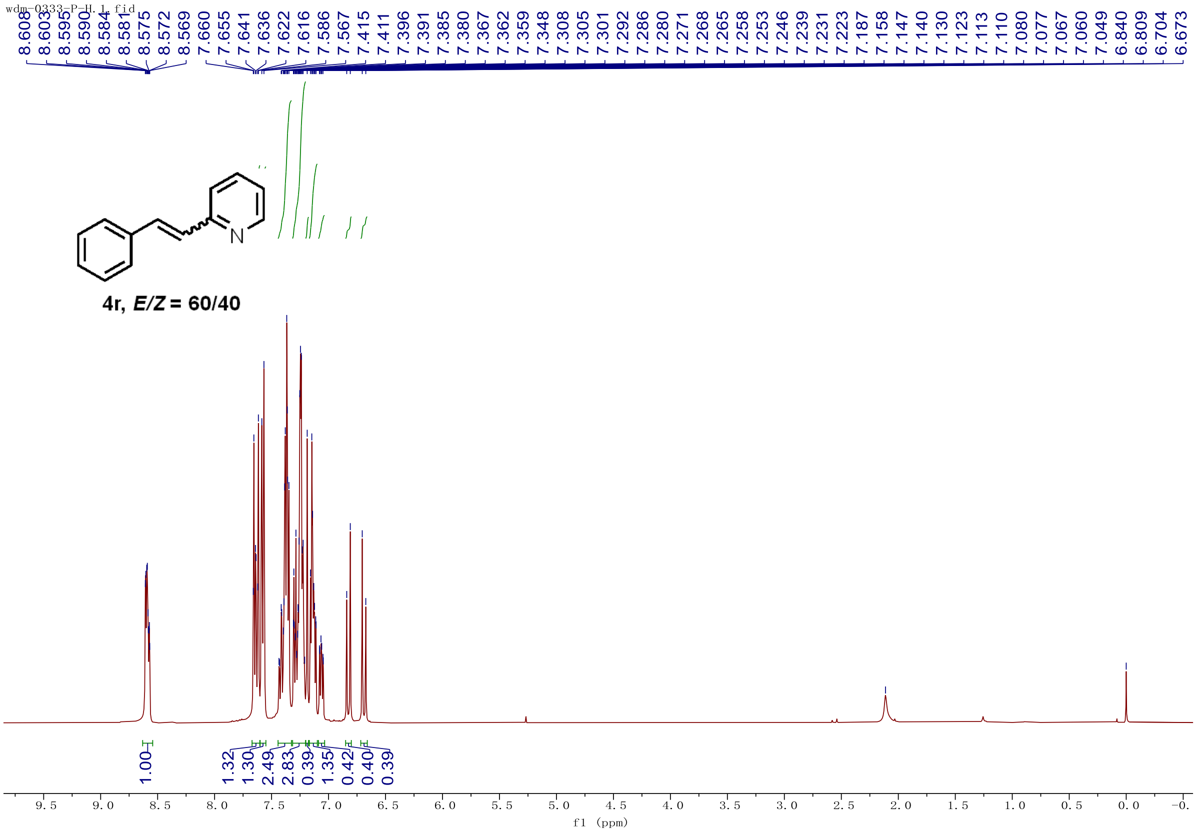

Supplementary Figure 36. ^{1}**H NMR (400 MHz, CDCl**$_3$**) spectrum of 4r**

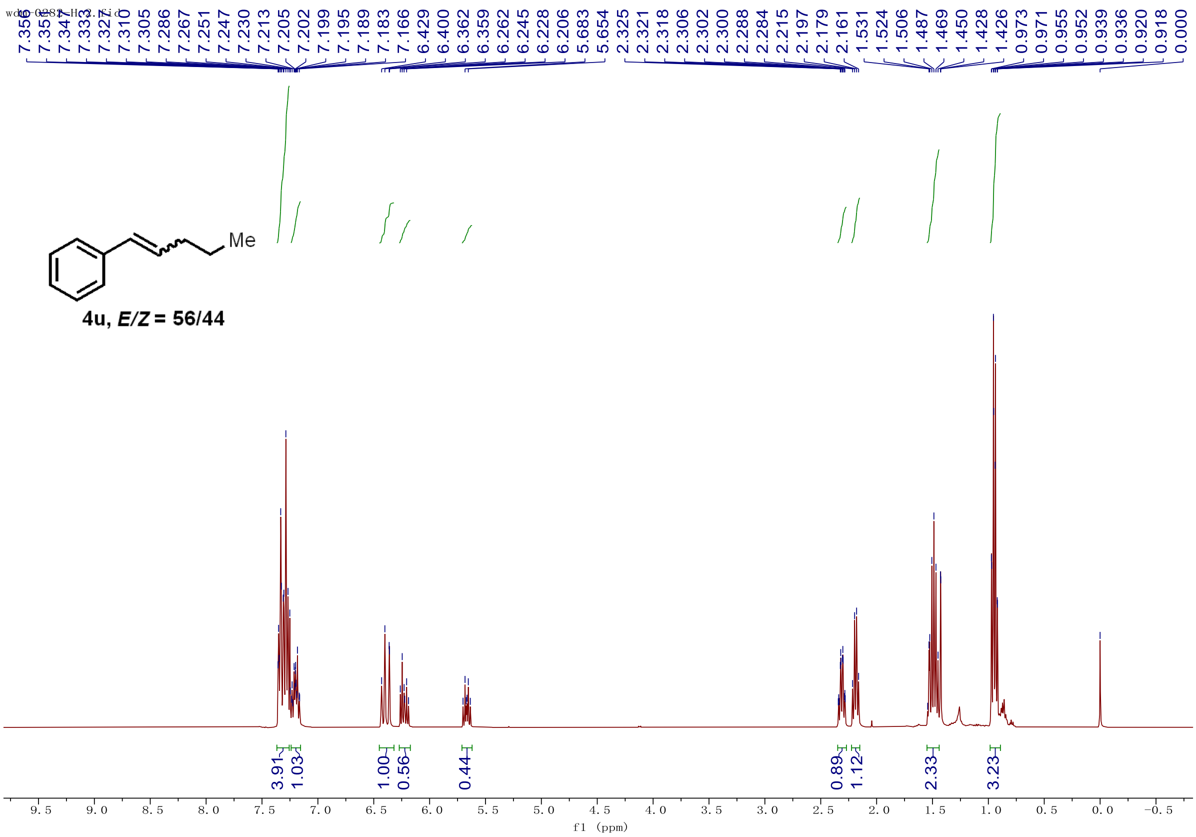

Supplementary Figure 37. ^{1}H NMR (400 MHz, $CDCl_3$) spectrum of 4u

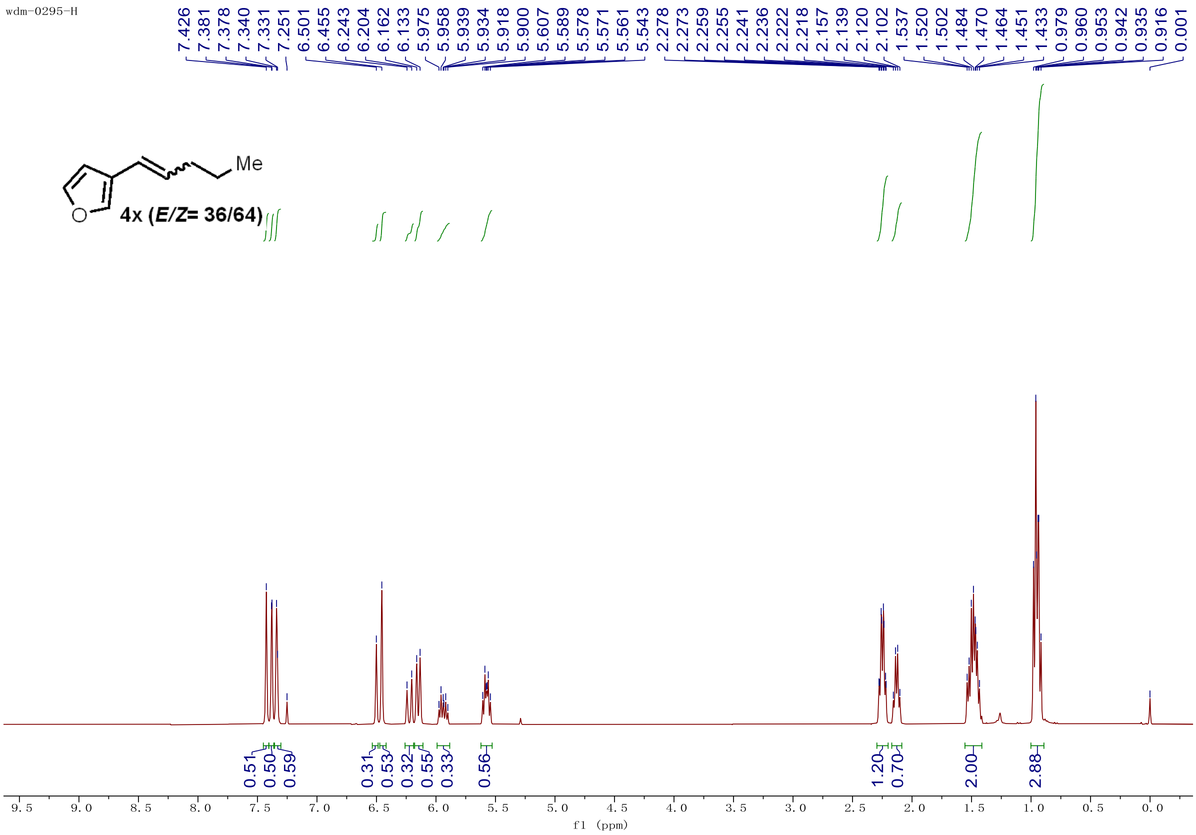

Supplementary Figure 38. ^{1}H NMR (400 MHz, $CDCl_3$) spectrum of 4x

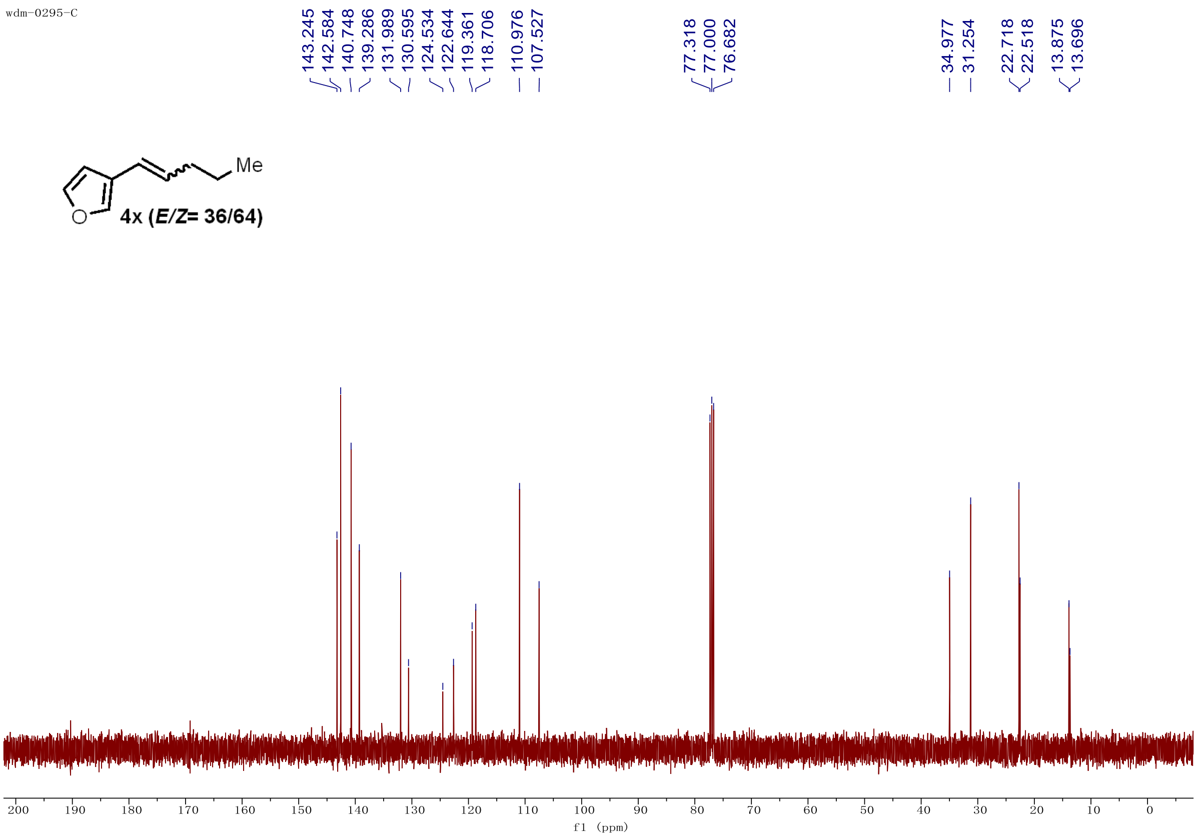

Supplementary Figure 39. ^{13}C NMR (100 MHz, $CDCl_3$) spectrum of 4x

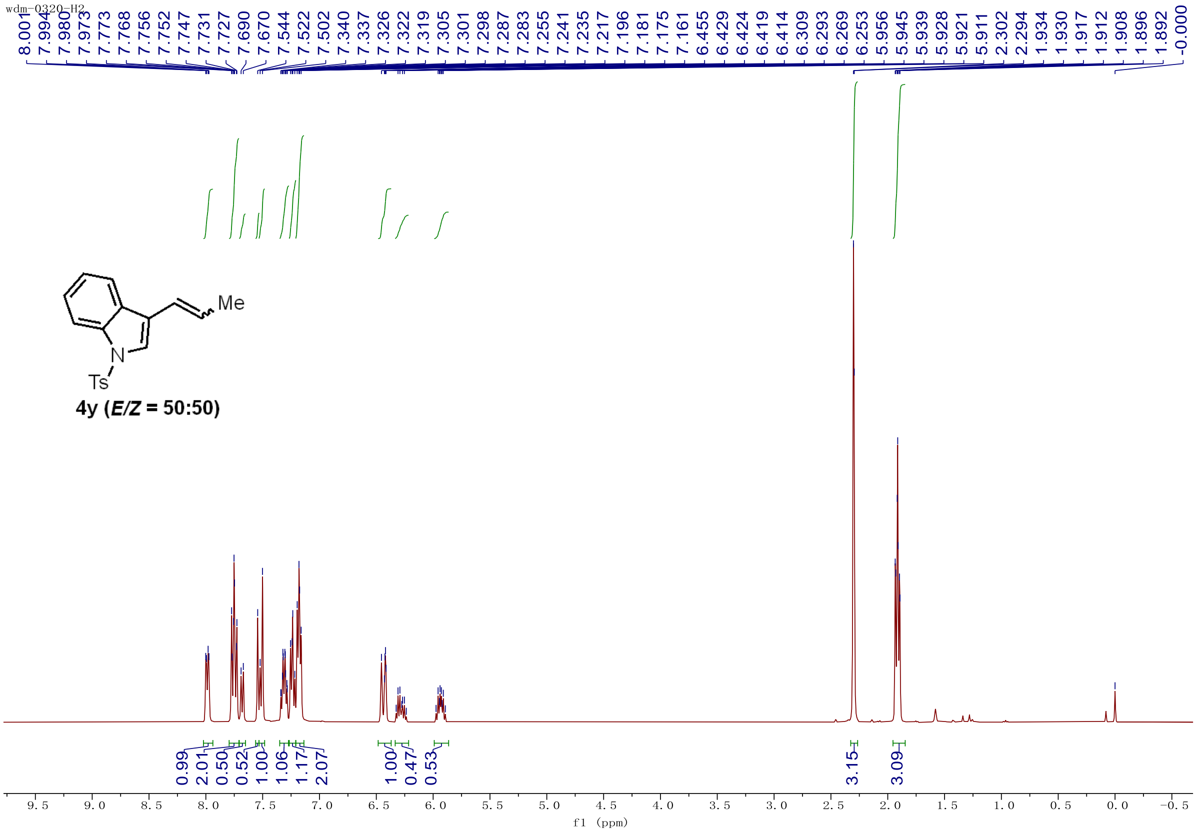

Supplementary Figure 40. ^{1}H NMR (400 MHz, $CDCl_3$) spectrum of 4y

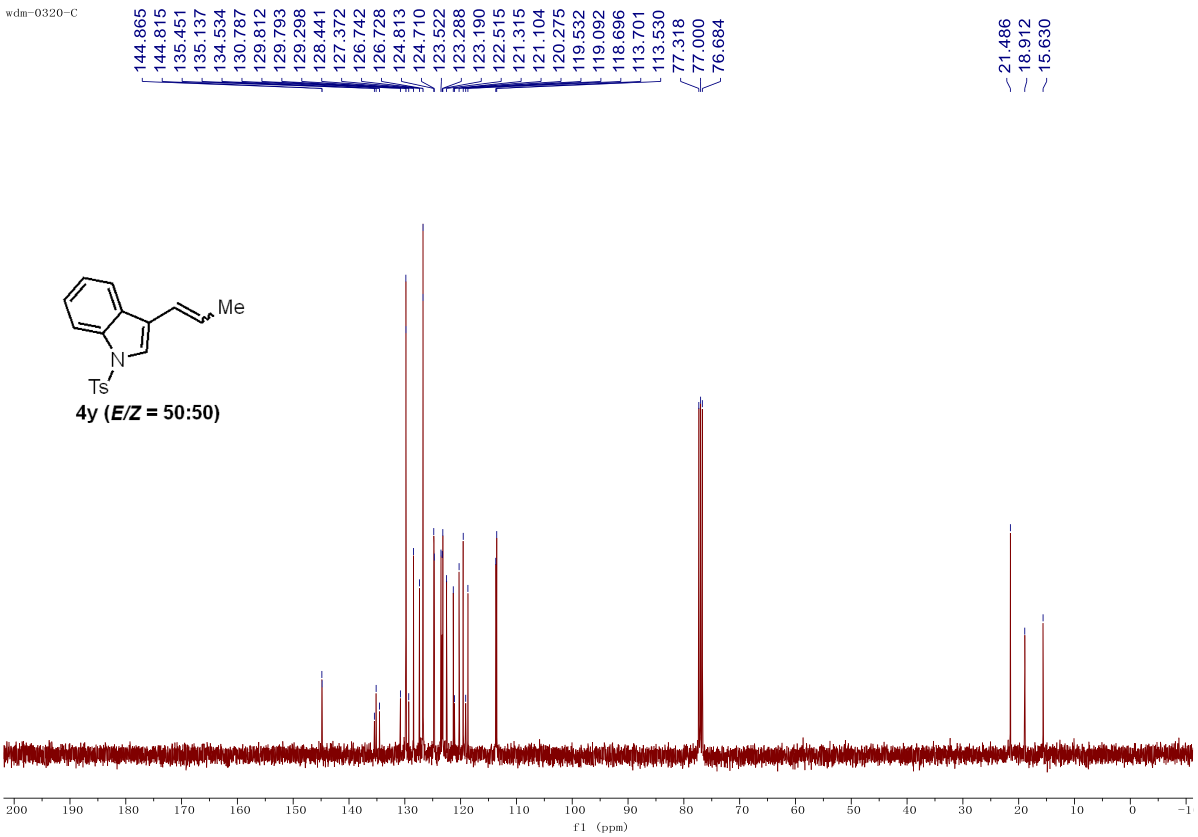

Supplementary Figure 41. ^{13}**C NMR (100 MHz, CDCl**$_3$**) spectrum of 4y**

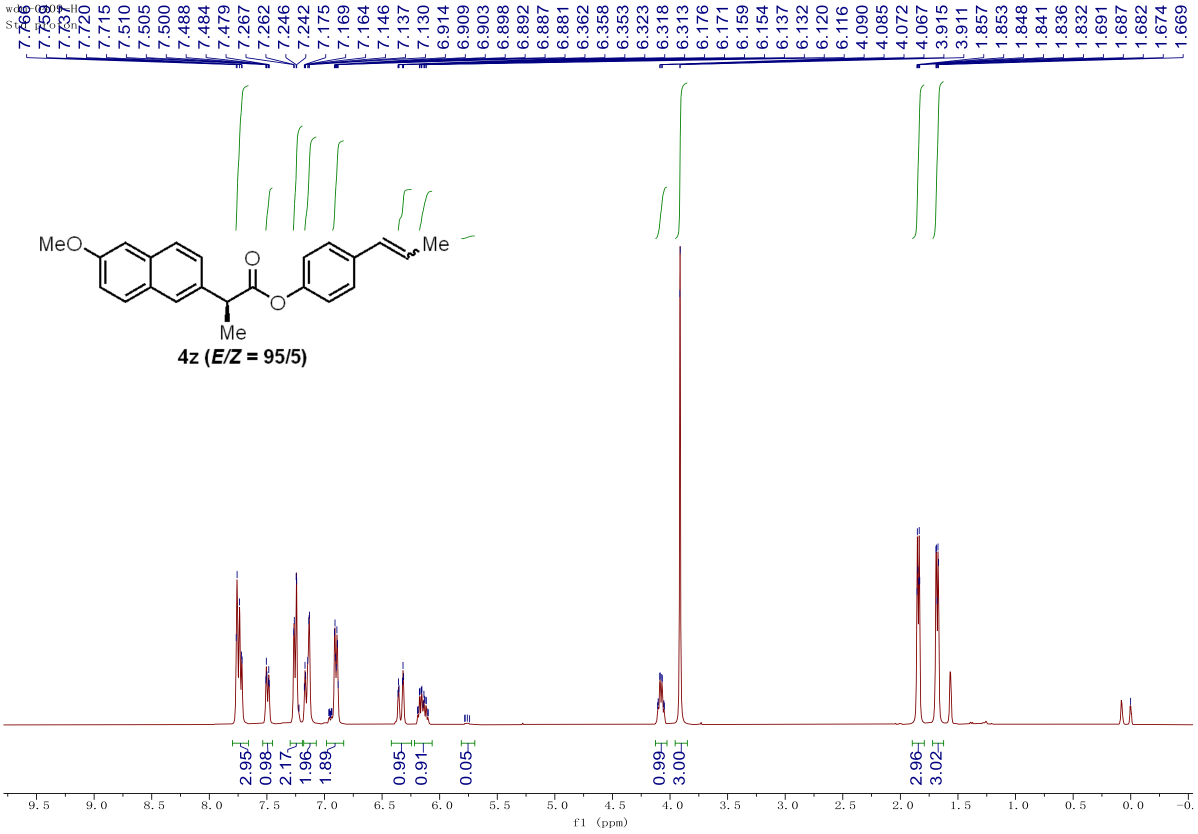

Supplementary Figure 42. ^{1}**H NMR (400 MHz, CDCl**$_3$**) spectrum of 4z**

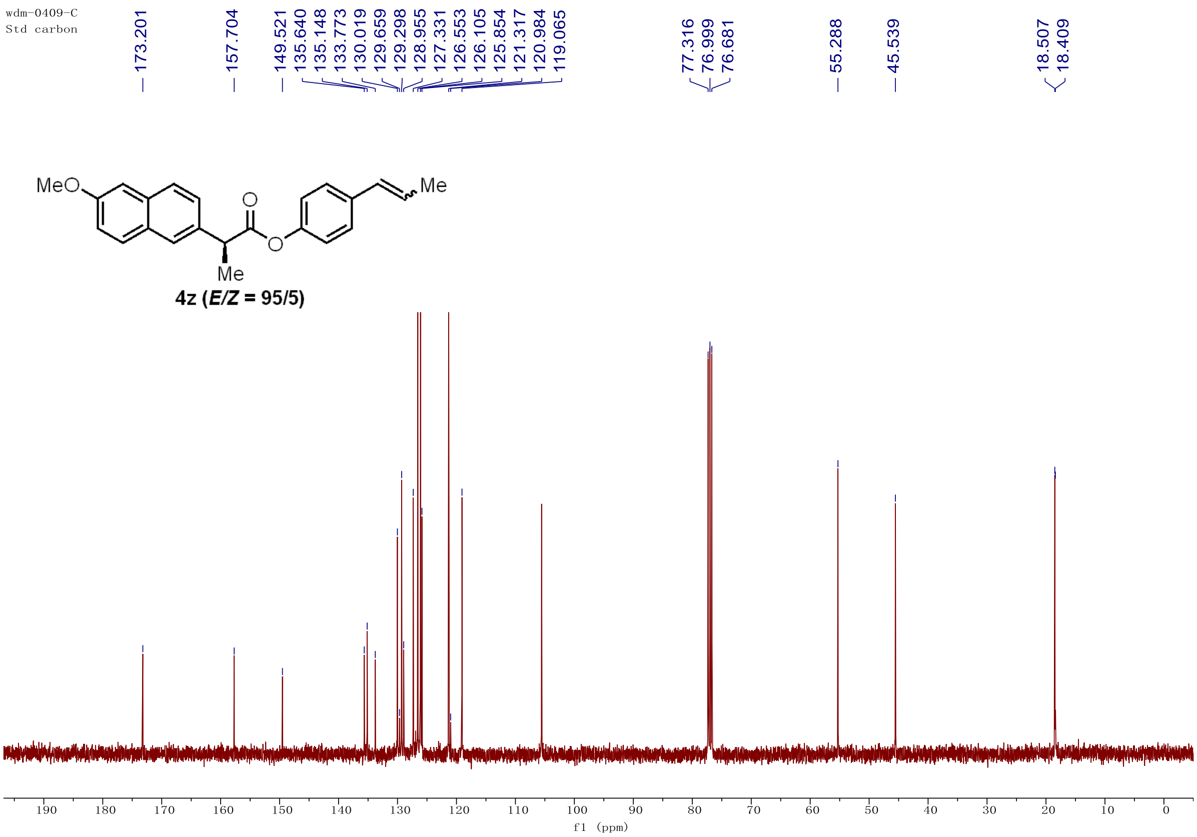

Supplementary Figure 43. ^{13}**C NMR (100 MHz, CDCl**$_3$**) spectrum of 4z**

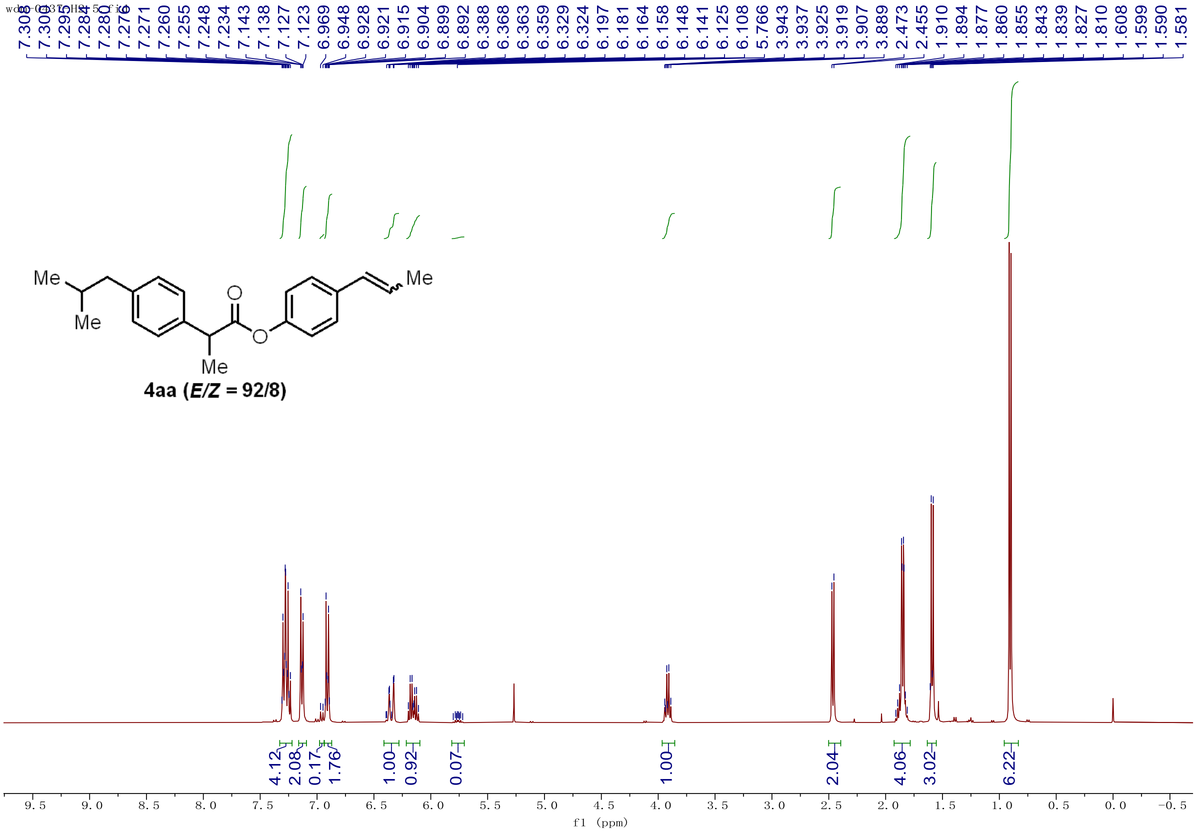

Supplementary Figure 44. ^{1}**H NMR (400 MHz, CDCl**$_3$**) spectrum of 4aa**

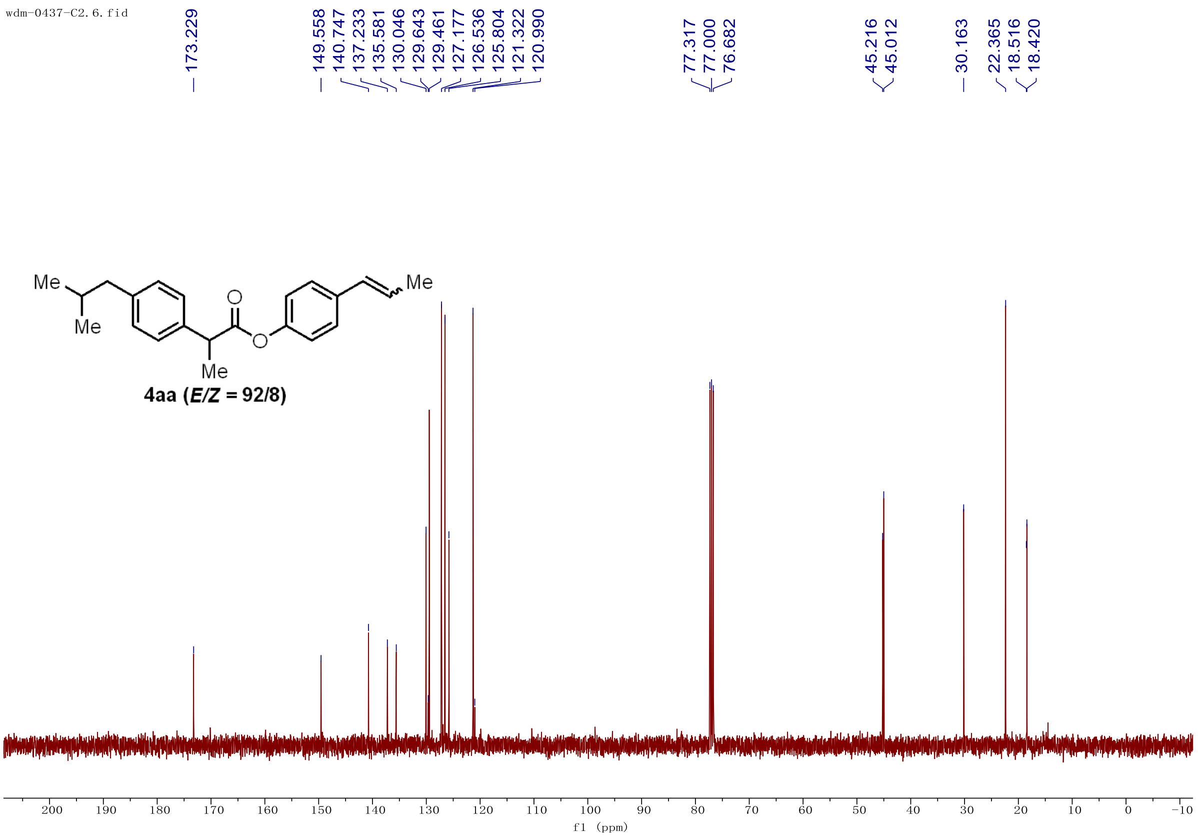

Supplementary Figure 45. ^{13}C NMR (100 MHz, $CDCl_3$) spectrum of 4aa

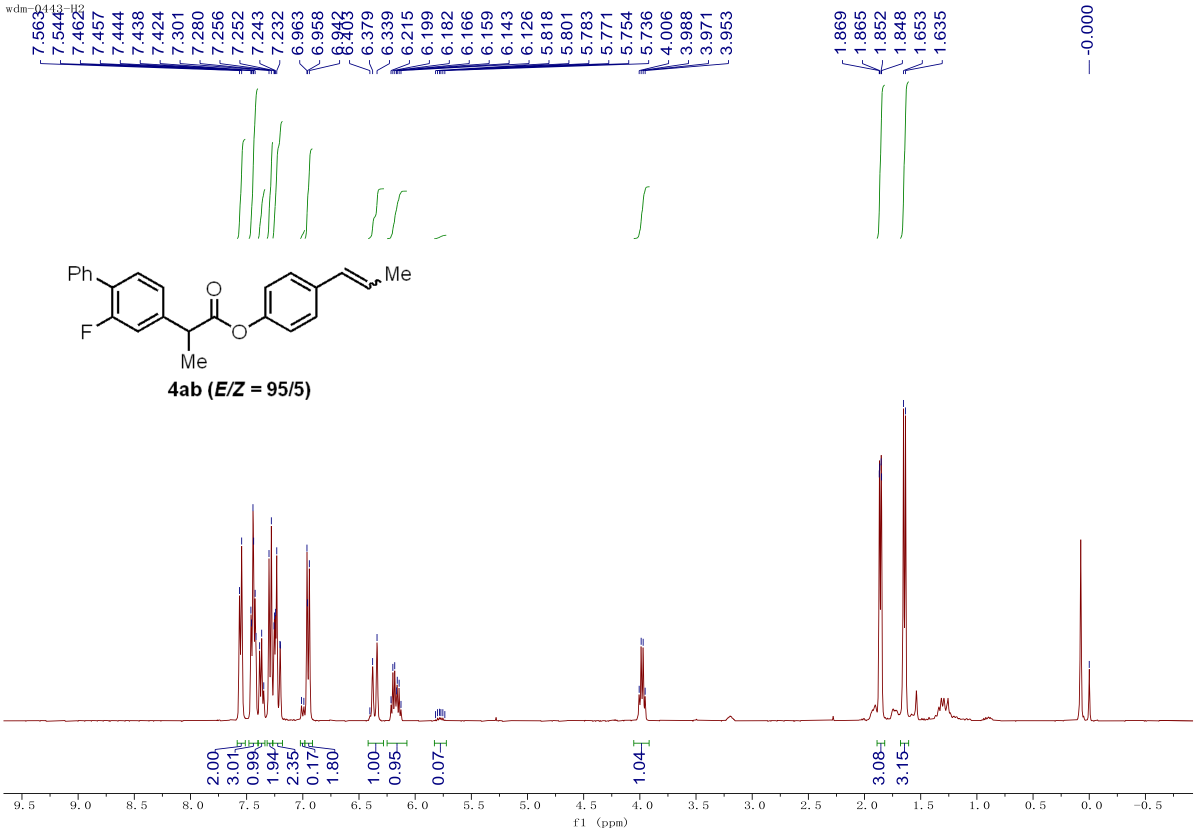

Supplementary Figure 46. ^{1}H NMR (400 MHz, $CDCl_3$) spectrum of 4ab

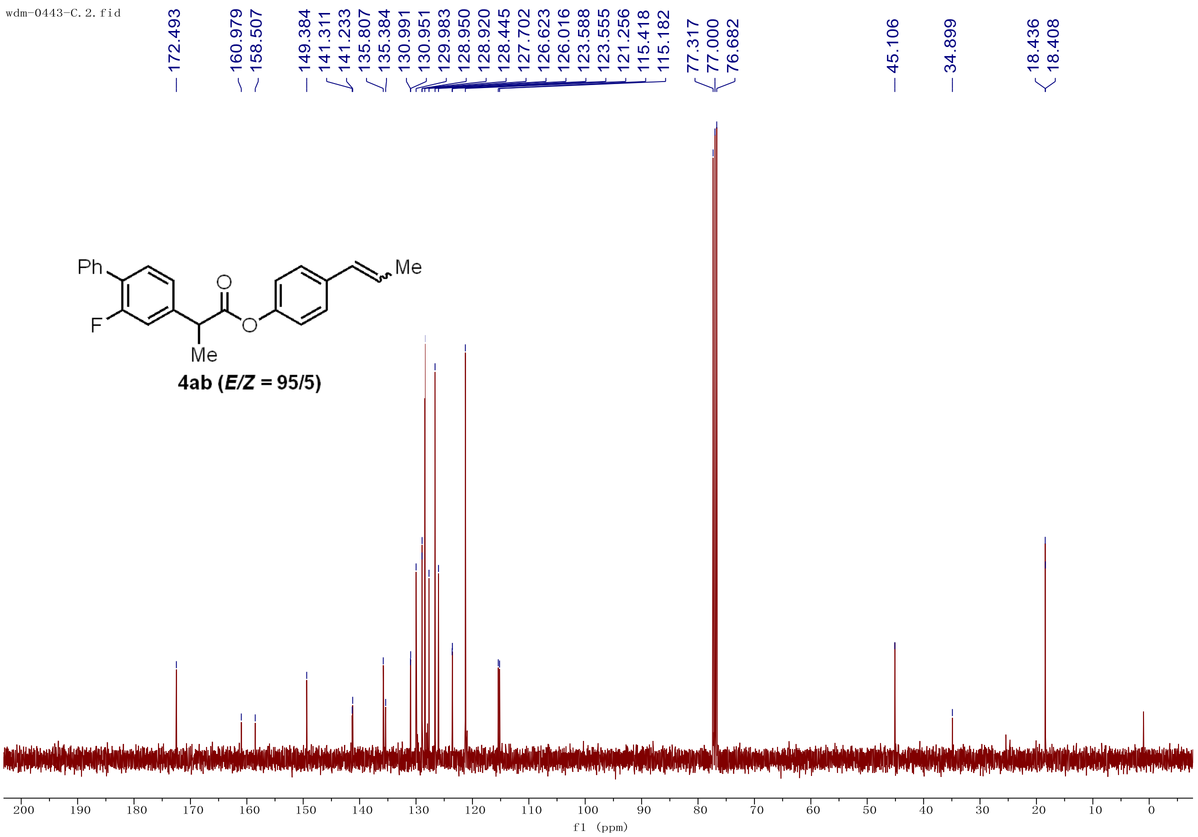

Supplementary Figure 47. ^{13}**C NMR (400 MHz,** $CDCl_3$**) spectrum of 4ab**

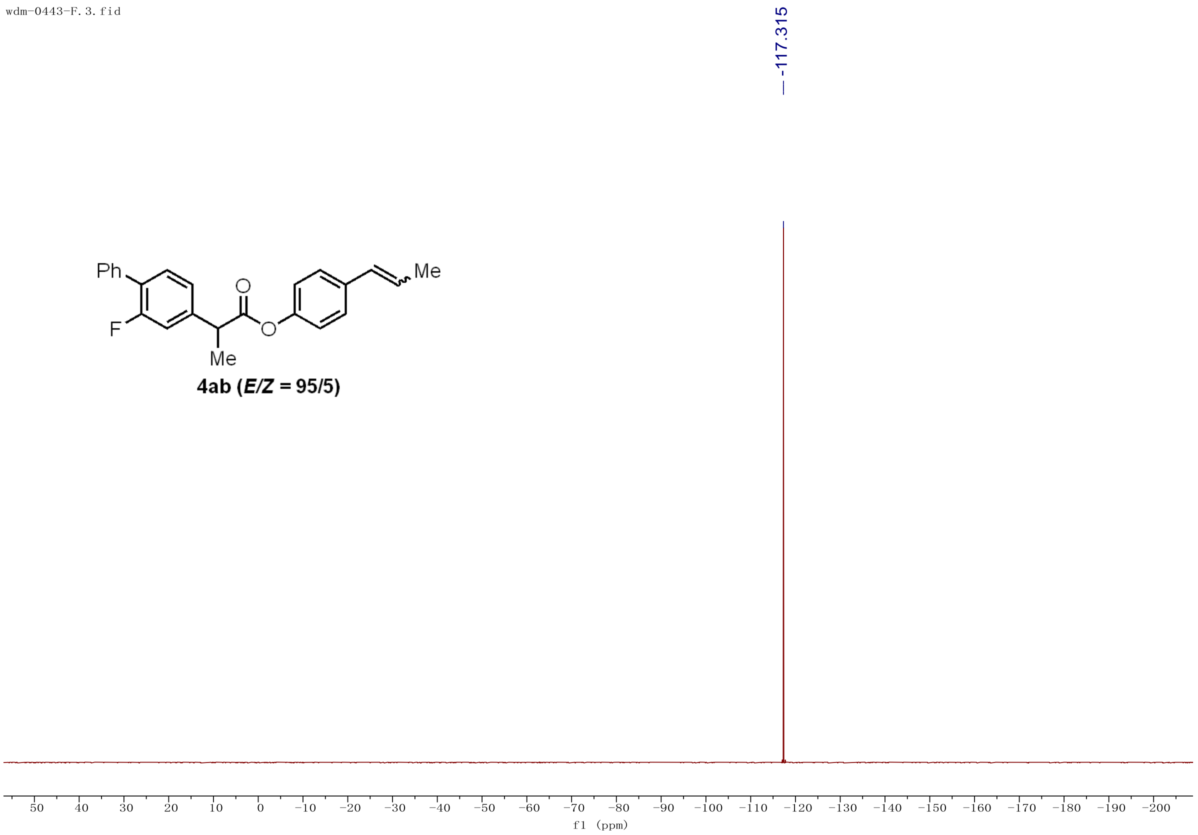

Supplementary Figure 48. ^{19}**F NMR (375 MHz,** $CDCl_3$**) spectrum of 4ab**

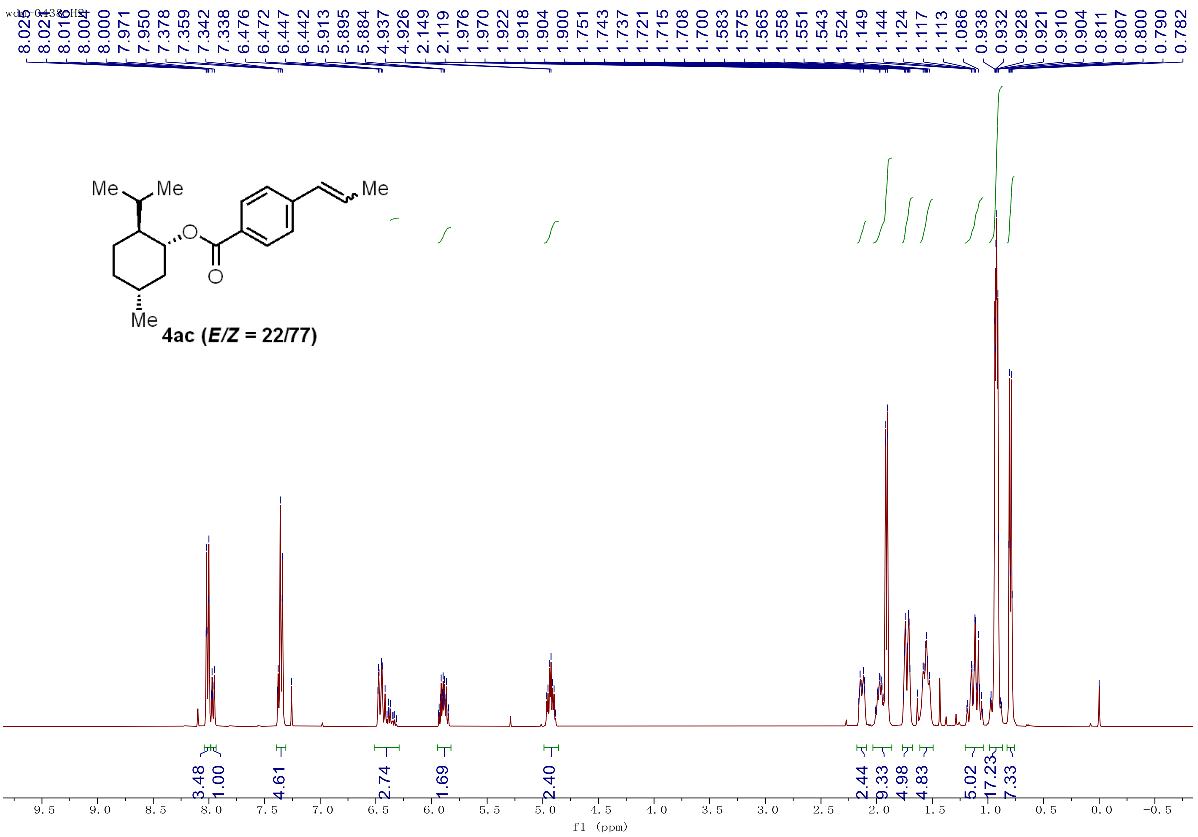

Supplementary Figure 49. ^{1}H **NMR (400 MHz, CDCl₃) spectrum of 4ac**

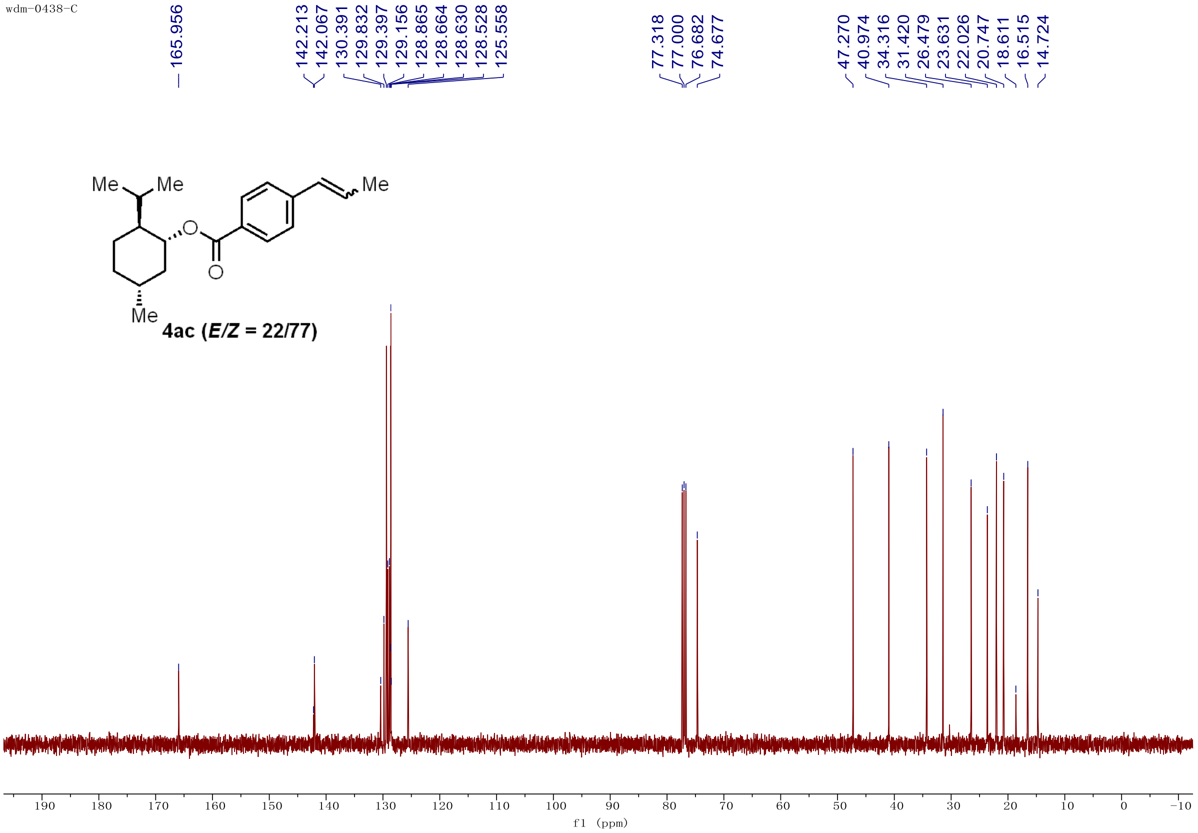

Supplementary Figure 50. ^{13}C **NMR (100 MHz, CDCl₃) spectrum of 4ac**

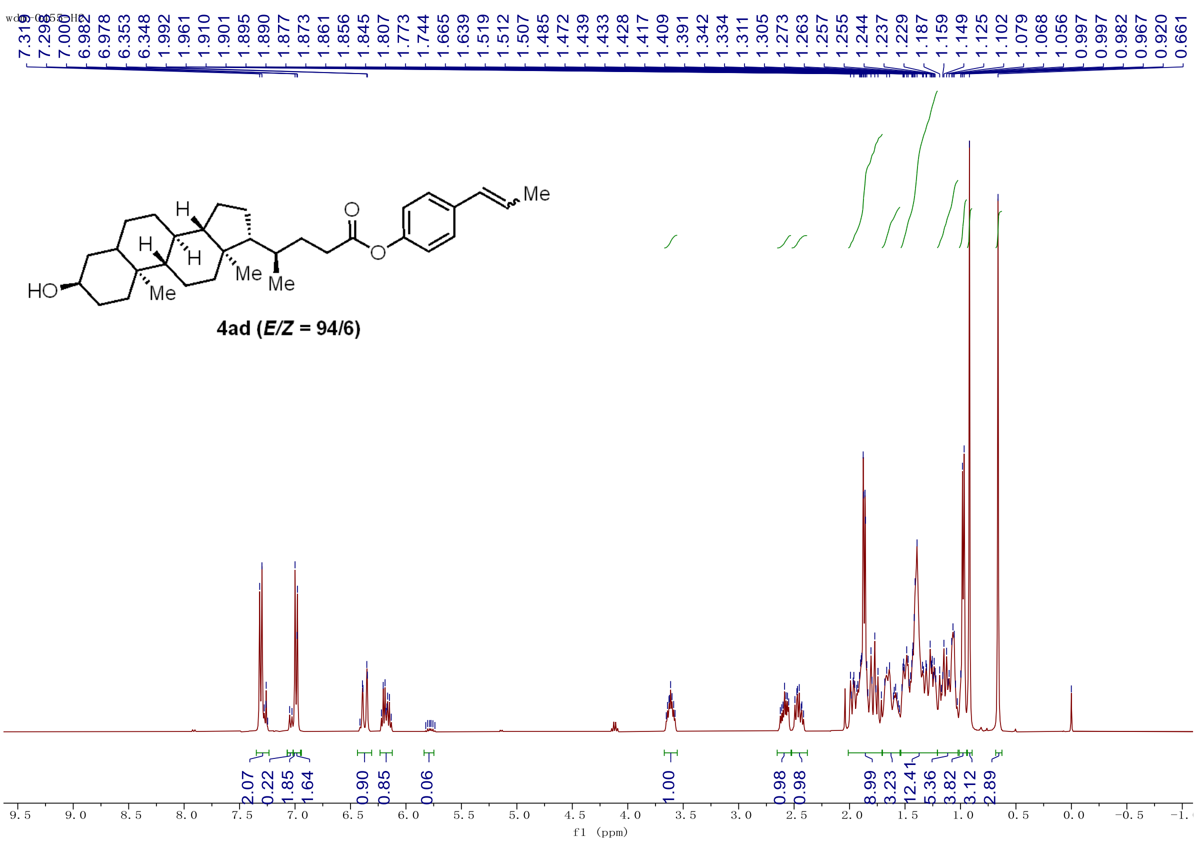

Supplementary Figure 51. ^{1}H NMR (400 MHz, CDCl$_3$) spectrum of 4ad

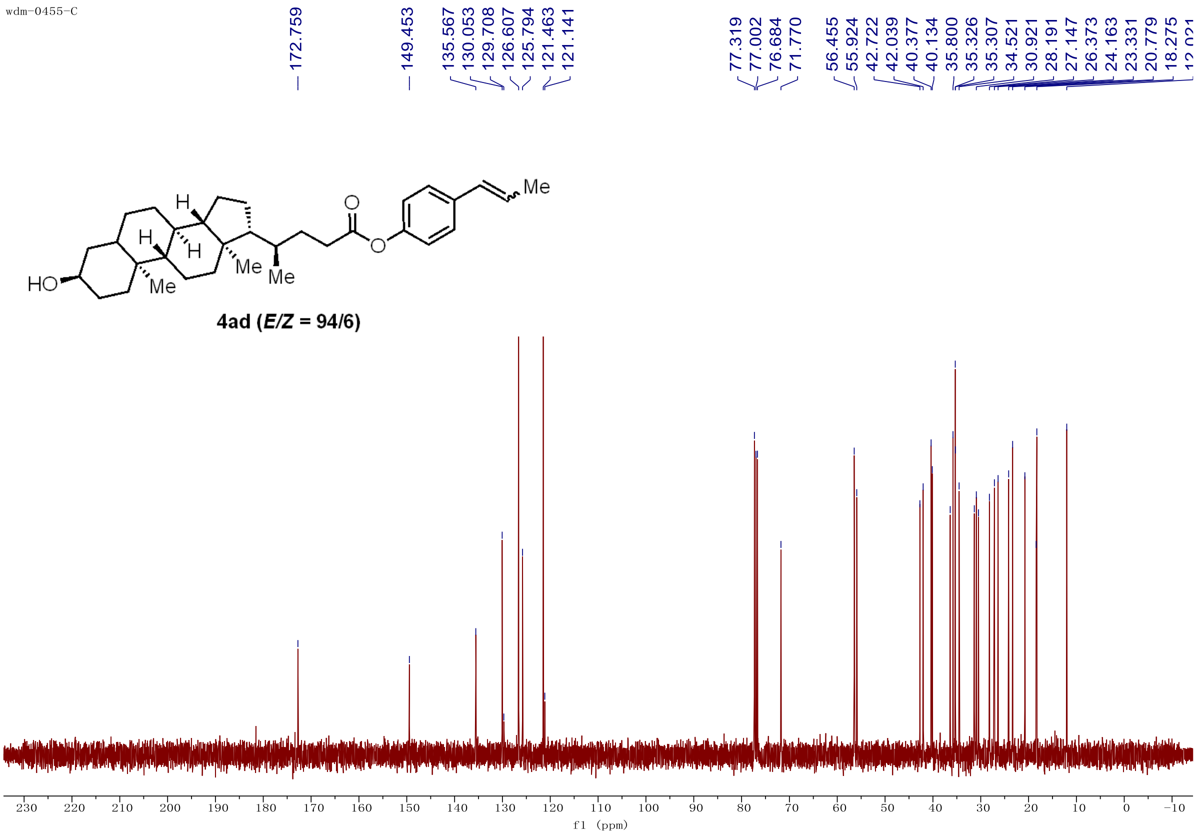

Supplementary Figure 52. ^{13}C NMR (100 MHz, CDCl$_3$) spectrum of 4ad

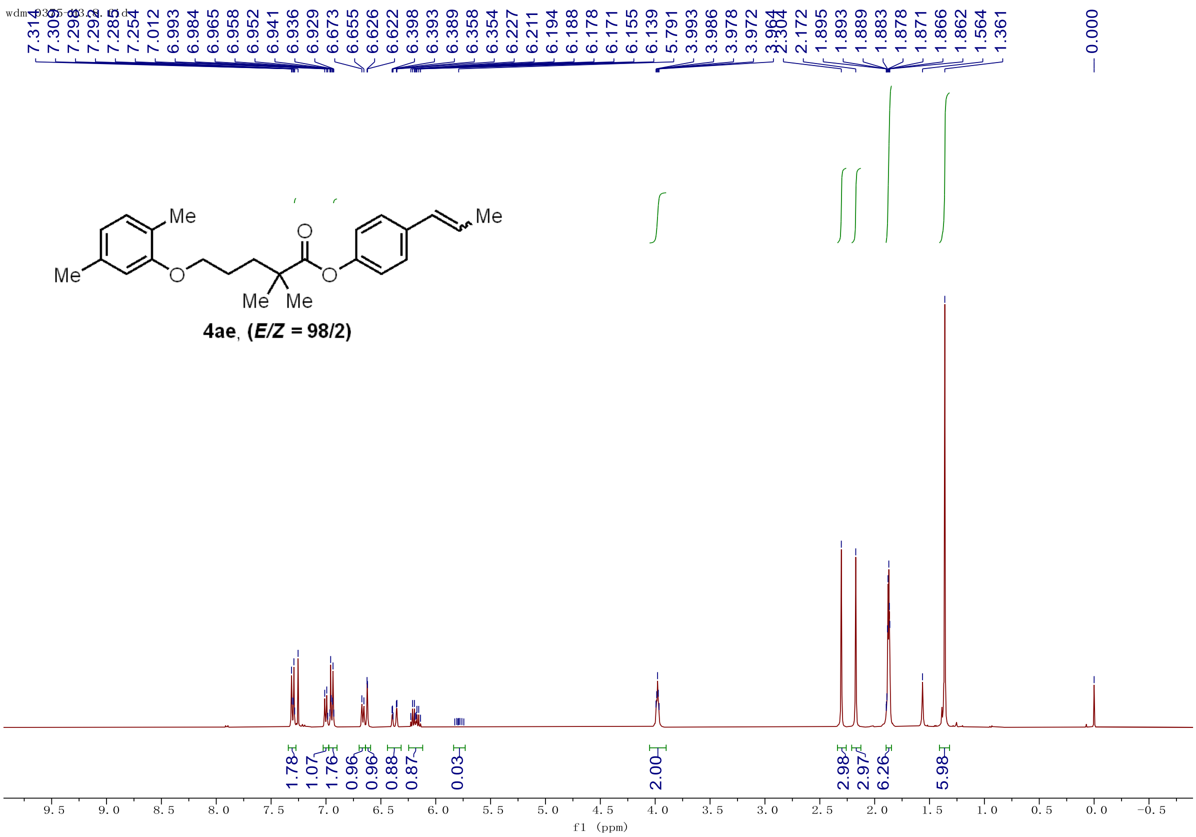

Supplementary Figure 53. ^{1}**H NMR (400 MHz, CDCl**$_3$**) spectrum of 4ae**

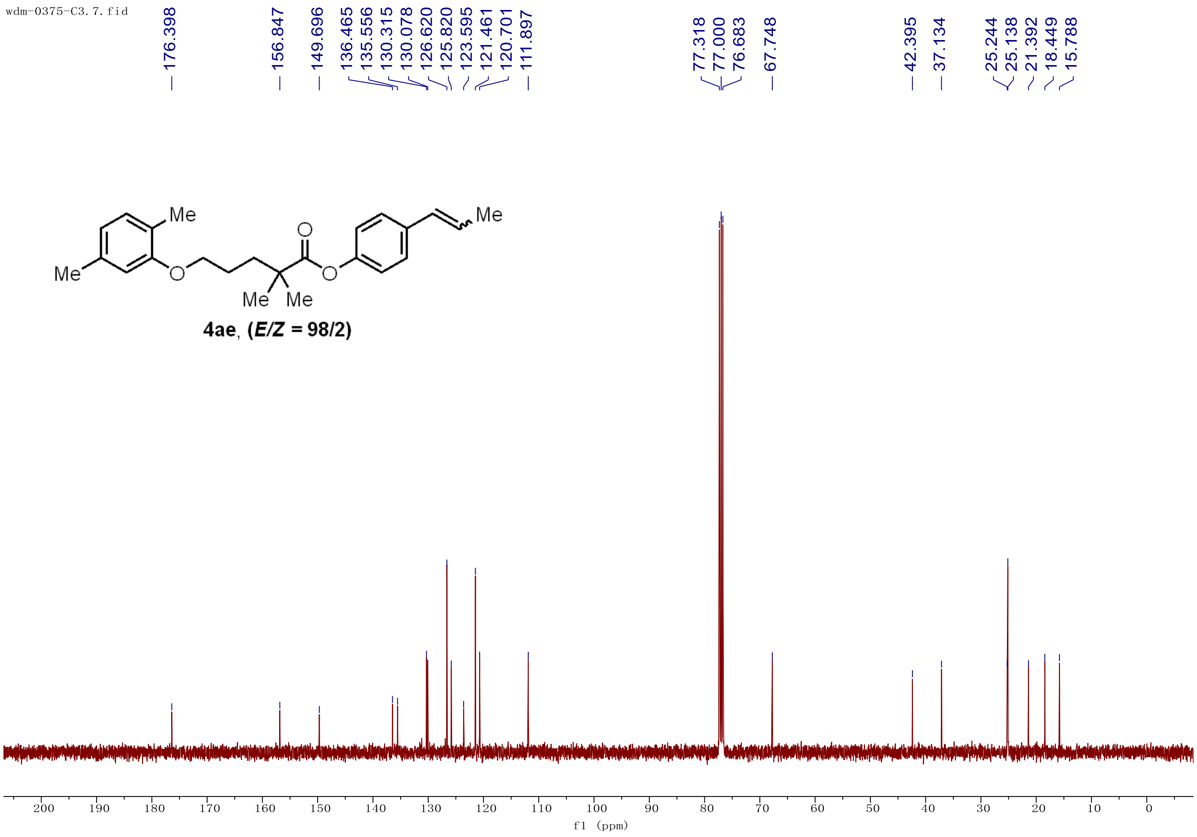

Supplementary Figure 54. ^{13}**C NMR (100 MHz, CDCl**$_3$**) spectrum of 4ae**

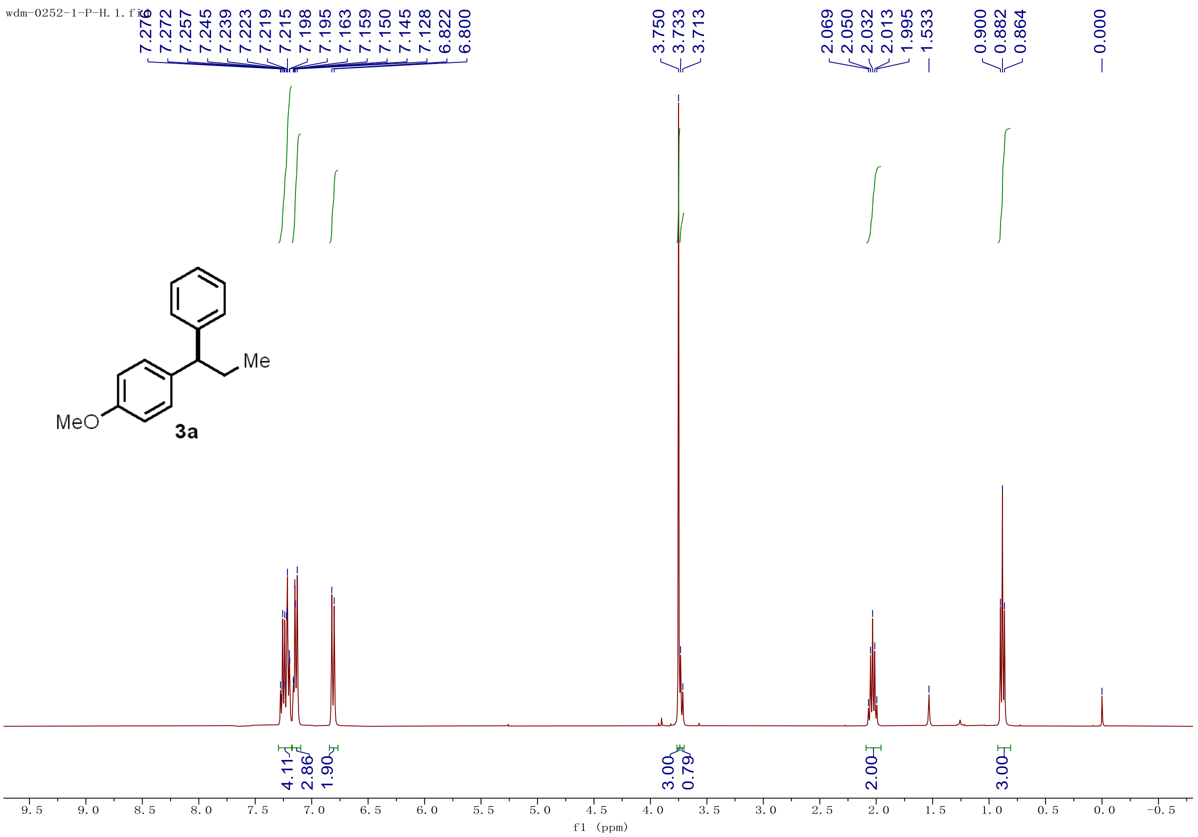

Supplementary Figure 55. ^{1}H **NMR (400 MHz,** $CDCl_3$**) spectrum of 3a**

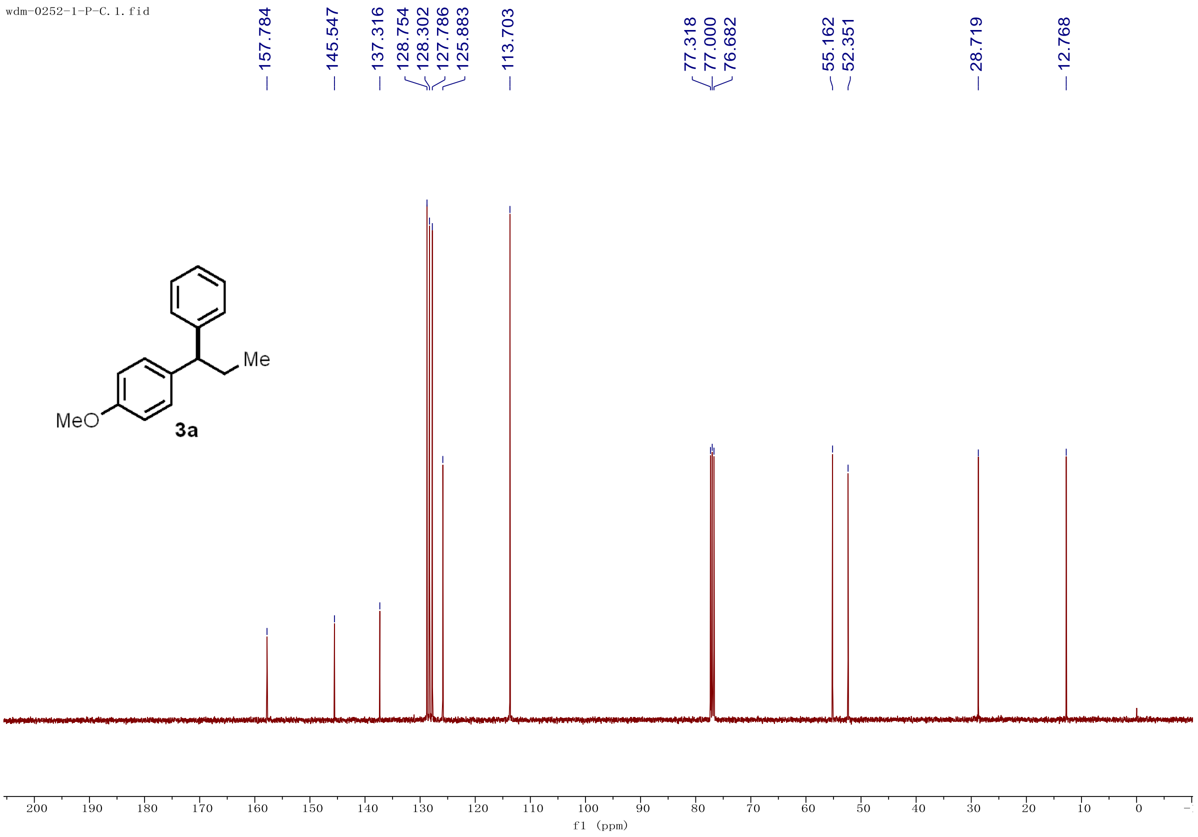

Supplementary Figure 56. ^{13}C **NMR (100 MHz,** $CDCl_3$**) spectrum of 3a**

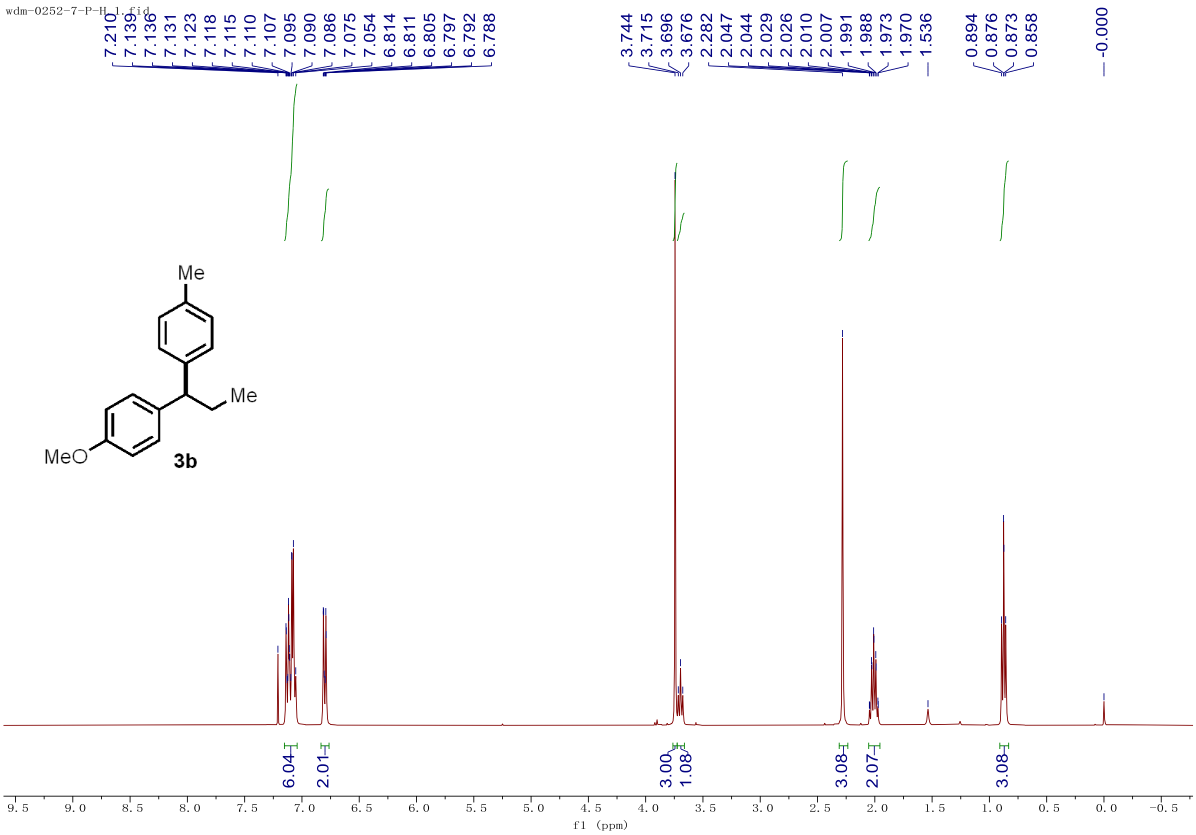

Supplementary Figure 57. ^{1}H NMR (400 MHz, $CDCl_3$) spectrum of 3b

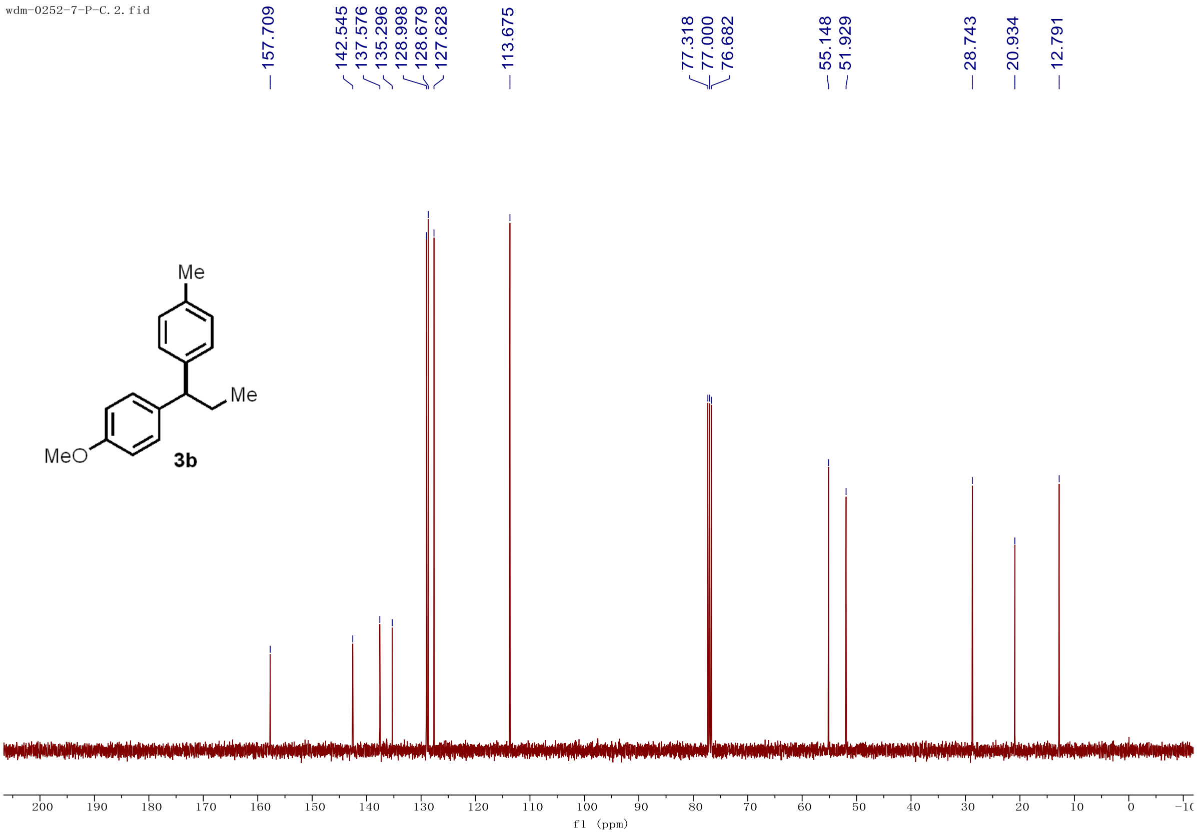

Supplementary Figure 58. ^{13}C NMR (100 MHz, $CDCl_3$) spectrum of 3b

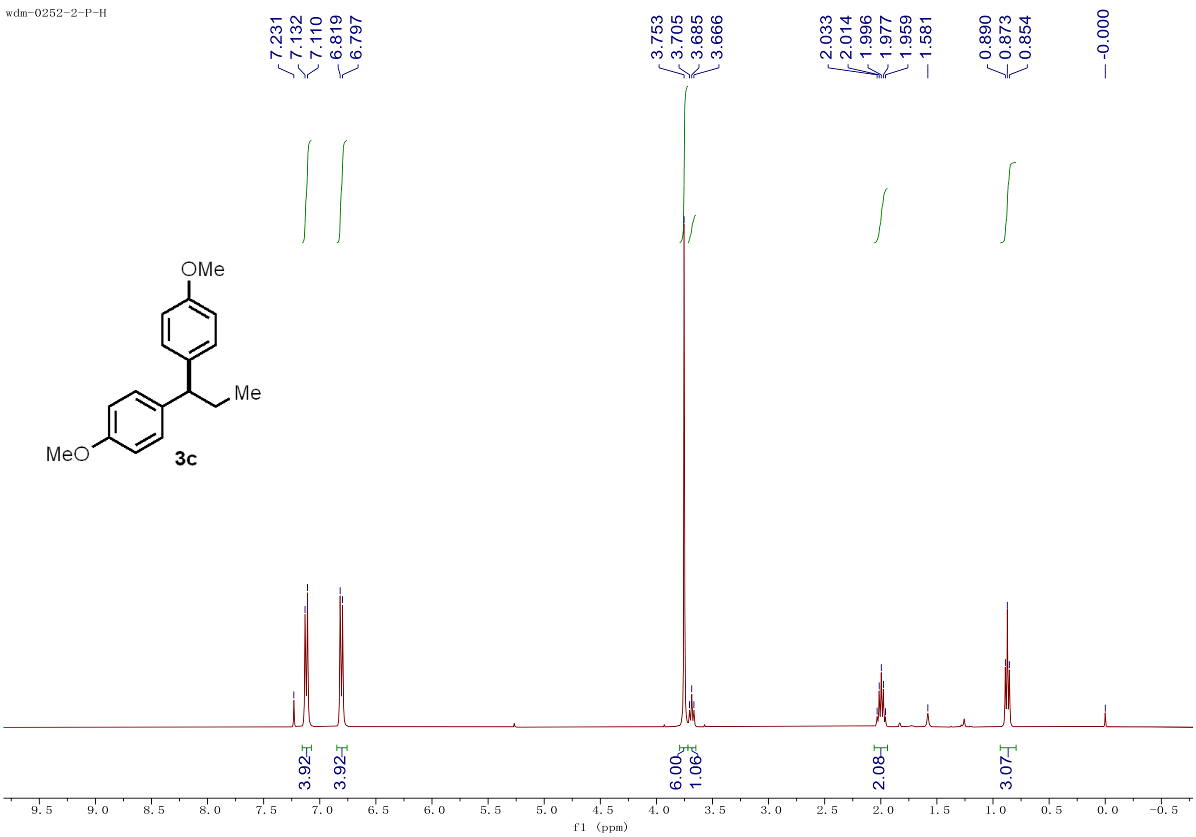

Supplementary Figure 59. ¹H NMR (400 MHz, CDCl₃) spectrum of 3c

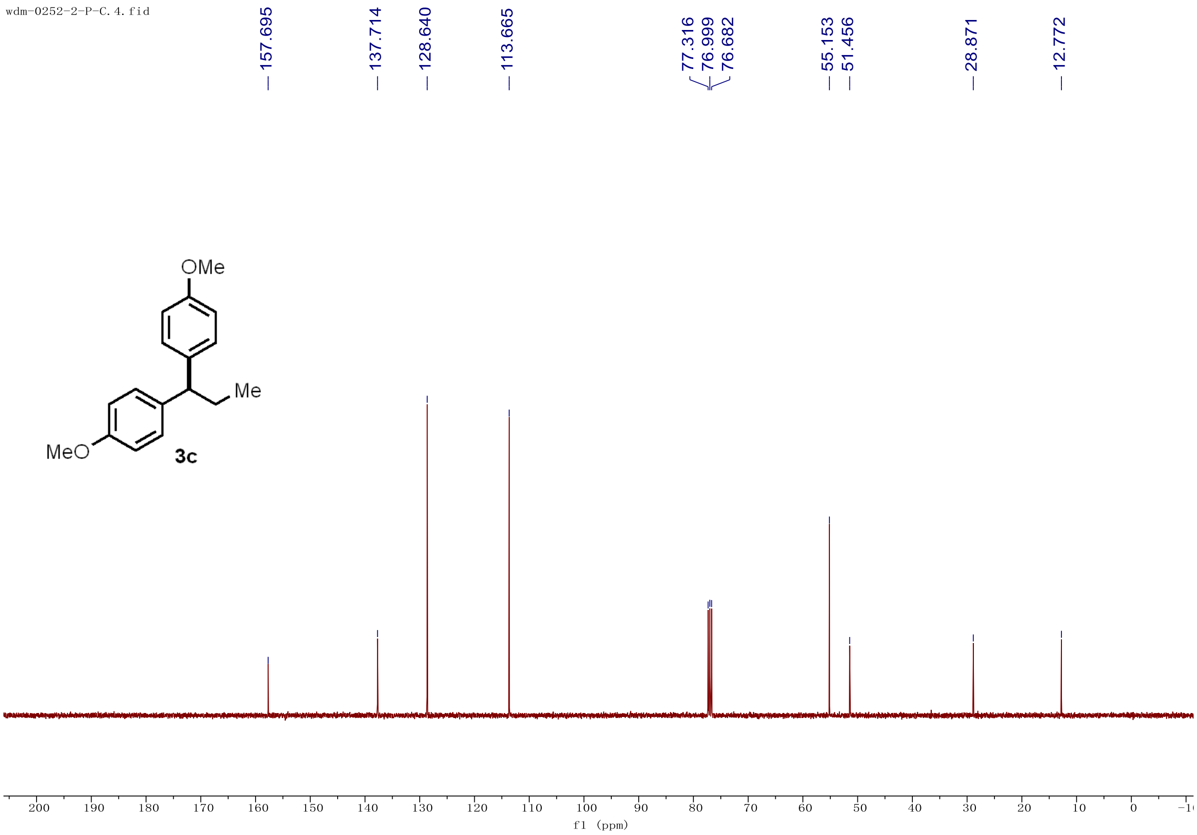

Supplementary Figure 60. ¹³C NMR (100 MHz, CDCl₃) spectrum of 3c

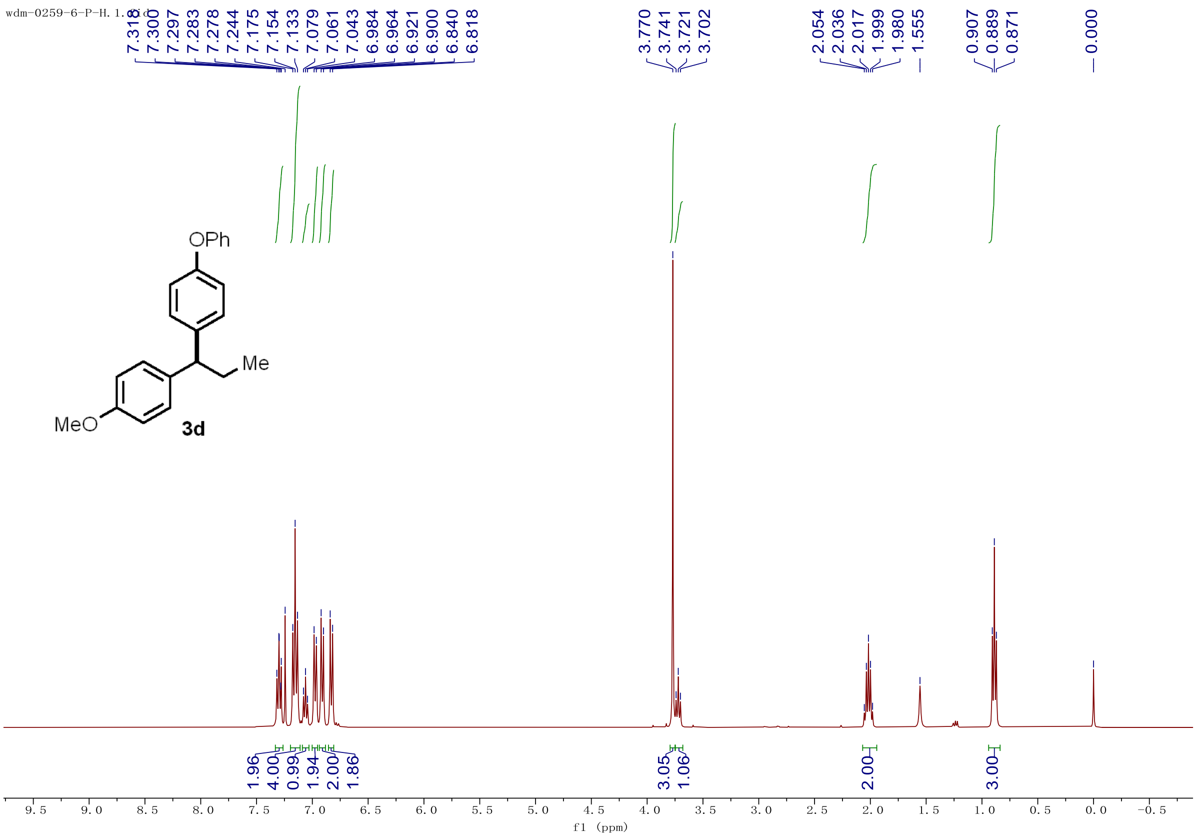

Supplementary Figure 61. ^{1}H NMR (400 MHz, $CDCl_3$) spectrum of 3d

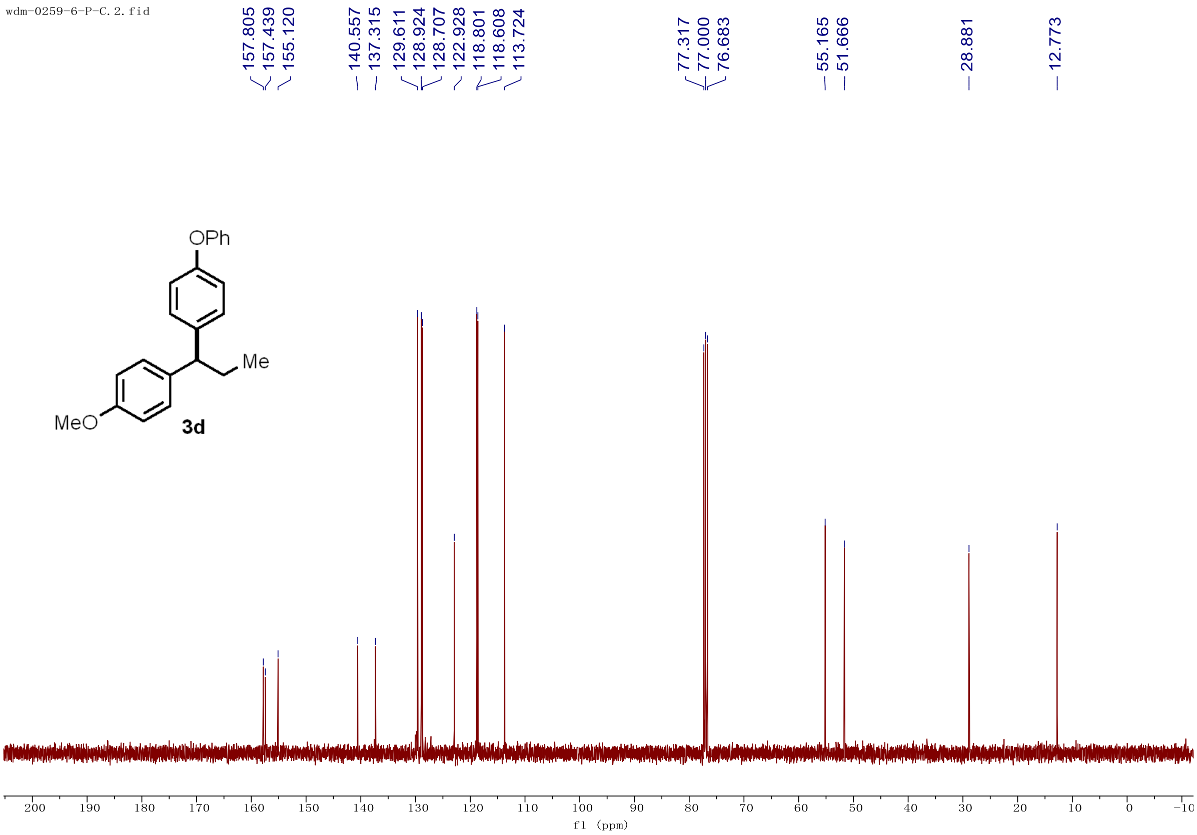

Supplementary Figure 62. ^{13}C NMR (100 MHz, $CDCl_3$) spectrum of 3d

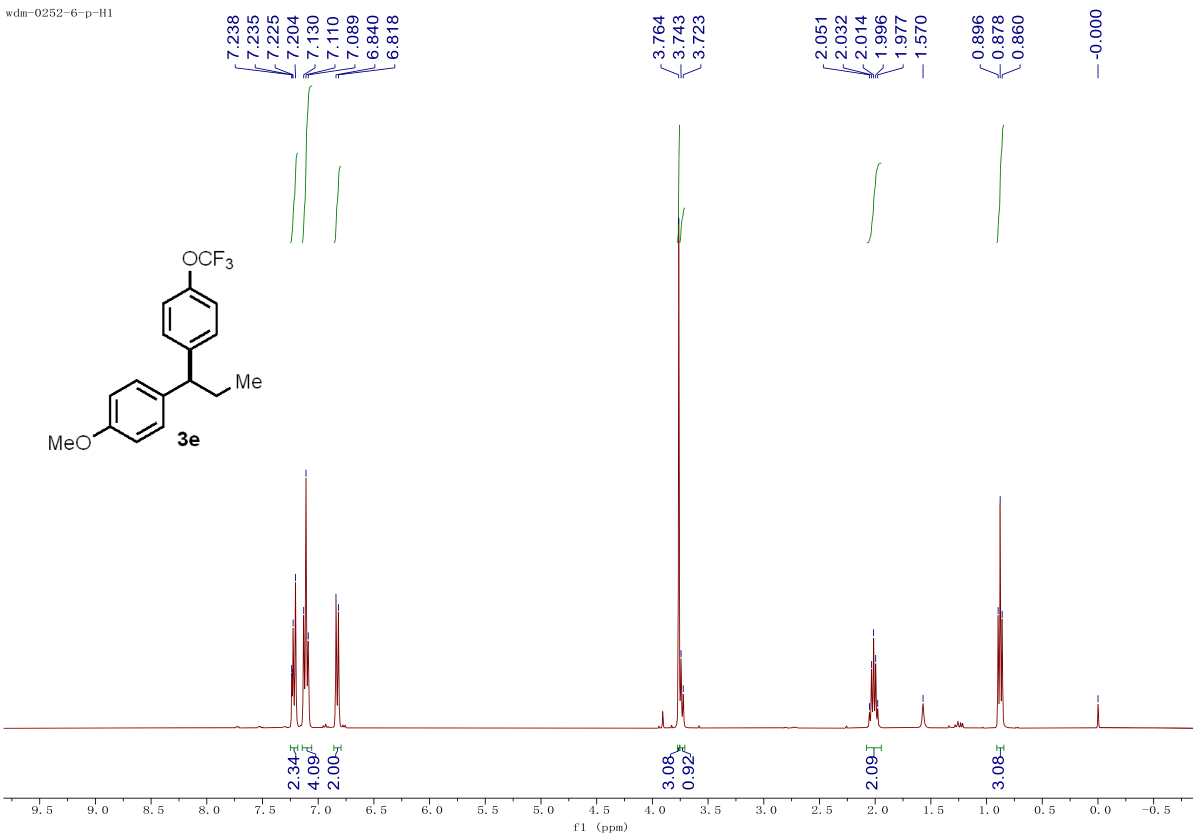

Supplementary Figure 63. ^{1}**H NMR (400 MHz, CDCl**$_3$**) spectrum of 3e**

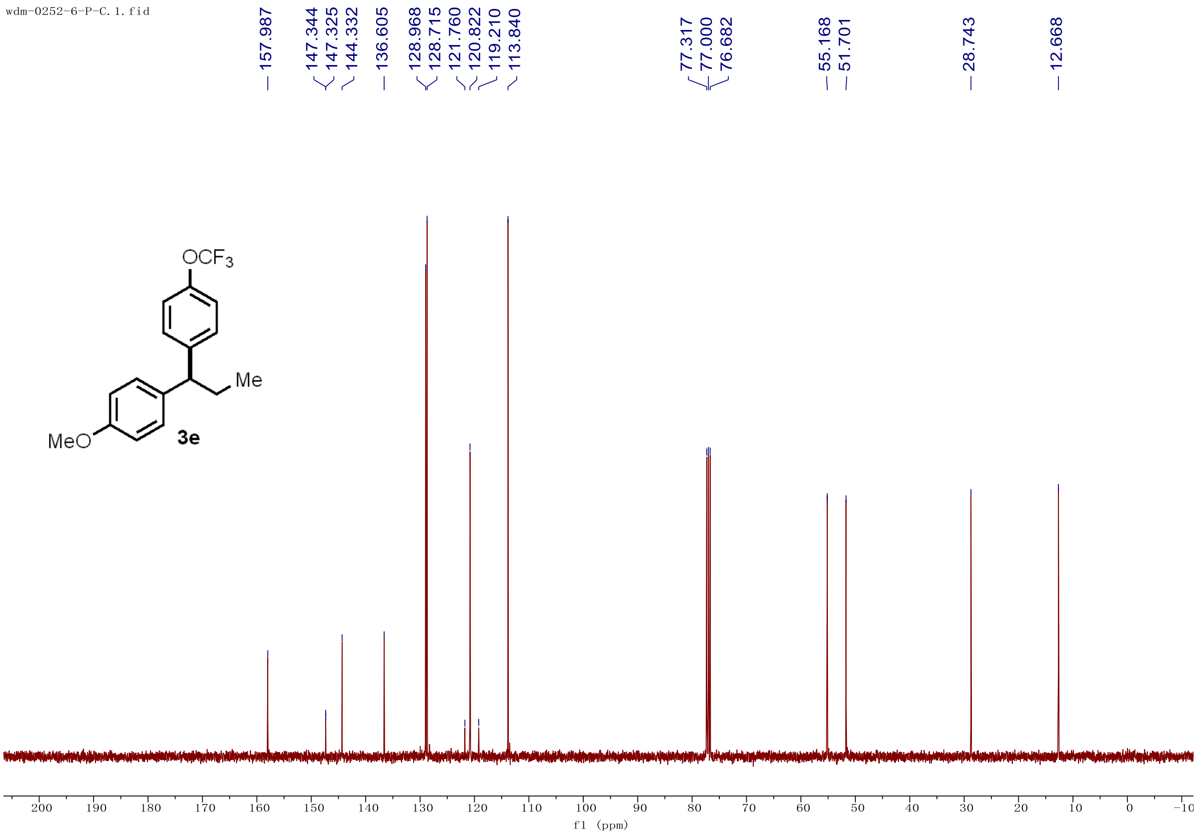

Supplementary Figure 64. ^{13}**C NMR (100 MHz, CDCl**$_3$**) spectrum of 3e**

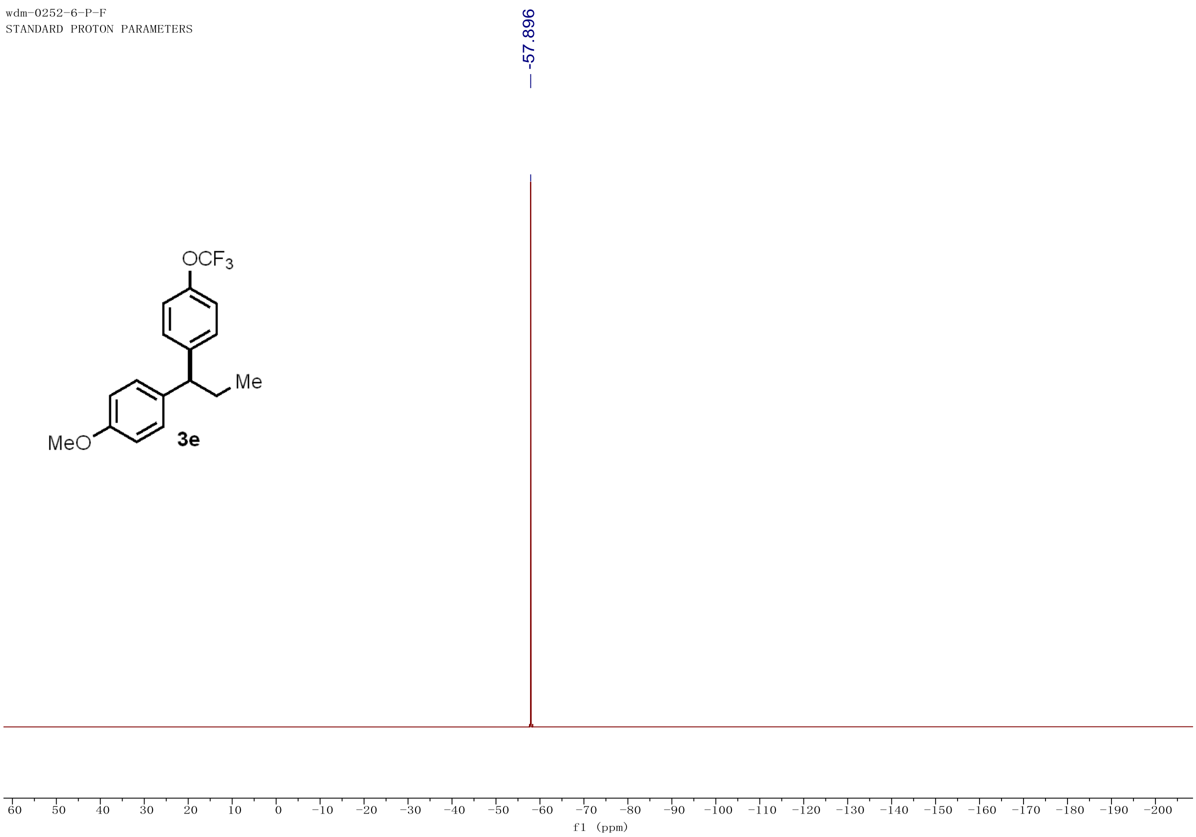

Supplementary Figure 65. ^{19}F NMR (375 MHz, CDCl$_3$) spectrum of 3e

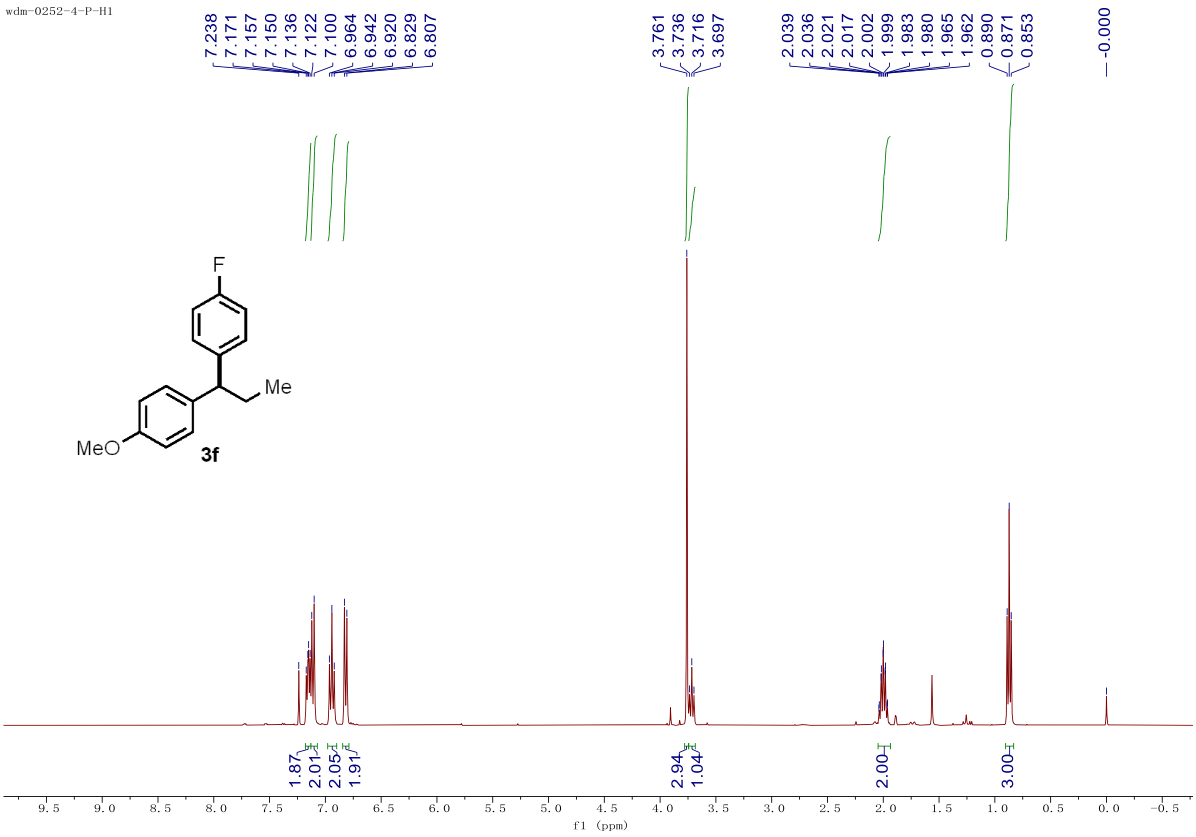

Supplementary Figure 66. ^{1}H NMR (400 MHz, CDCl$_3$) spectrum of 3f

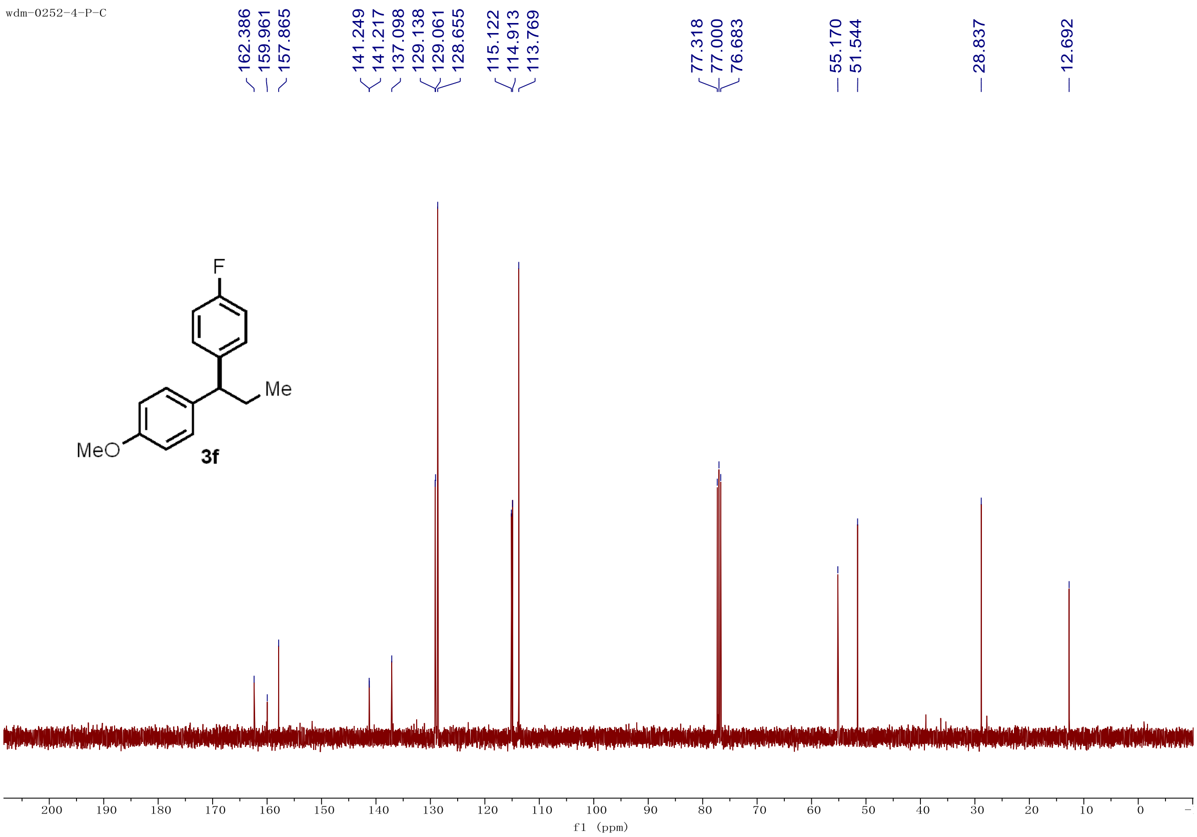

Supplementary Figure 67. ^{13}C NMR (100 MHz, CDCl$_3$) spectrum of 3f

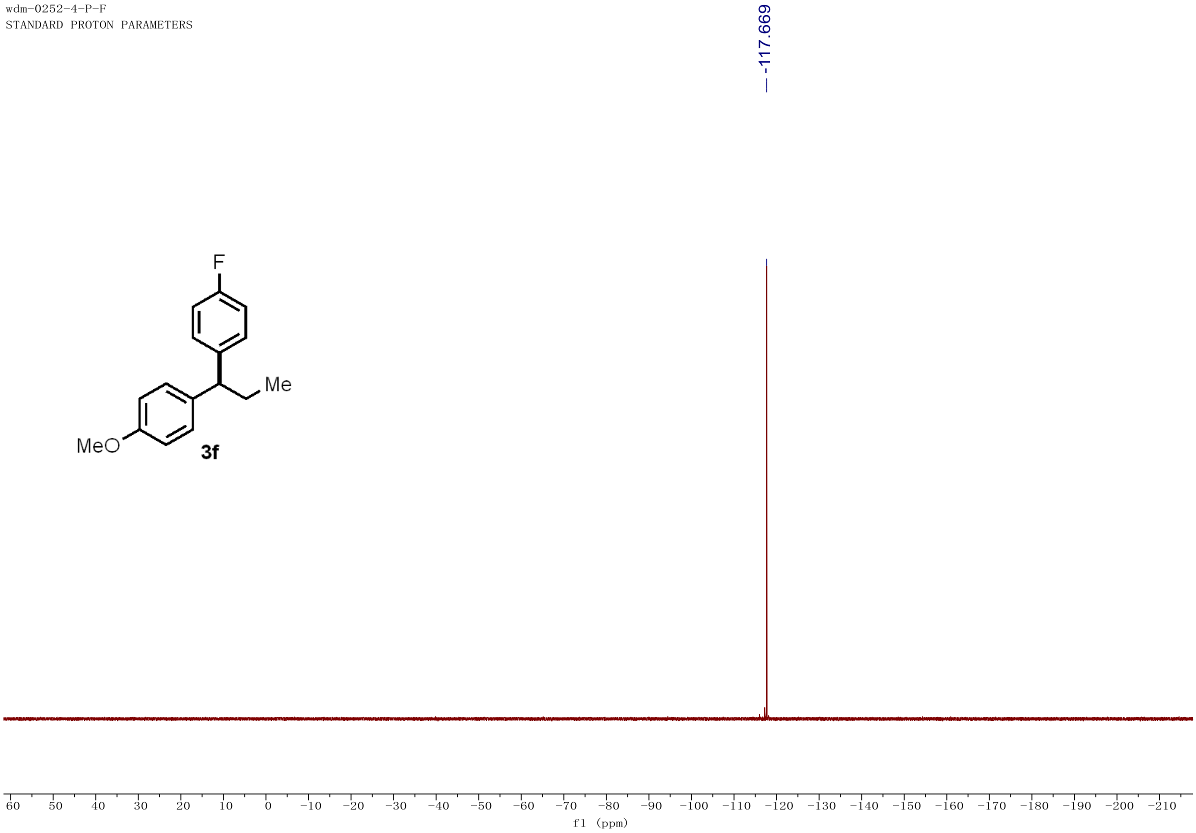

Supplementary Figure 68. ^{19}F NMR (375 MHz, CDCl$_3$) spectrum of 3f

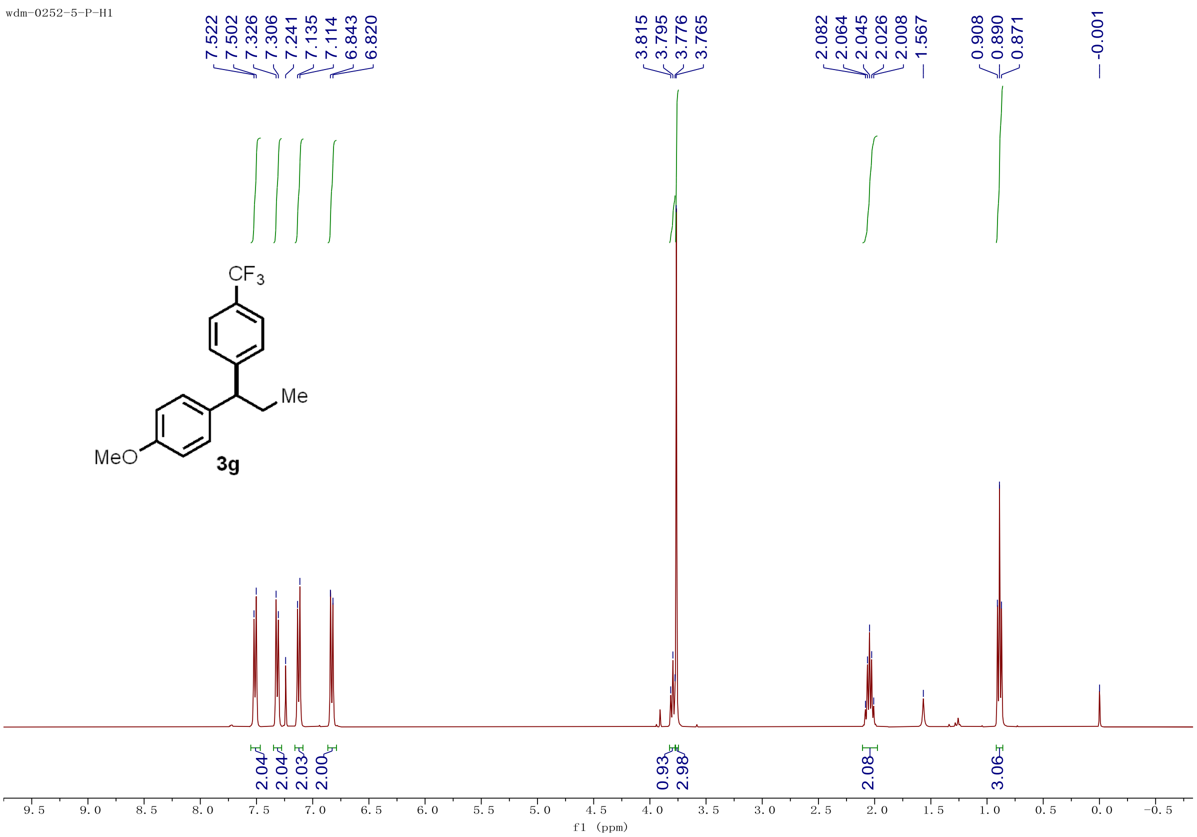

Supplementary Figure 69. ^{1}H NMR (400 MHz, $CDCl_3$) spectrum of 3g

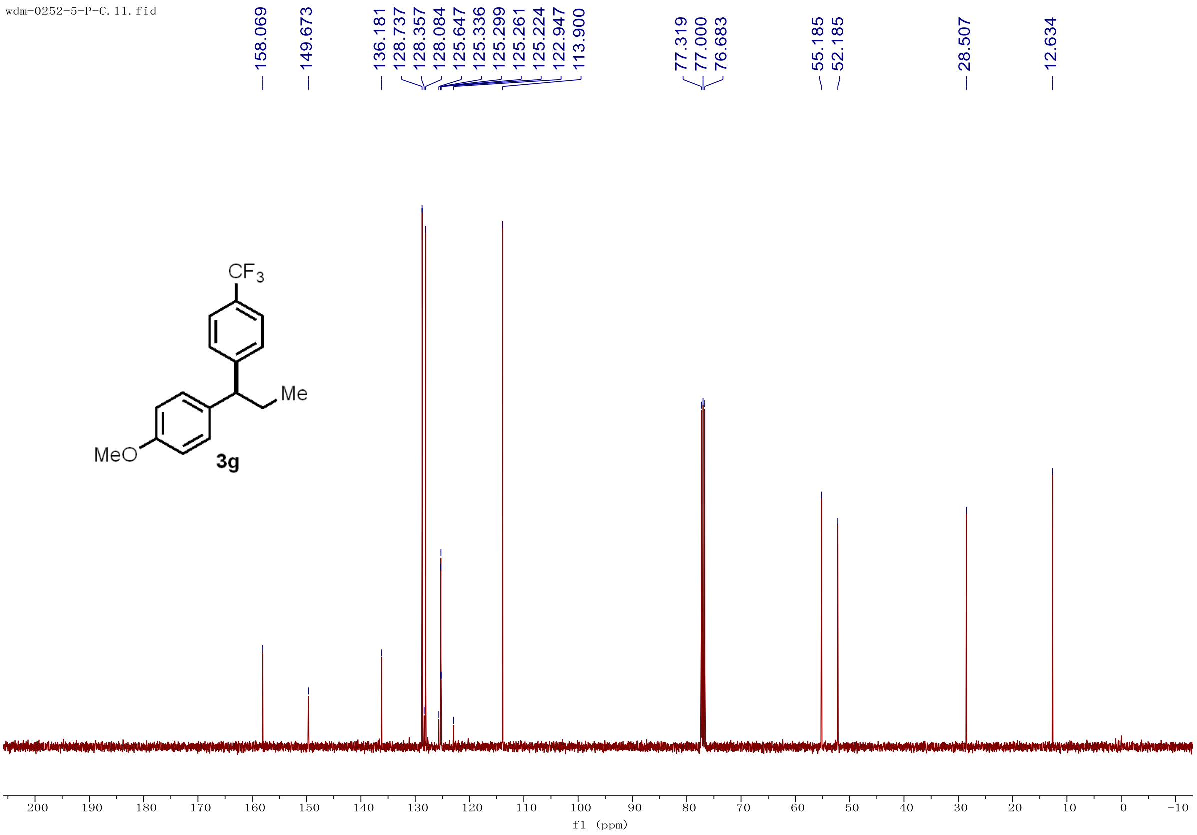

Supplementary Figure 70. ^{13}C NMR (100 MHz, $CDCl_3$) spectrum of 3g

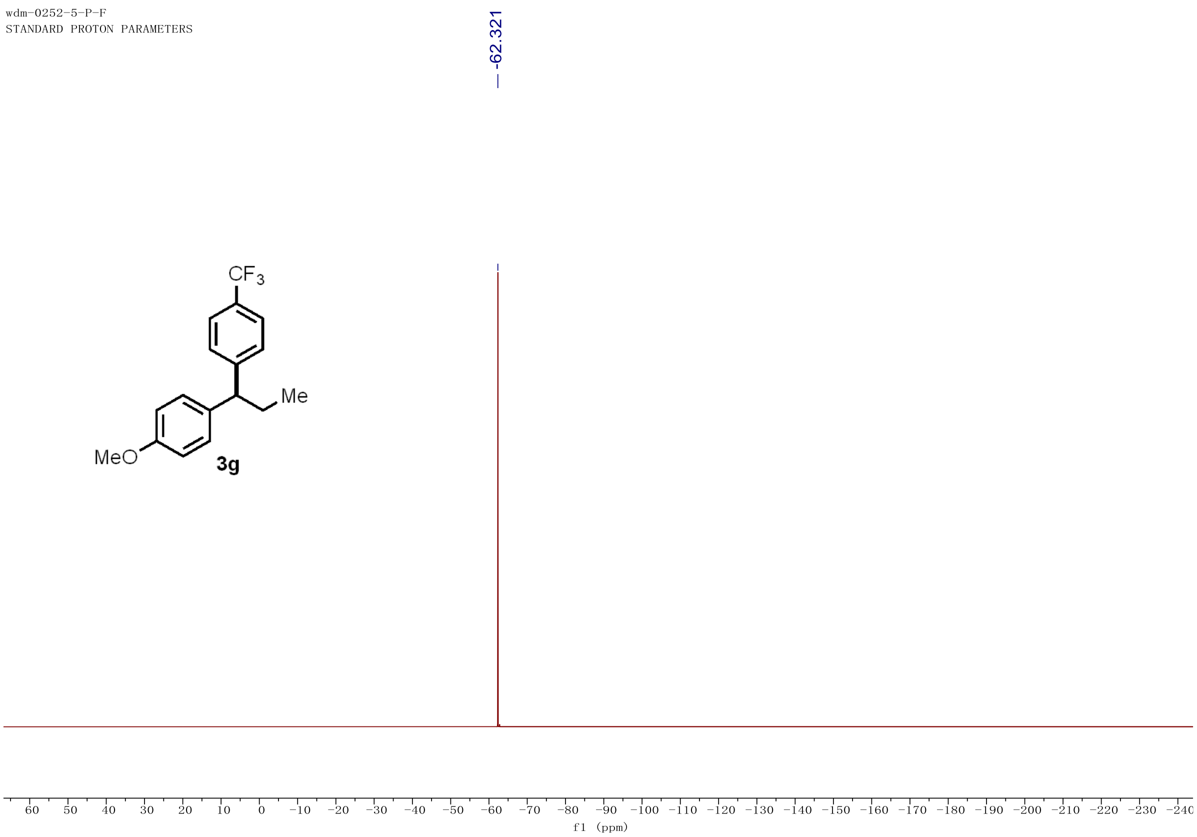

Supplementary Figure 71. ^{19}F NMR (375 MHz, CDCl$_3$) spectrum of 3g

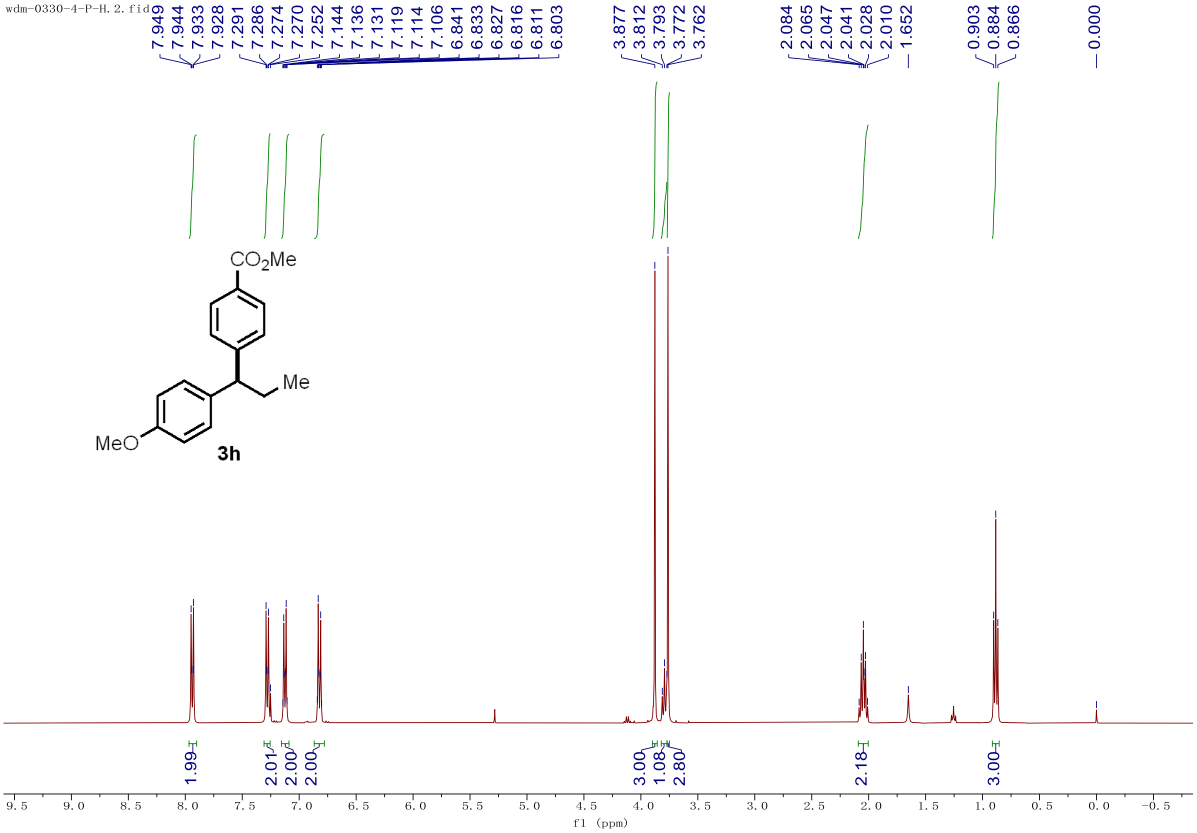

Supplementary Figure 72. ^{1}H NMR (400 MHz, CDCl$_3$) spectrum of 3h

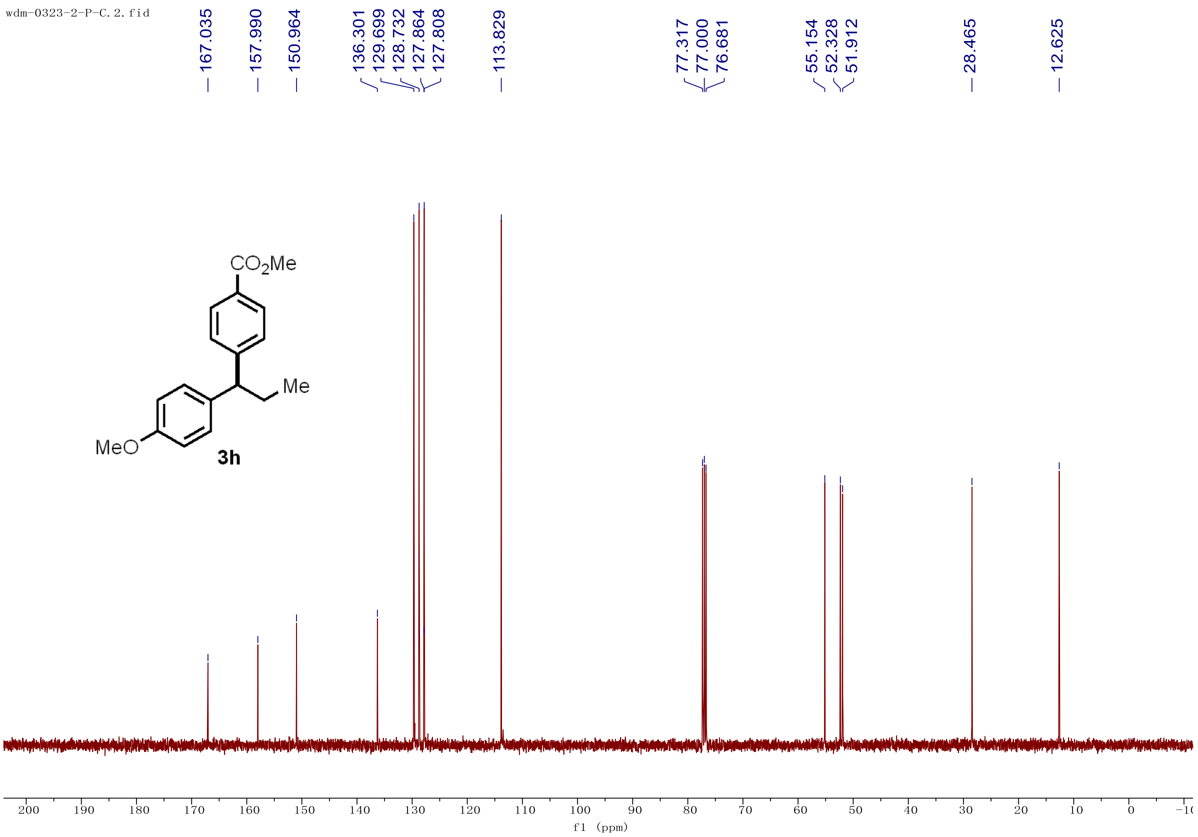

Supplementary Figure 73. ^{13}C NMR (100 MHz, CDCl$_3$) spectrum of 3h

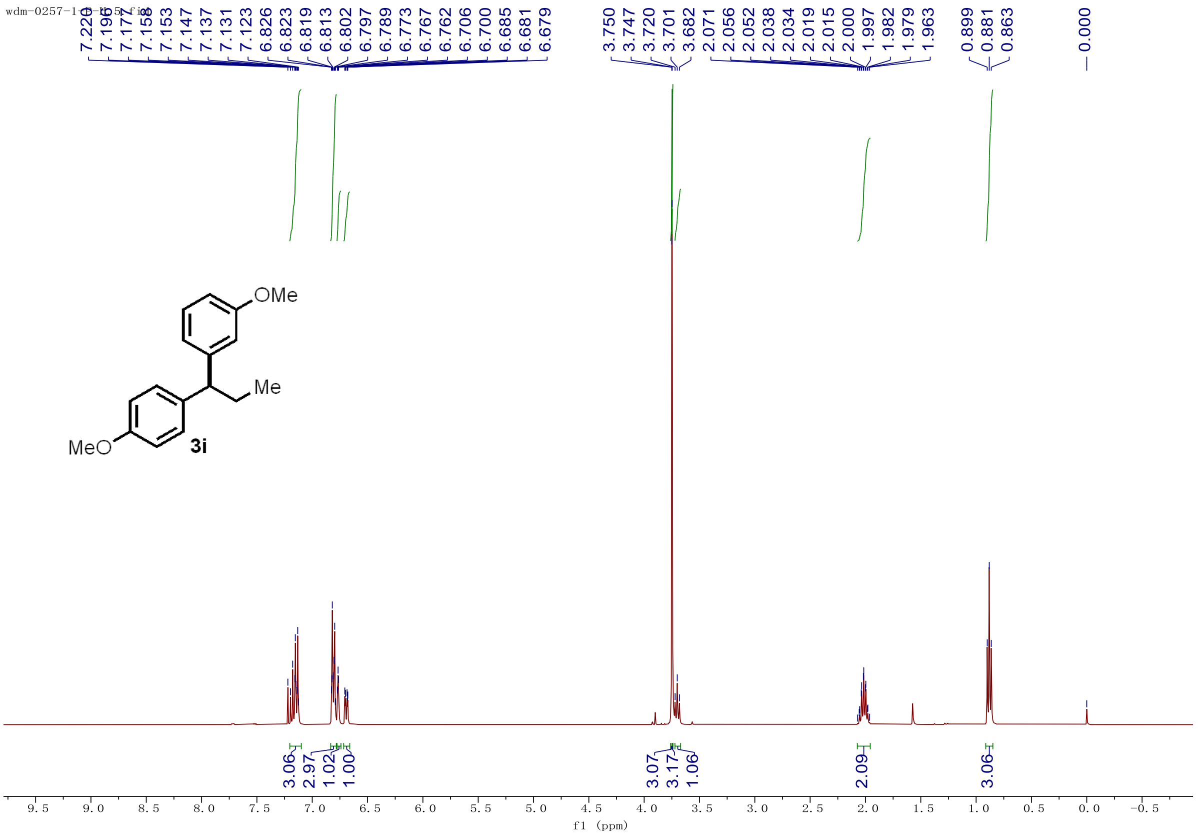

Supplementary Figure 74. ^{1}H NMR (400 MHz, CDCl$_3$) spectrum of 3i

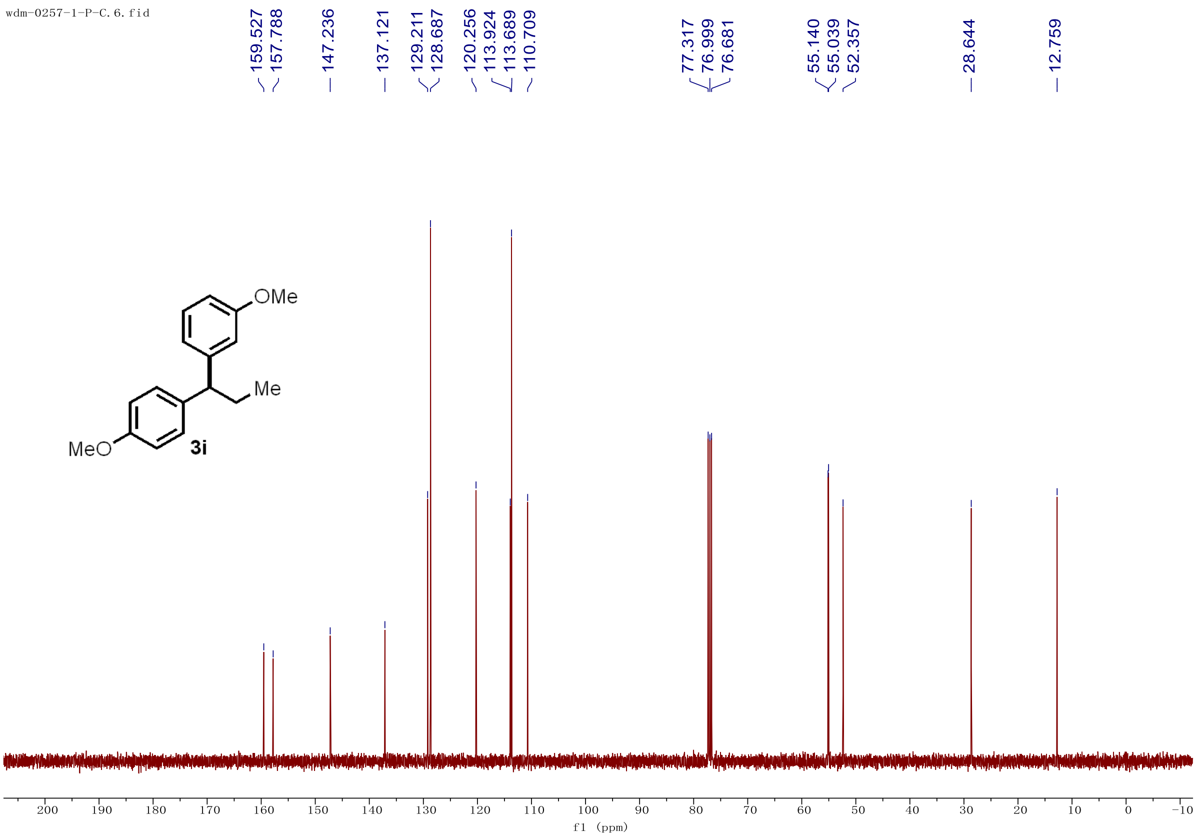

Supplementary Figure 75. ^{13}C NMR (100 MHz, $CDCl_3$) spectrum of 3i

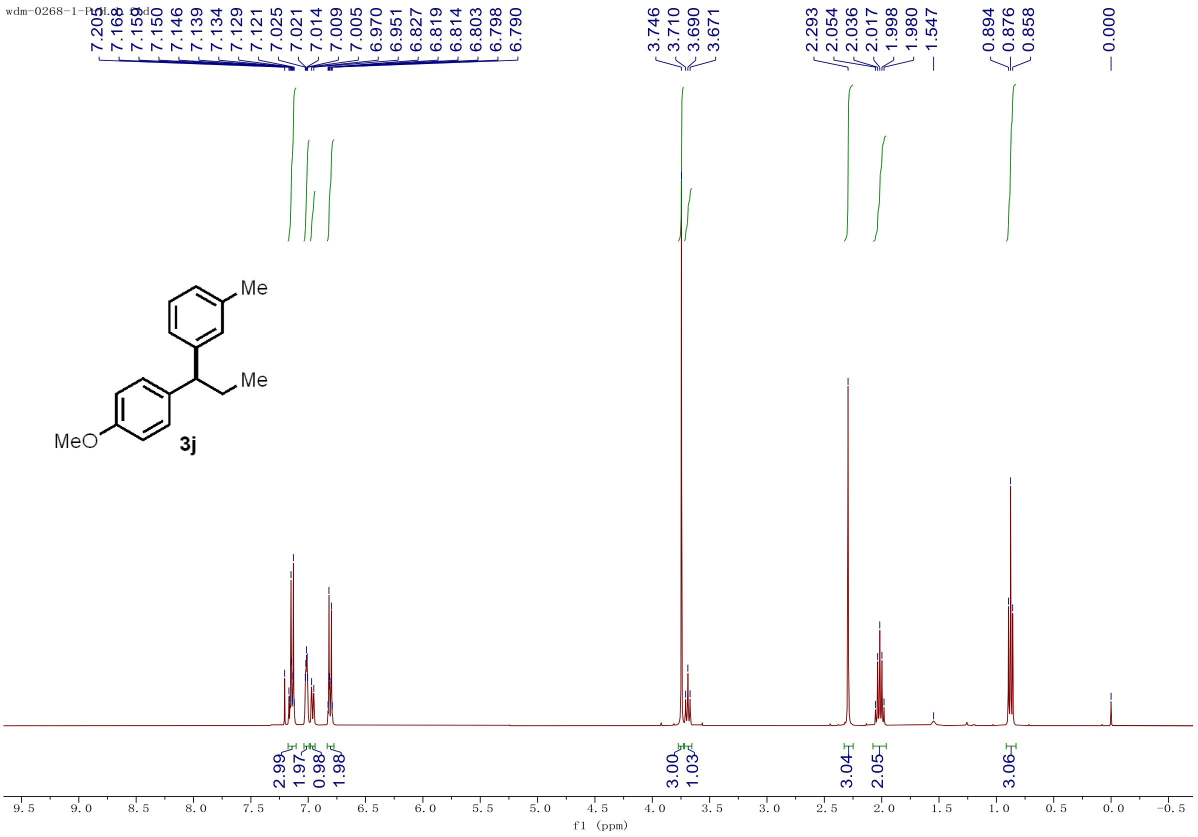

Supplementary Figure 76. ^{1}H NMR (400 MHz, $CDCl_3$) spectrum of 3j

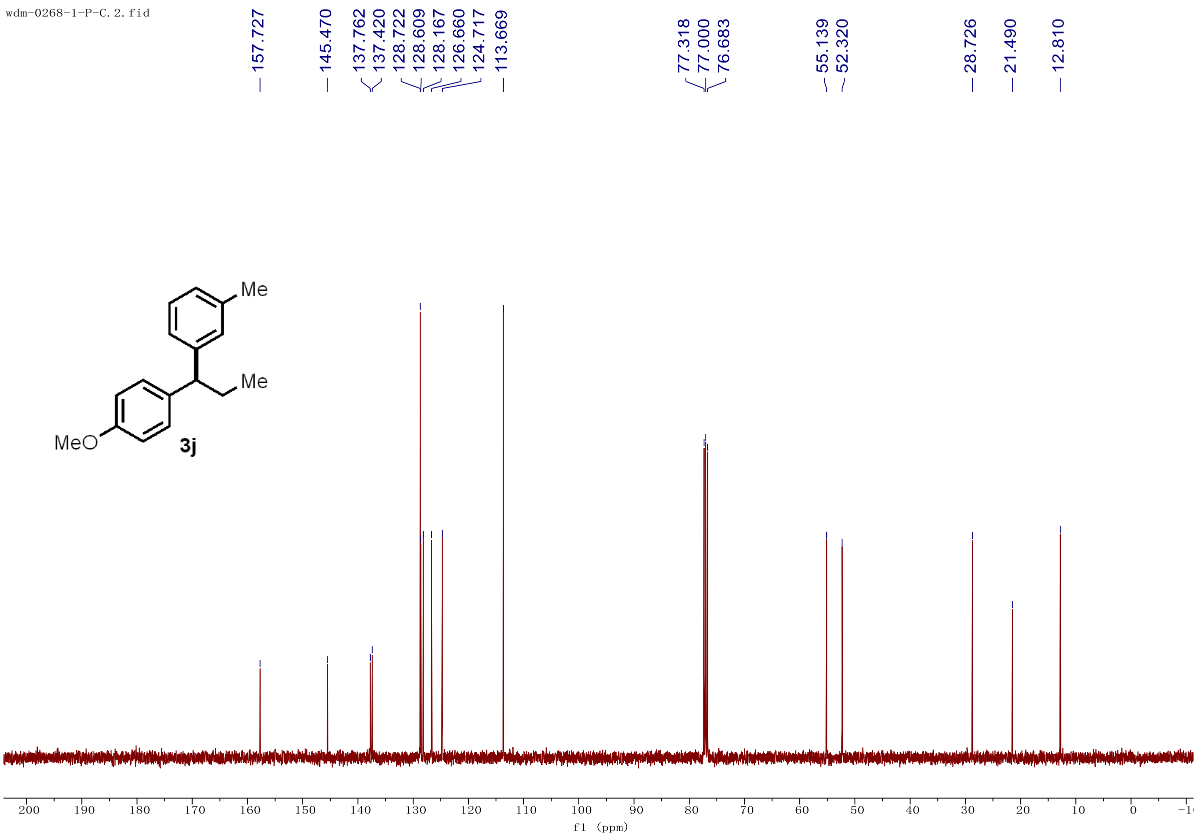

Supplementary Figure 77. ^{13}C NMR (100 MHz, $CDCl_3$) spectrum of 3j

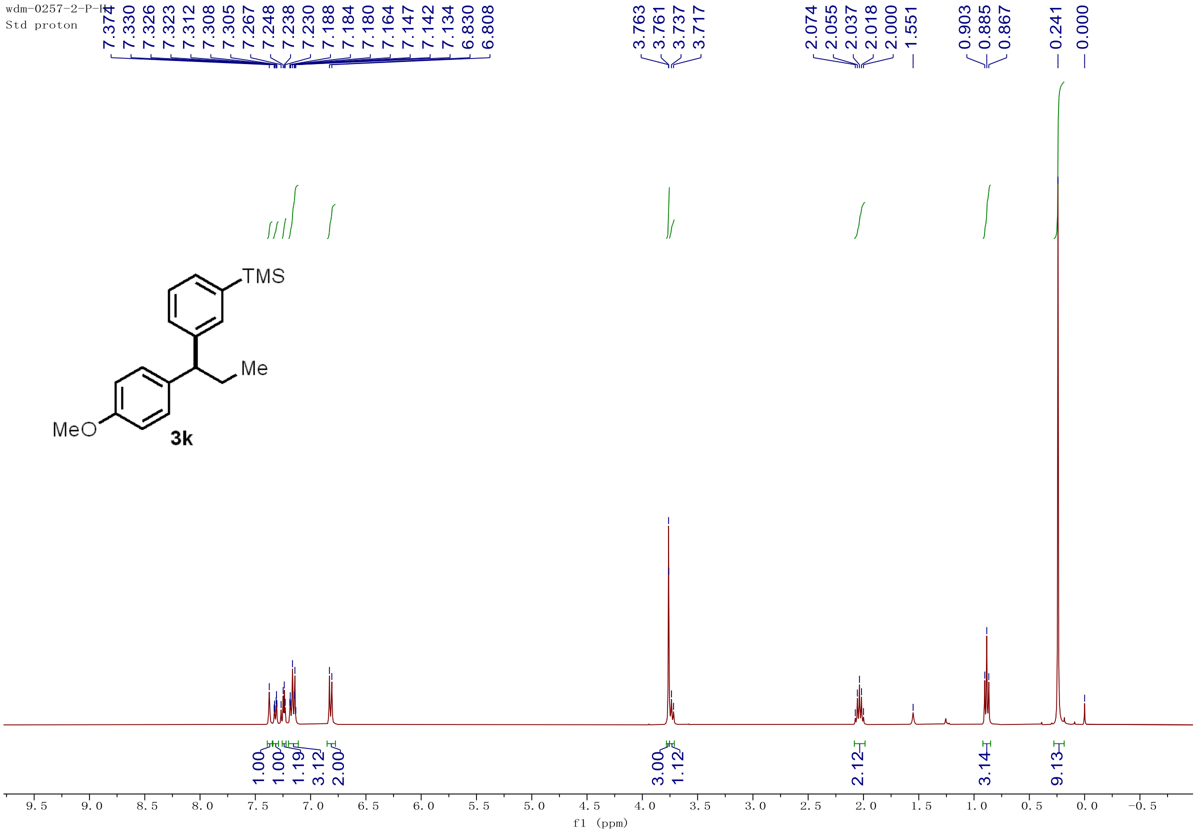

Supplementary Figure 78. ^{1}H NMR (400 MHz, $CDCl_3$) spectrum of 3k

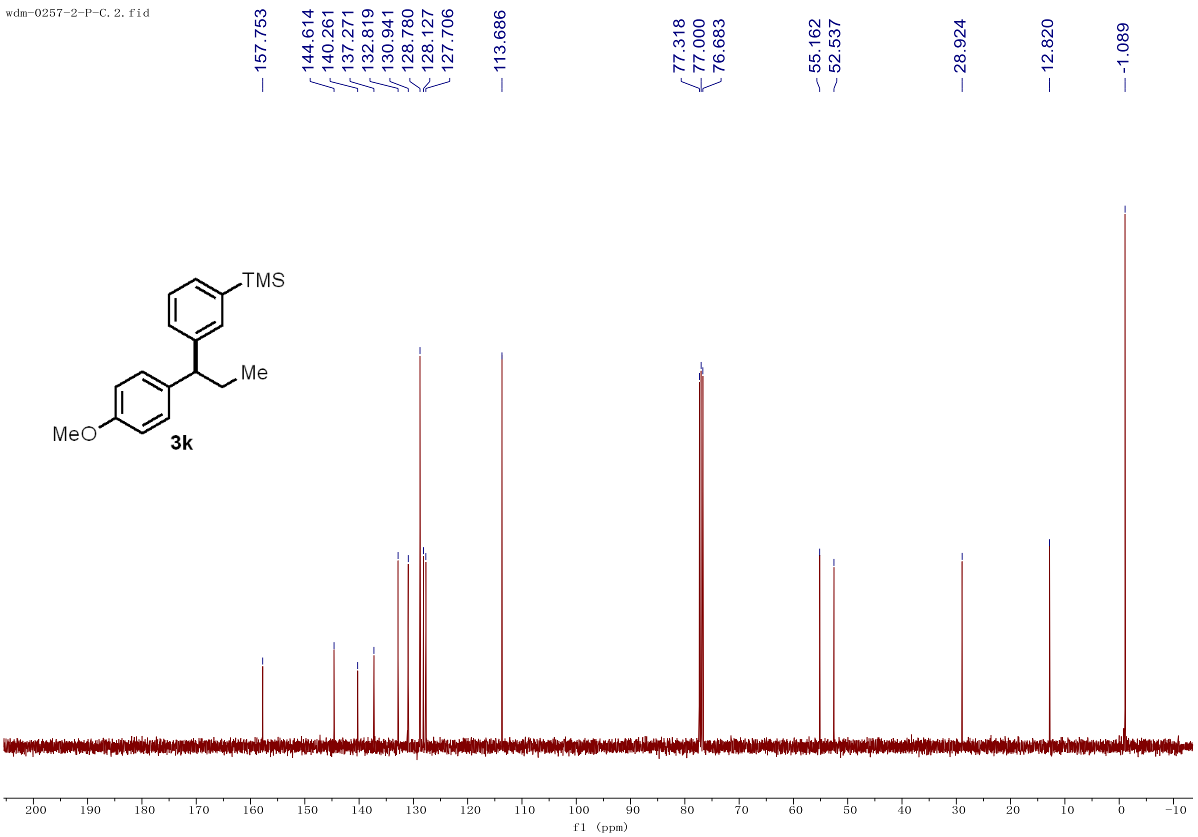

Supplementary Figure 79. ^{13}C NMR (100 MHz, $CDCl_3$) spectrum of 3k

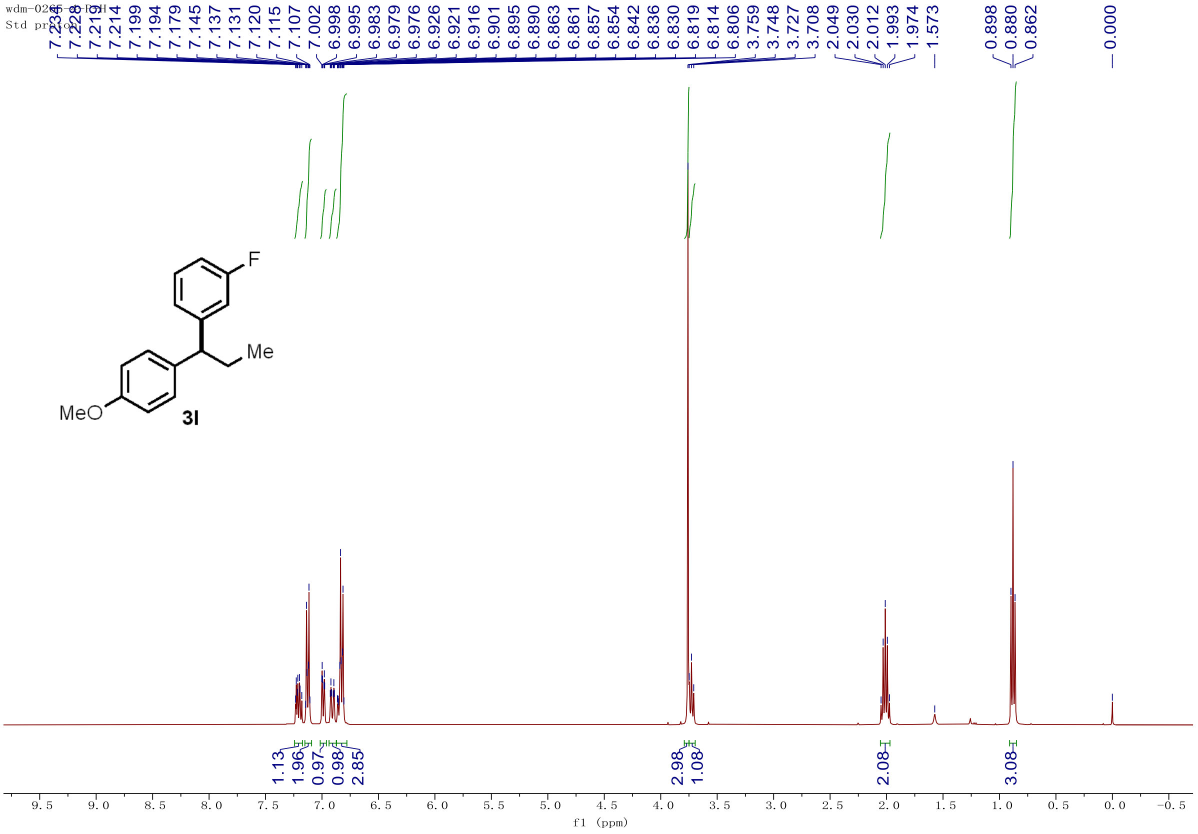

Supplementary Figure 80. ^{1}H NMR (400 MHz, $CDCl_3$) spectrum of 3l

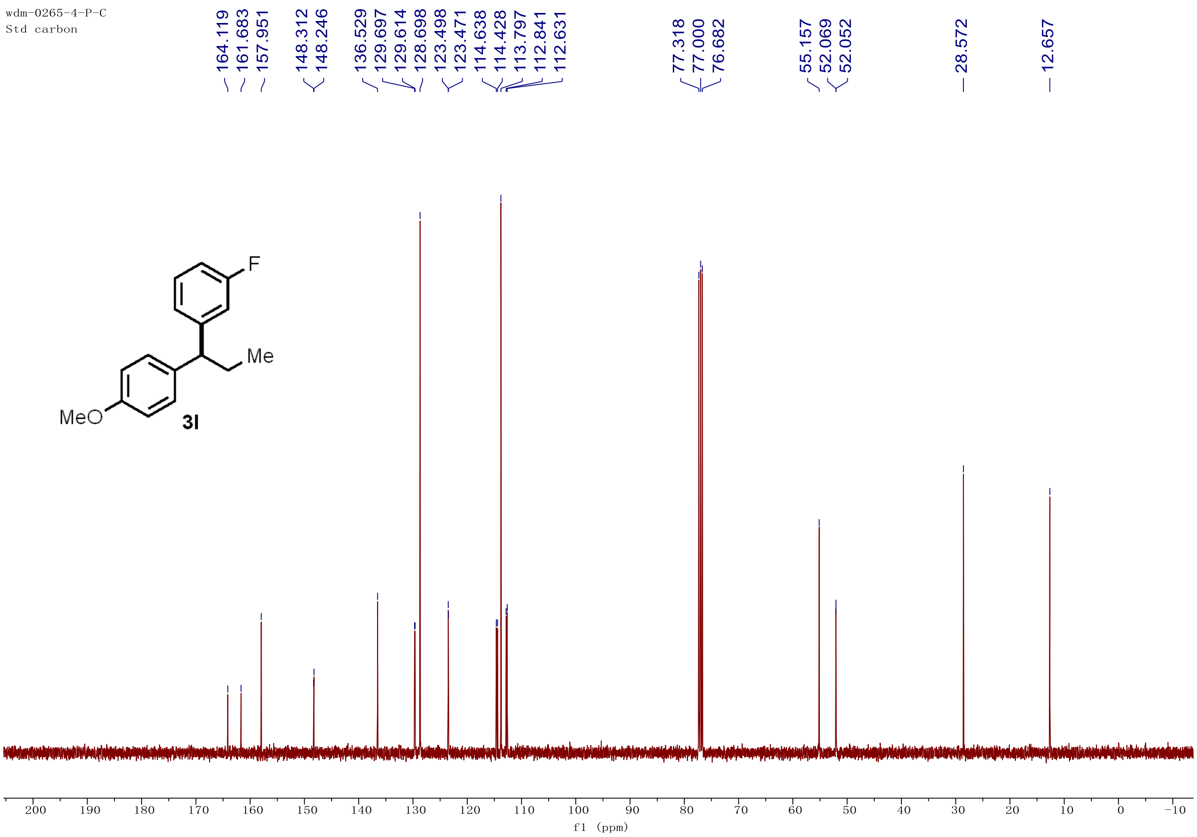

Supplementary Figure 81 ^{13}C NMR (100 MHz, CDCl$_3$) spectrum of 3l

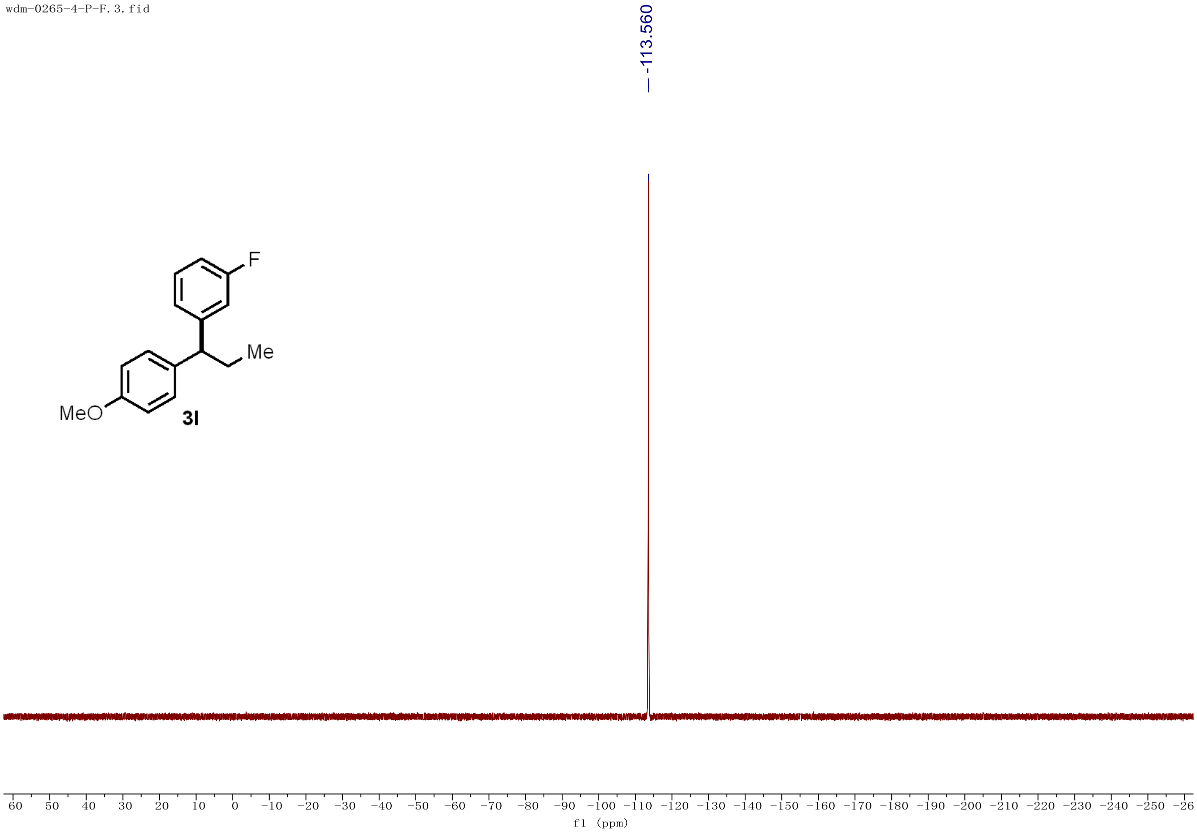

Supplementary Figure 82. ^{19}F NMR (100 MHz, CDCl$_3$) spectrum of 3l

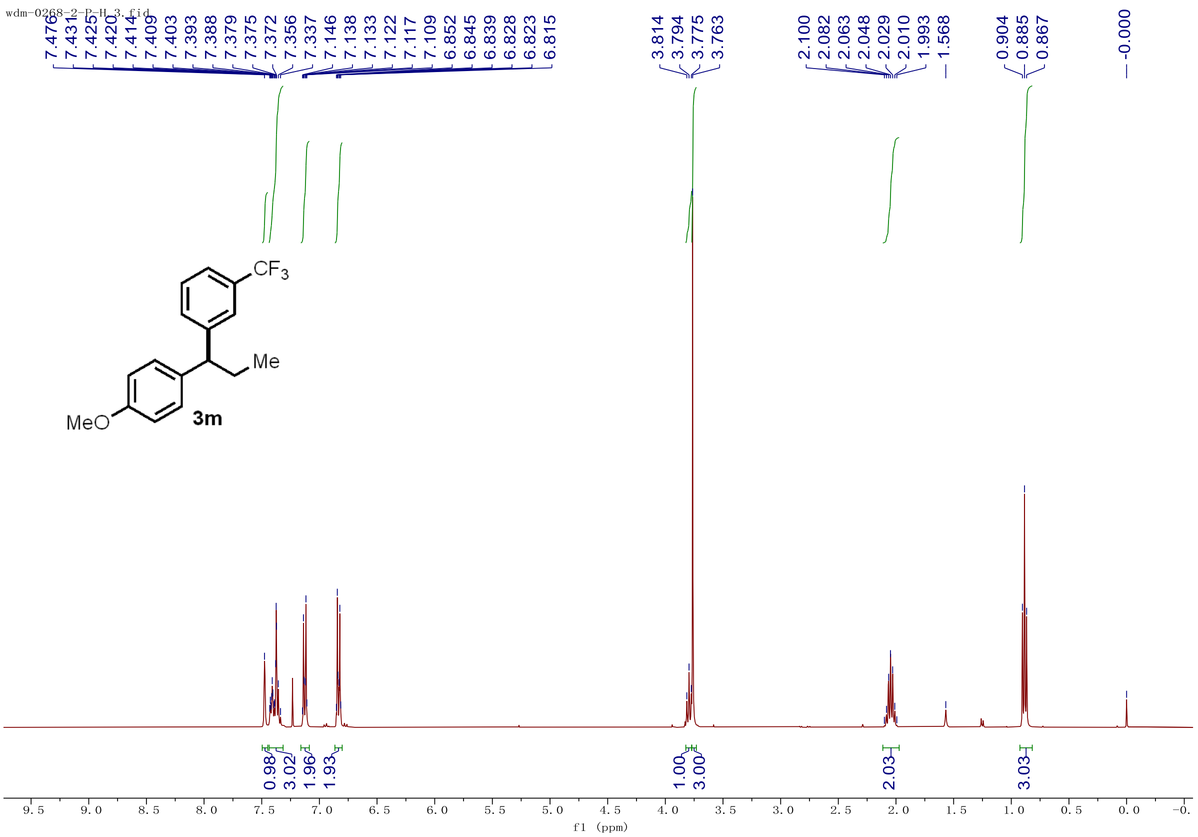

Supplementary Figure 83. **^{1}H NMR (400 MHz, $CDCl_3$) spectrum of 3m**

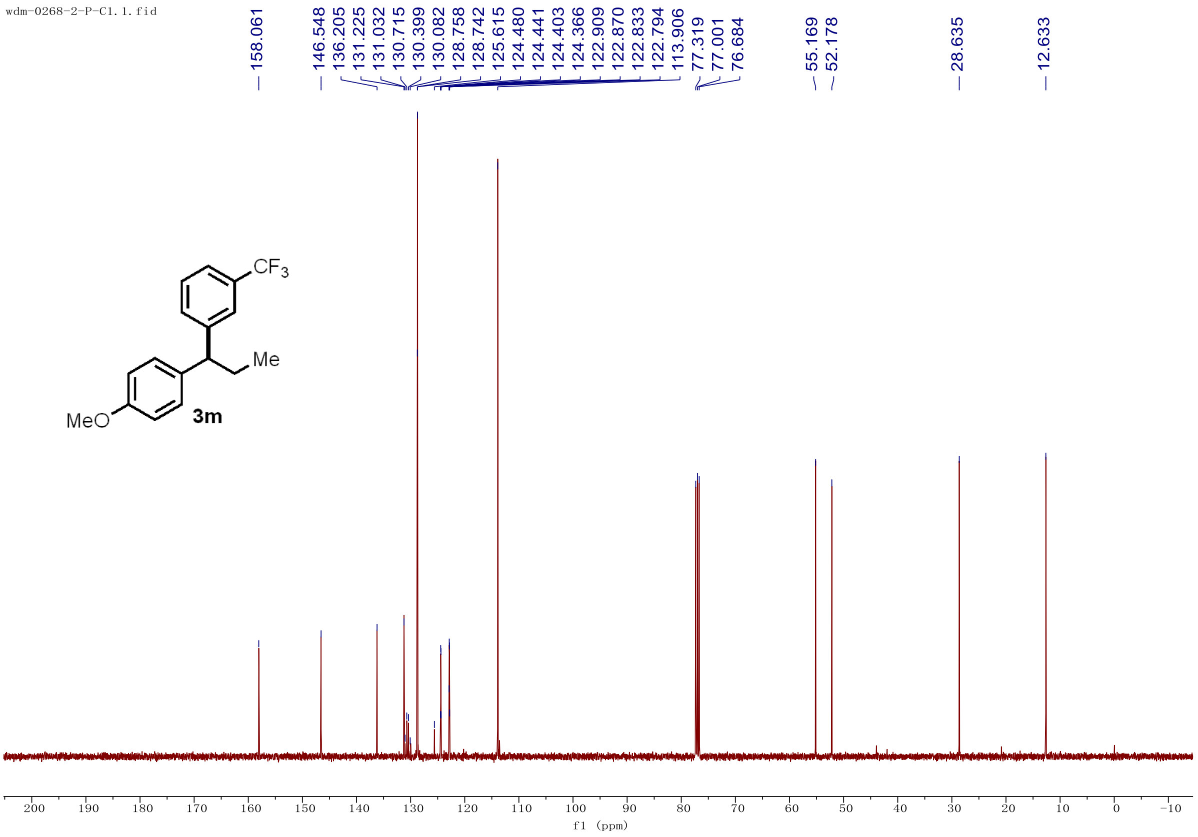

Supplementary Figure 84. **^{13}C NMR (100 MHz, $CDCl_3$) spectrum of 3m**

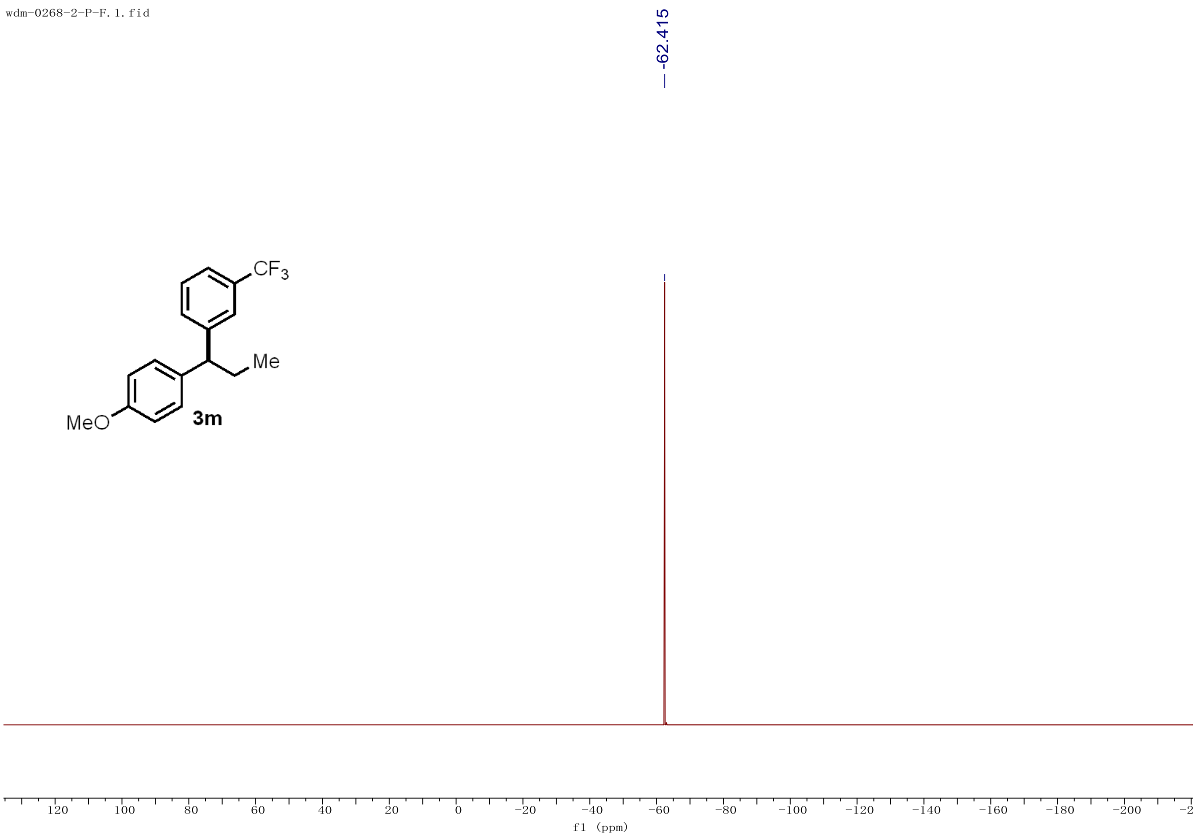

Supplementary Figure 85. ^{19}F NMR (100 MHz, CDCl$_3$) spectrum of 3m

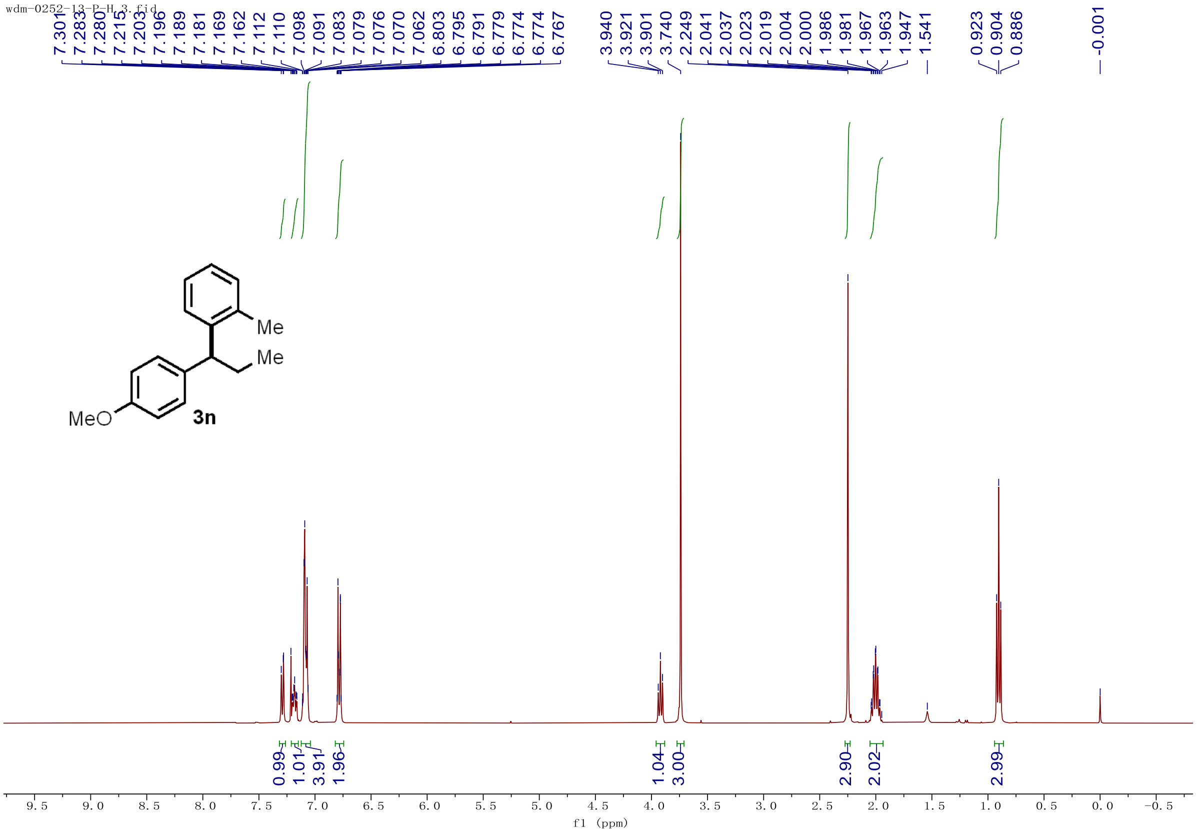

Supplementary Figure 86 ^{1}H NMR (400 MHz, CDCl$_3$) spectrum of 3n

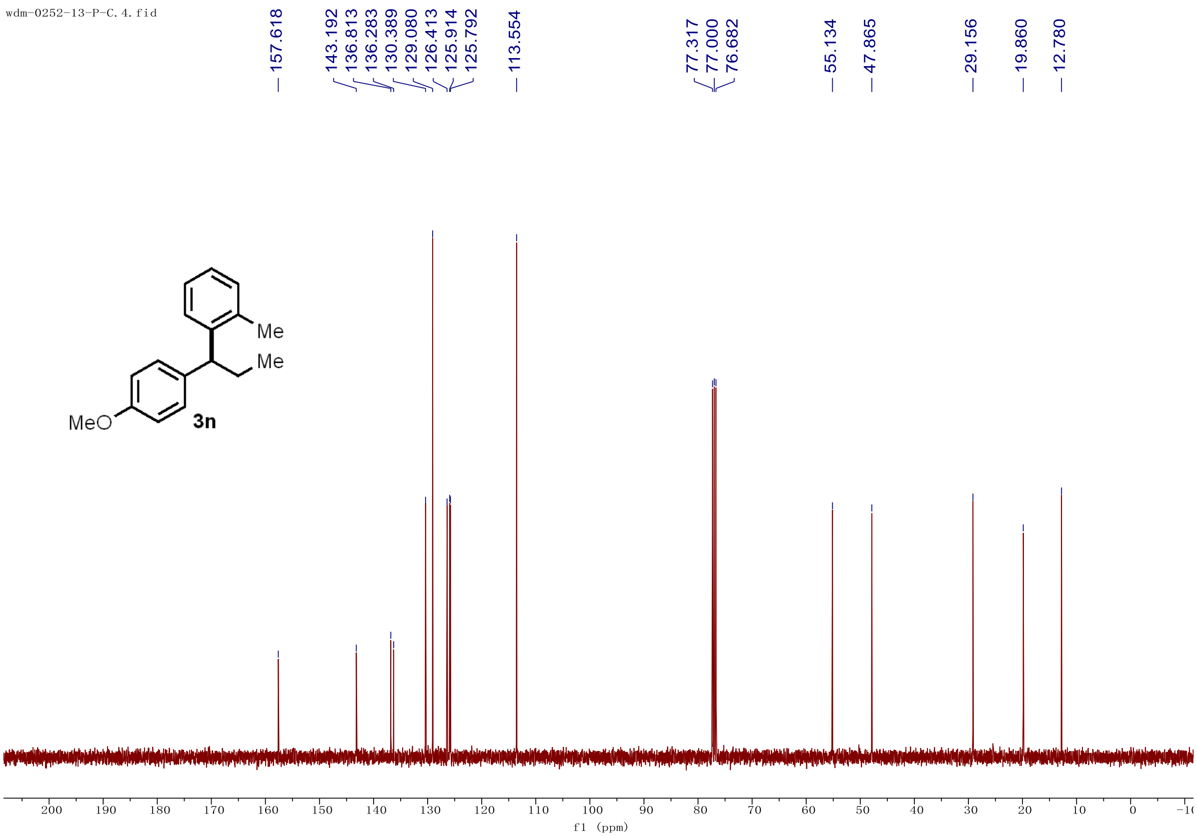

Supplementary Figure 87. ^{13}C NMR (100 MHz, $CDCl_3$) spectrum of 3n

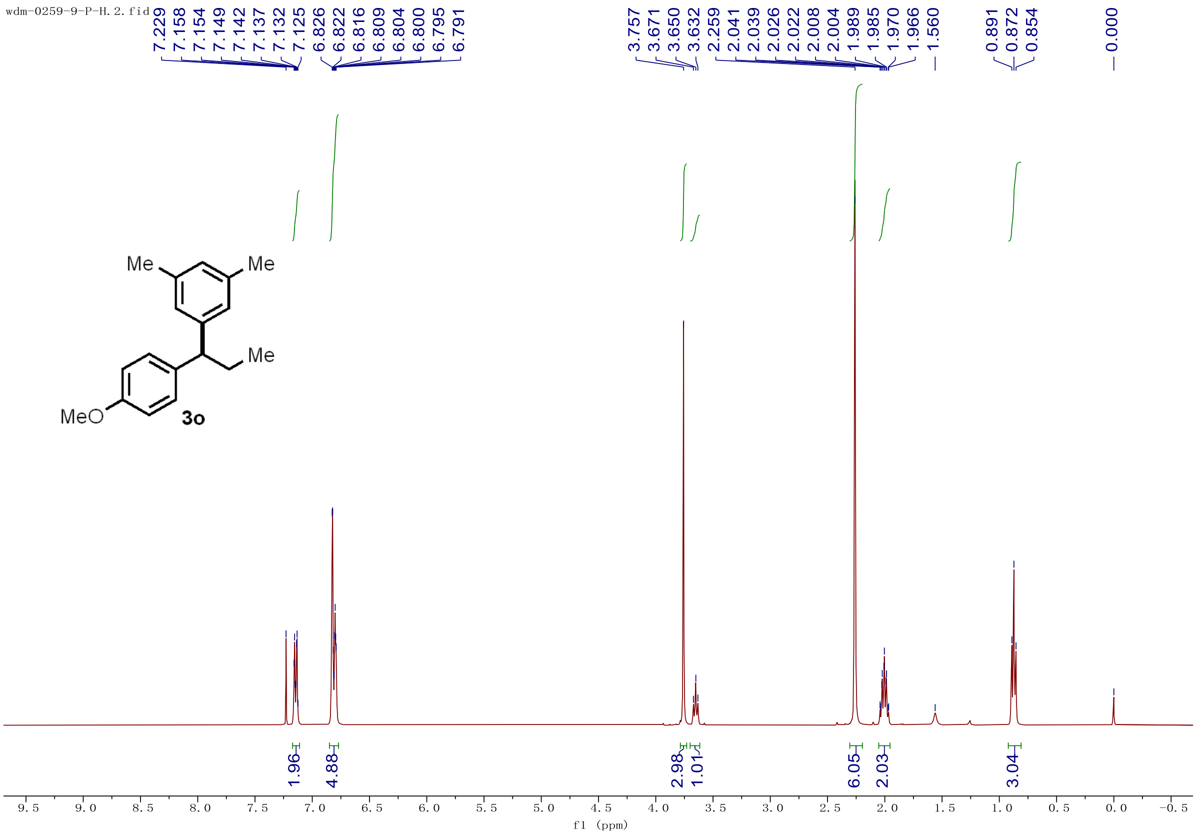

Supplementary Figure 88. ^{1}H NMR (400 MHz, $CDCl_3$) spectrum of 3o

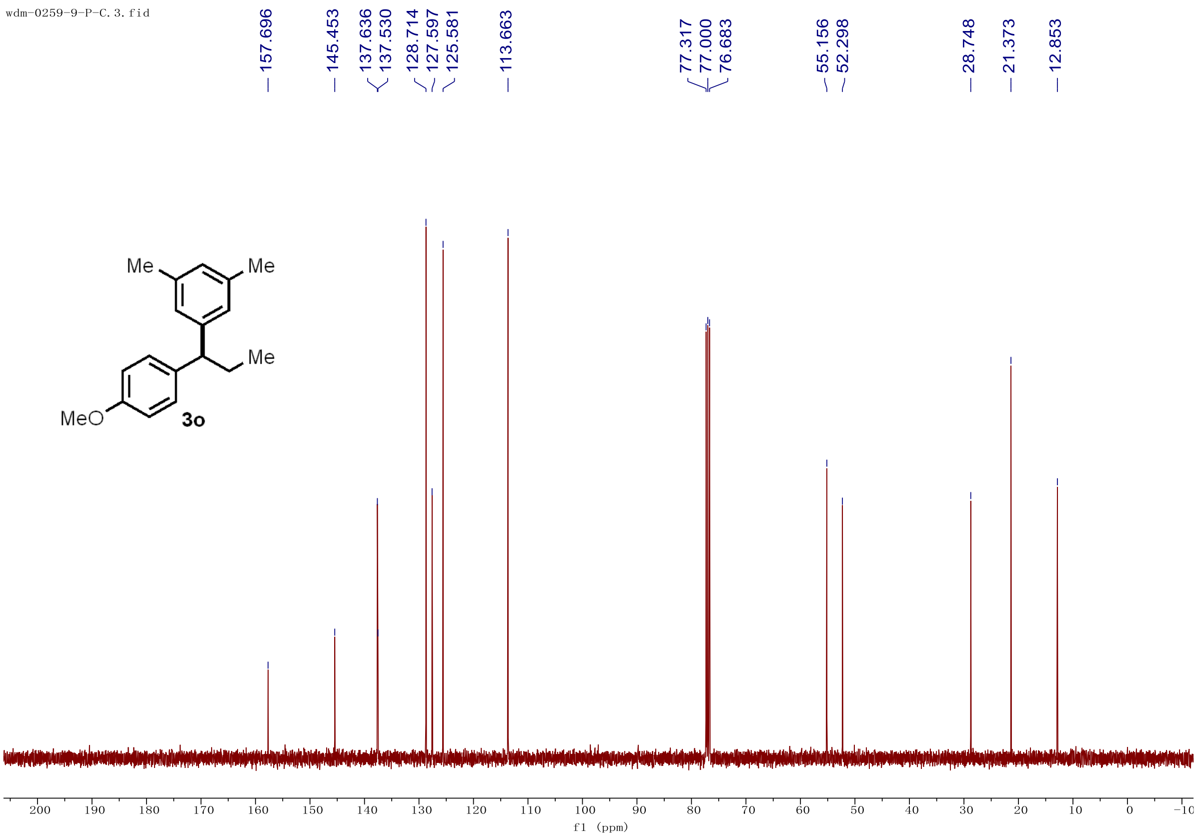

Supplementary Figure 89. ^{13}C NMR (100 MHz, $CDCl_3$) spectrum of 3o

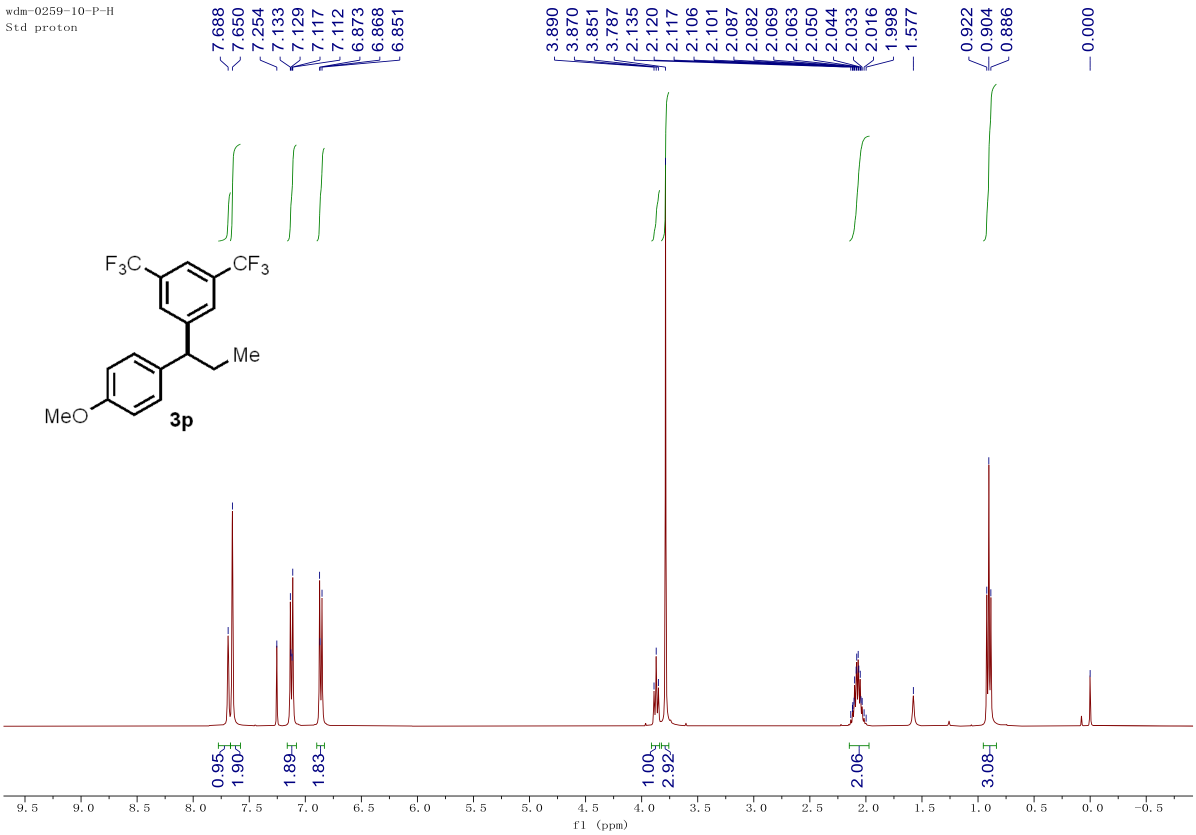

Supplementary Figure 90. ^{1}H NMR (400 MHz, $CDCl_3$) spectrum of 3p

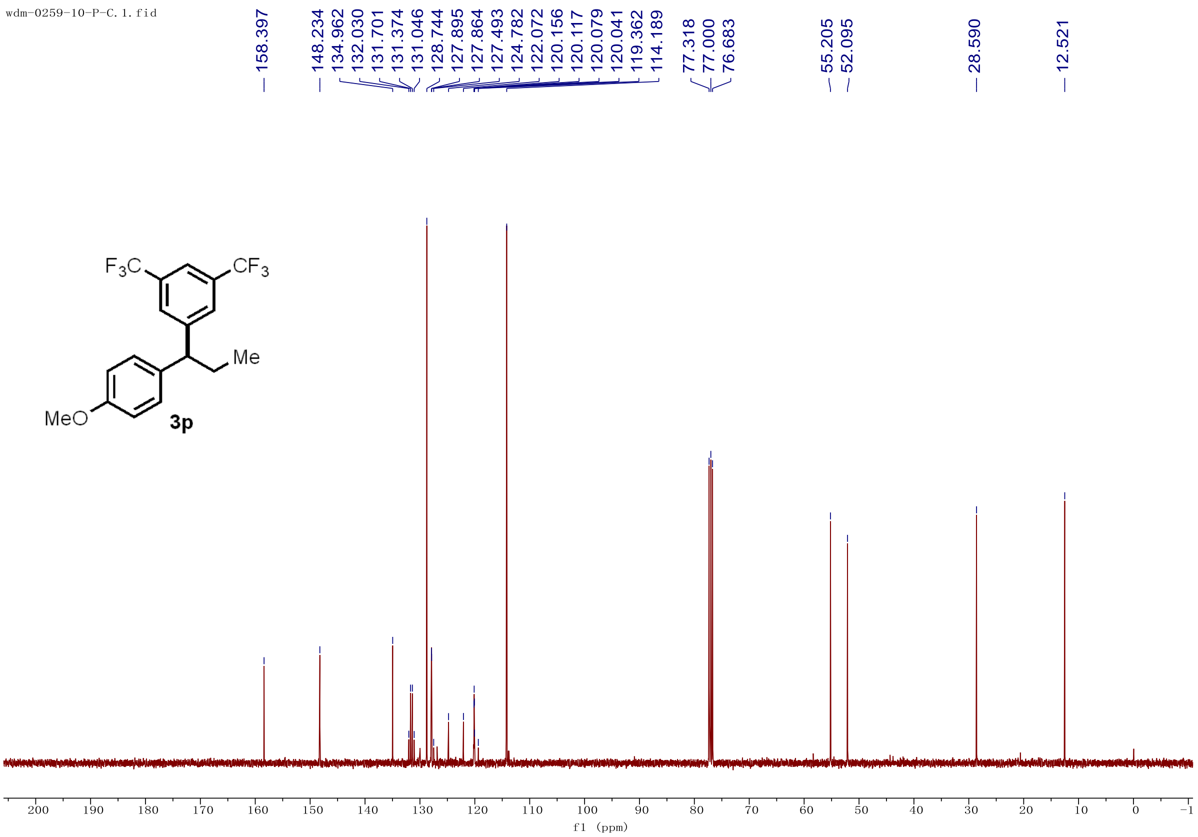

Supplementary Figure 91. ^{13}**C NMR (100 MHz,** $CDCl_3$**) spectrum of 3p**

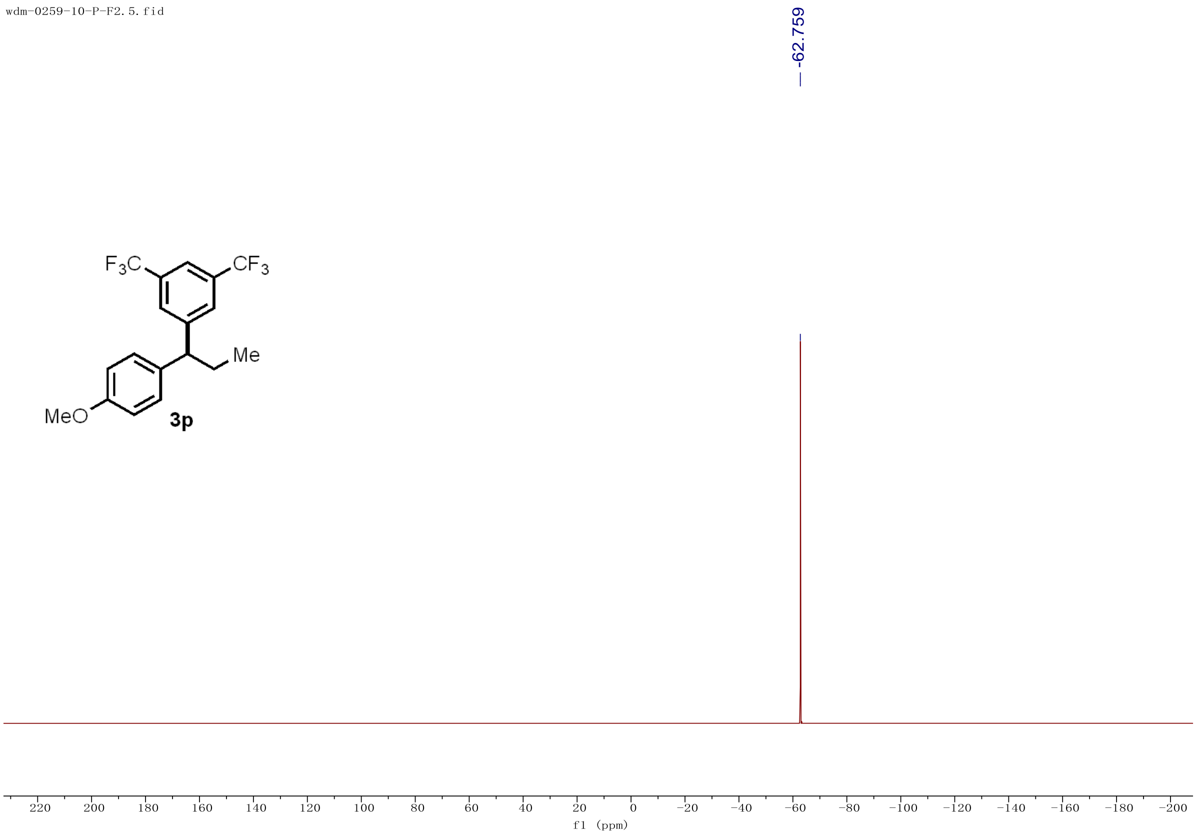

Supplementary Figure 92. ^{19}**F NMR (375 MHz,** $CDCl_3$**) spectrum of 3p**

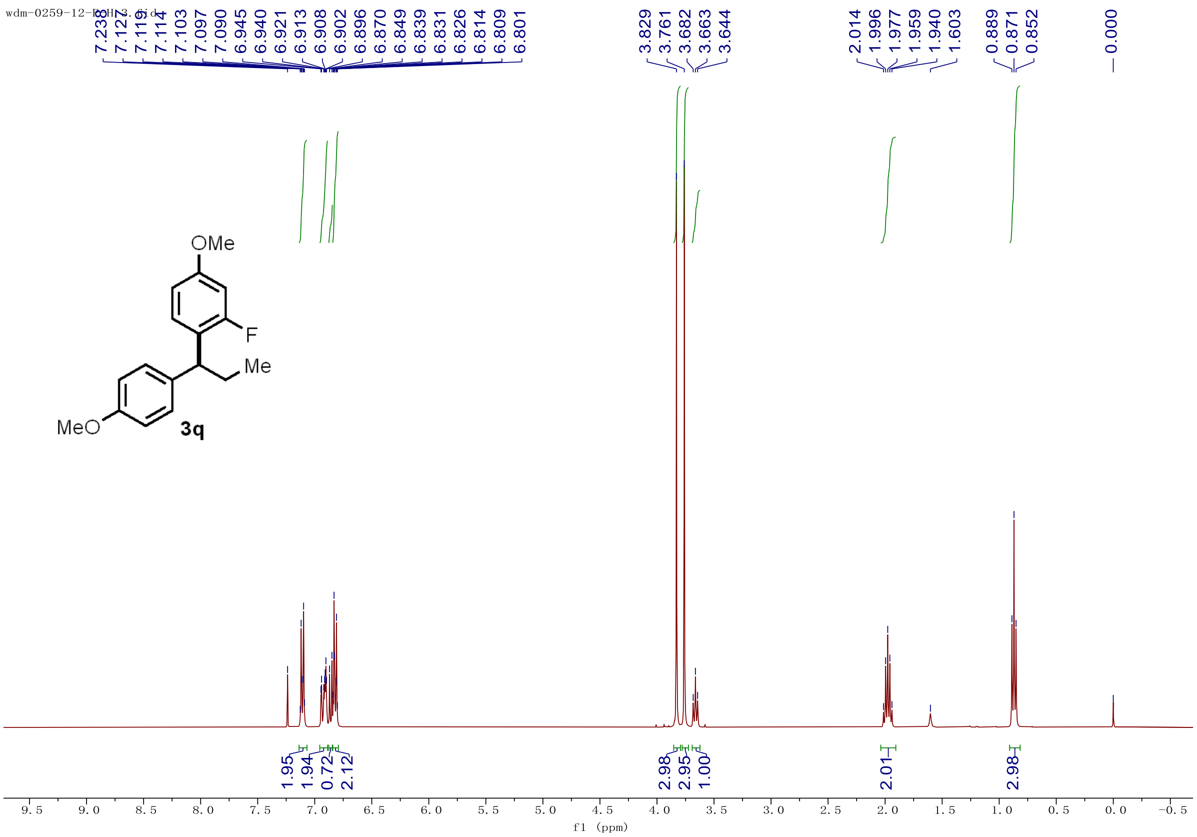

Supplementary Figure 93. ^{1}H **NMR (400 MHz,** $CDCl_3$**) spectrum of 3q**

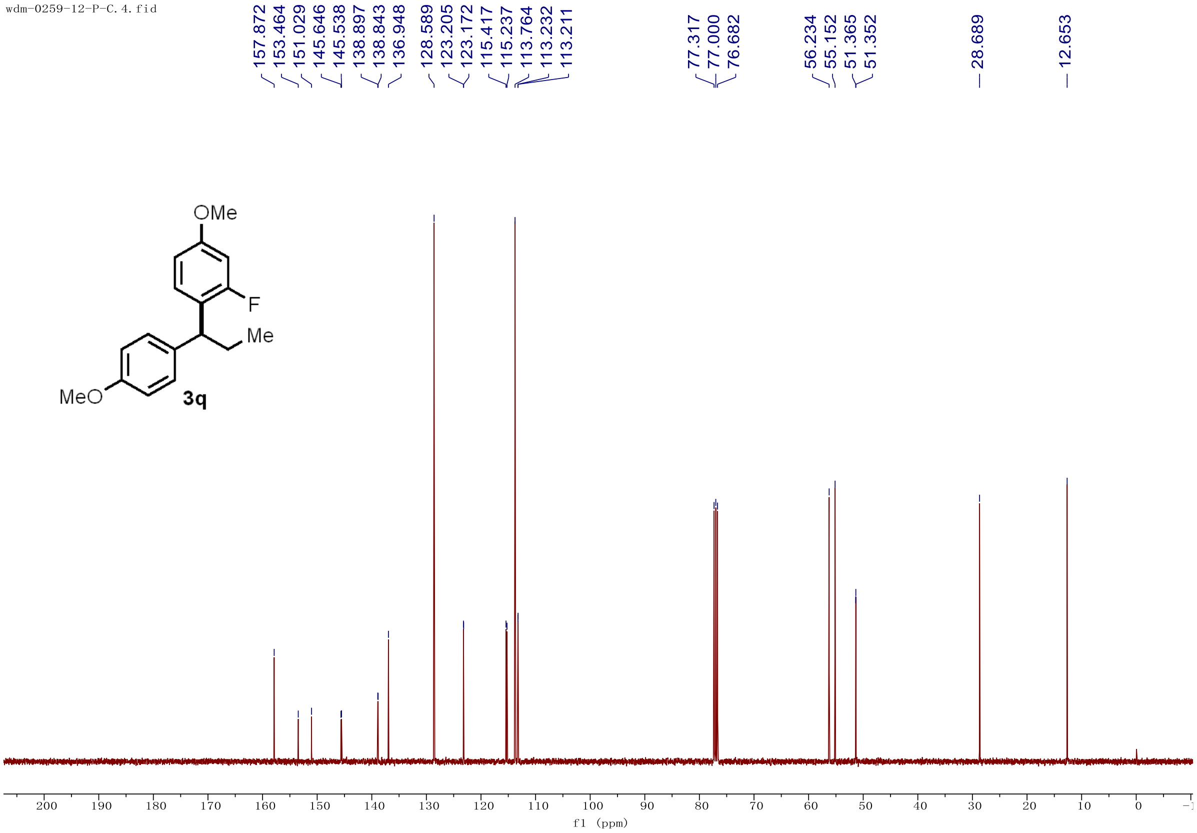

Supplementary Figure 94. ^{13}C **NMR (100 MHz,** $CDCl_3$**) spectrum of 3q**

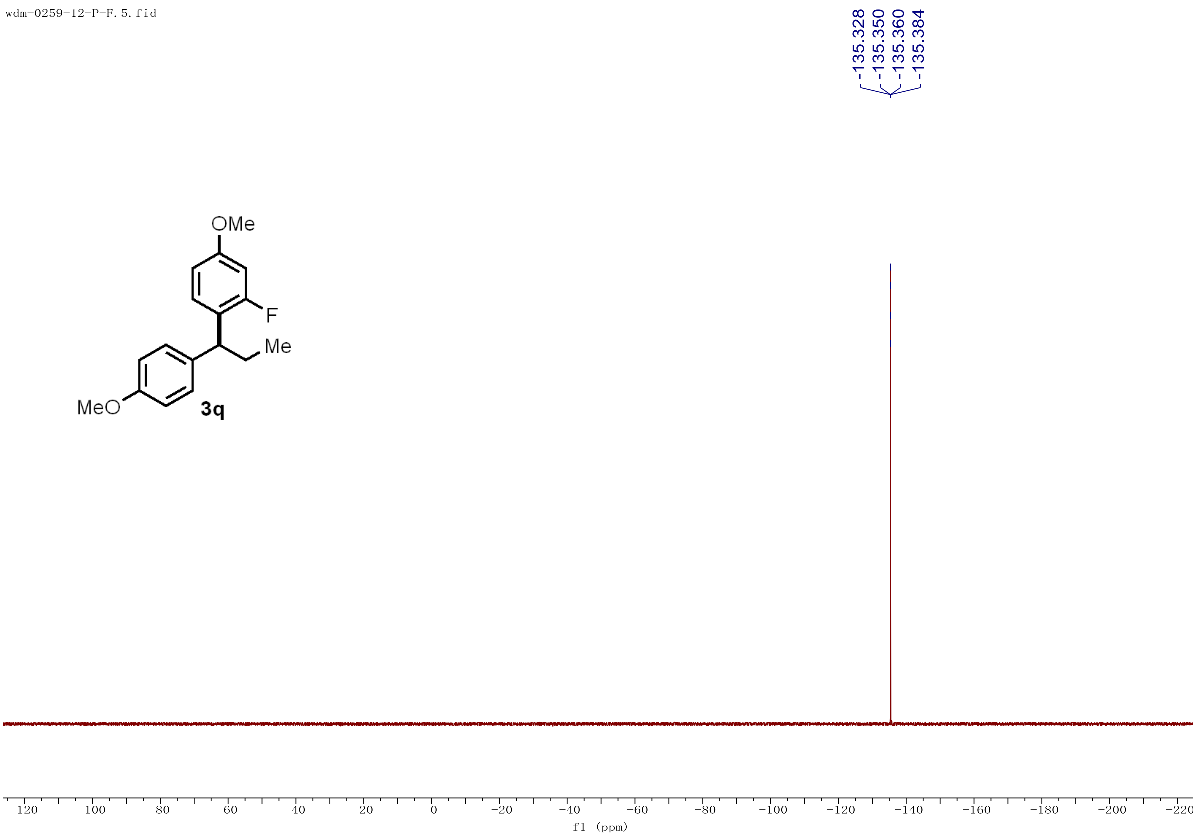

Supplementary Figure 95. ^{19}F NMR (375 MHz, $CDCl_3$) spectrum of 3q

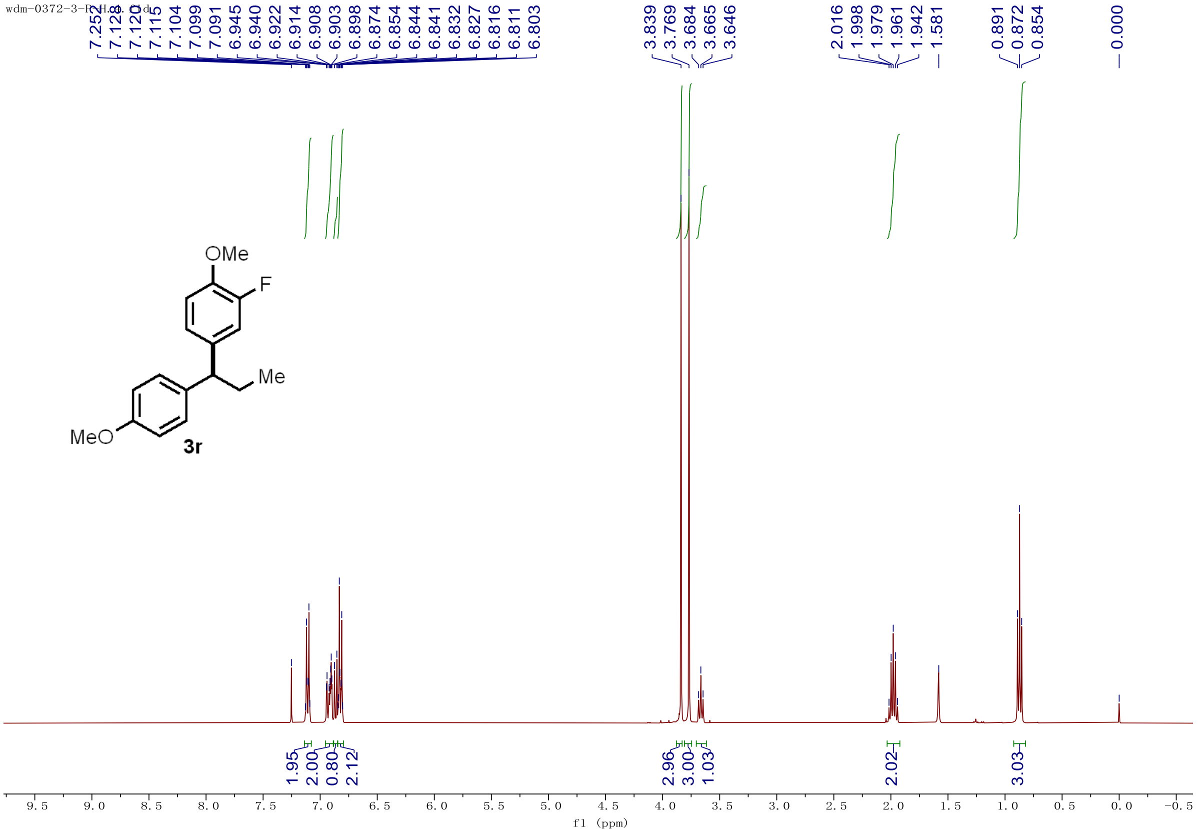

Supplementary Figure 96. ^{1}H NMR (400 MHz, $CDCl_3$) spectrum of 3r

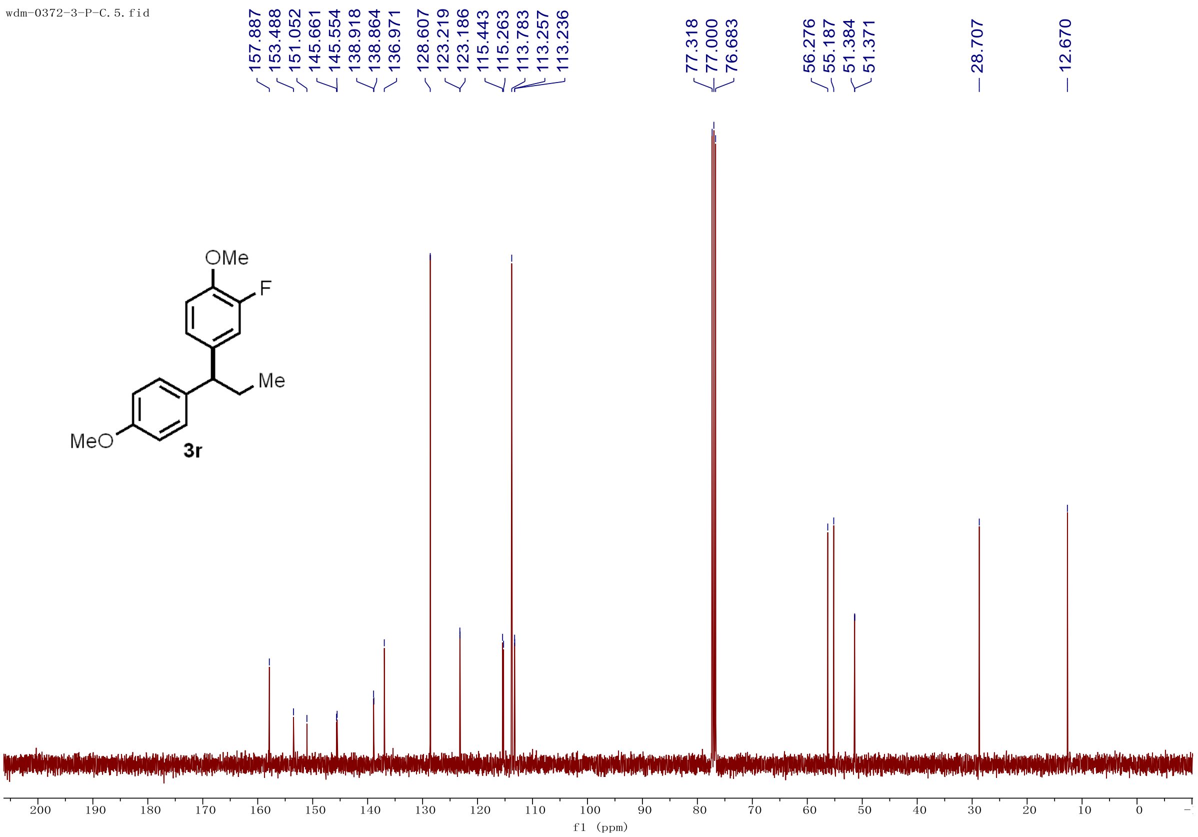

Supplementary Figure 97. ^{13}**C NMR (100 MHz, CDCl$_3$) spectrum of 3r**

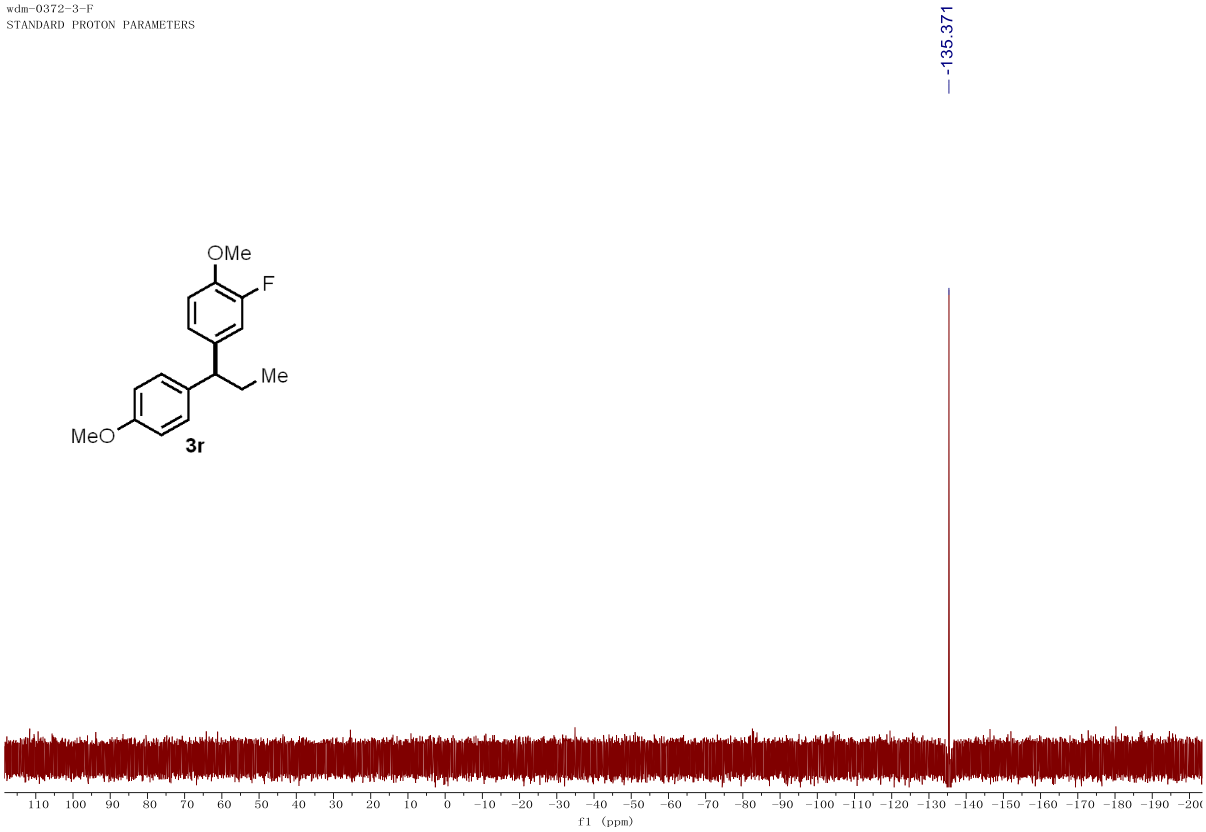

Supplementary Figure 98. ^{19}**F NMR (375 MHz, CDCl$_3$) spectrum of 3r**

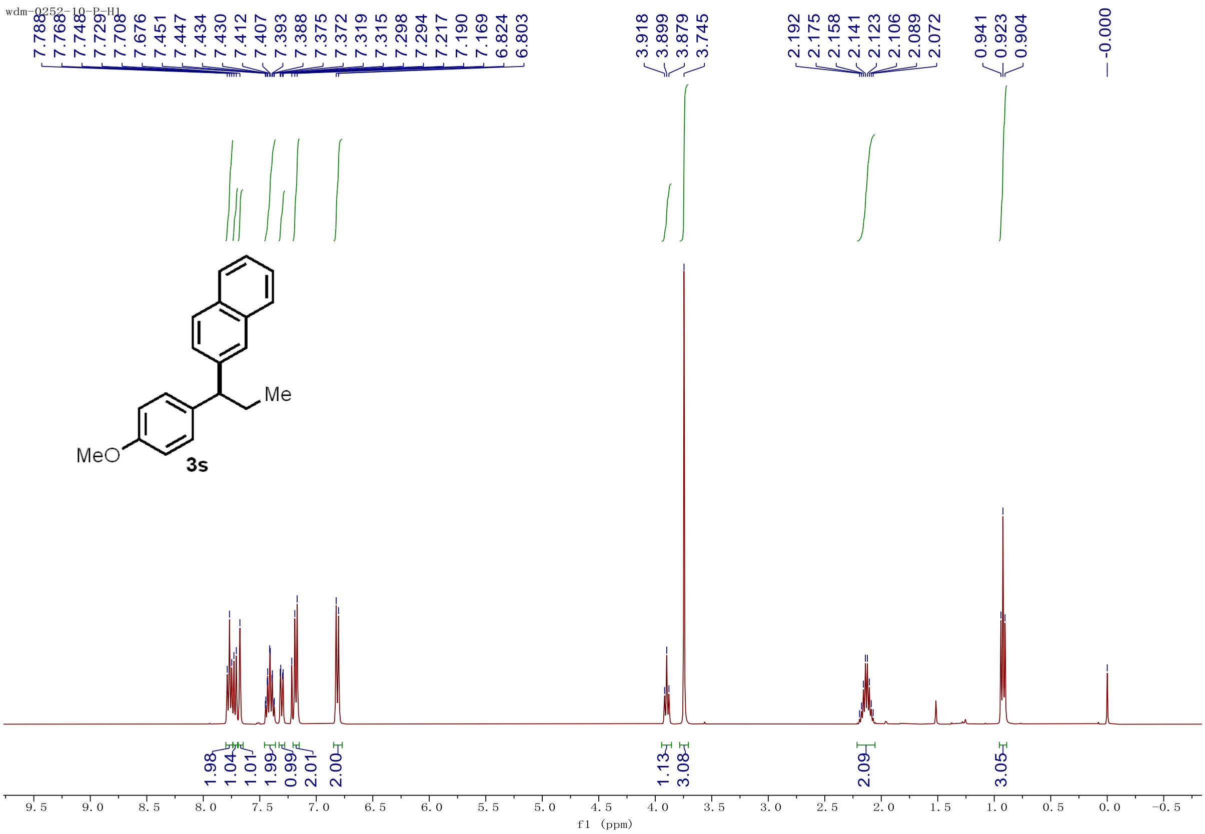

Supplementary Figure 99. ^{1}H NMR (400 MHz, CDCl$_3$) spectrum of 3s

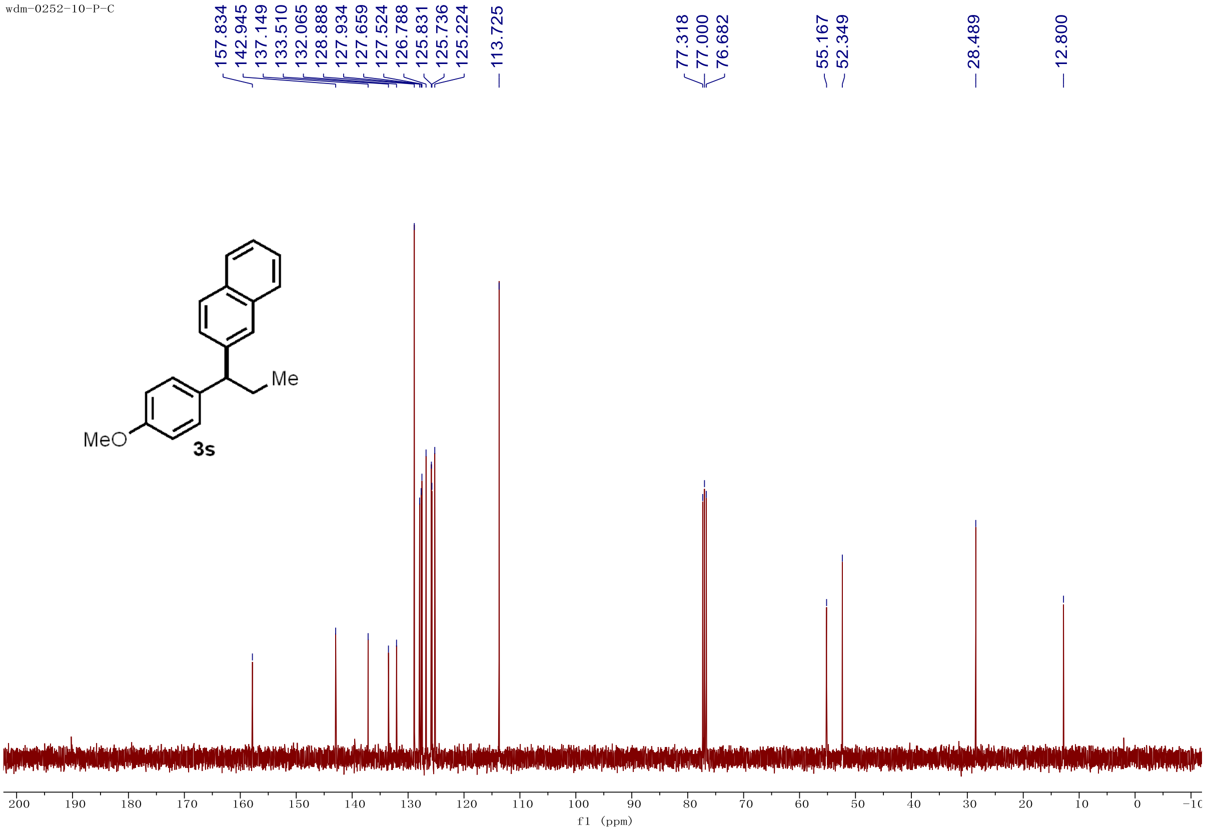

Supplementary Figure 100. ^{13}C NMR (100 MHz, CDCl$_3$) spectrum of 3s

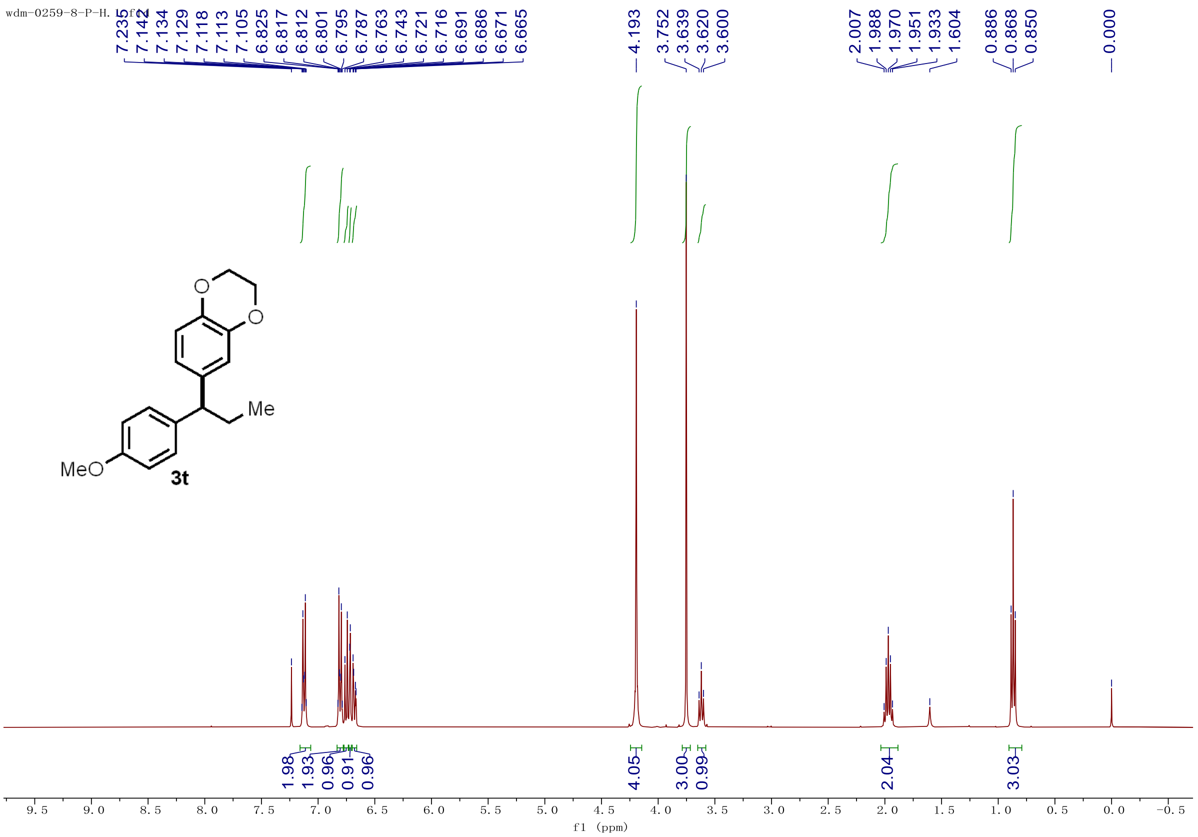

Supplementary Figure 101. ^{1}H NMR (400 MHz, CDCl$_3$) spectrum of 3t

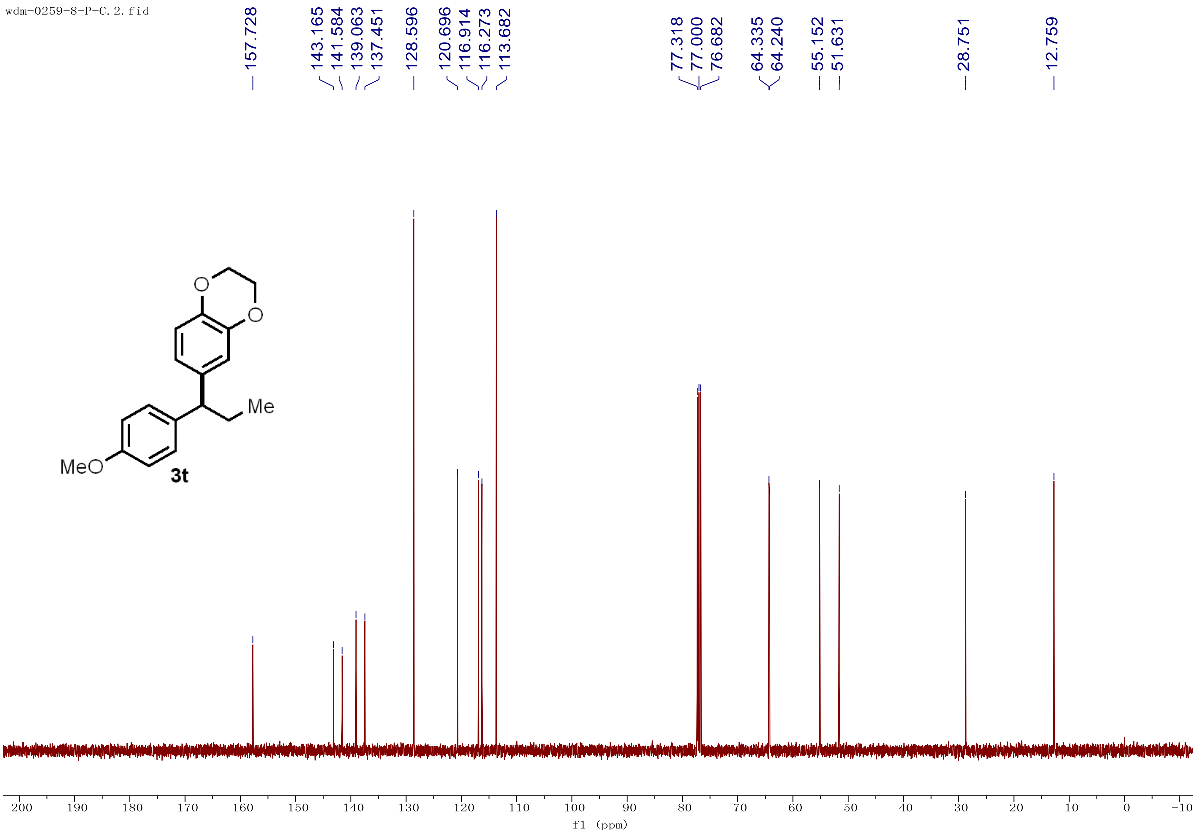

Supplementary Figure 102. ^{13}C NMR (100 MHz, CDCl$_3$) spectrum of 3t

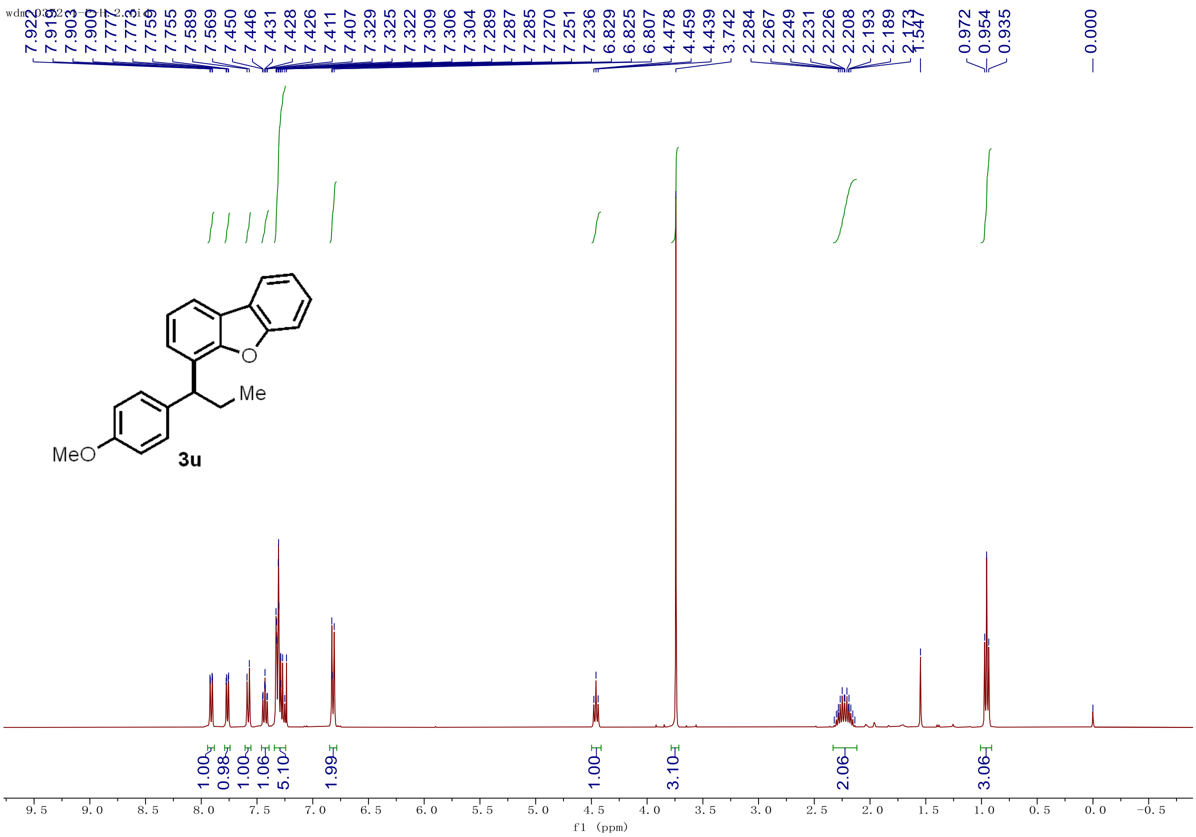

Supplementary Figure 103. ^{1}H NMR (400 MHz, $CDCl_3$) spectrum of 3u

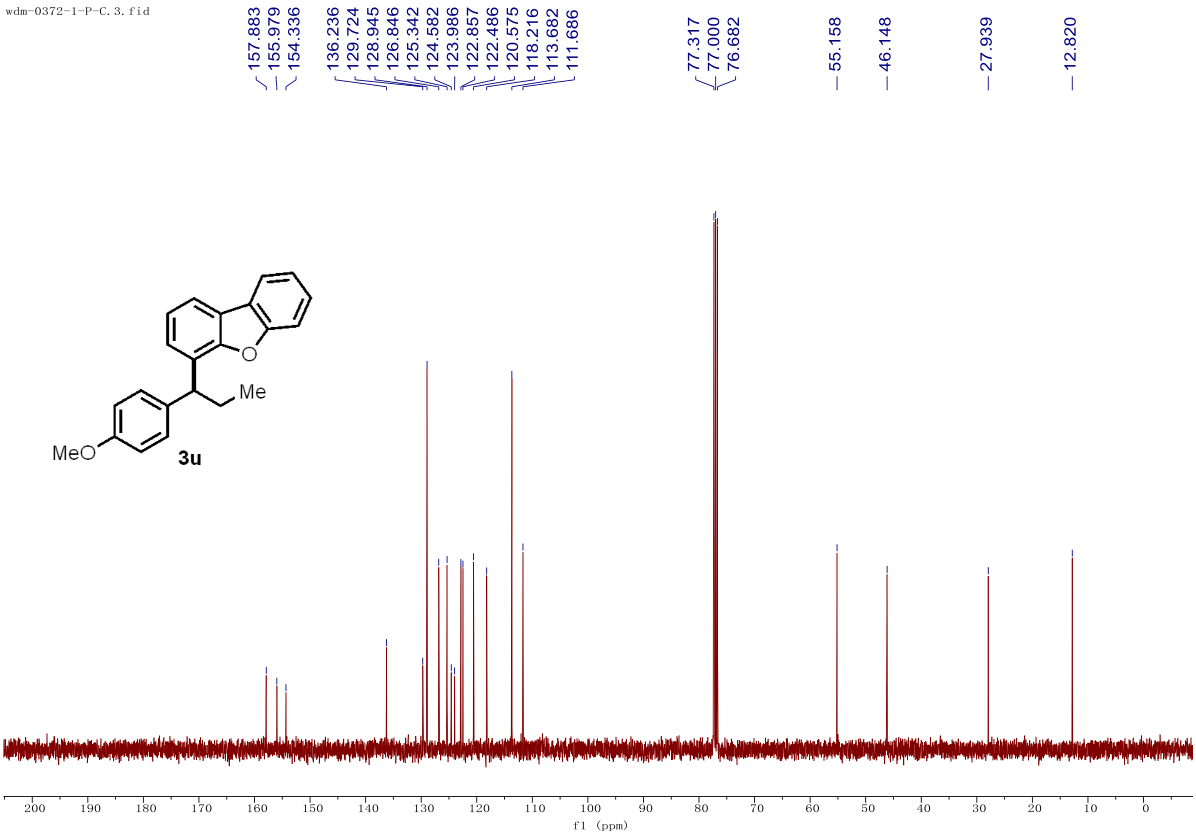

Supplementary Figure 104. ^{13}C NMR (100 MHz, $CDCl_3$) spectrum of 3u

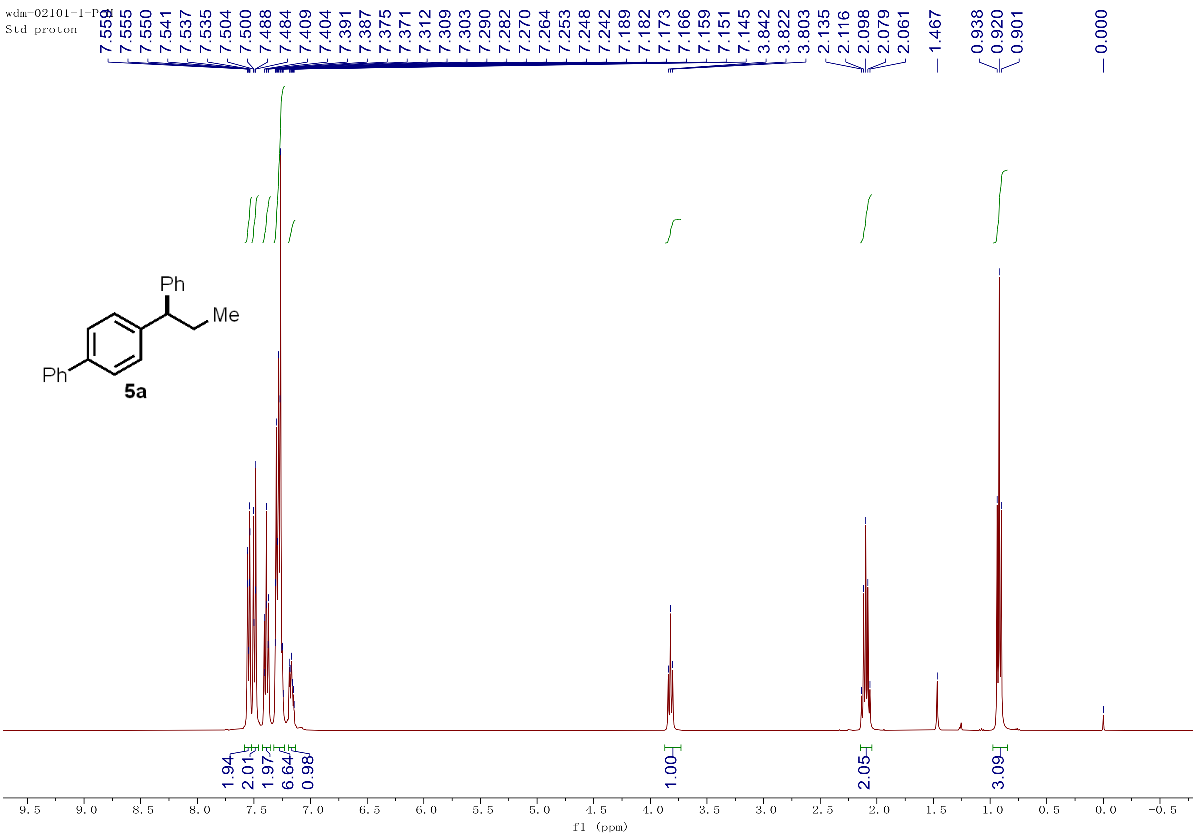

Supplementary Figure 105. ^{1}H NMR (400 MHz, CDCl$_3$) spectrum of 5a

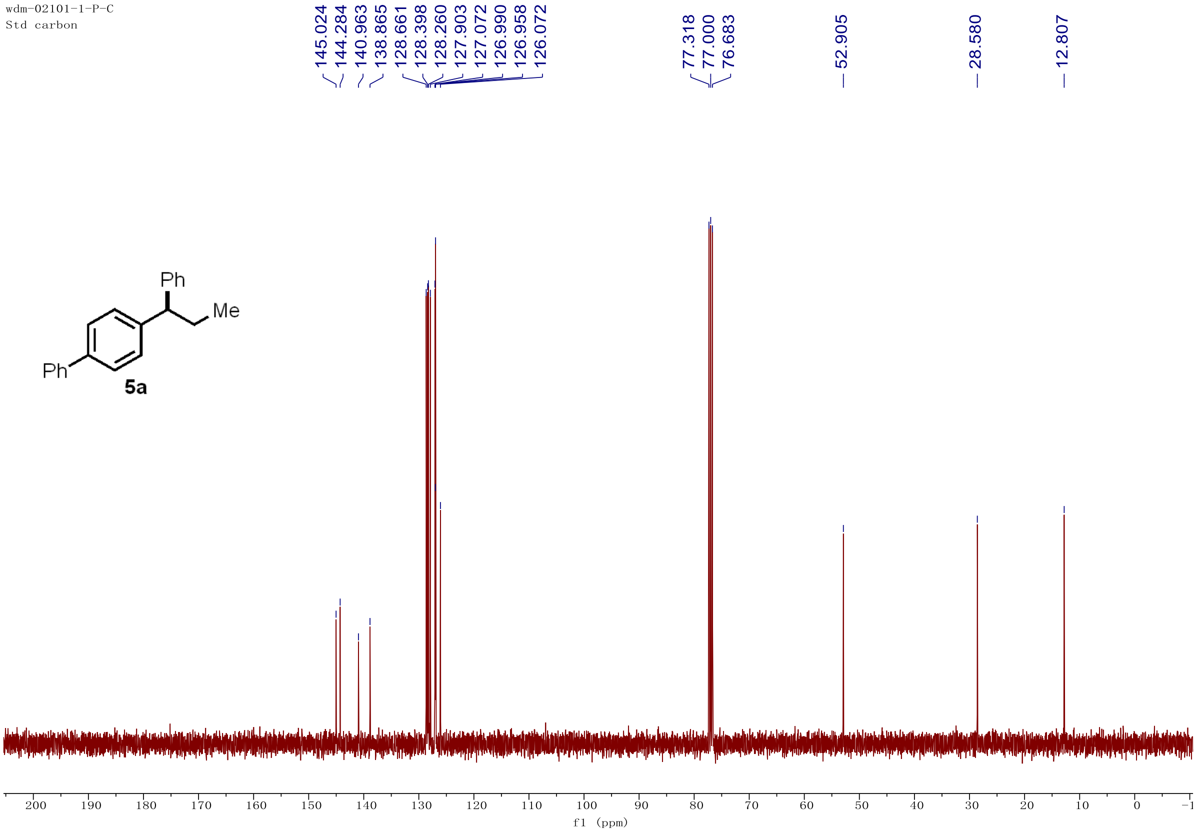

Supplementary Figure 106. ^{13}C NMR (100 MHz, CDCl$_3$) spectrum of 5a

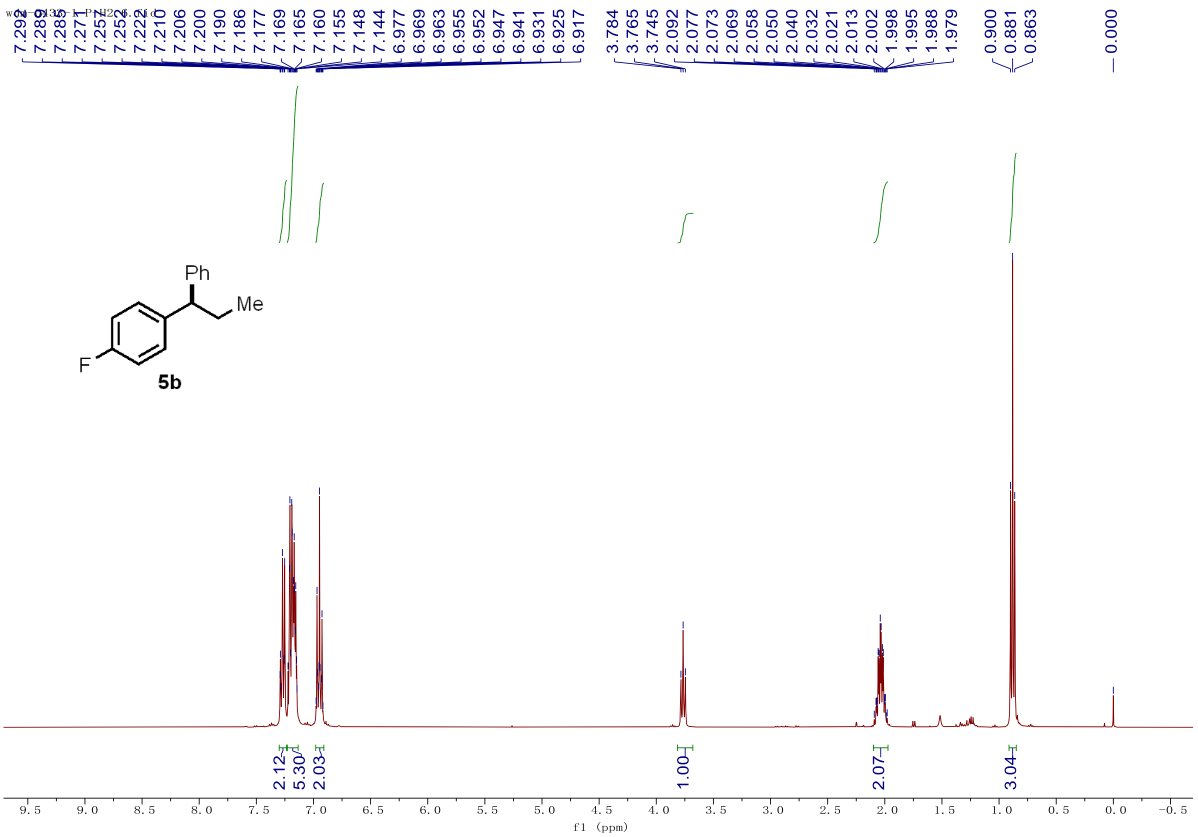

Supplementary Figure 107. ^{1}H NMR (400 MHz, $CDCl_3$) spectrum of 5b

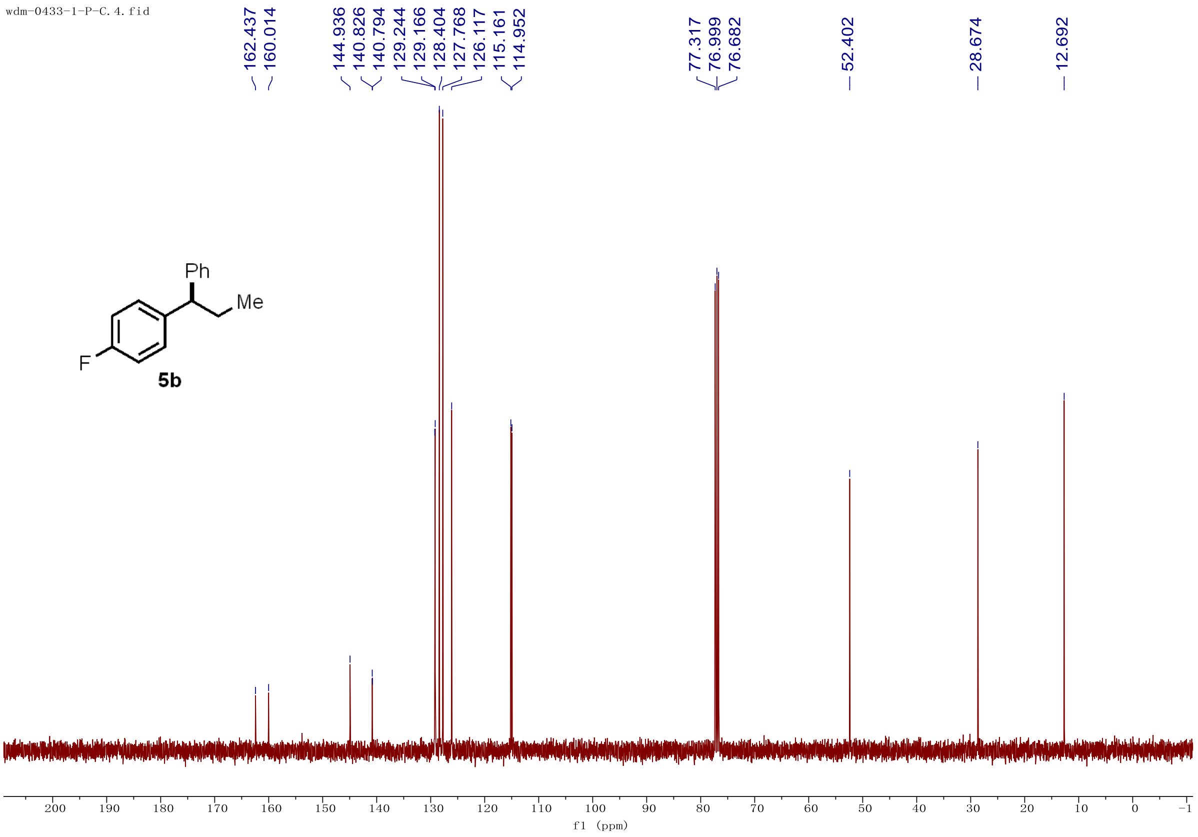

Supplementary Figure 108. ^{13}C NMR (100 MHz, $CDCl_3$) spectrum of 5b

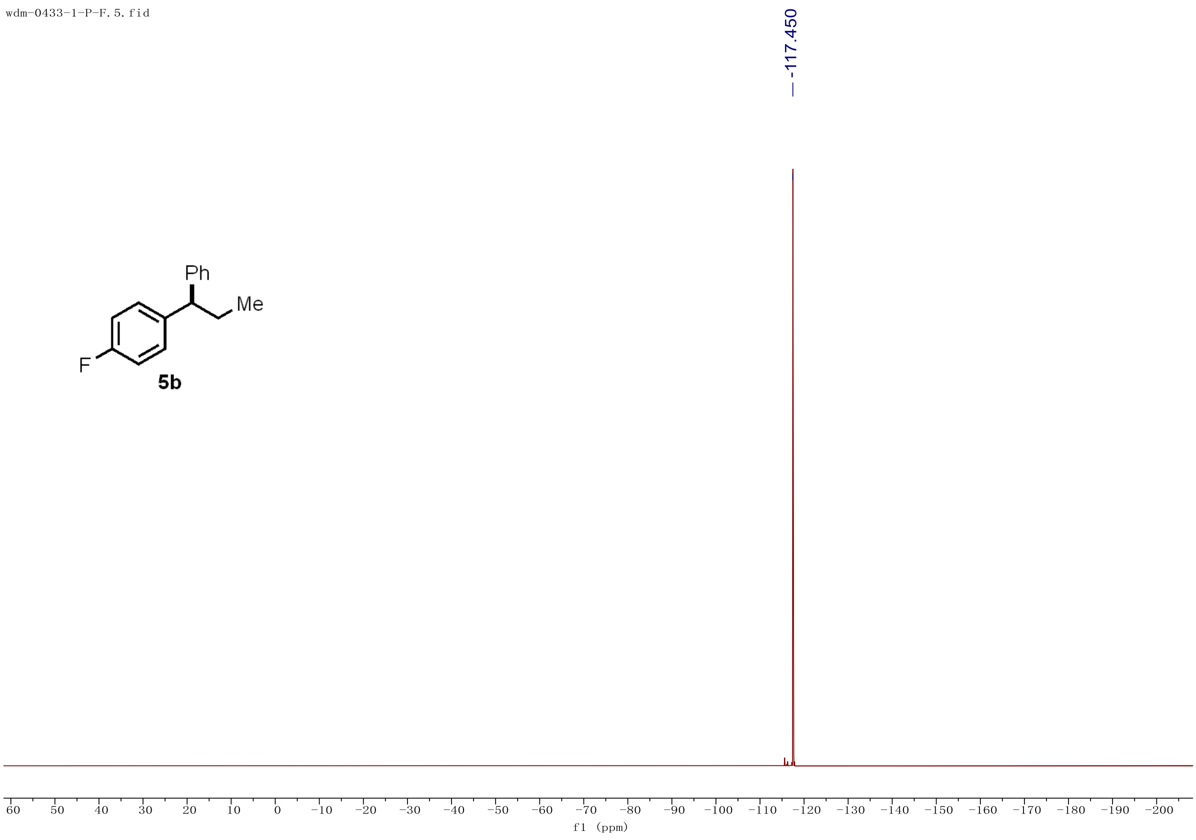

Supplementary Figure 109. ^{17}F NMR (375 MHz, CDCl₃) spectrum of 5b

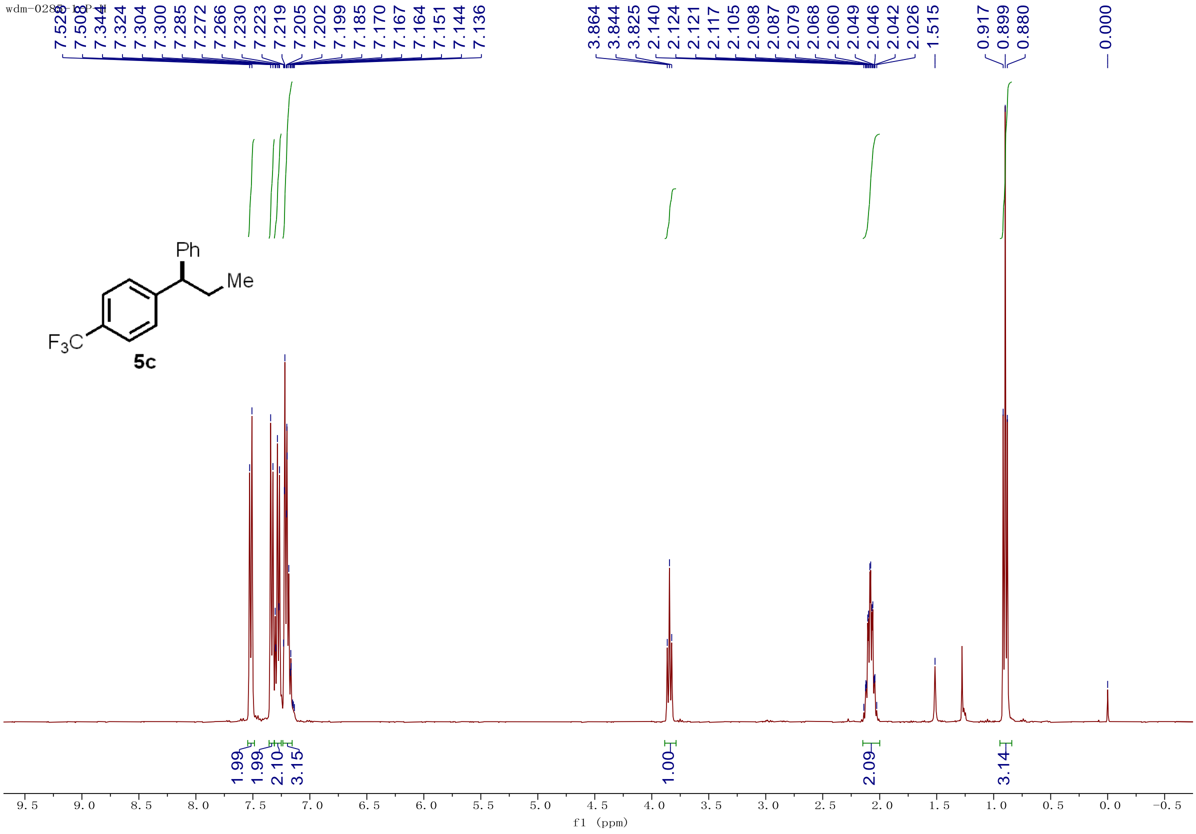

Supplementary Figure 110. ^{1}H NMR (400 MHz, CDCl₃) spectrum of 5c

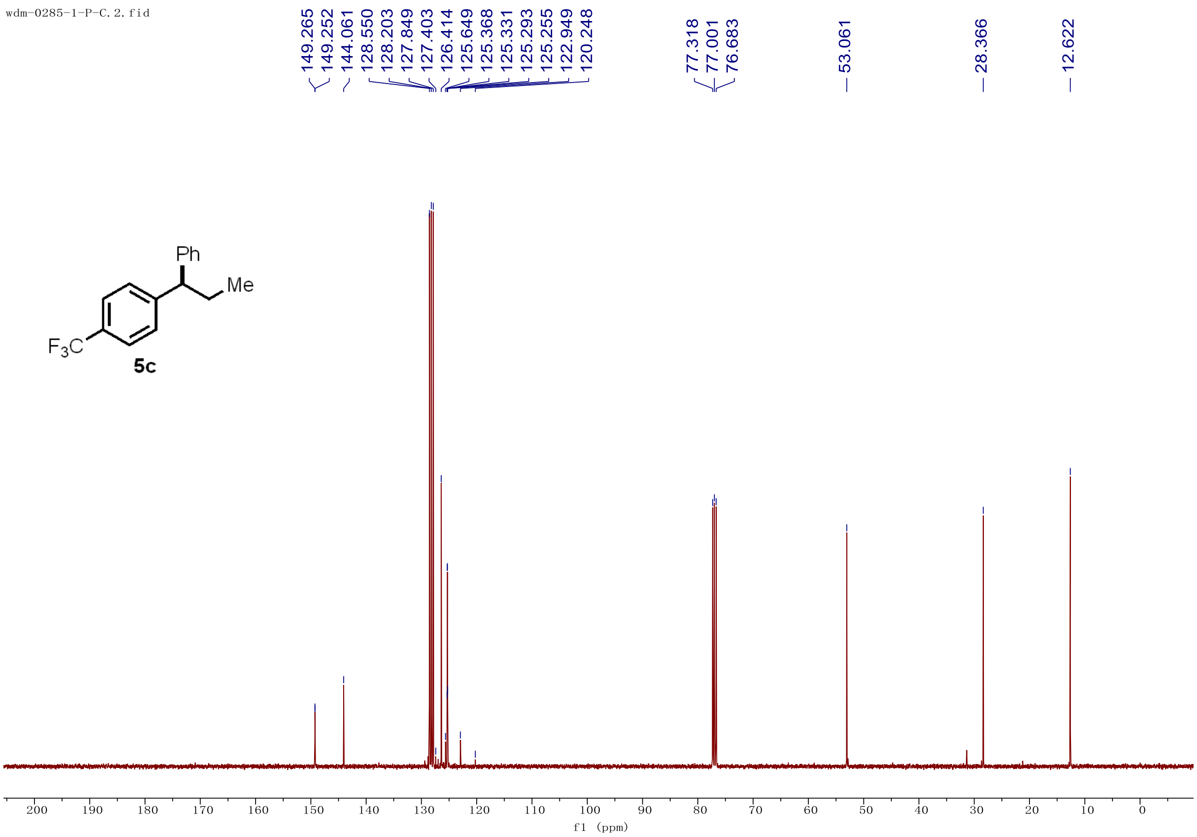

Supplementary Figure 111. ^{13}C **NMR (100 MHz, CDCl3) spectrum of 5c**

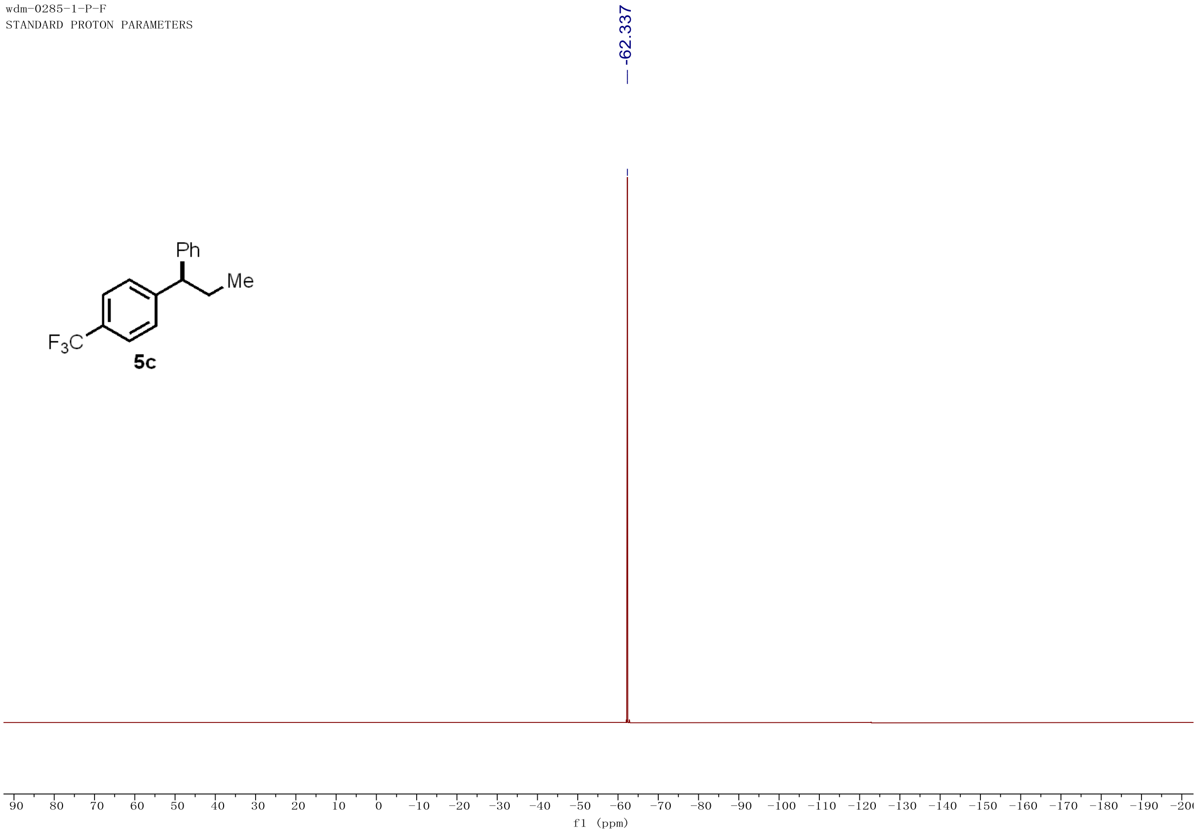

Supplementary Figure 112. ^{19}F **NMR (375 MHz, CDCl3) spectrum of 5c**

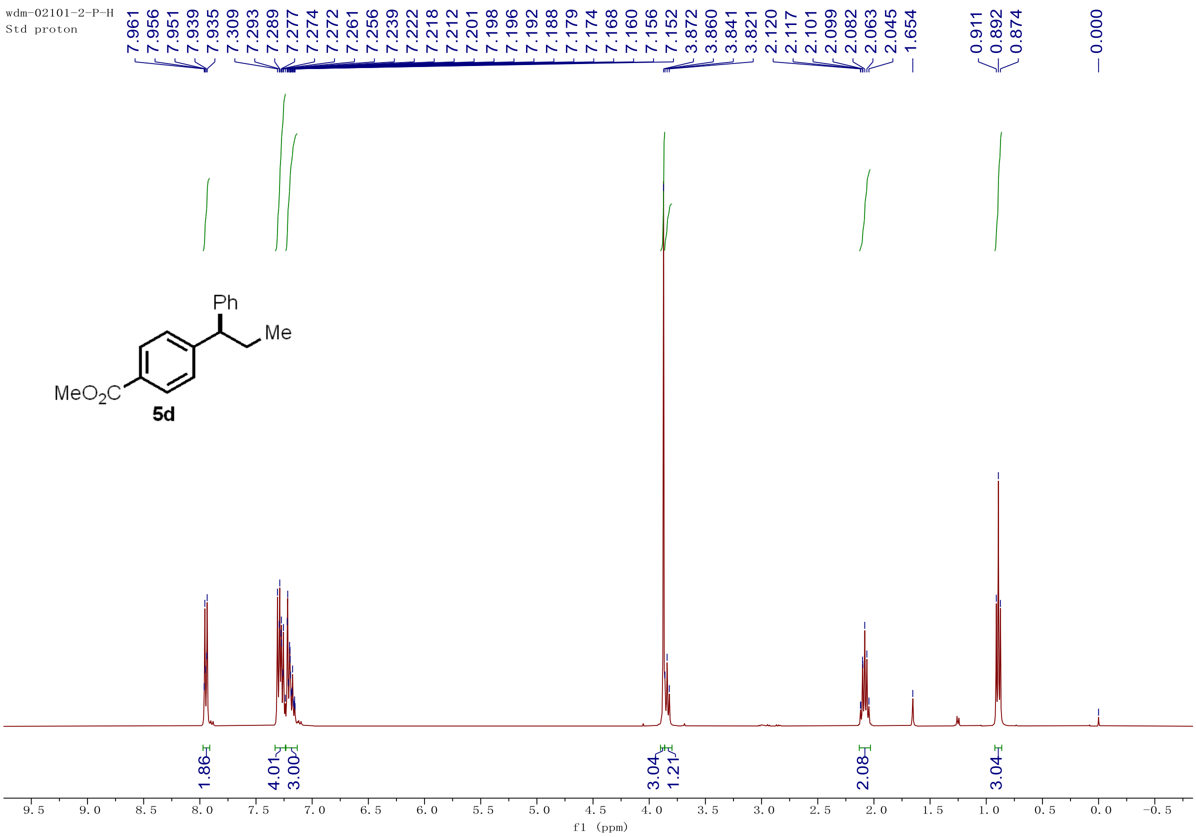

Supplementary Figure 113. ^{1}H **NMR (400 MHz,** $CDCl_3$**) spectrum of 5d**

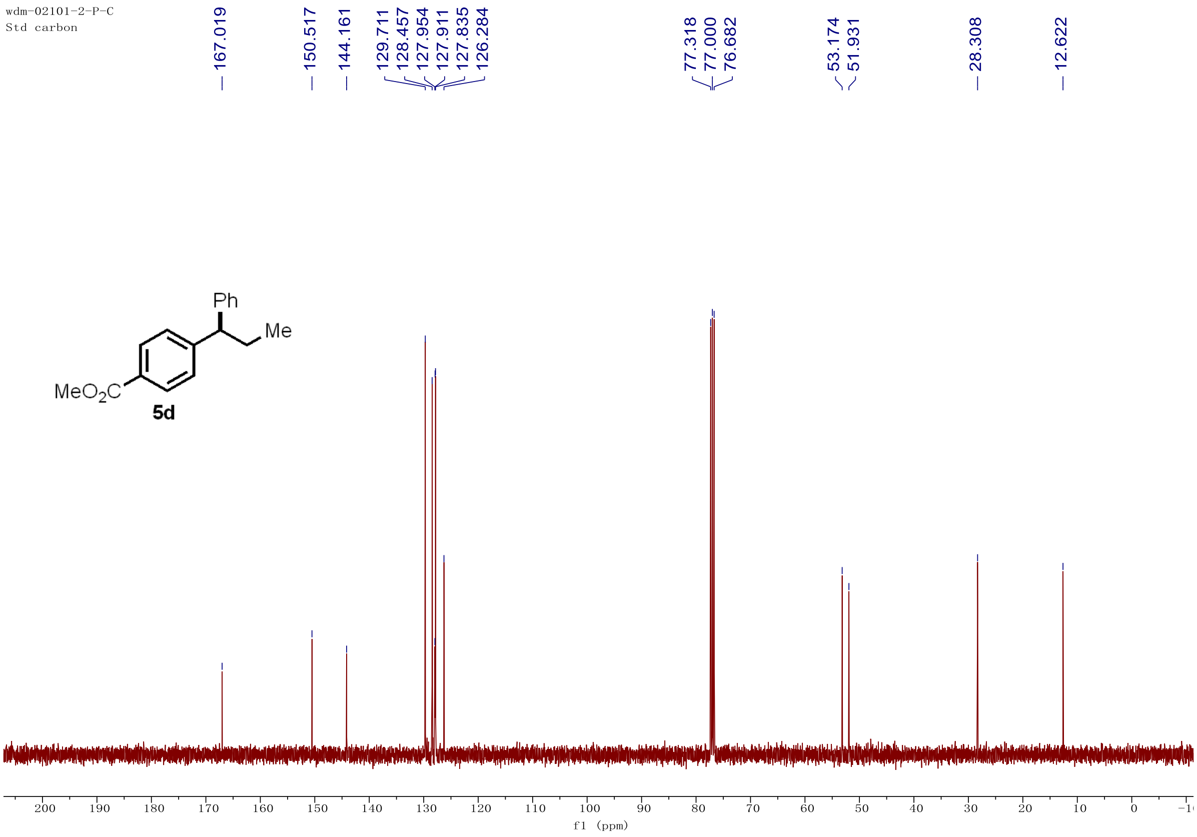

Supplementary Figure 114. ^{13}C **NMR (100 MHz,** $CDCl_3$**) spectrum of 5d**

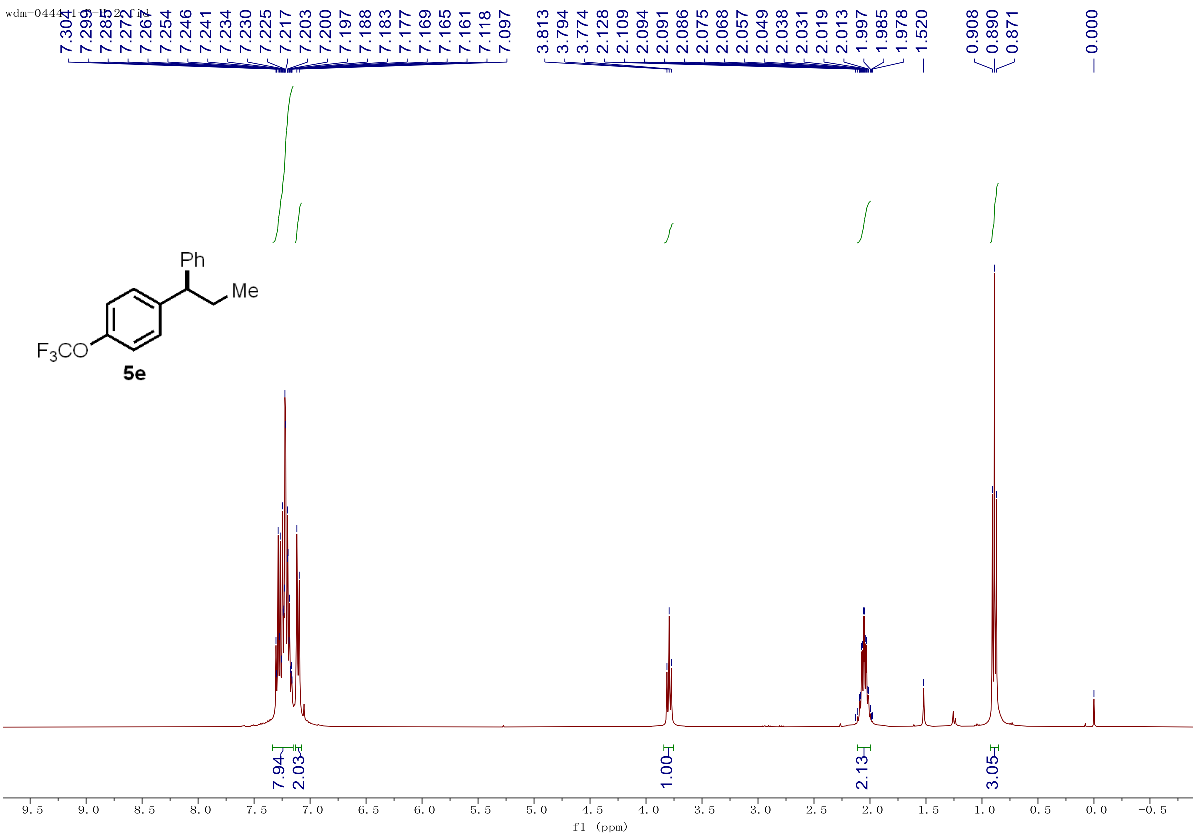

Supplementary Figure 115. ^{1}H NMR (400 MHz, CDCl₃) spectrum of 5e

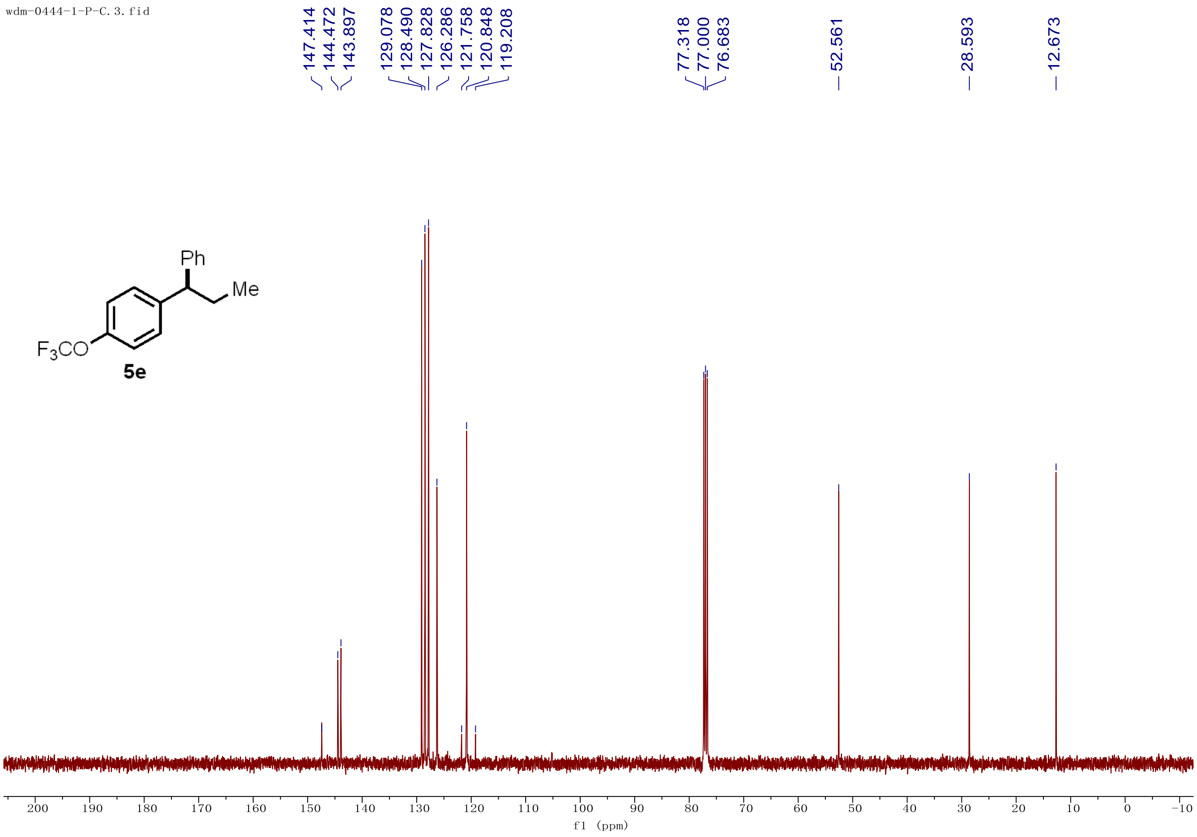

Supplementary Figure 116. ^{13}C NMR (100 MHz, CDCl₃) spectrum of 5e

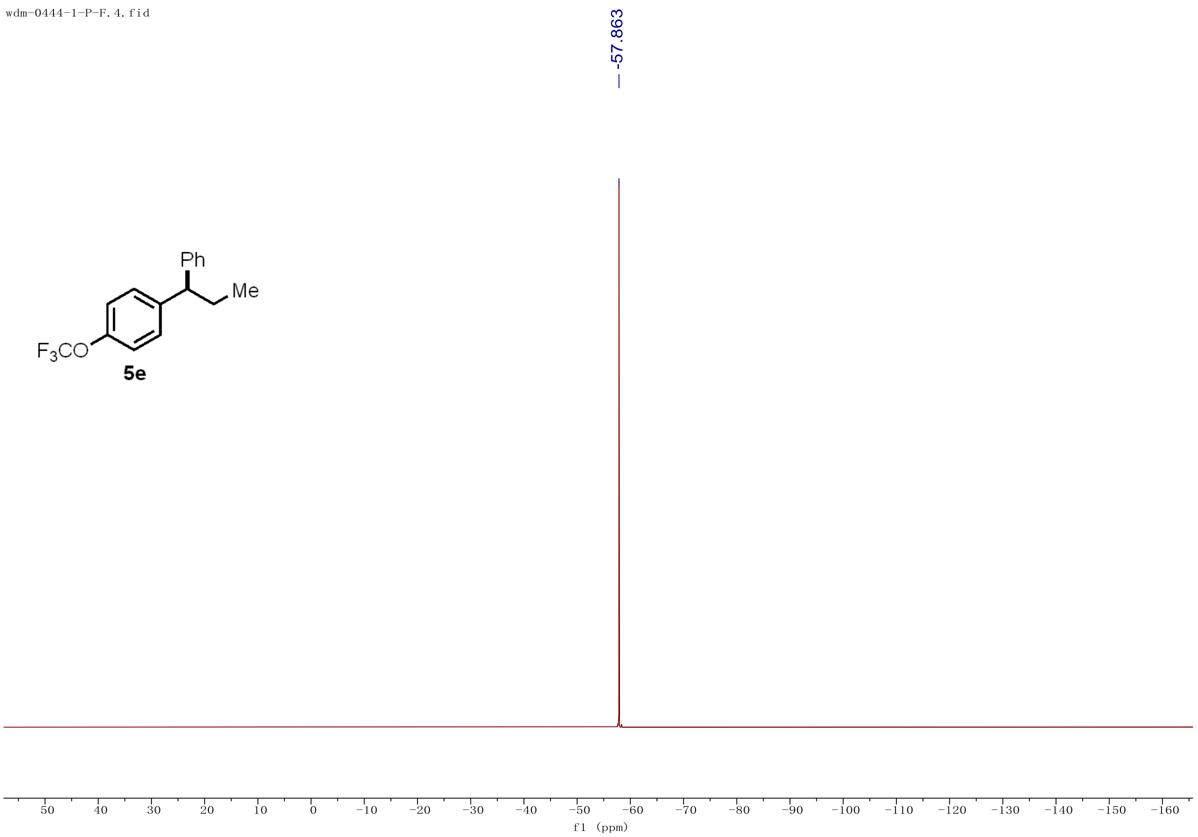

Supplementary Figure 117. ^{19}F NMR (375 MHz, CDCl₃) spectrum of 5e

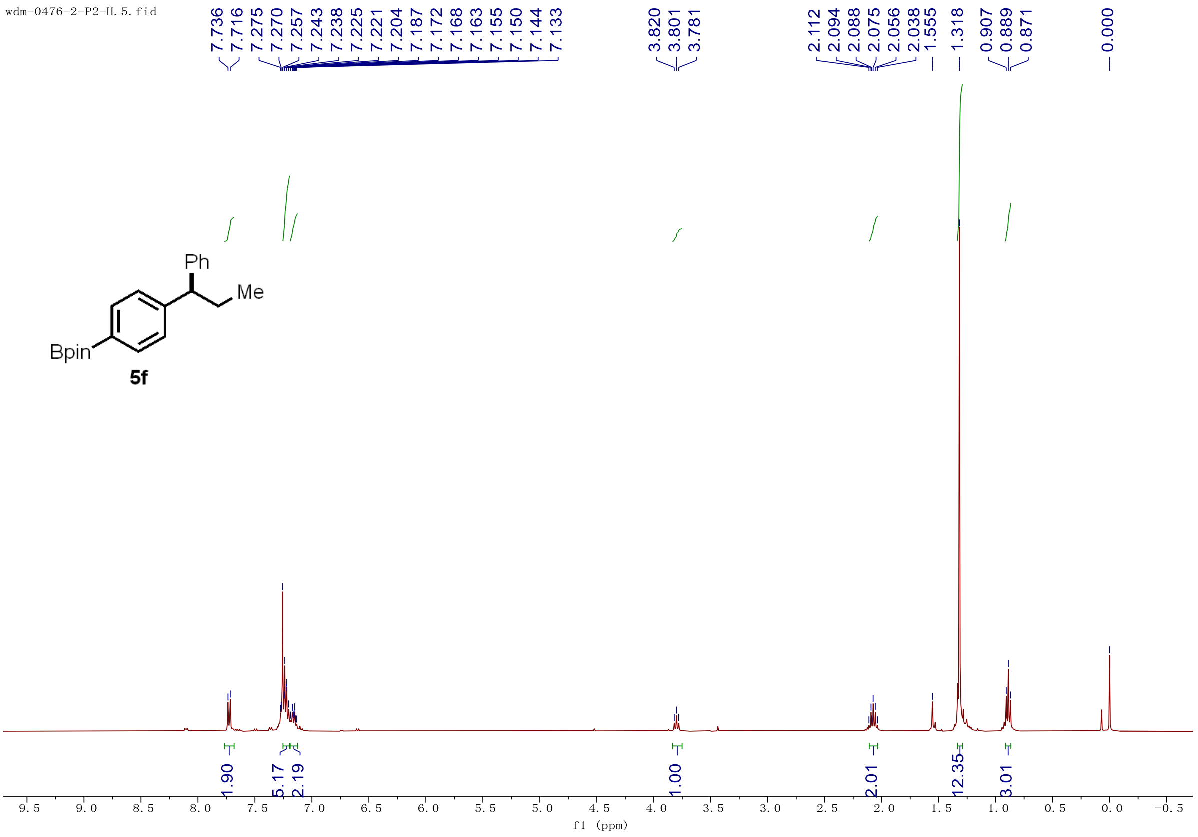

Supplementary Figure 118. ^{1}H NMR (400 MHz, CDCl₃) spectrum of 5f

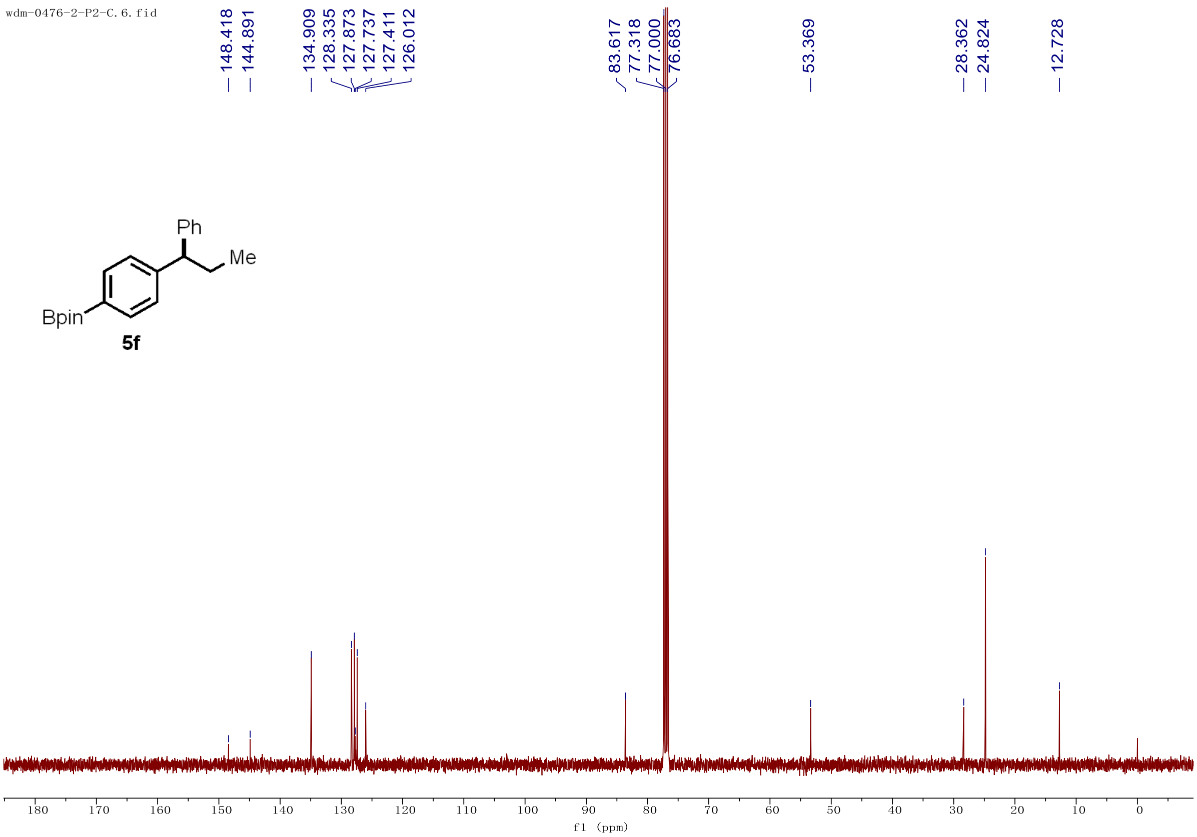

Supplementary Figure 119. ^{13}C NMR (100 MHz, CDCl$_3$) spectrum of 5f

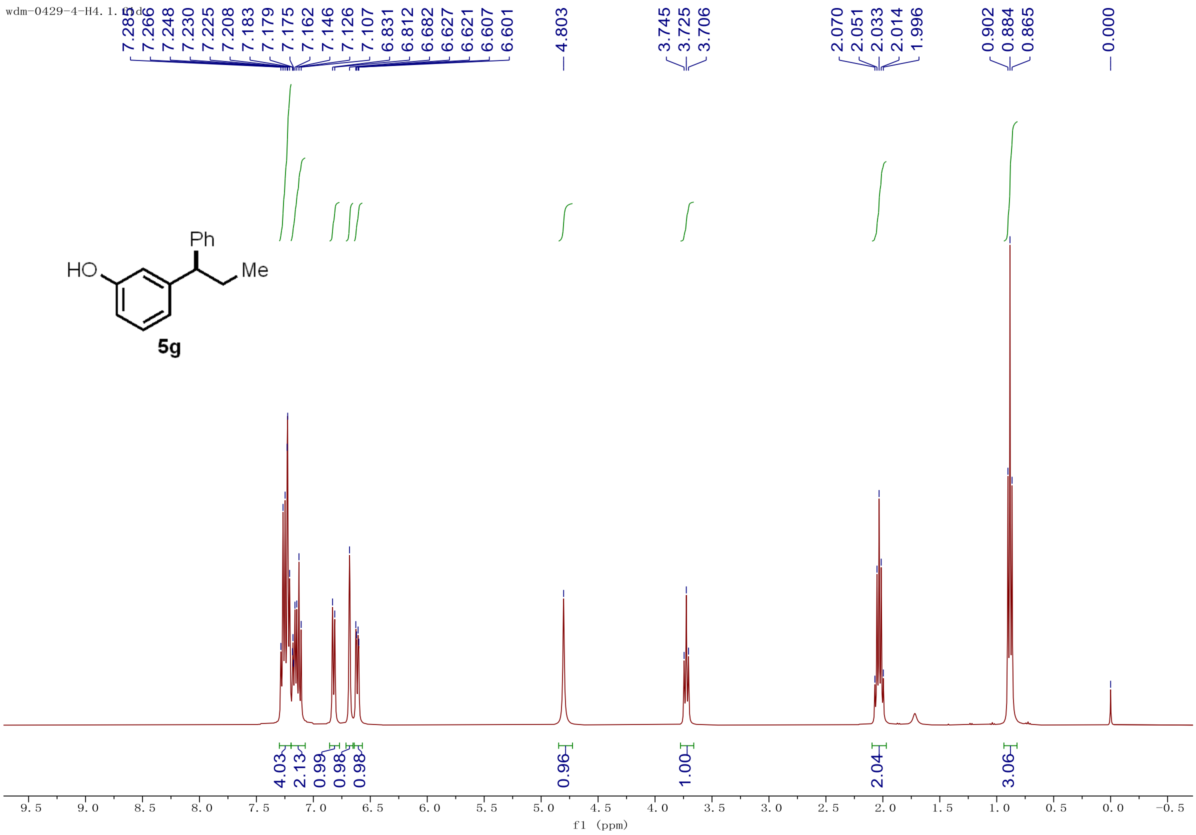

Supplementary Figure 120. ^{1}H NMR (400 MHz, CDCl$_3$) spectrum of 5g

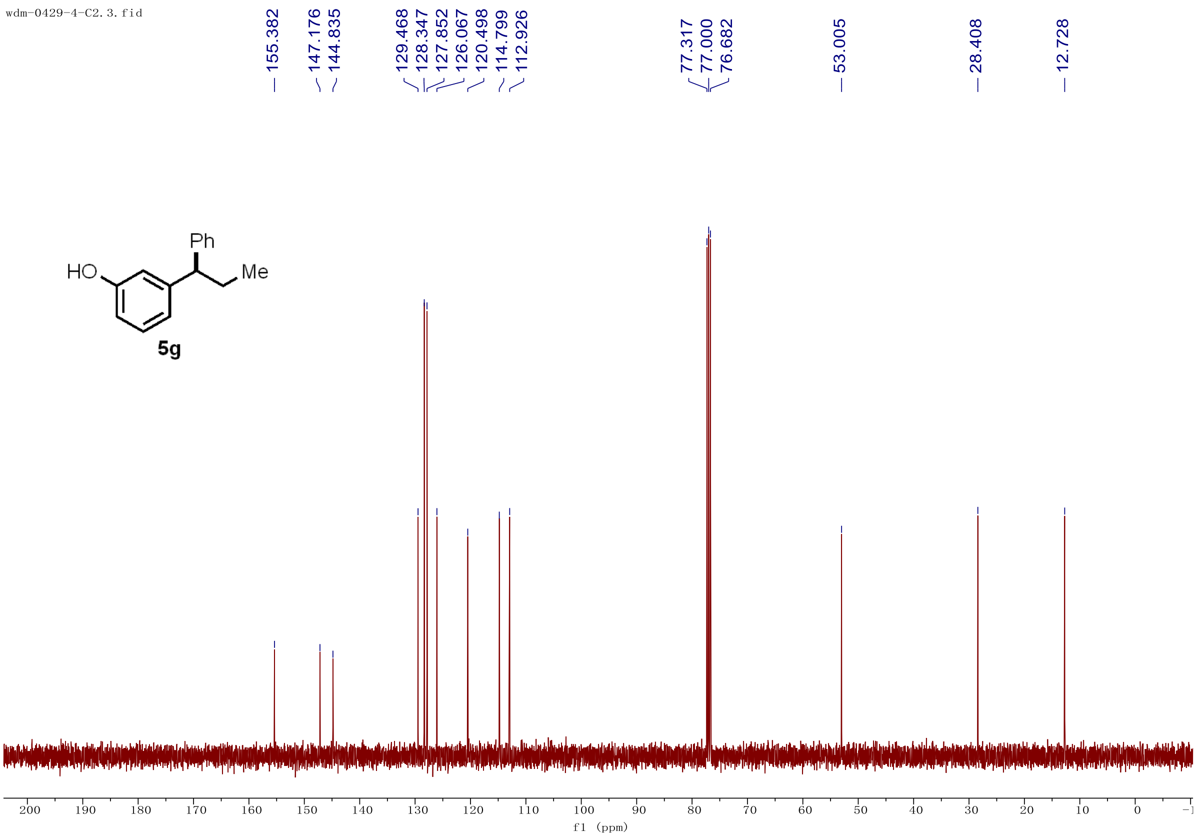

Supplementary Figure 121. ^{13}C NMR (100 MHz, CDCl$_3$) spectrum of 5g

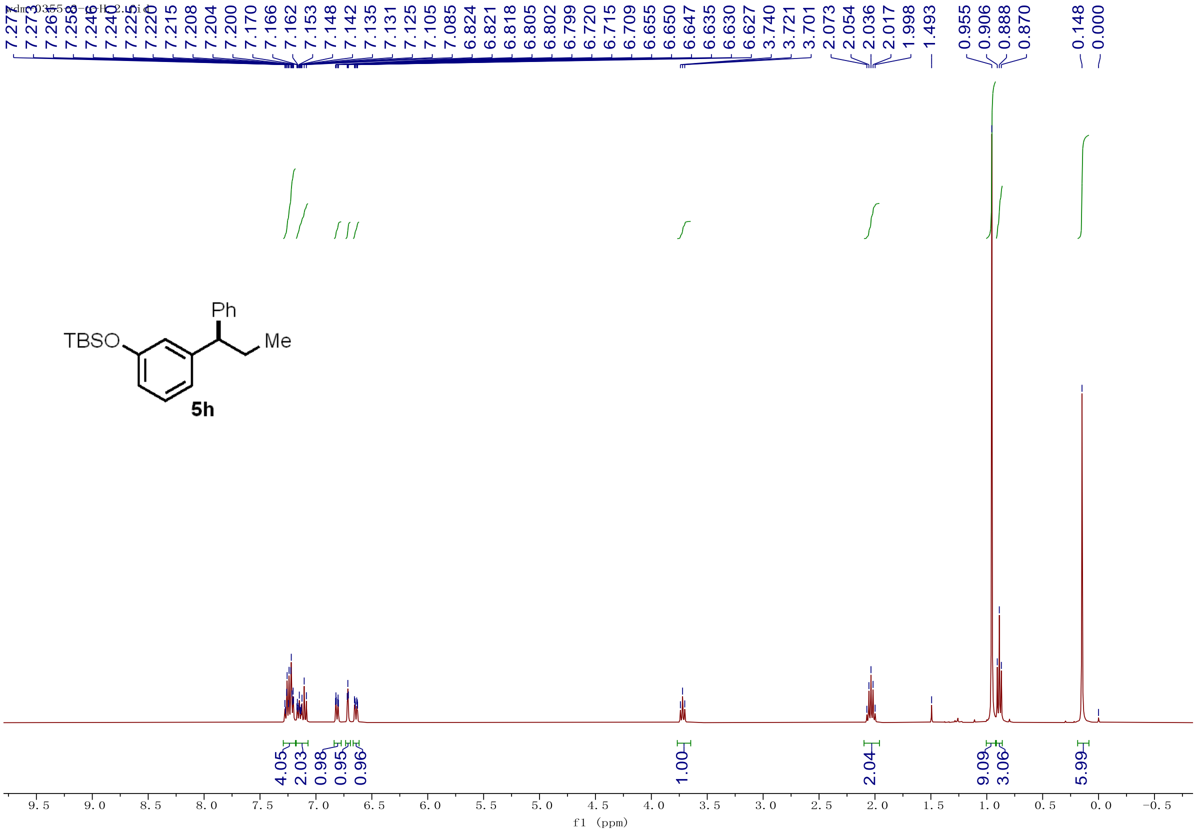

Supplementary Figure 122. ^{1}H NMR (400 MHz, CDCl$_3$) spectrum of 5h

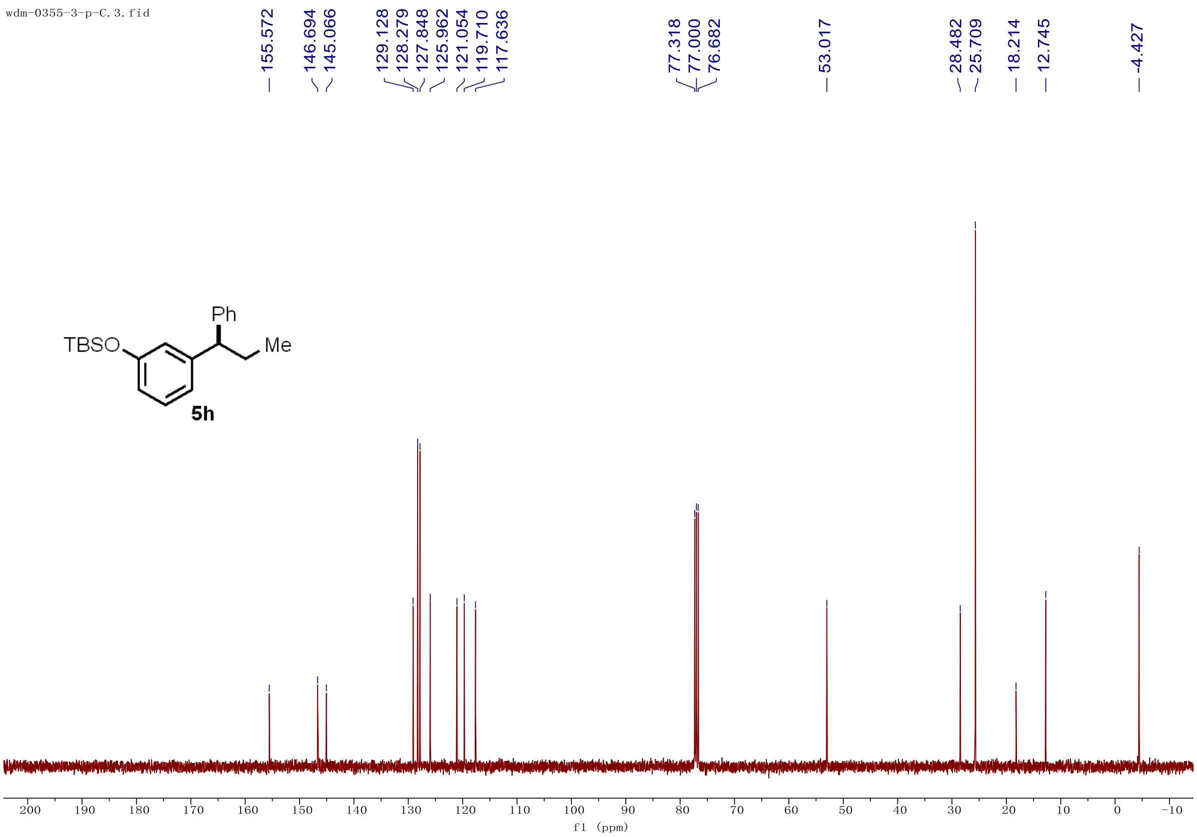

Supplementary Figure 123. ^{13}C NMR (100 MHz, $CDCl_3$) spectrum of 5h

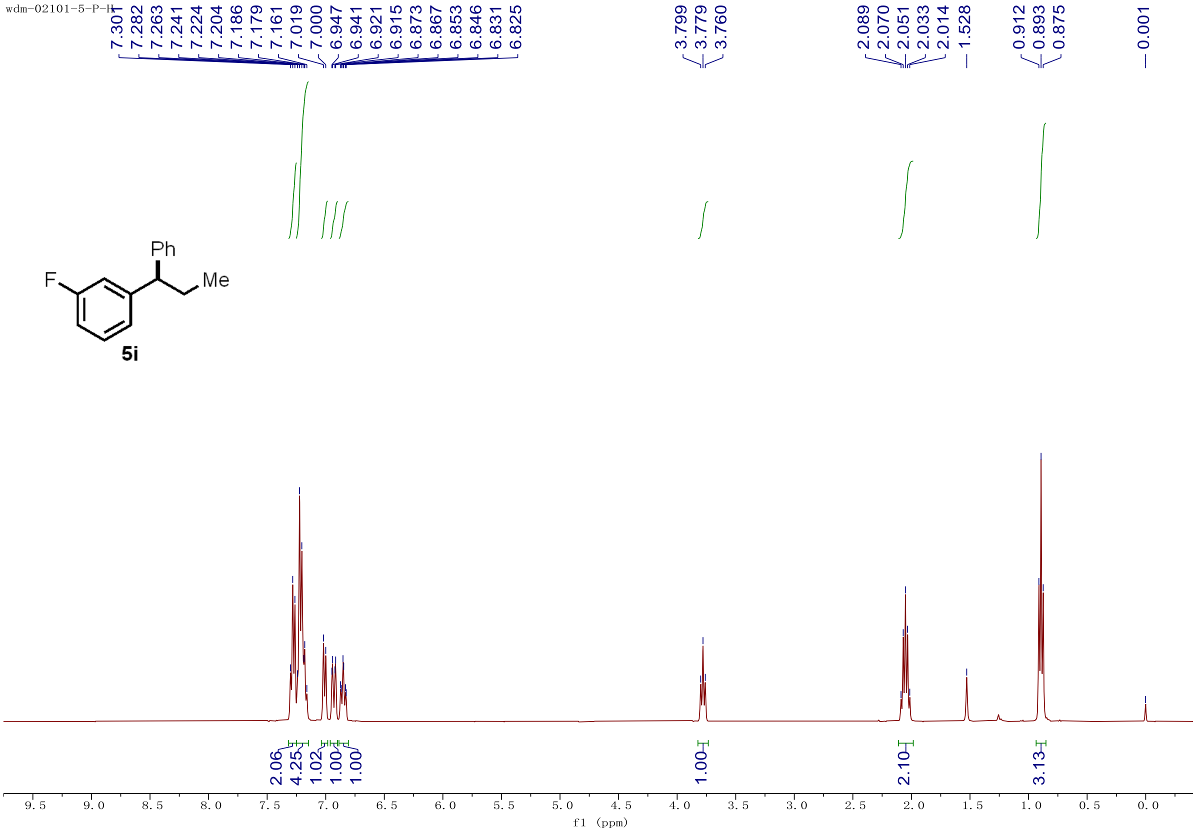

Supplementary Figure 124. ^{1}H NMR (400 MHz, $CDCl_3$) spectrum of 5i

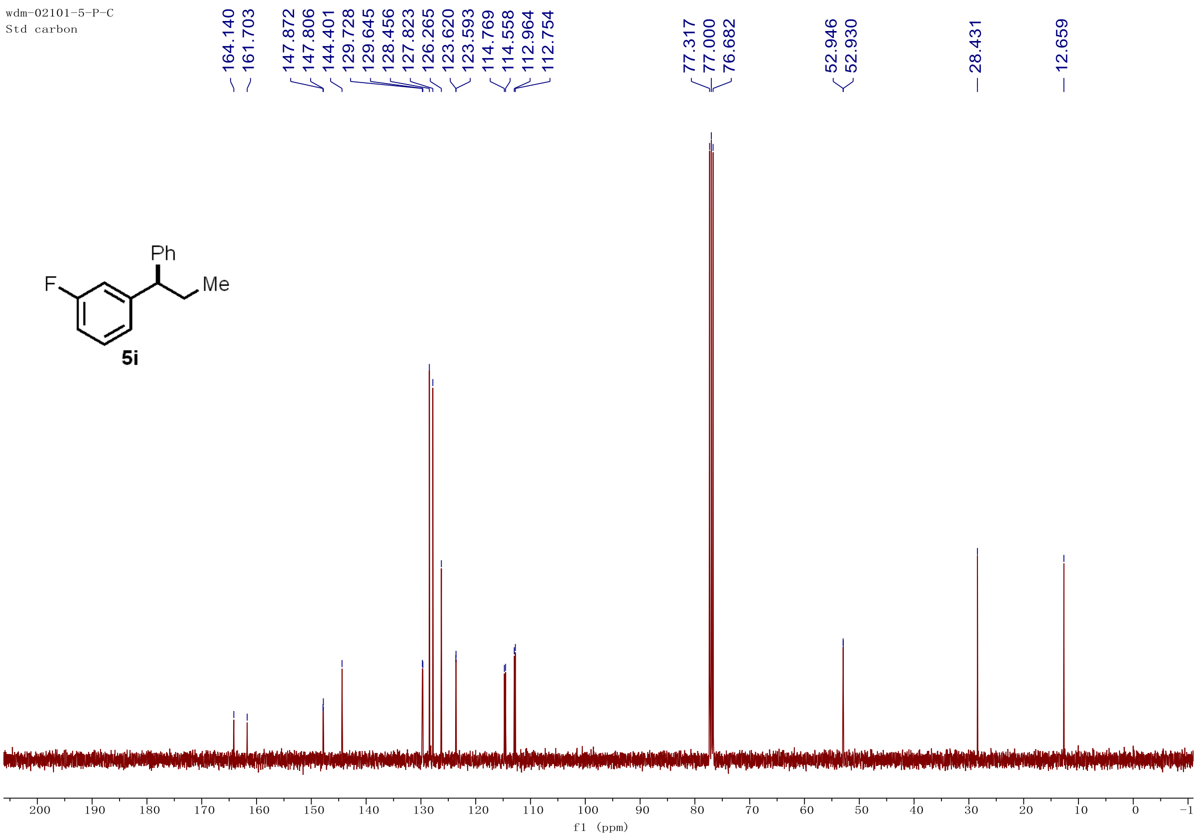

Supplementary Figure 125. ^{13}**C NMR (100 MHz, CDCl**$_3$**) spectrum of 5i**

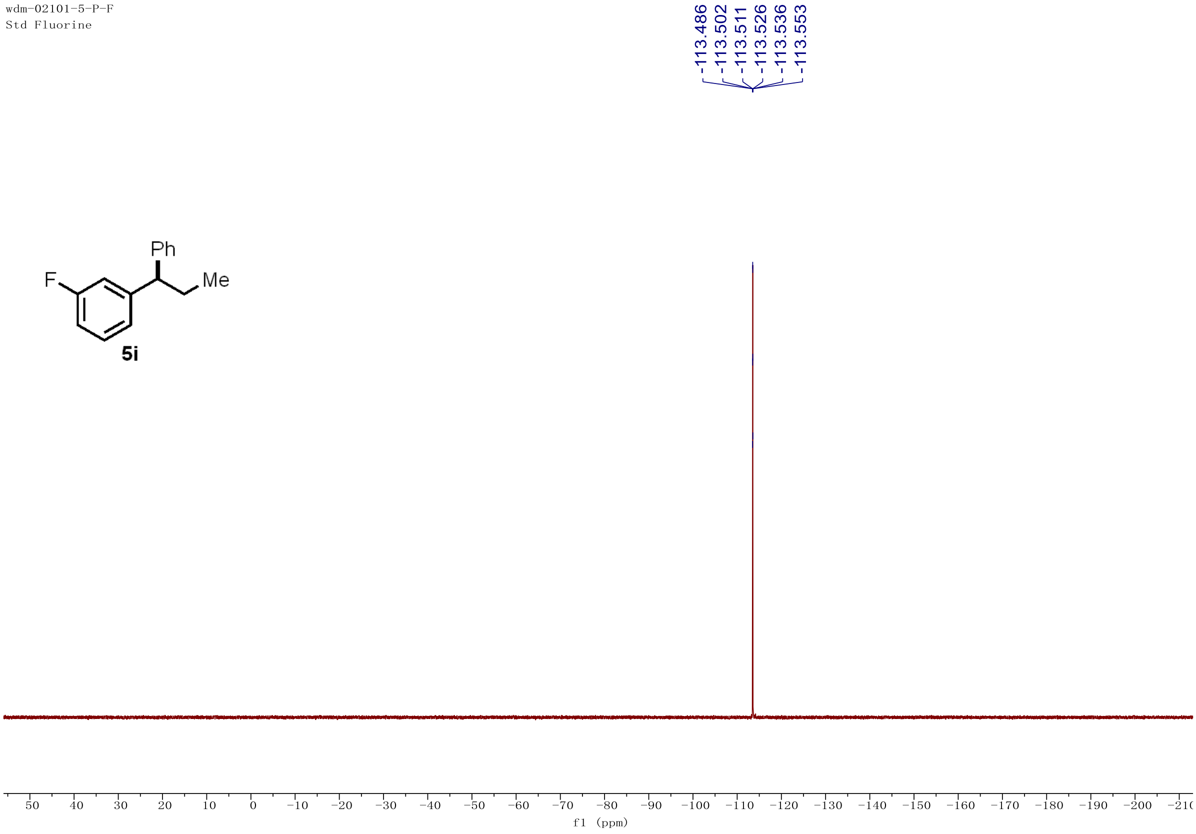

Supplementary Figure 126. ^{19}**F NMR (375 MHz, CDCl**$_3$**) spectrum of 5i**

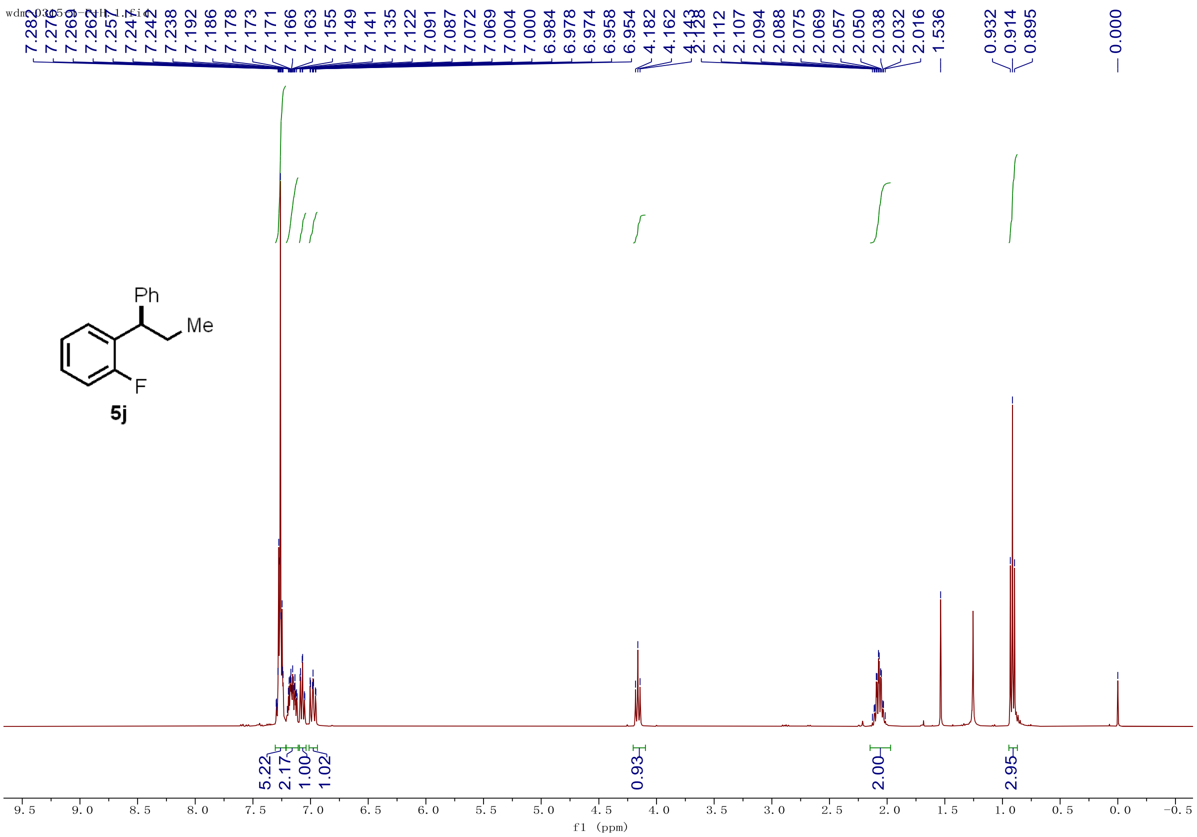

Supplementary Figure 127. ^{1}H NMR (400 MHz, $CDCl_3$) spectrum of 5j

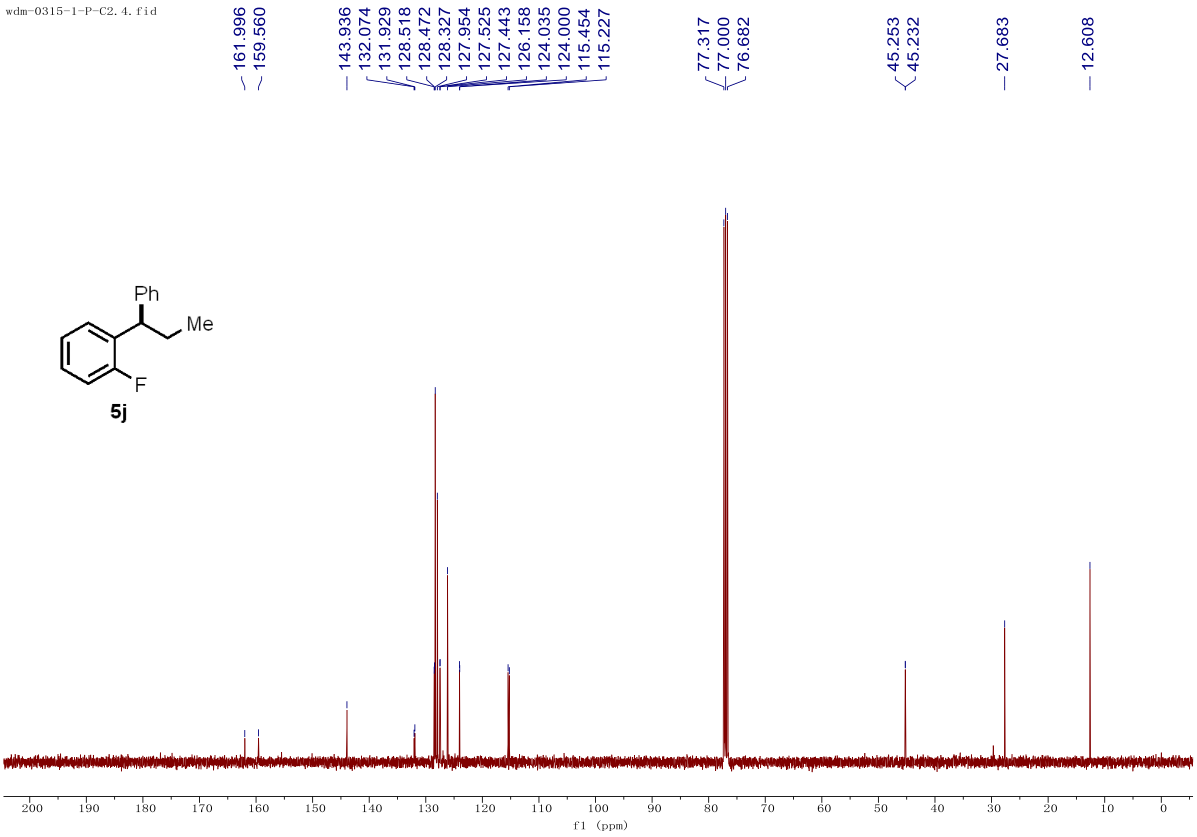

Supplementary Figure 128. ^{13}C NMR (100 MHz, $CDCl_3$) spectrum of 5j

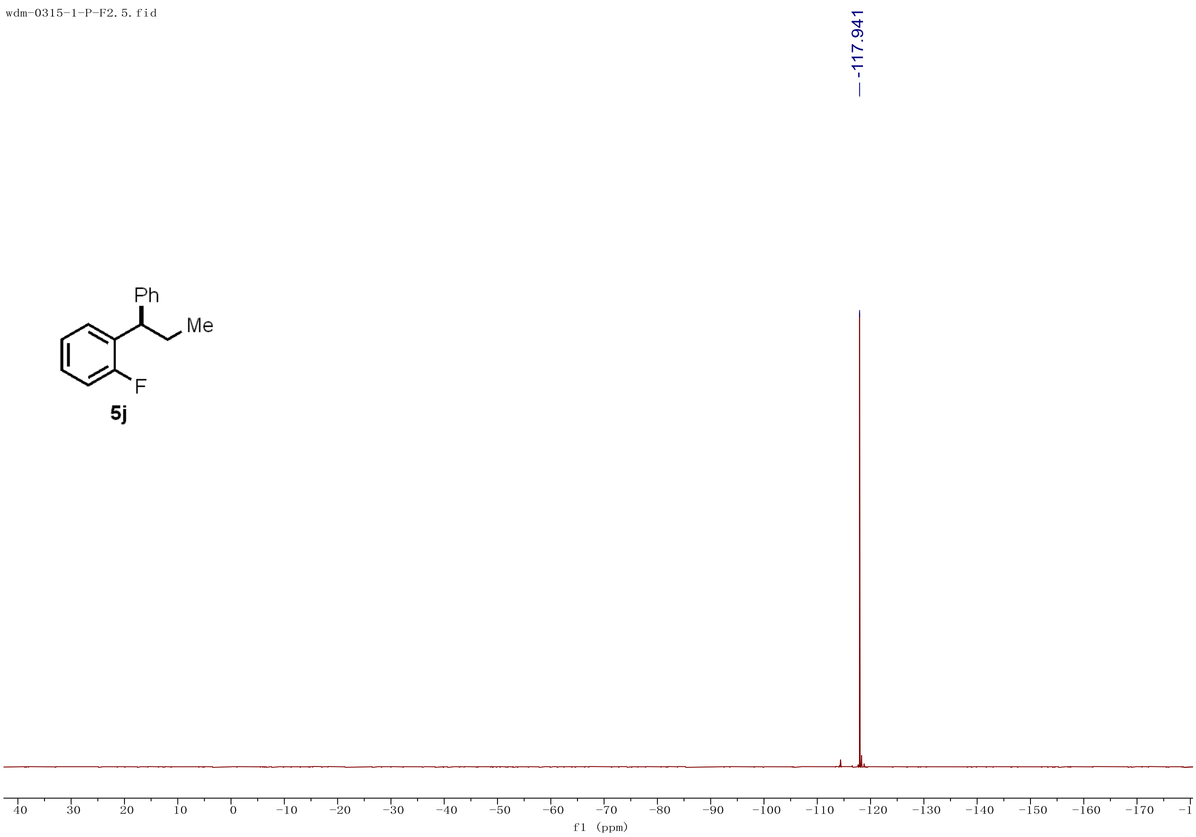

Supplementary Figure 129. ^{19}F NMR (375 MHz, CDCl$_3$) spectrum of 5j

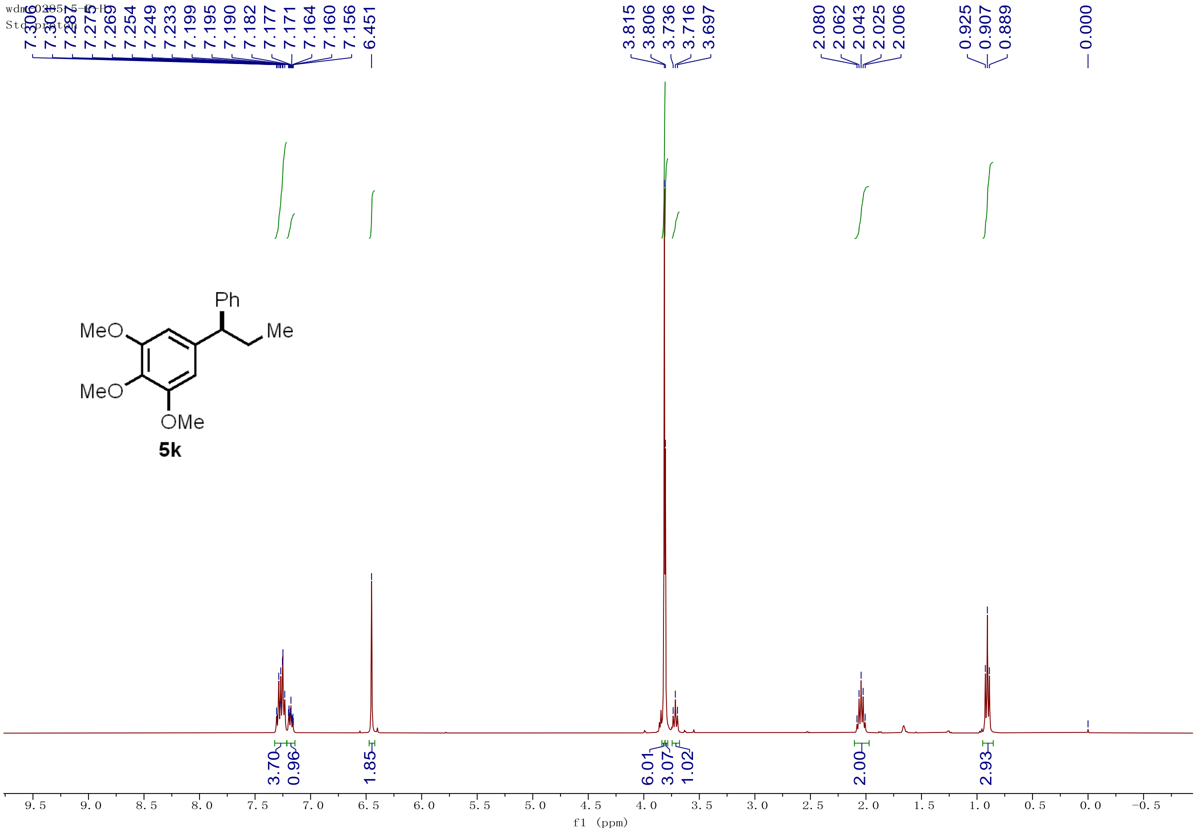

Supplementary Figure 130. ^{1}H NMR (400 MHz, CDCl$_3$) spectrum of 5k

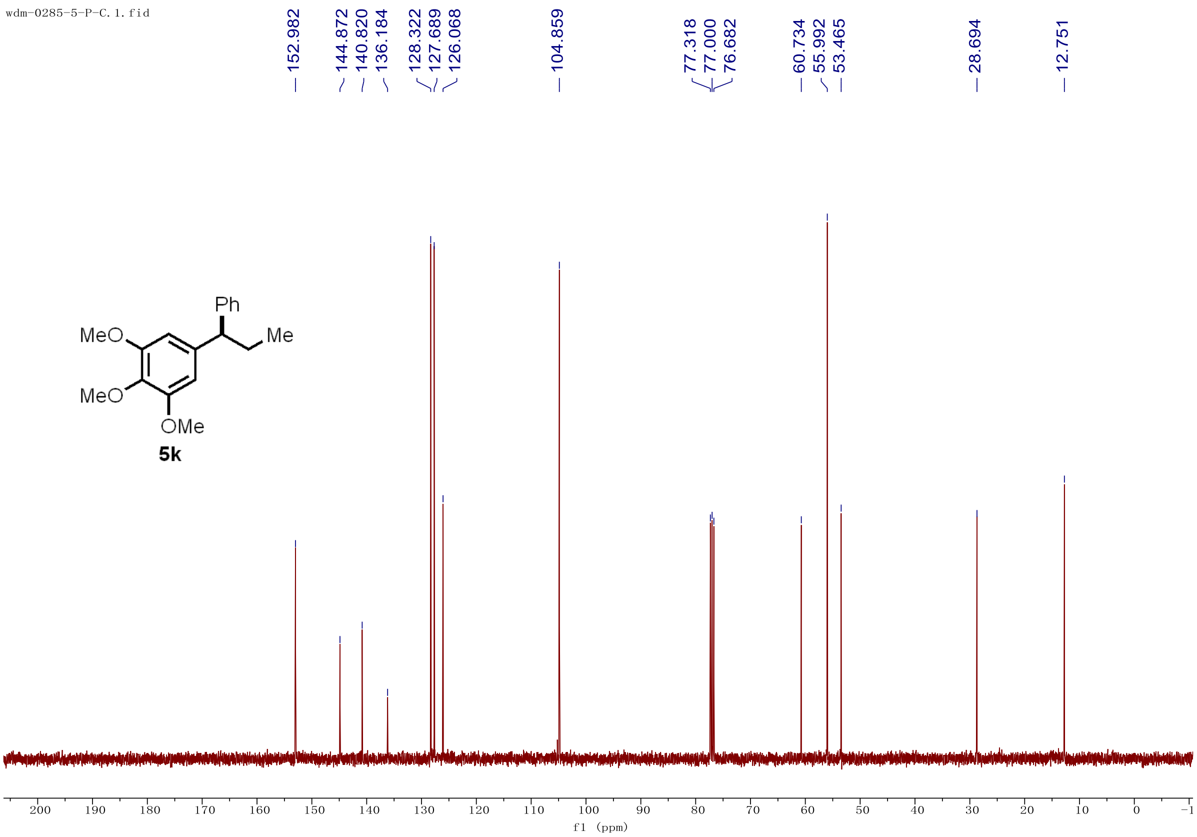

Supplementary Figure 131. ^{13}C NMR (100 MHz, CDCl$_3$) spectrum of 5k

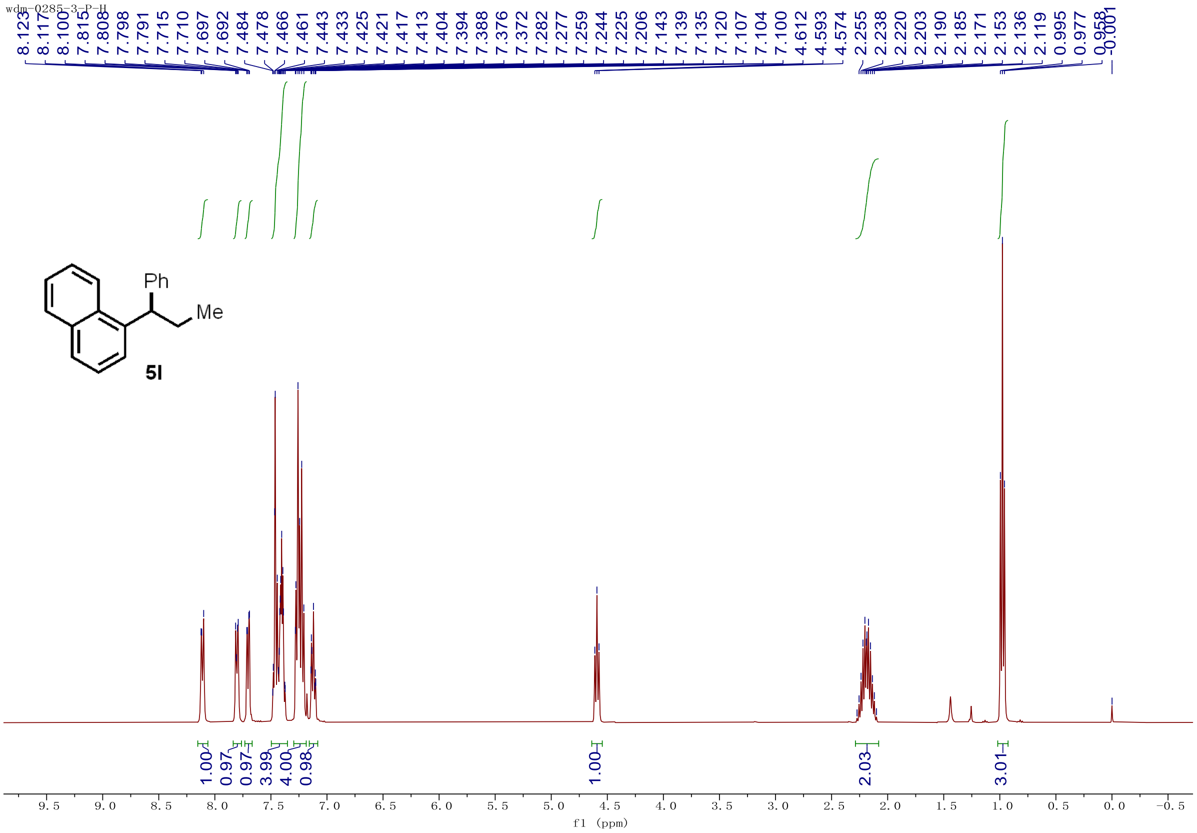

Supplementary Figure 132. ^{1}H NMR (400 MHz, CDCl$_3$) spectrum of 5l

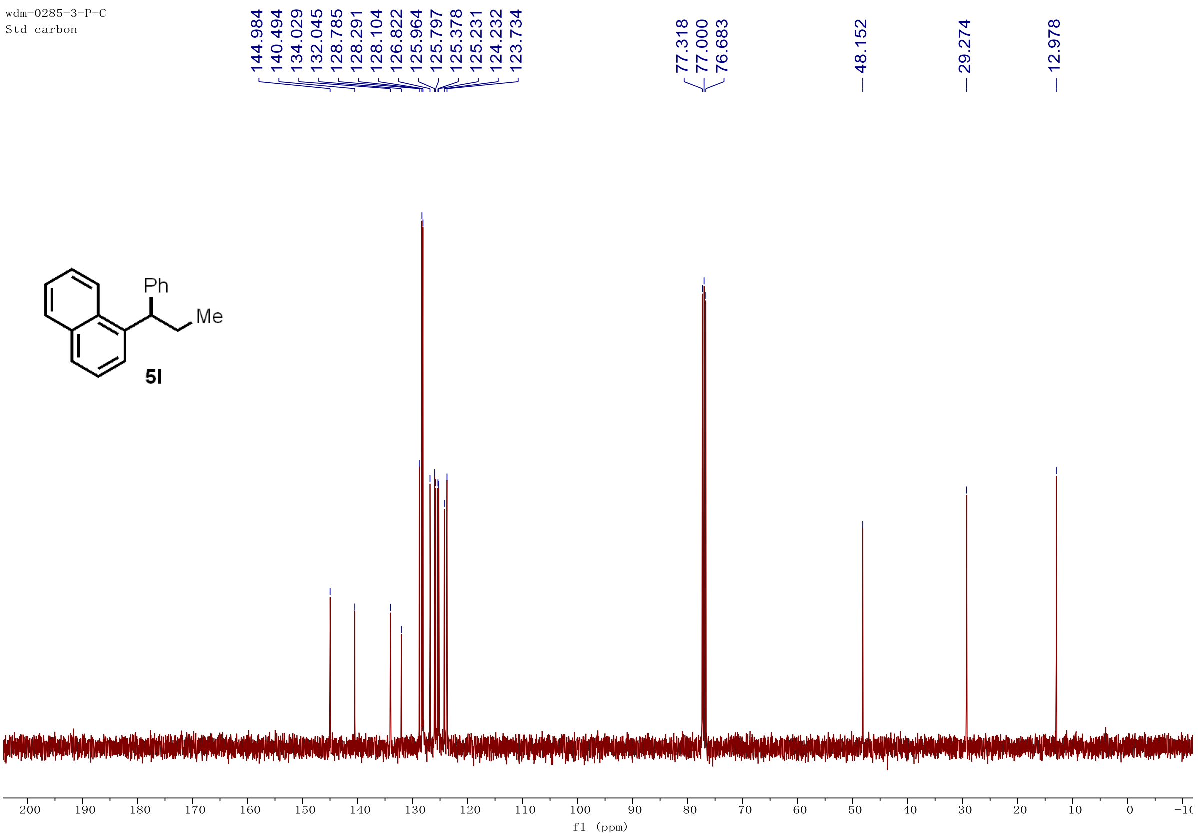

Supplementary Figure 133. ^{13}**C NMR (100 MHz, CDCl$_3$) spectrum of 5l**

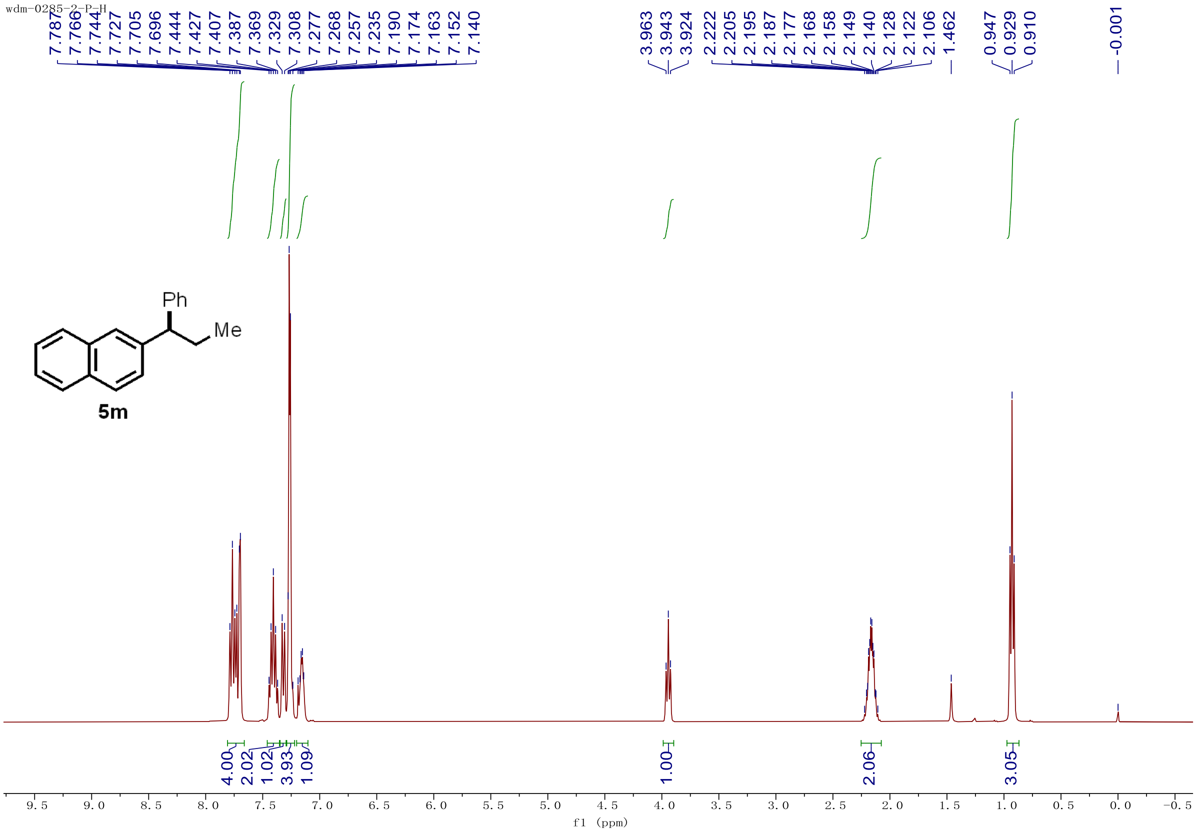

Supplementary Figure 134. ^{1}**H NMR (400 MHz, CDCl$_3$) spectrum of 5m**

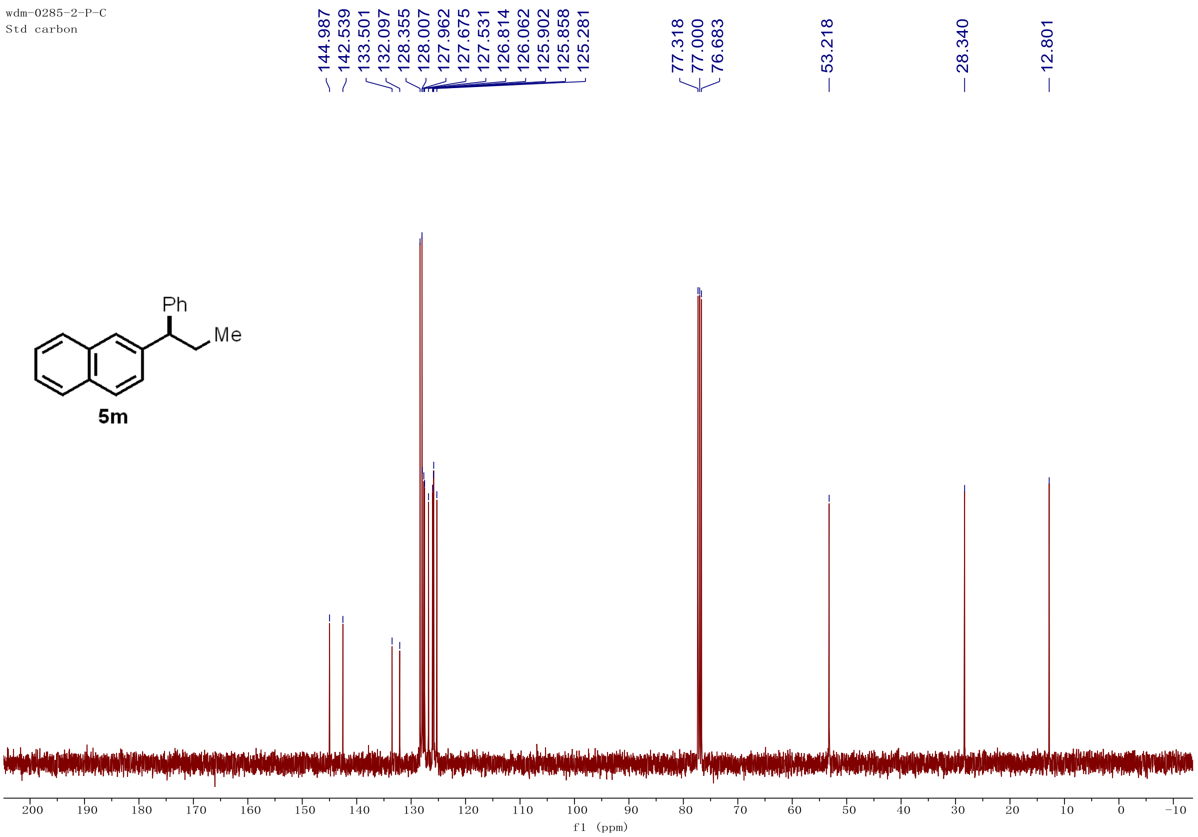

Supplementary Figure 135. ^{13}C NMR (100 MHz, CDCl$_3$) spectrum of 5m

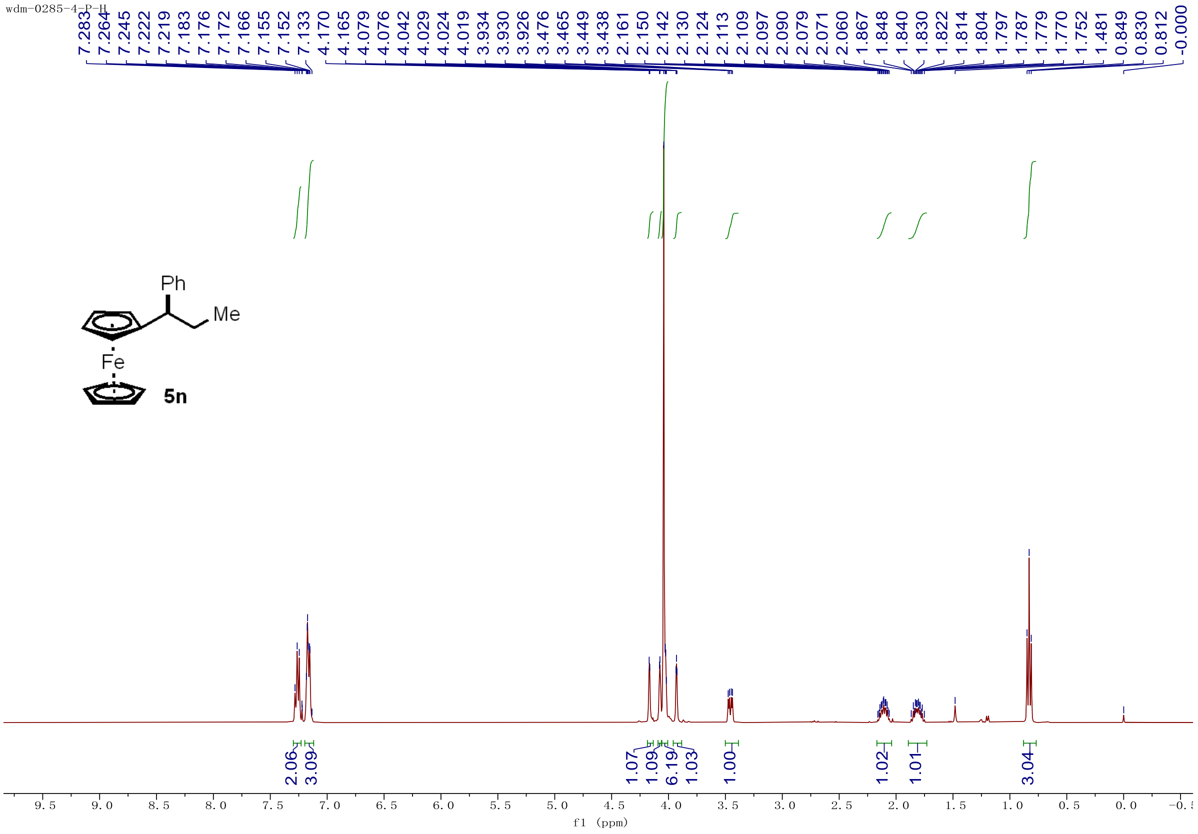

Supplementary Figure 136. ^{1}H NMR (400 MHz, CDCl$_3$) spectrum of 5n

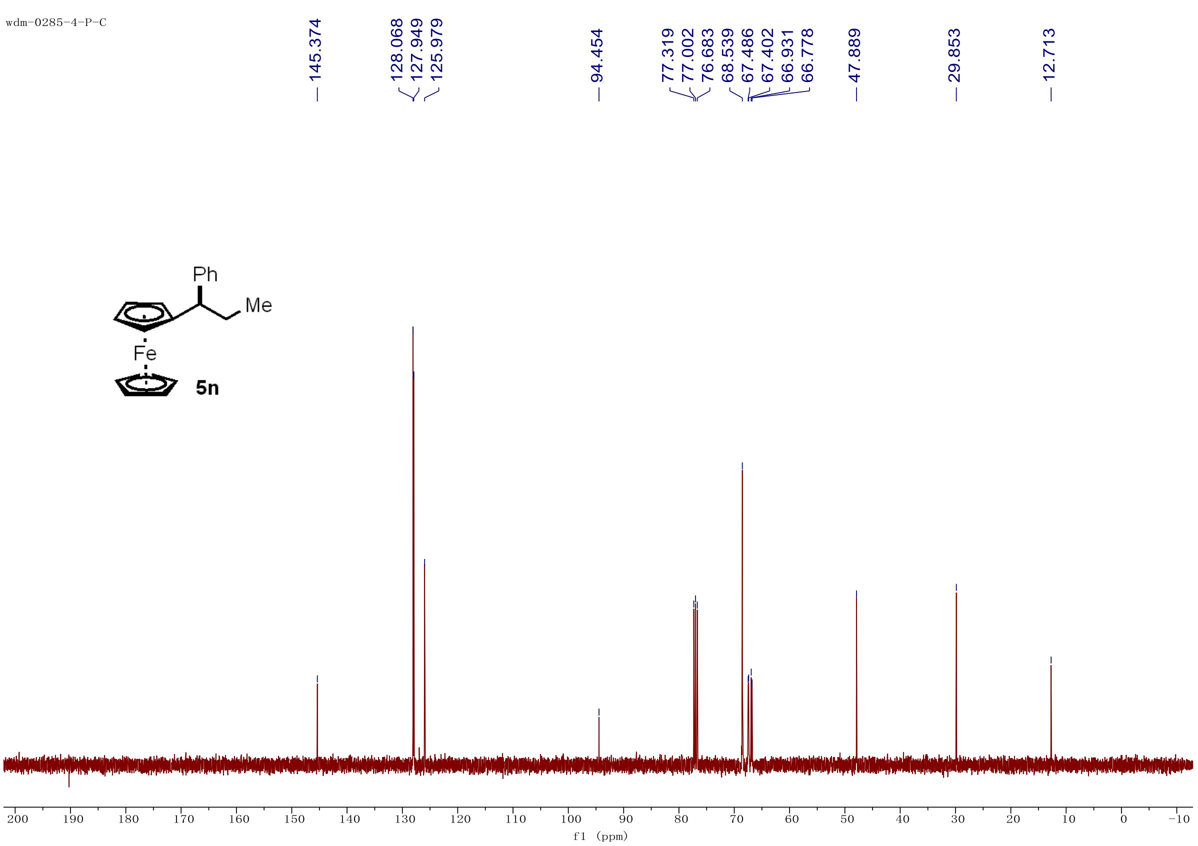

Supplementary Figure 137. ^{13}**C NMR (100 MHz, CDCl$_3$) spectrum of 5n**

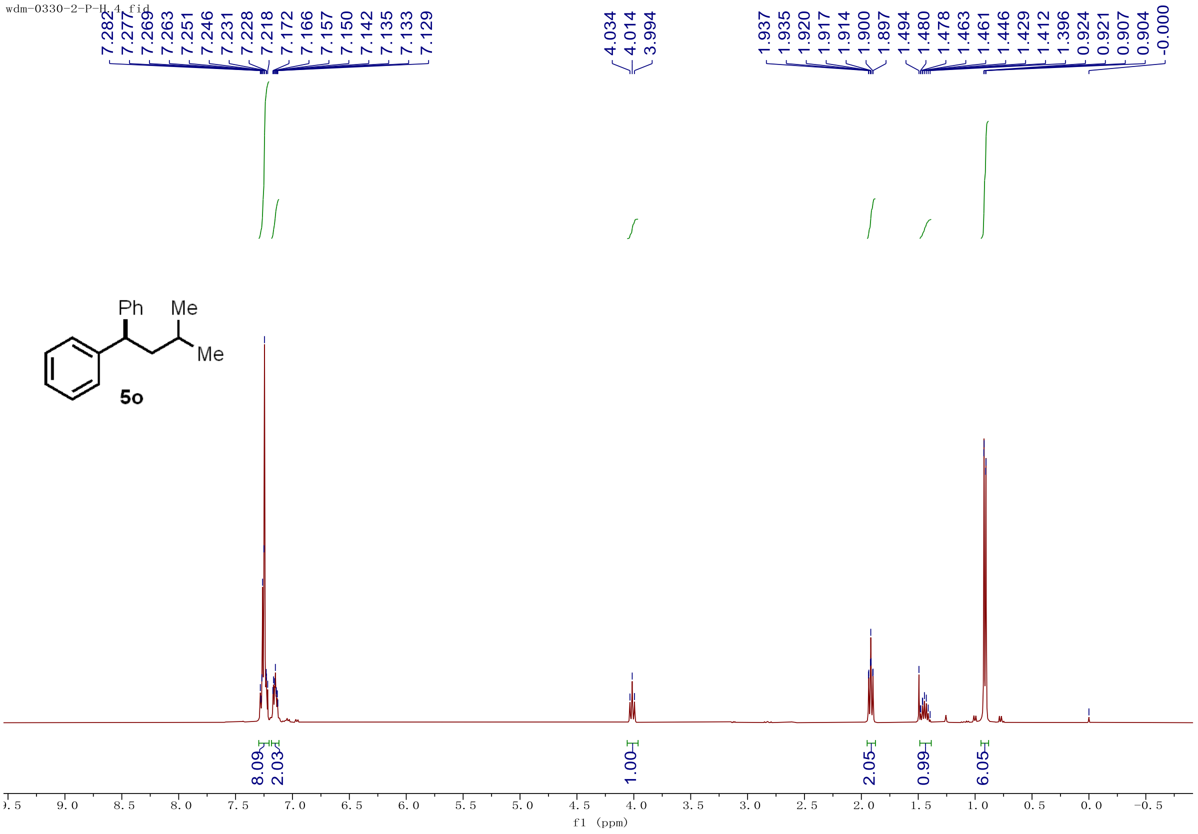

Supplementary Figure 138. ^{1}**H NMR (400 MHz, CDCl$_3$) spectrum of 5o**

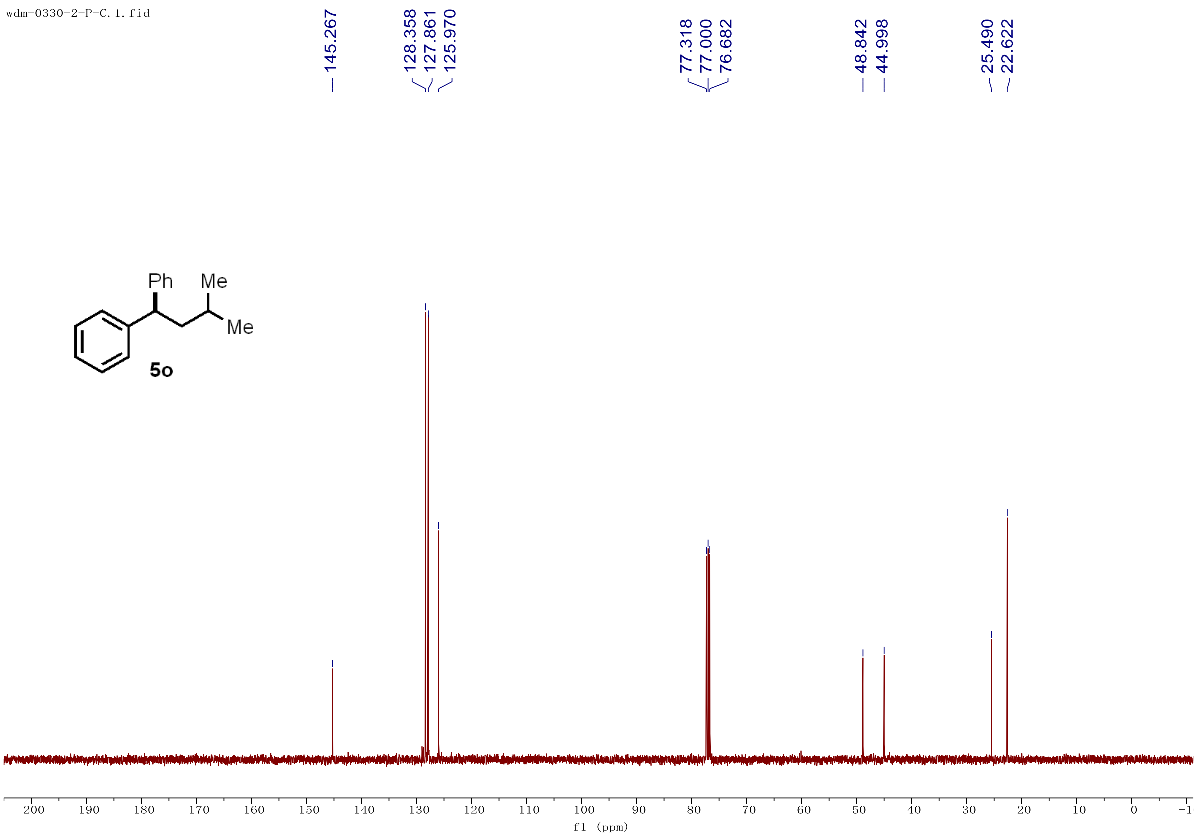

Supplementary Figure 139. ^{13}C NMR (100 MHz, $CDCl_3$) spectrum of 5o

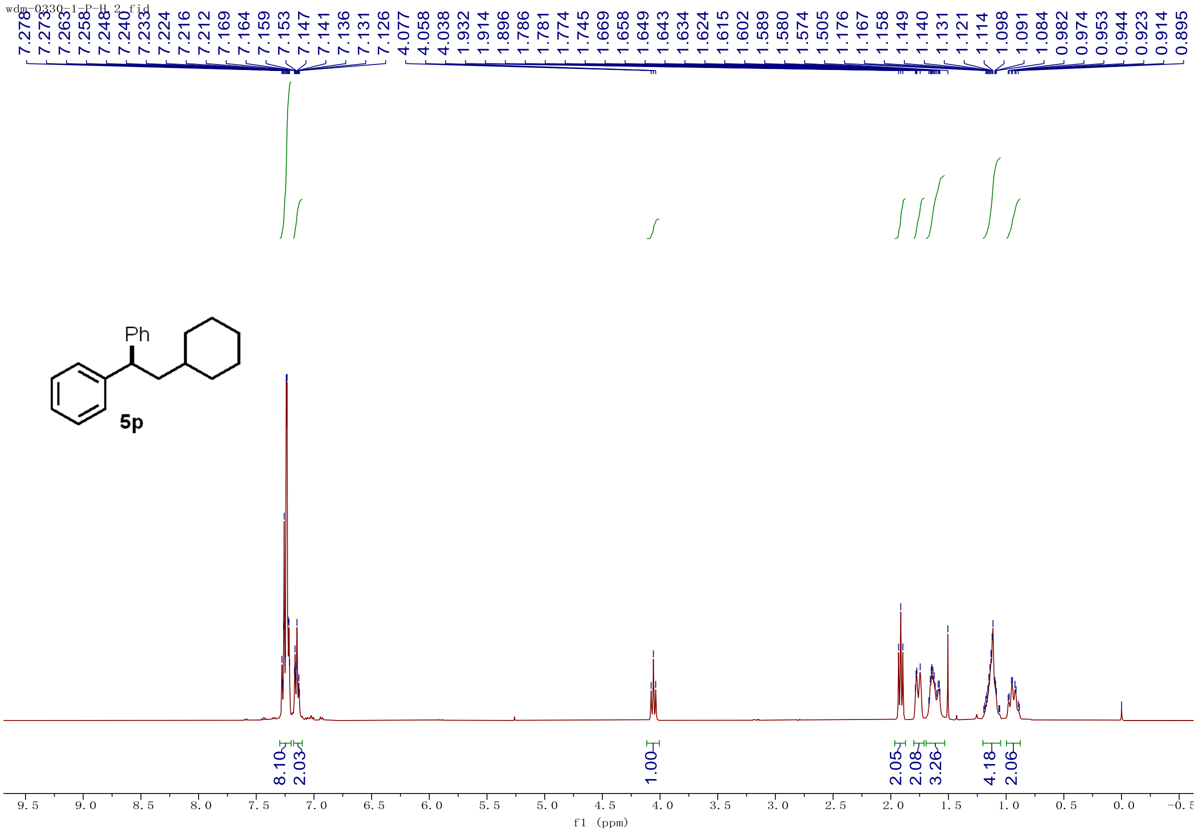

Supplementary Figure 140. ^{1}H NMR (400 MHz, $CDCl_3$) spectrum of 5p

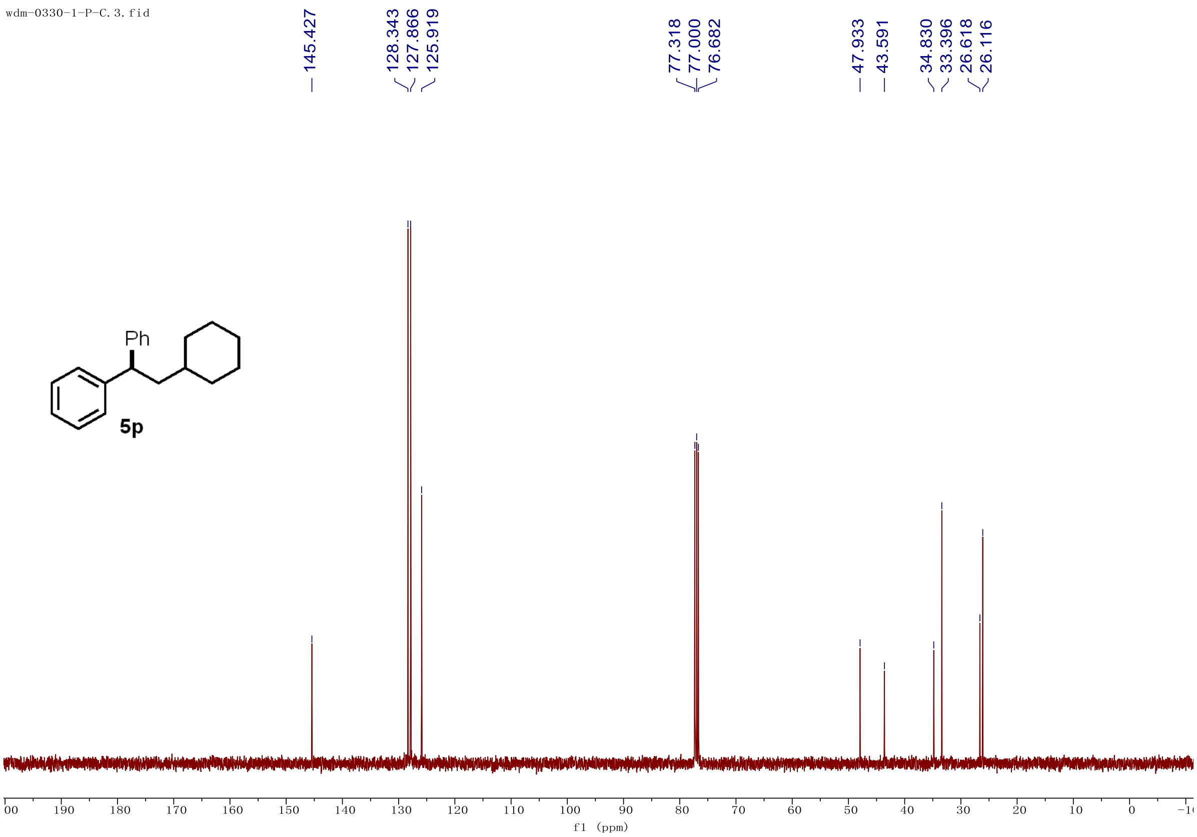

Supplementary Figure 141. **^{13}C NMR (100 MHz, CDCl₃) spectrum of 5p**

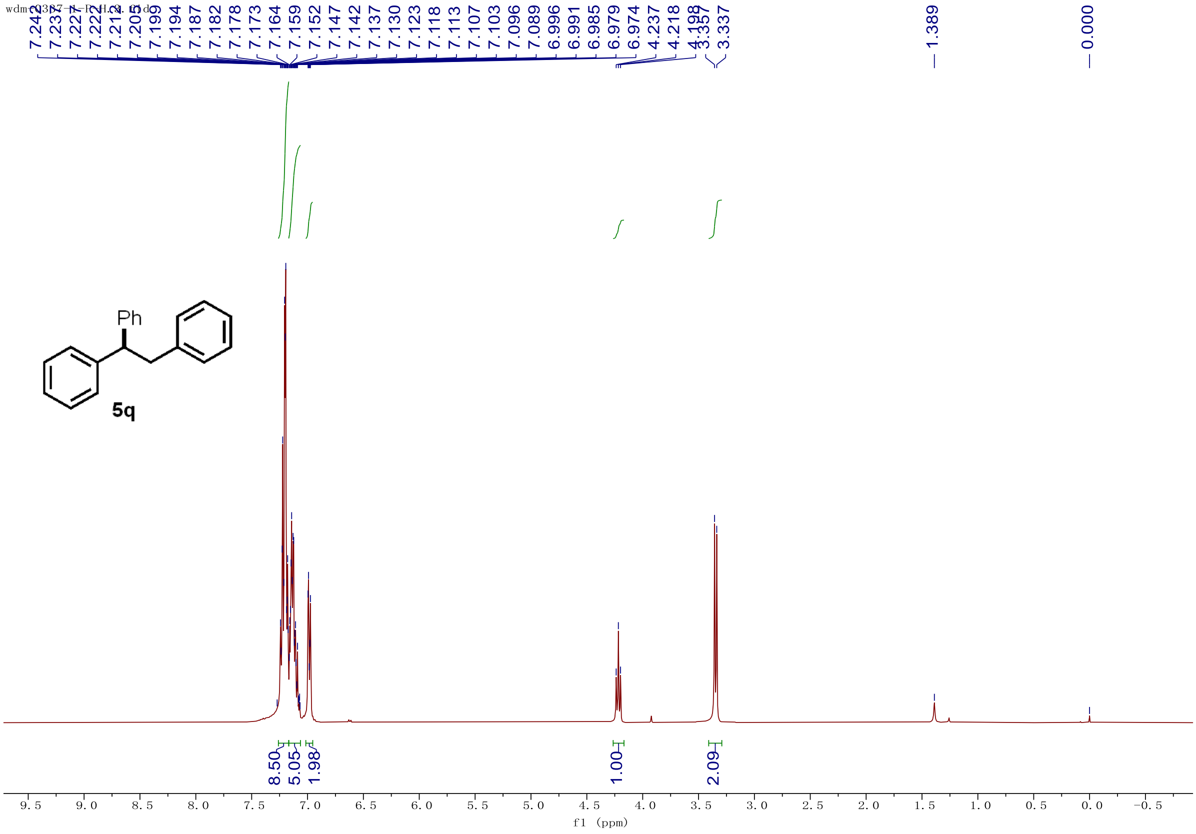

Supplementary Figure 142. **^{1}H NMR (400 MHz, CDCl₃) spectrum of 5q**

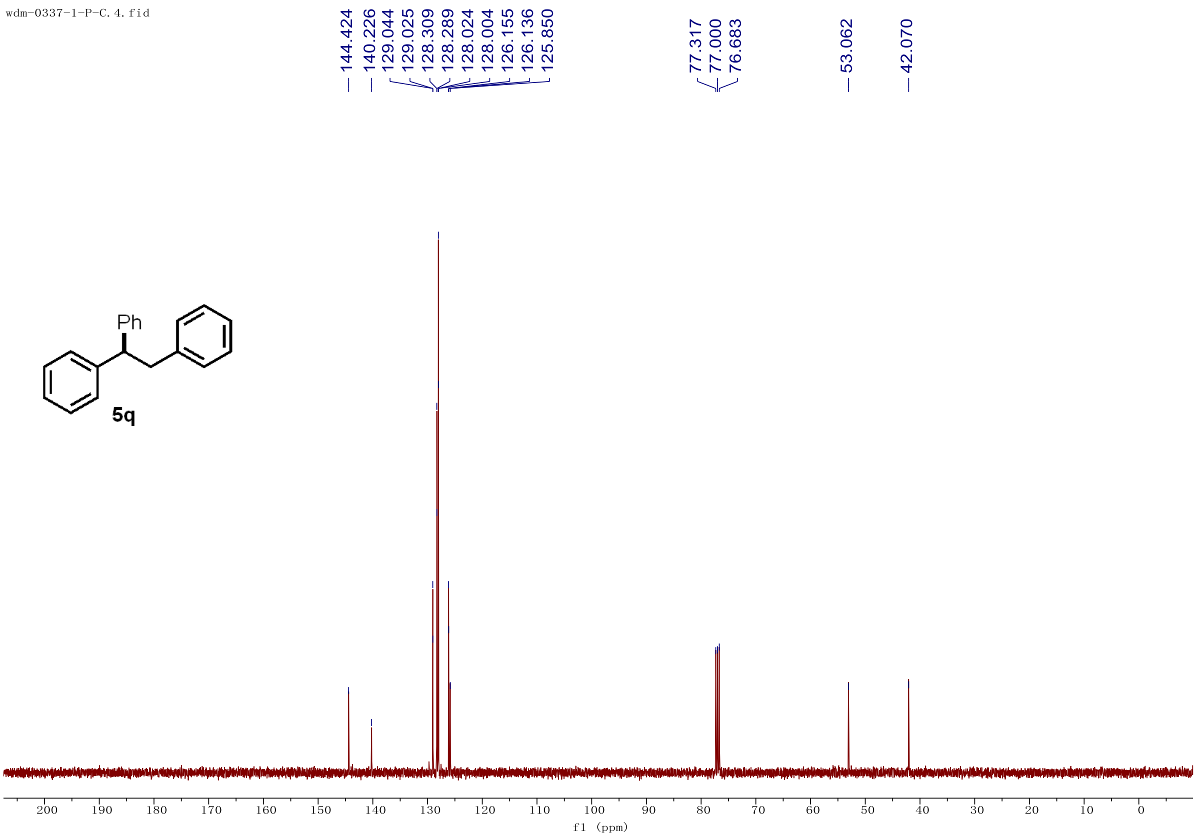

Supplementary Figure 143. ^{13}**C NMR (100 MHz, CDCl**$_3$**) spectrum of 5q**

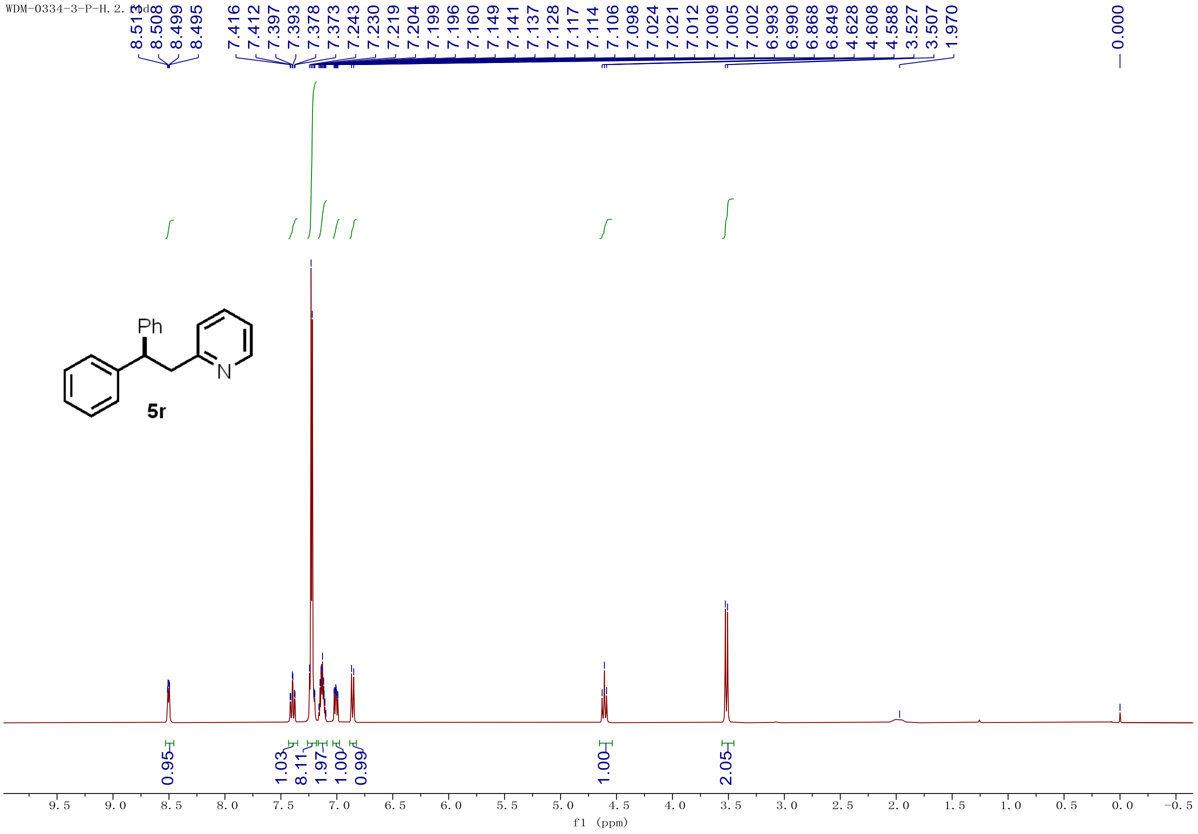

Supplementary Figure 144. ^{1}**H NMR (400 MHz, CDCl**$_3$**) spectrum of 5r**

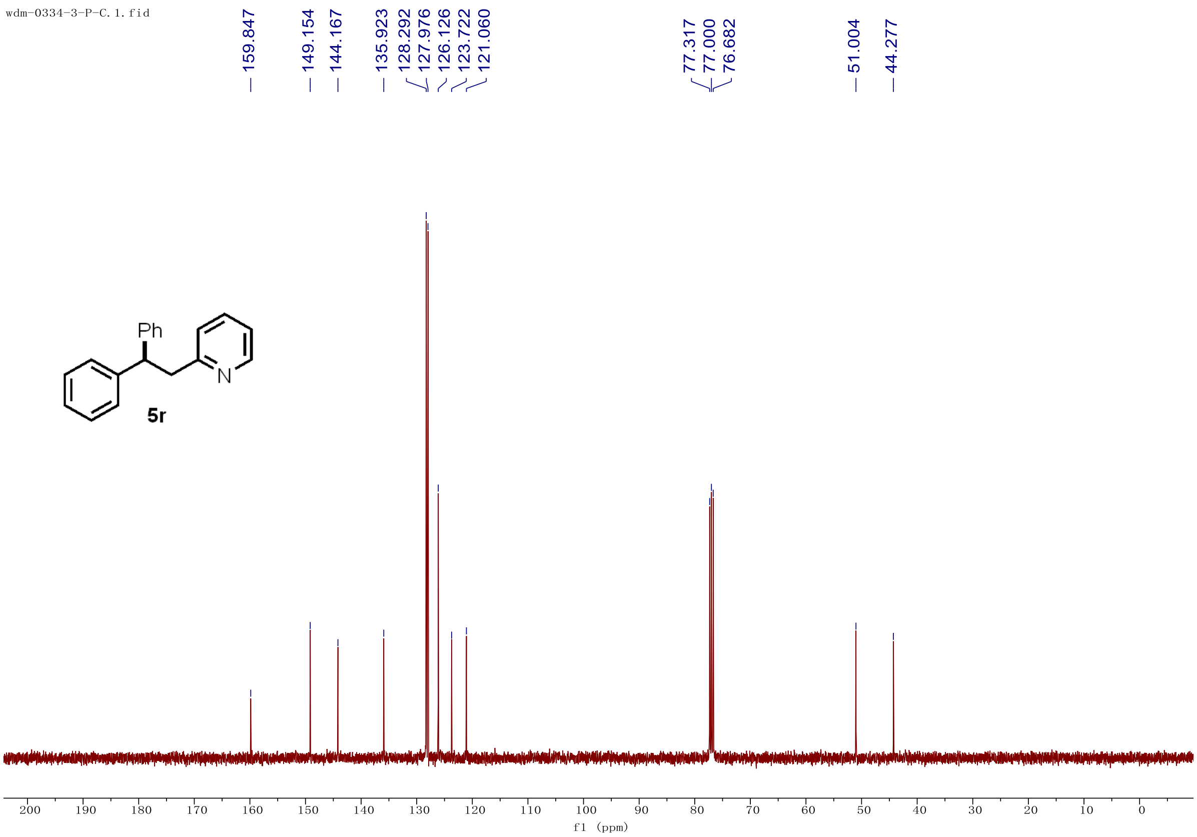

Supplementary Figure 145. ^{13}C NMR (100 MHz, $CDCl_3$) spectrum of 5r

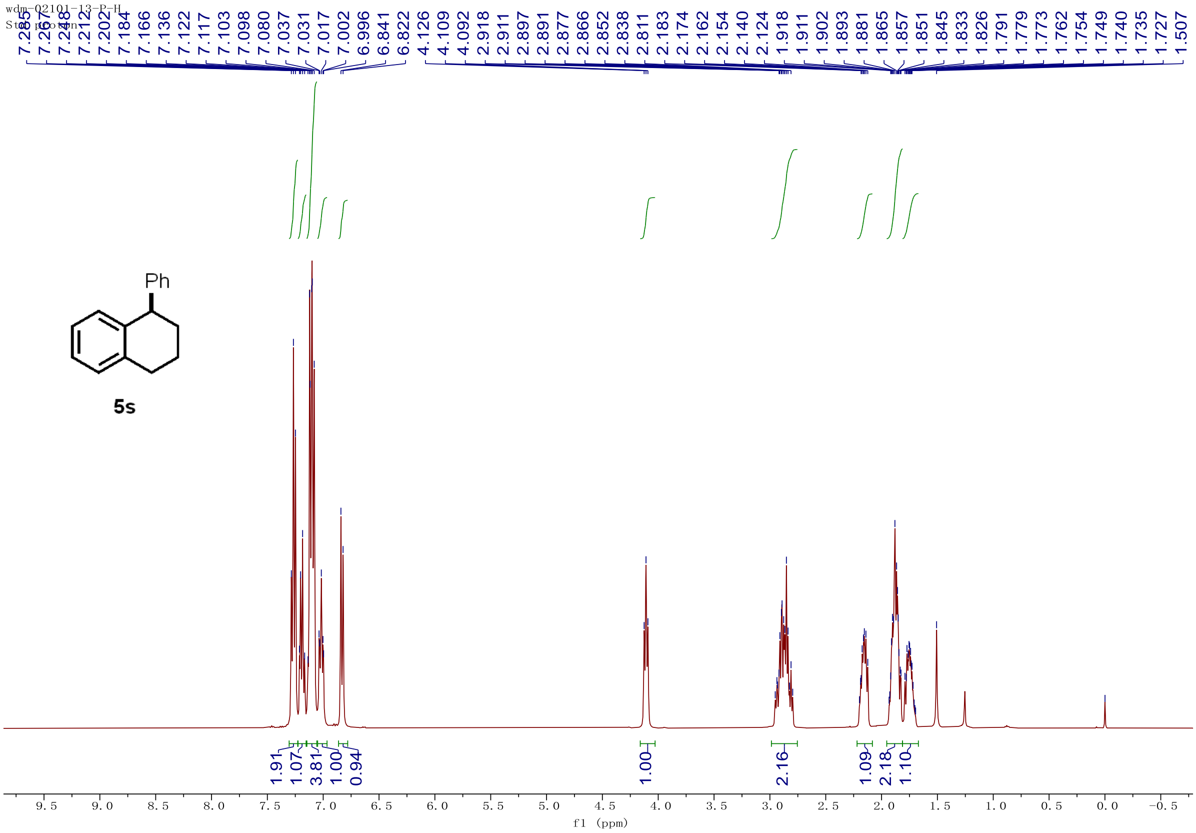

Supplementary Figure 146. ^{1}H NMR (400 MHz, $CDCl_3$) spectrum of 5s

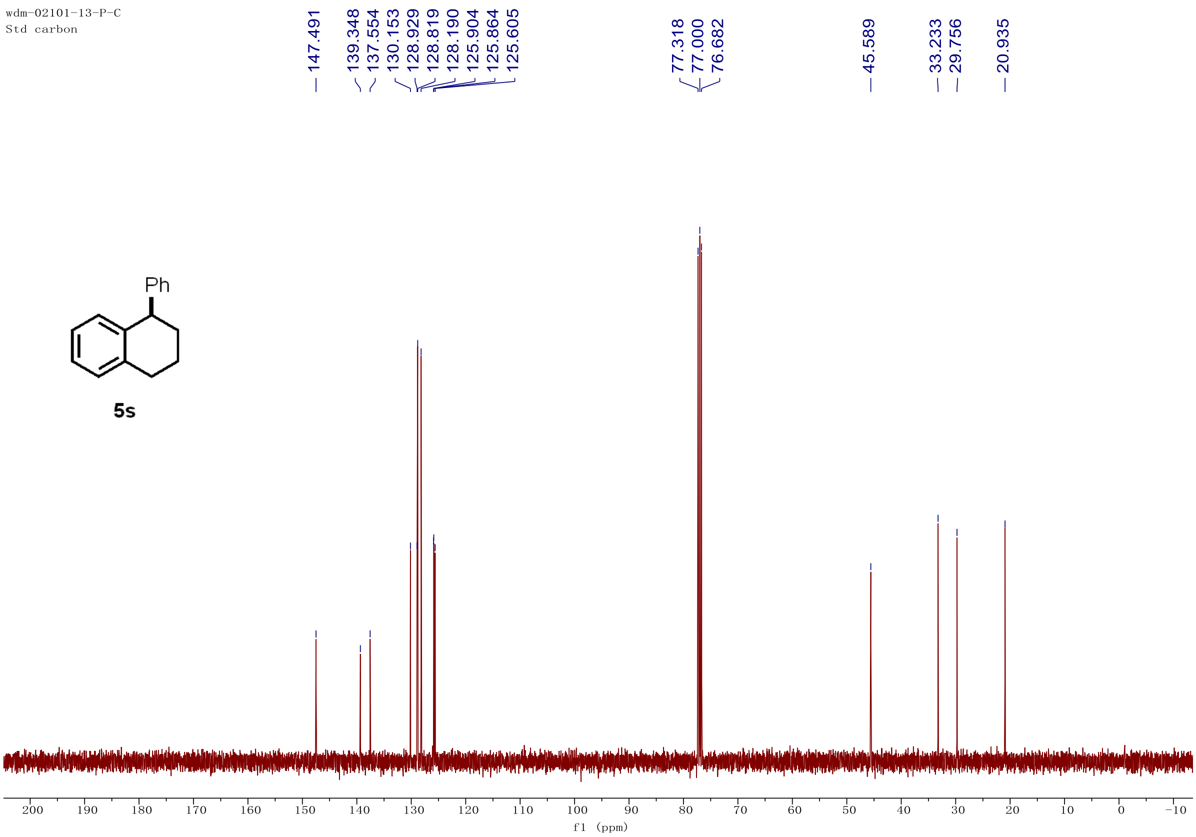

Supplementary Figure 147. ^{13}C NMR (100 MHz, $CDCl_3$) spectrum of 5s

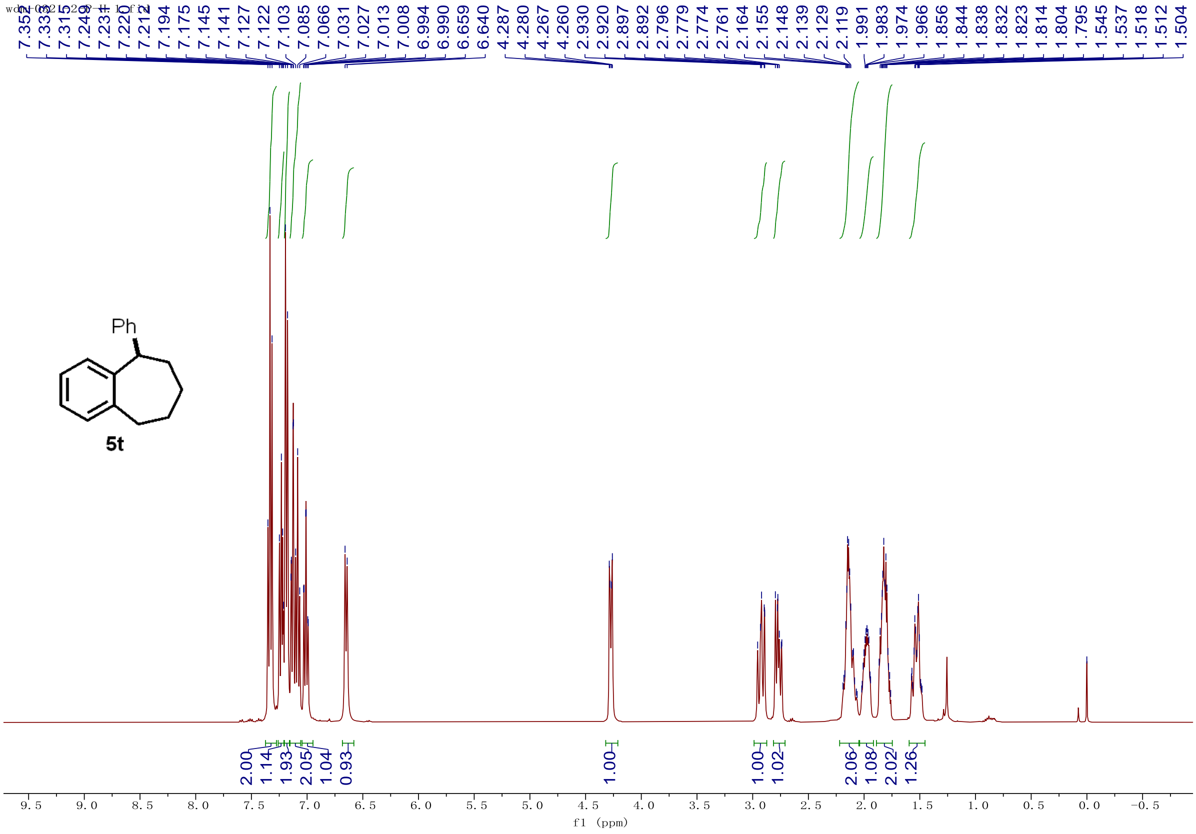

Supplementary Figure 148. ^{1}H NMR (400 MHz, $CDCl_3$) spectrum of 5t

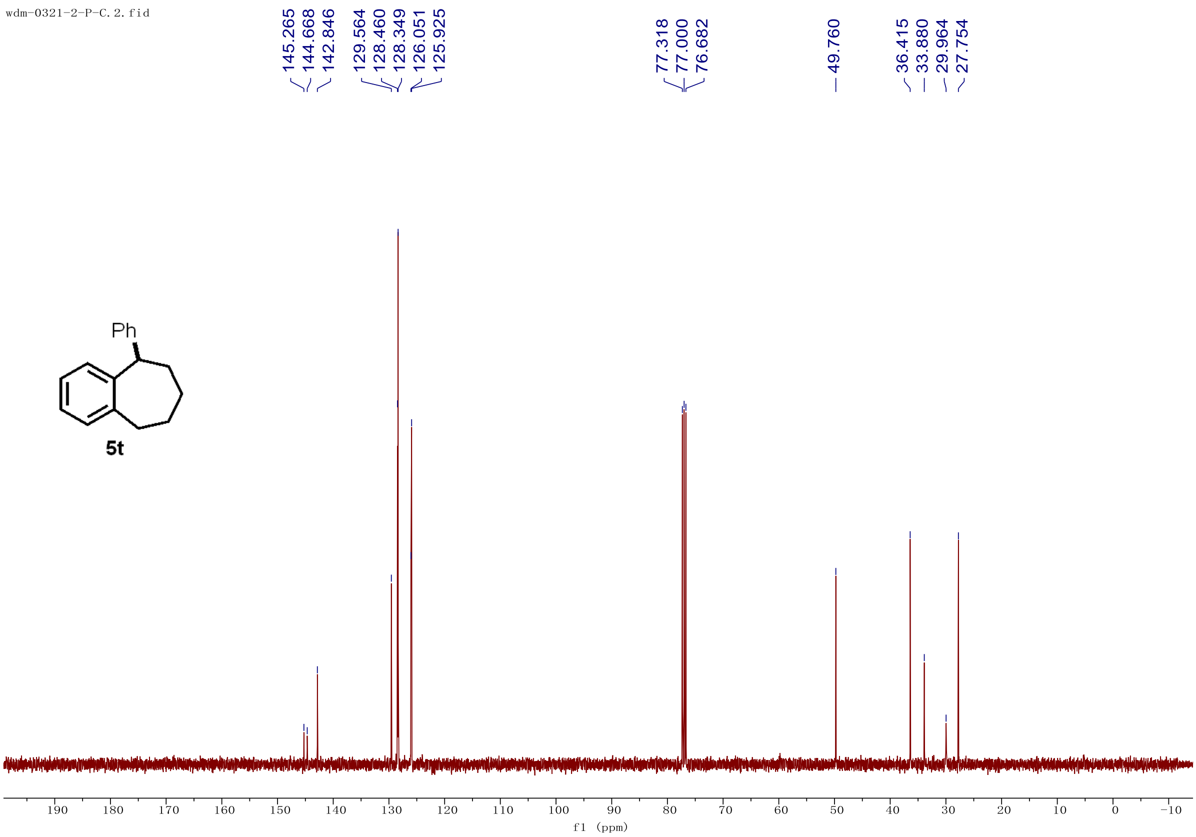

Supplementary Figure 149. ^{13}C NMR (100 MHz, $CDCl_3$) spectrum of 5t

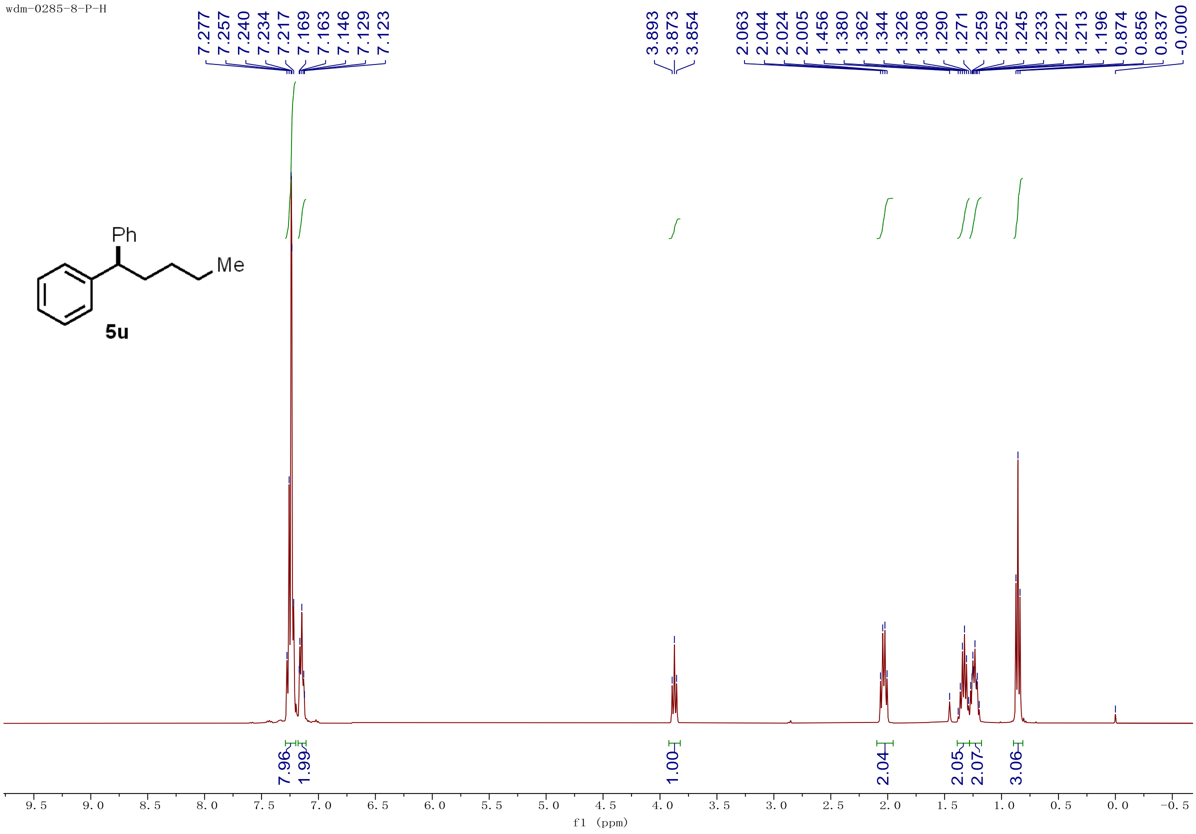

Supplementary Figure 150. ^{1}H NMR (400 MHz, $CDCl_3$) spectrum of 5u

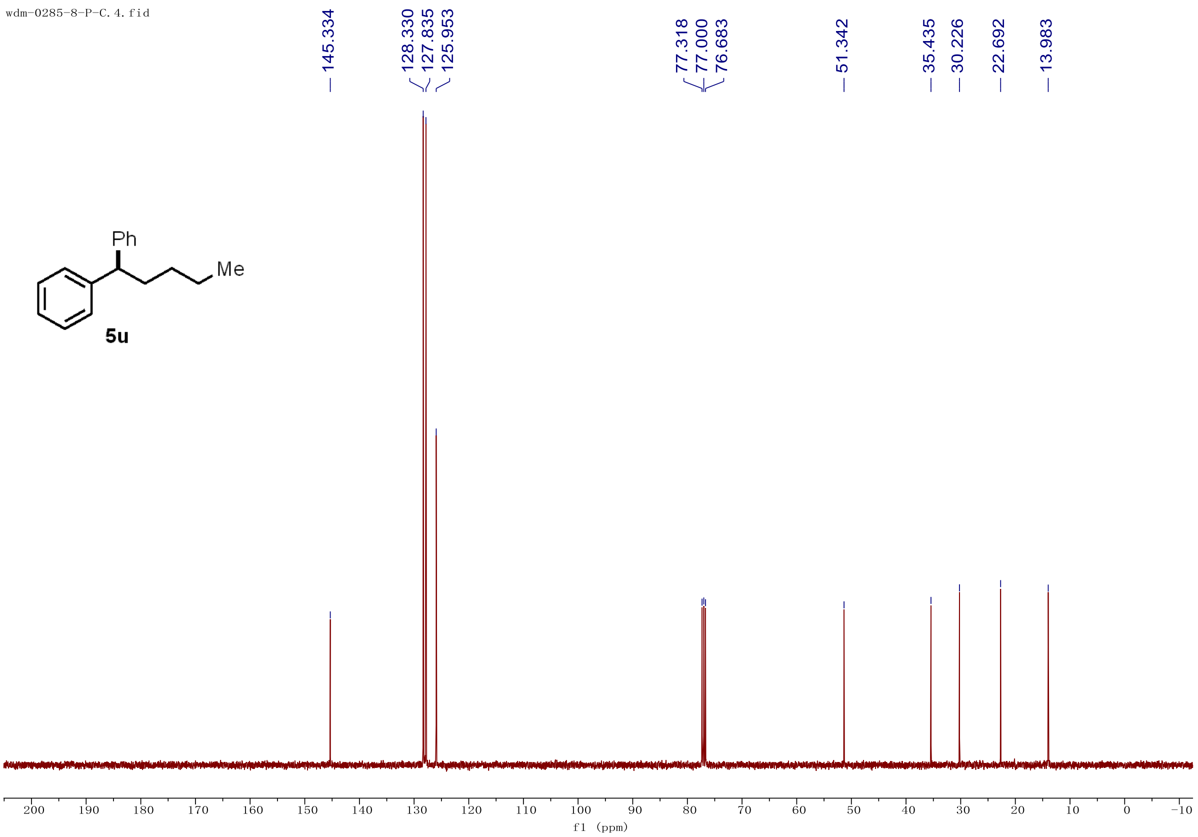

Supplementary Figure 151. ^{13}C NMR (100 MHz, $CDCl_3$) spectrum of 5u

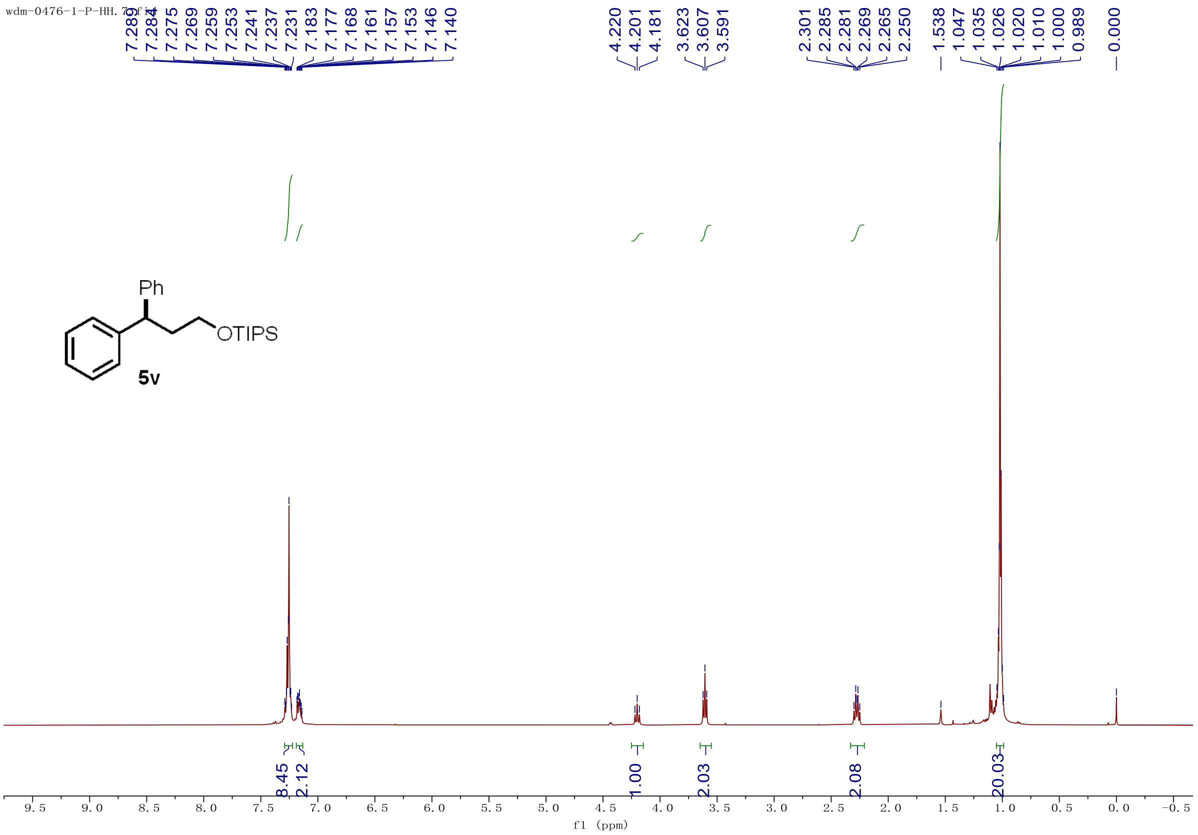

Supplementary Figure 152. ^{1}H NMR (400 MHz, $CDCl_3$) spectrum of 5v

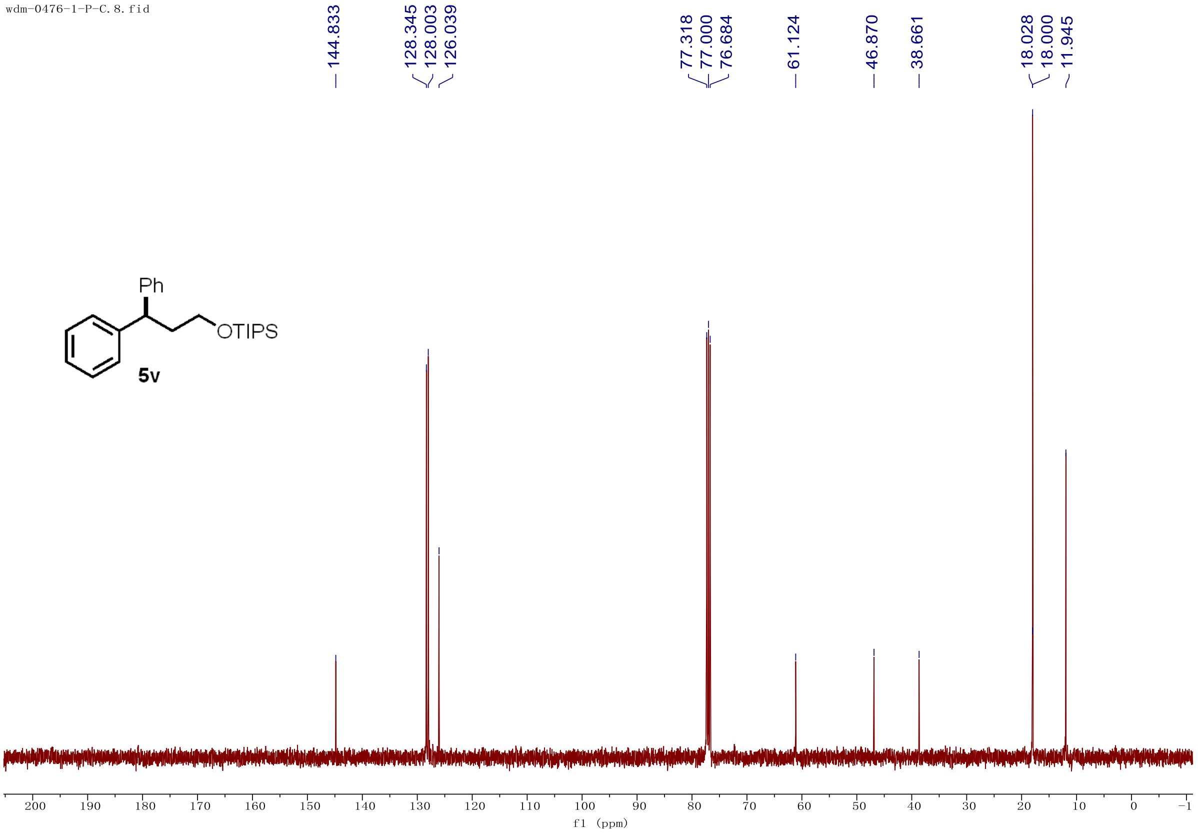

Supplementary Figure 153. ^{13}**C NMR (100 MHz, CDCl$_3$) spectrum of 5v**

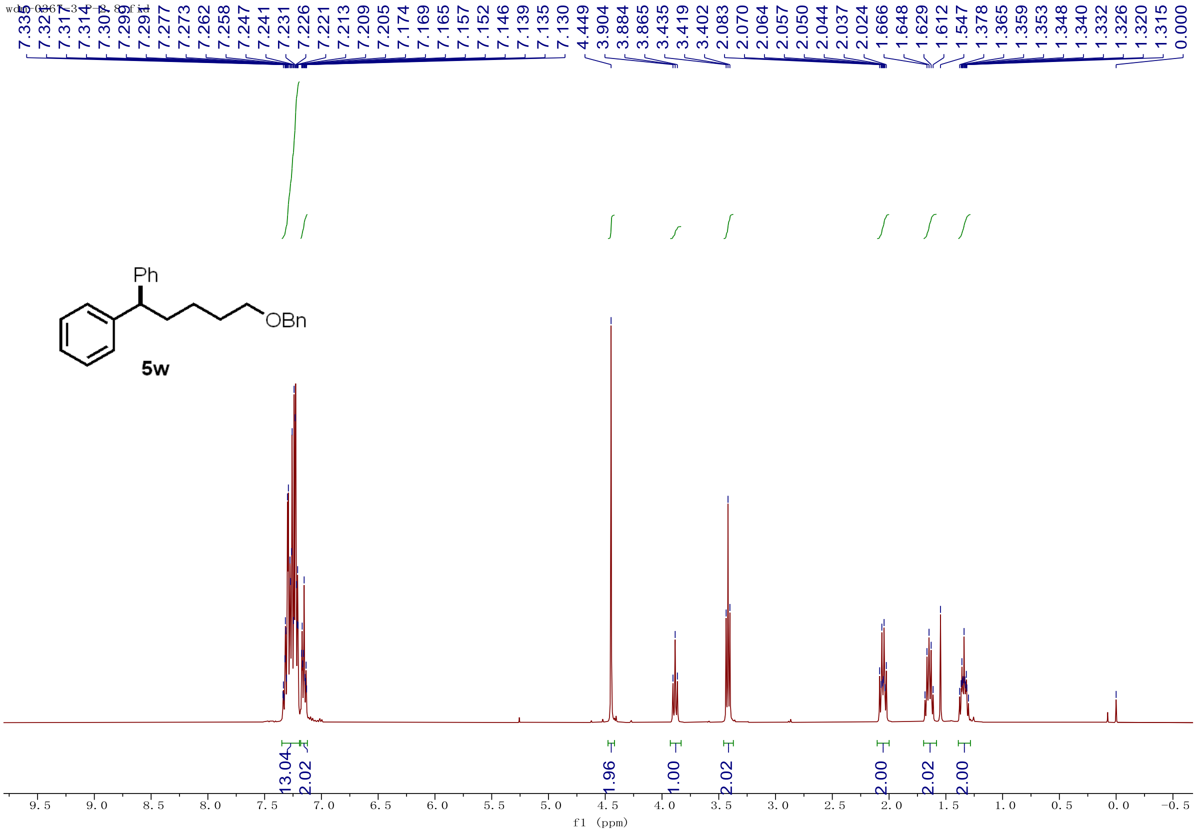

Supplementary Figure 154. ^{1}**H NMR (400 MHz, CDCl$_3$) spectrum of 5w**

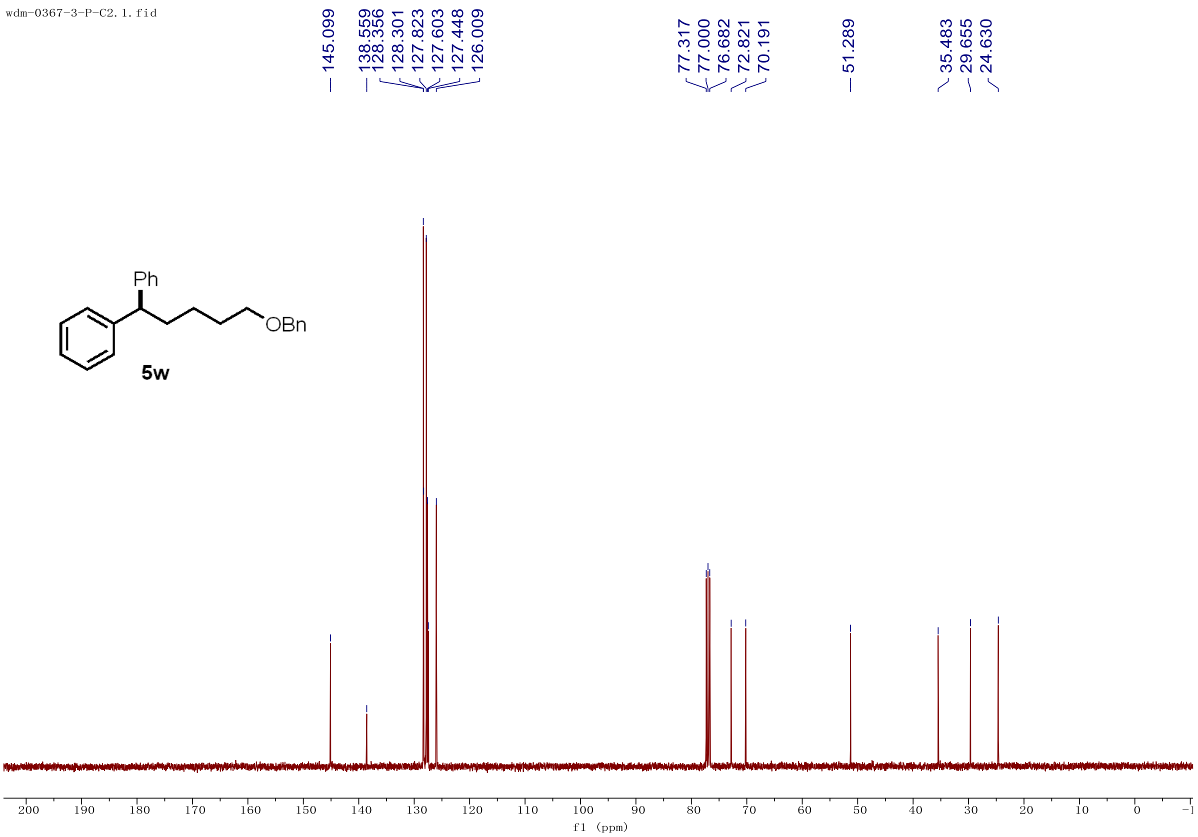

Supplementary Figure 155. ^{13}C NMR (100 MHz, $CDCl_3$) spectrum of 5w

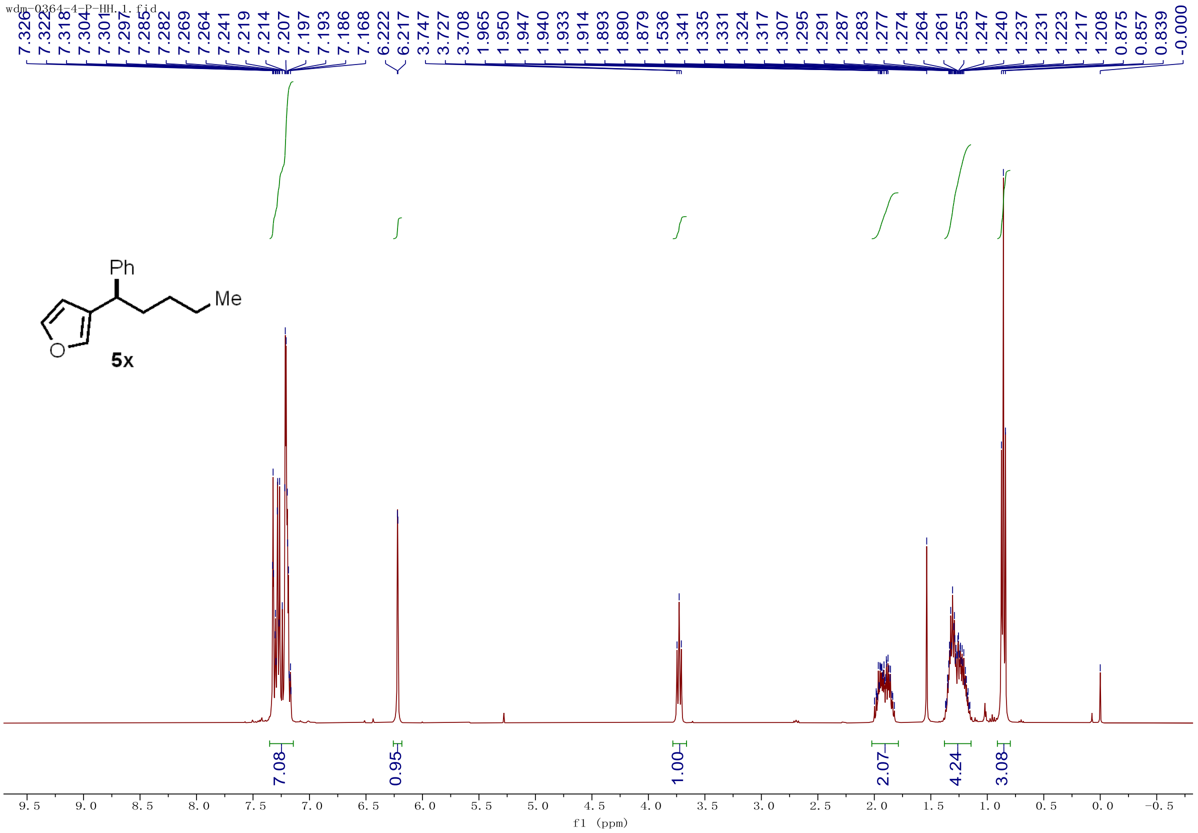

Supplementary Figure 156. ^{1}H NMR (400 MHz, $CDCl_3$) spectrum of 5x

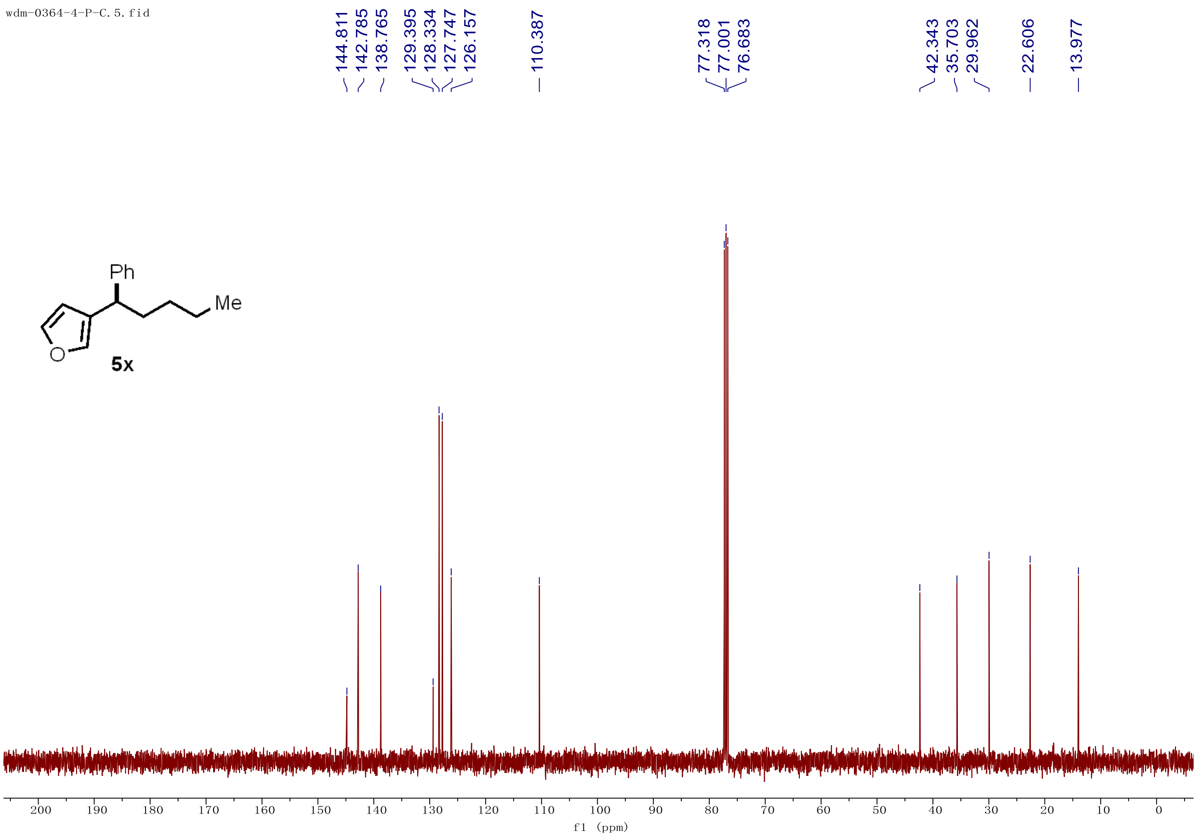

Supplementary Figure 157. ^{13}C NMR (100 MHz, CDCl$_3$) spectrum of 5x

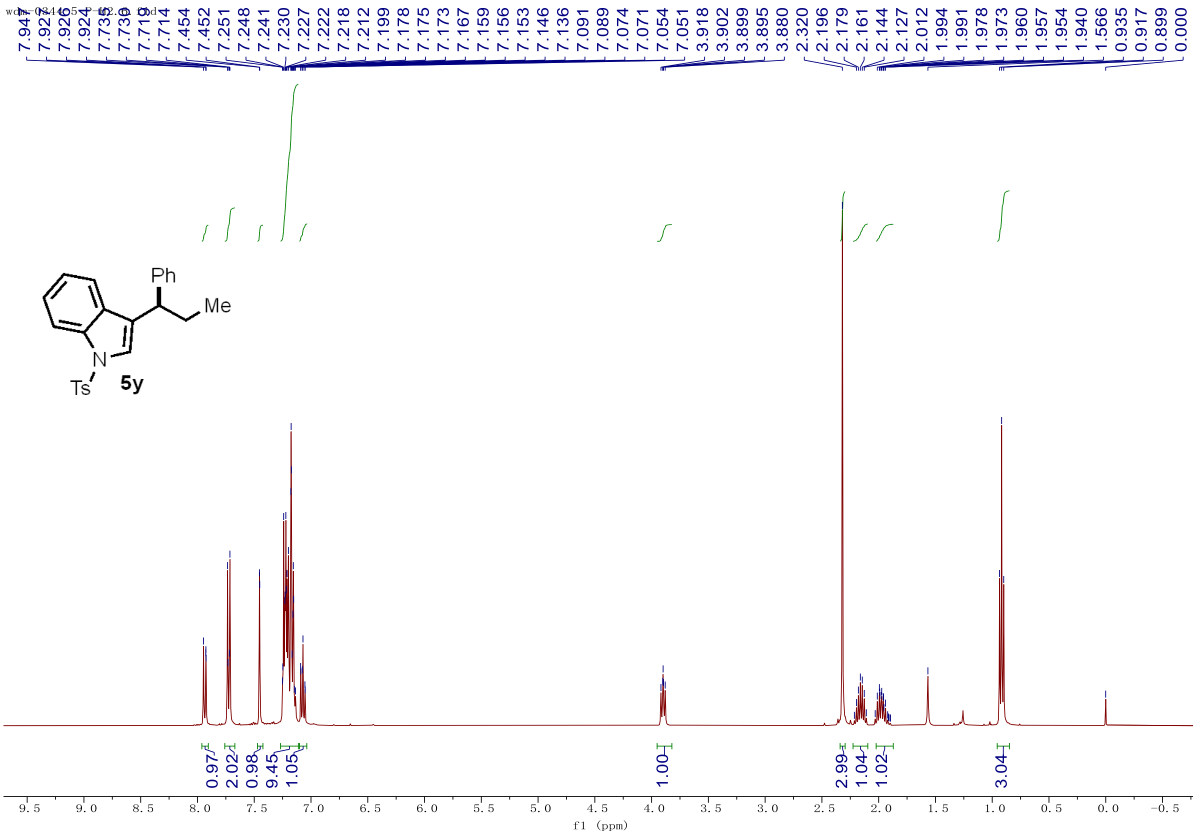

Supplementary Figure 158. ^{1}H NMR (400 MHz, CDCl$_3$) spectrum of 5y

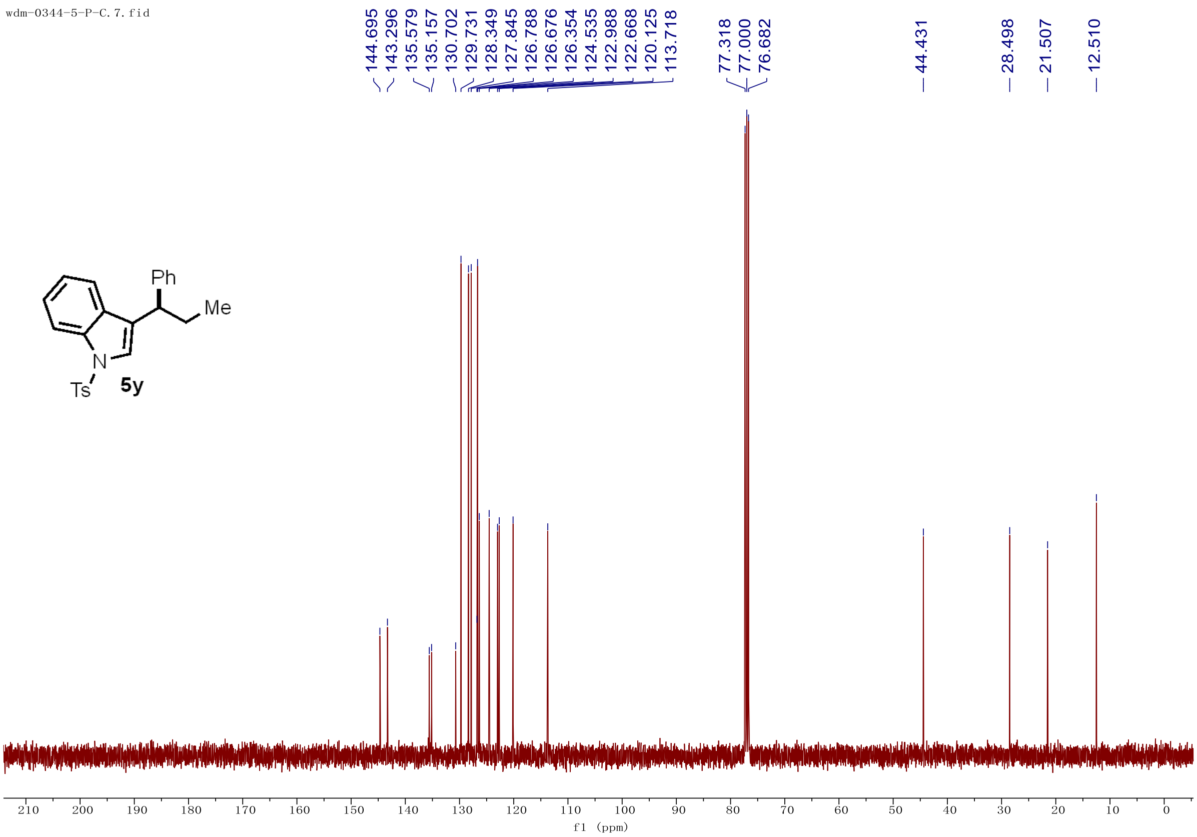

Supplementary Figure 159. ^{13}C NMR (100 MHz, $CDCl_3$) spectrum of 5y

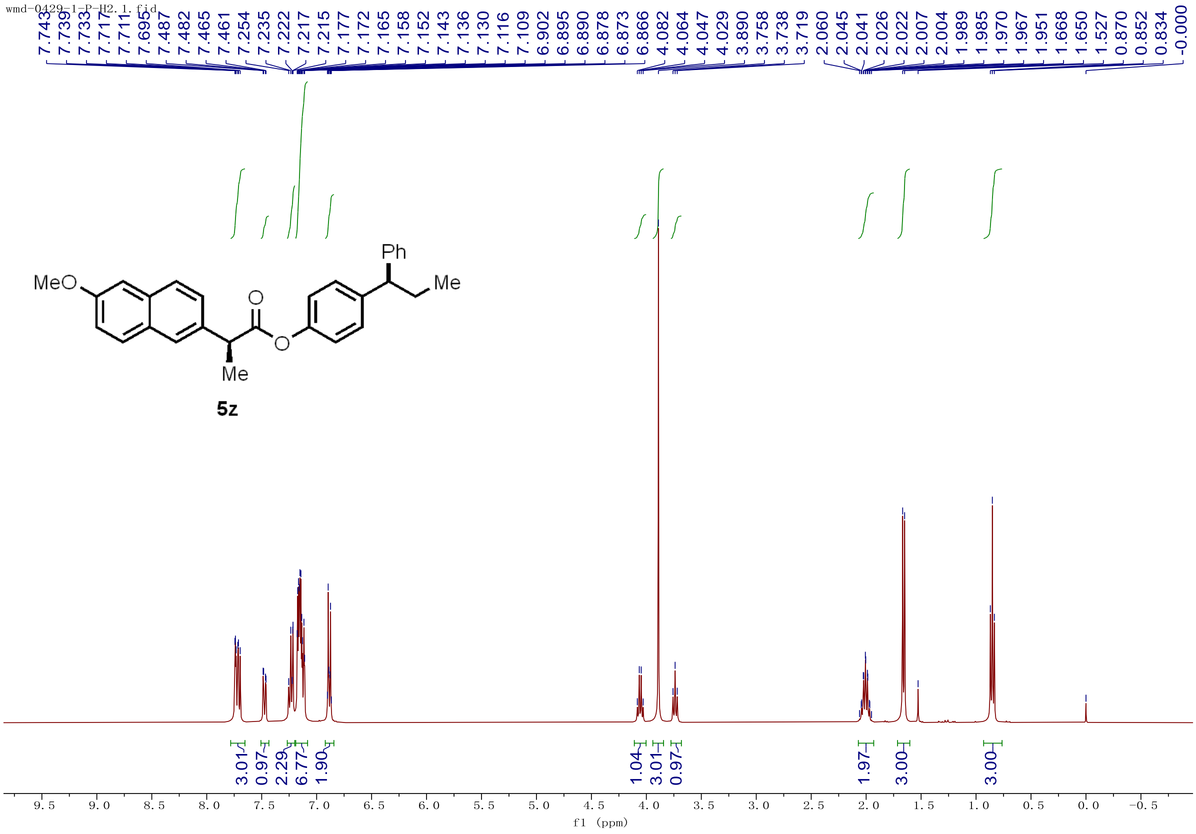

Supplementary Figure 160. ^{1}H NMR (400 MHz, $CDCl_3$) spectrum of 5z

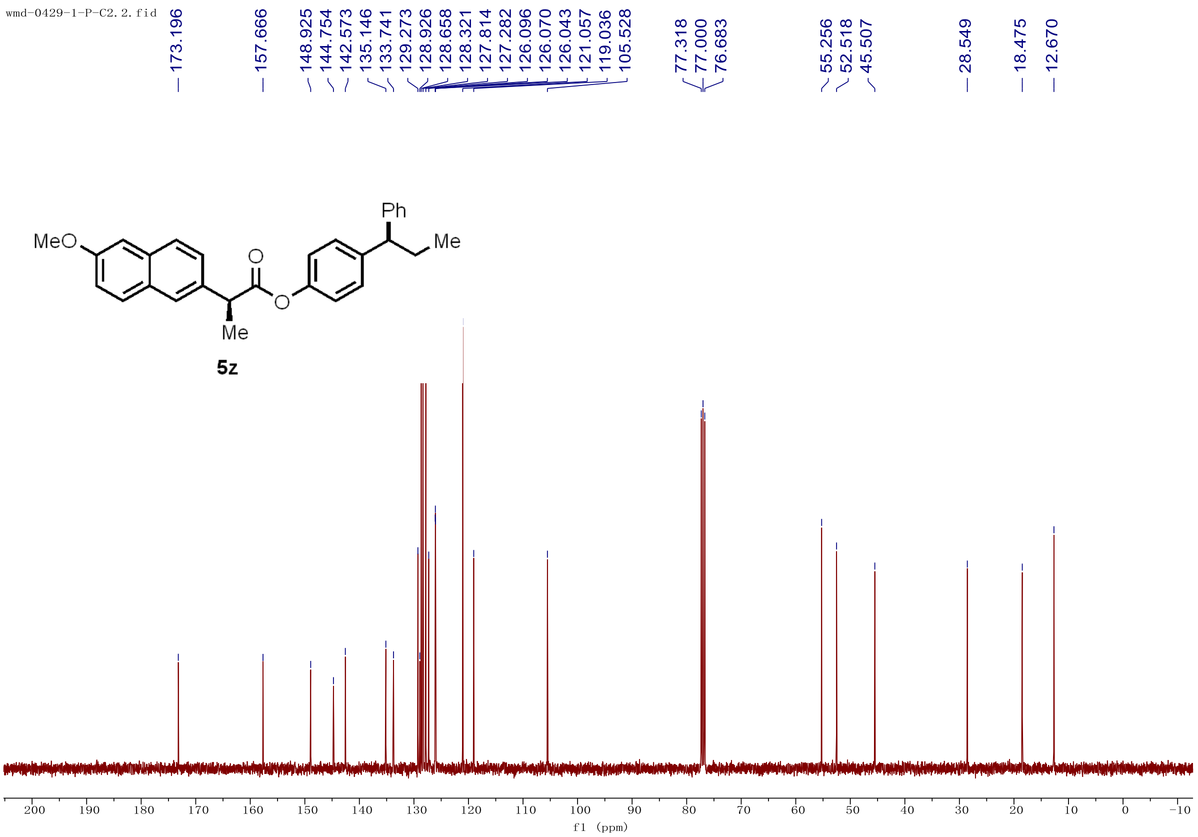

Supplementary Figure 161. ^{13}C NMR (100 MHz, $CDCl_3$) spectrum of 5z

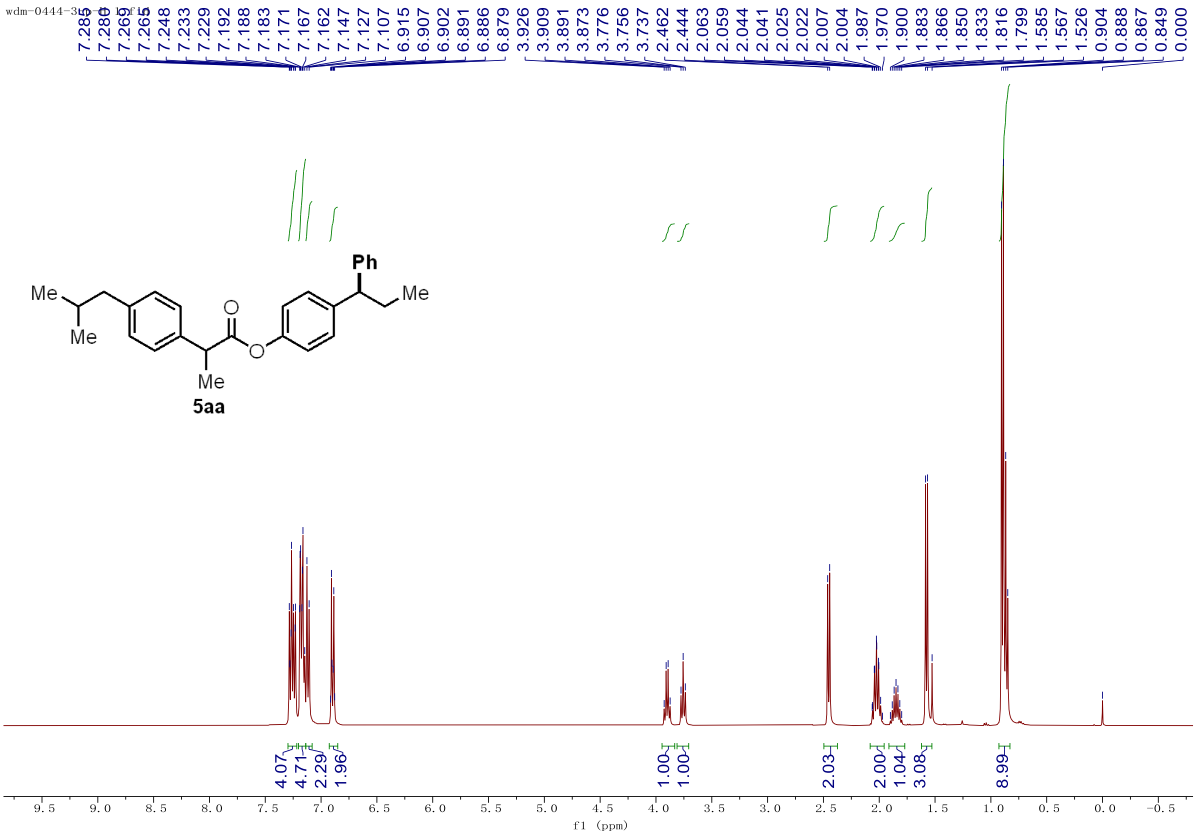

Supplementary Figure 162. ^{1}H NMR (400 MHz, $CDCl_3$) spectrum of 5aa

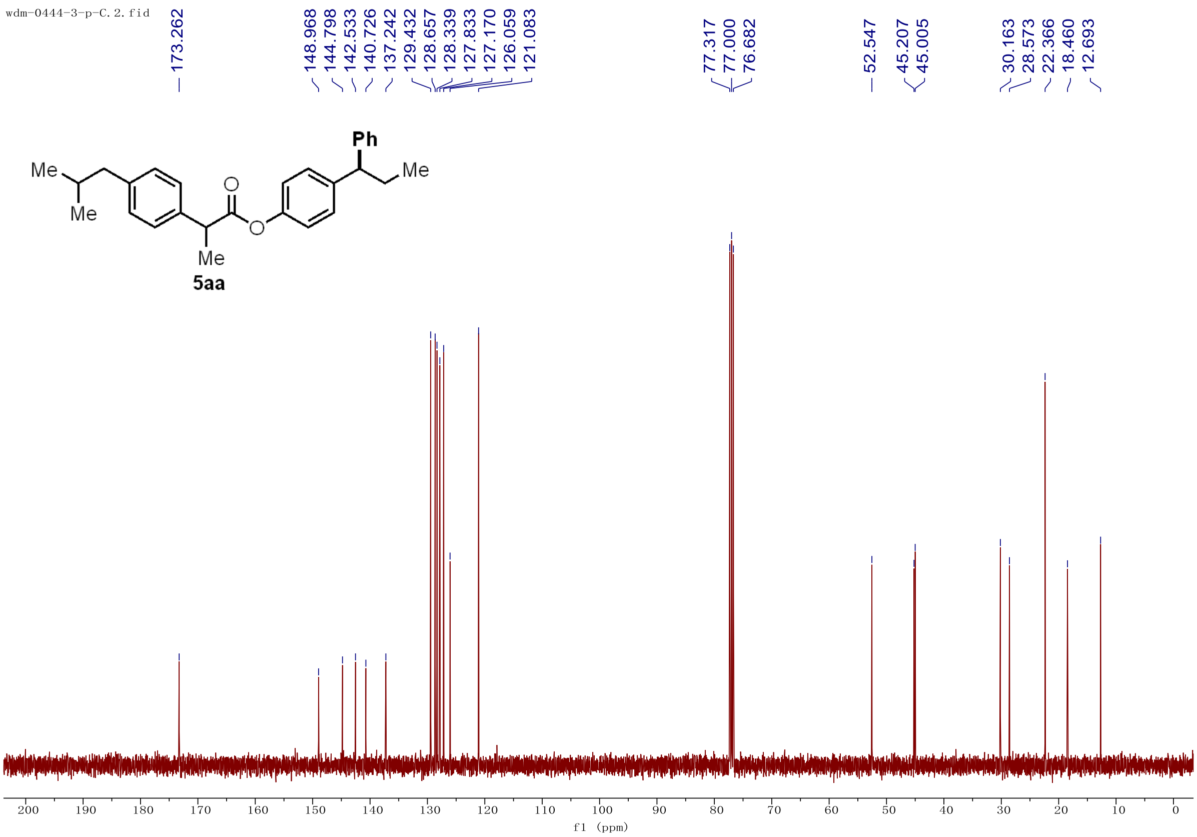

Supplementary Figure 163. ^{13}C NMR (100 MHz, CDCl$_3$) spectrum of 5aa

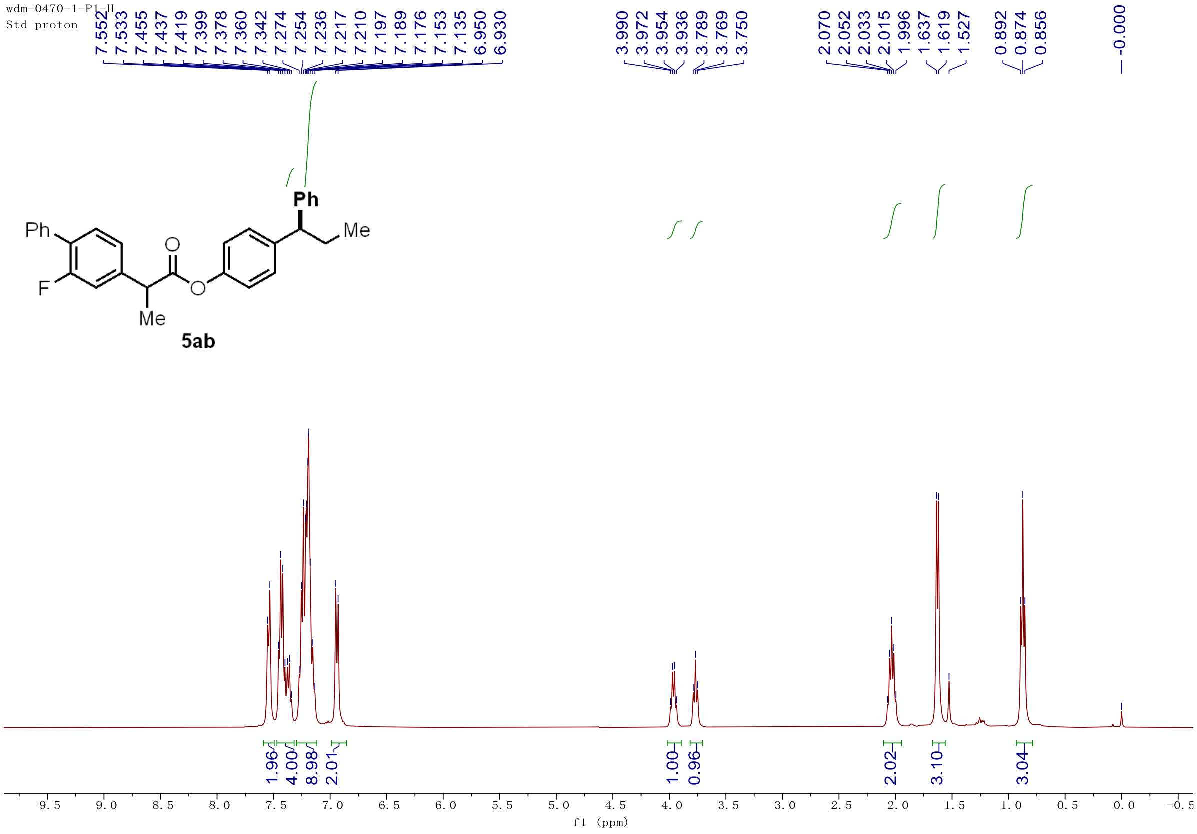

Supplementary Figure 164. ^{1}H NMR (400 MHz, CDCl$_3$) spectrum of 5ab

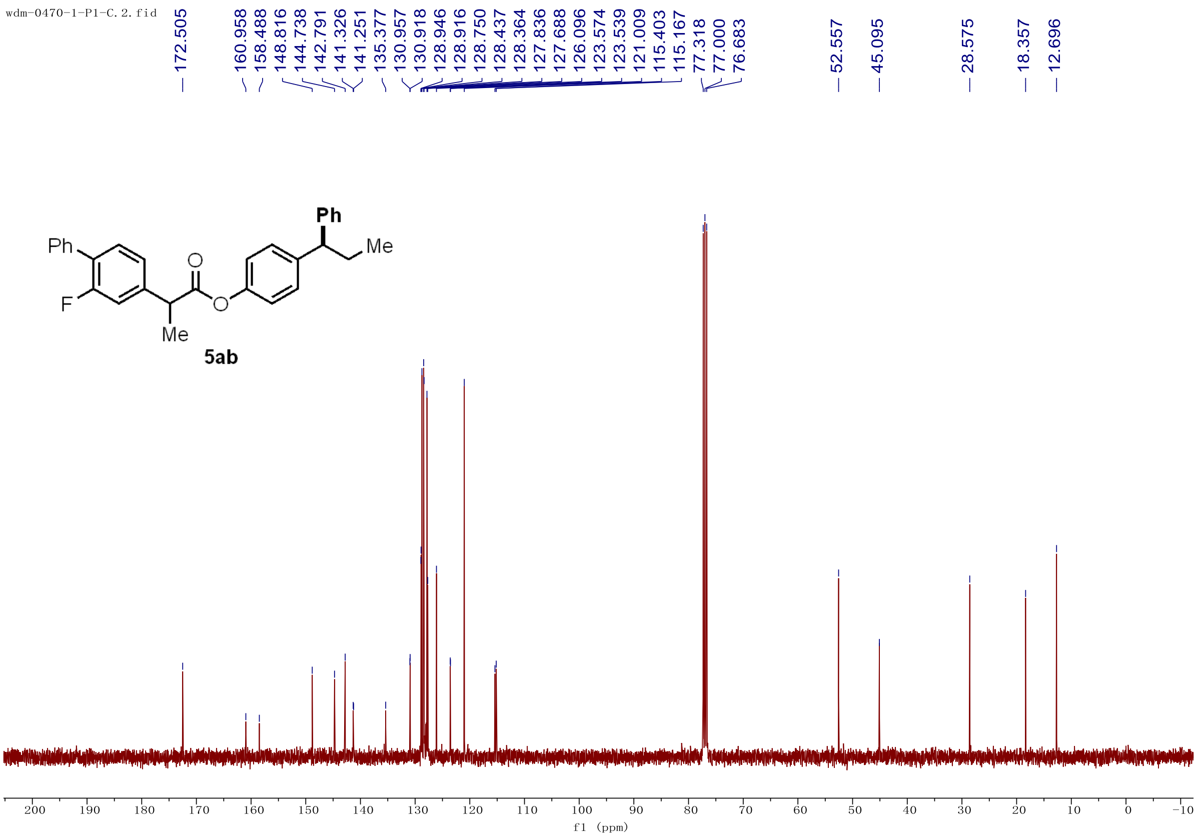

Supplementary Figure 165. ^{13}**C NMR (100 MHz, CDCl**$_3$**) spectrum of 5ab**

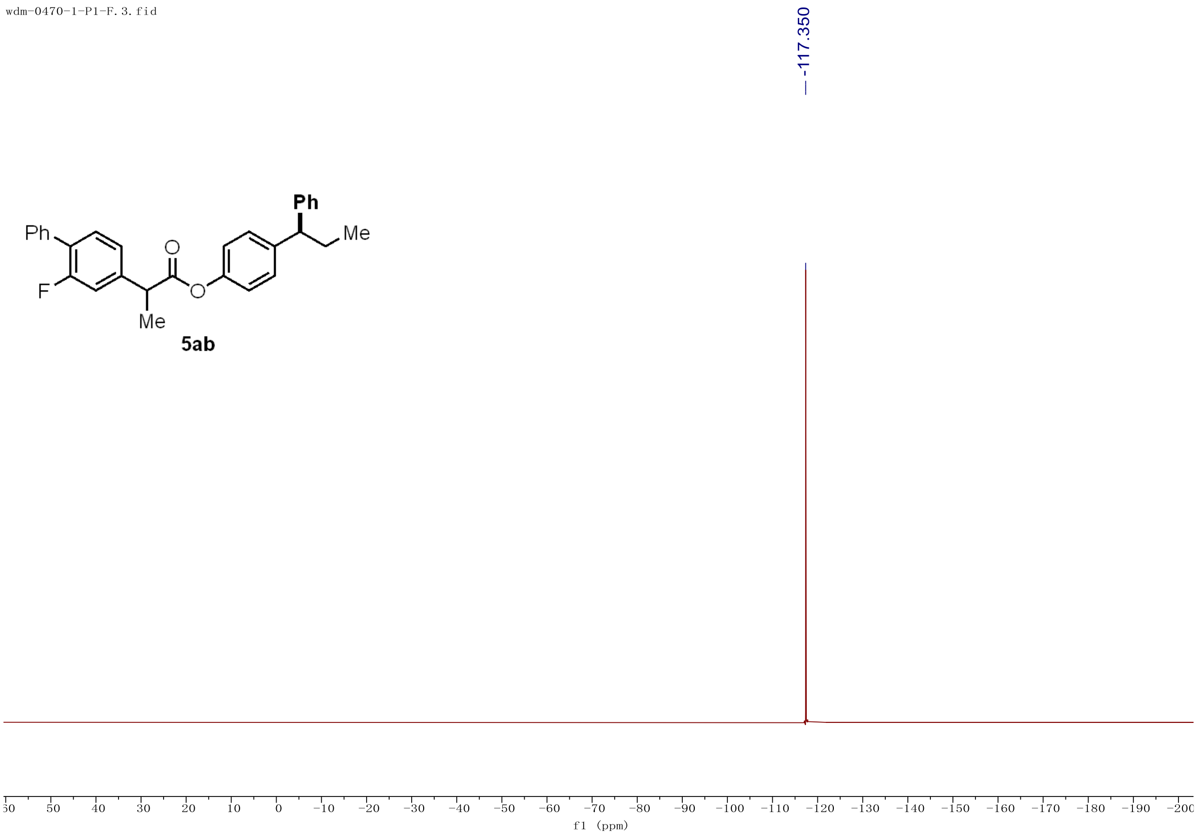

Supplementary Figure 166. ^{19}**F NMR (375 MHz, CDCl**$_3$**) spectrum of 5ab**

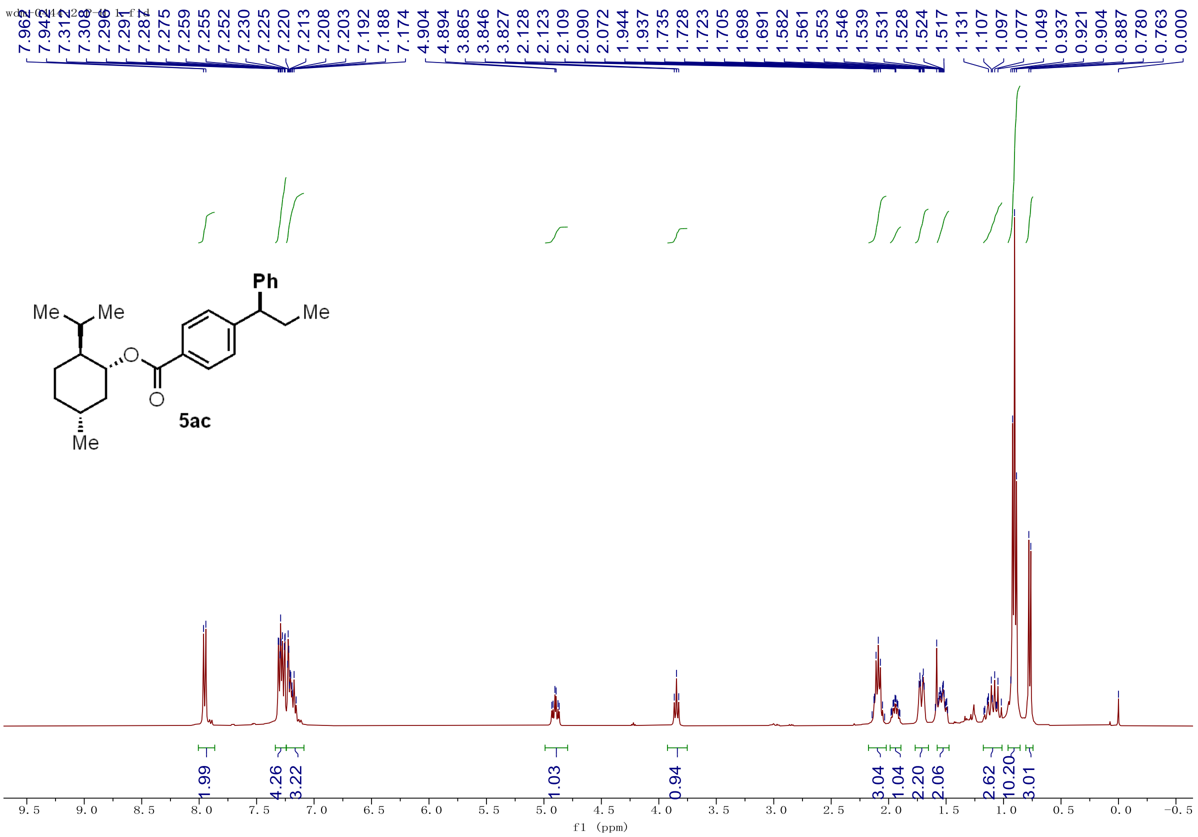

Supplementary Figure 167. **^{1}H NMR (400 MHz, CDCl3) spectrum of 5ac**

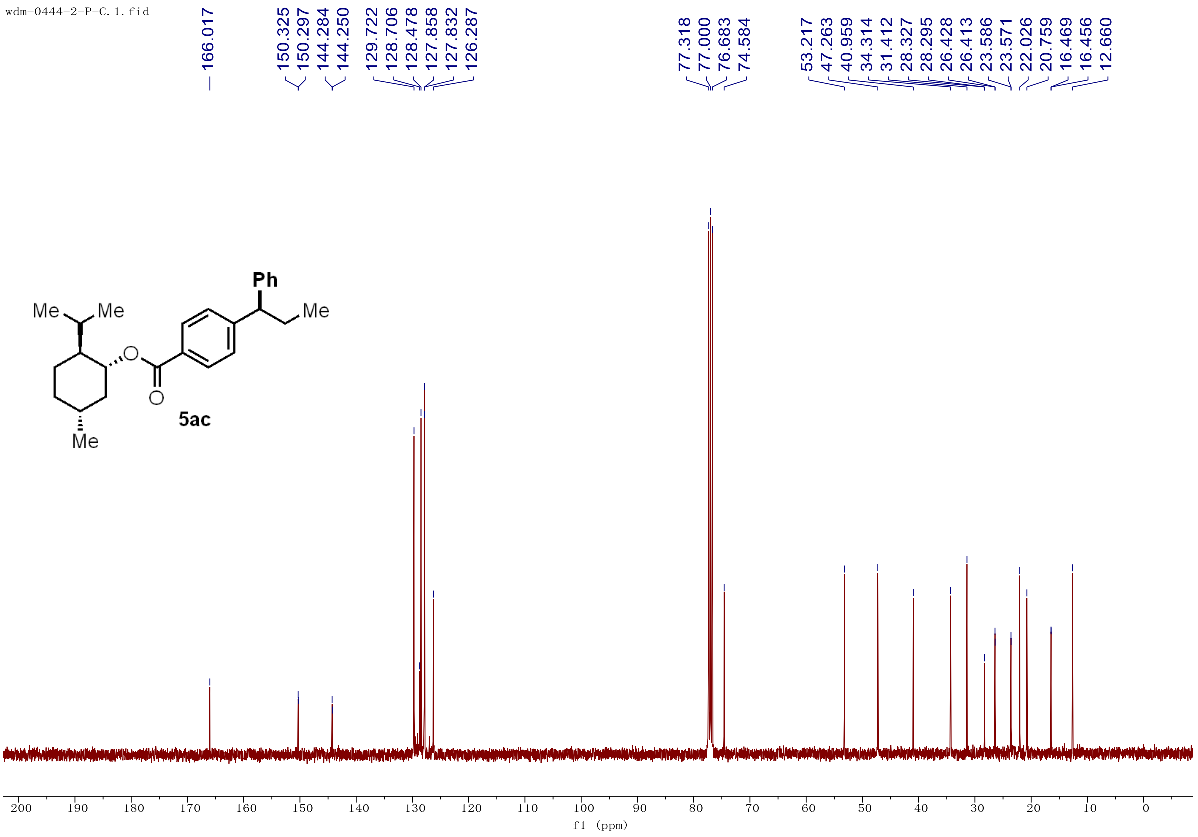

Supplementary Figure 168. **^{13}C NMR (100 MHz, CDCl3) spectrum of 5ac**

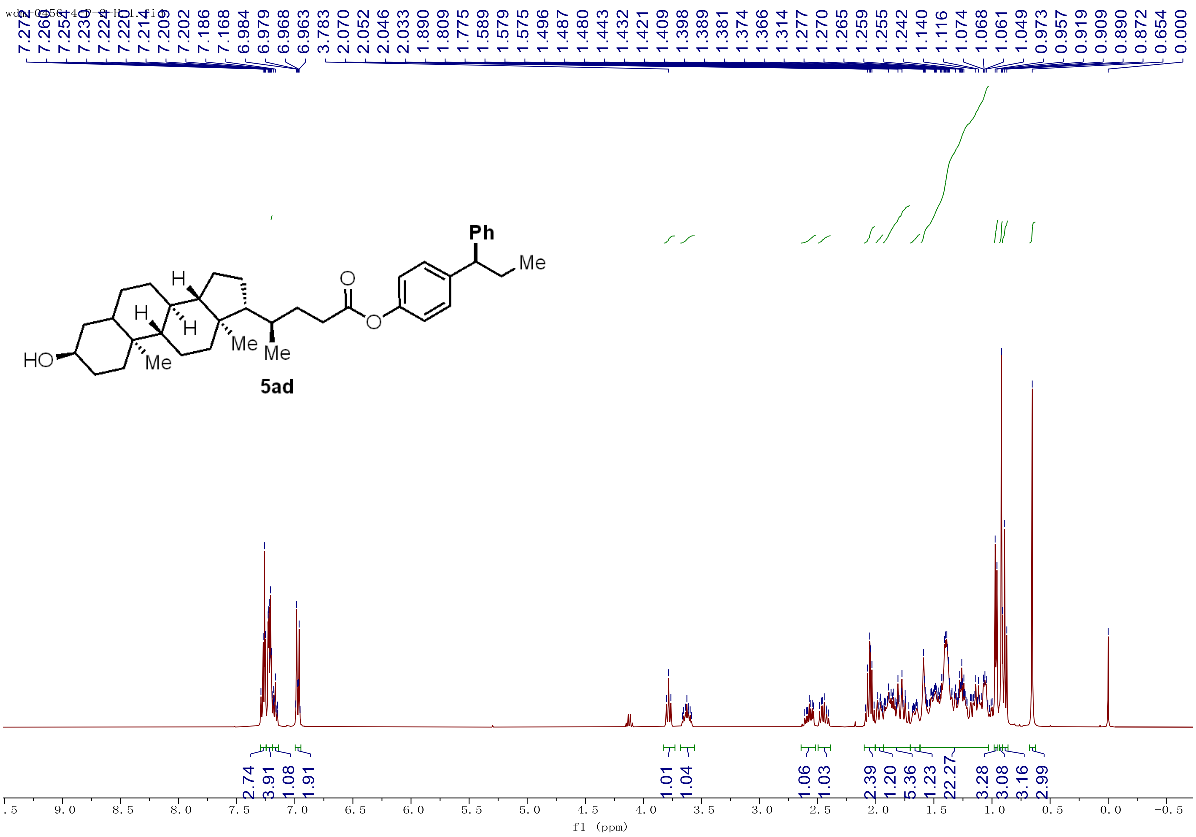

Supplementary Figure 169. ^{1}**H NMR (400 MHz, CDCl$_3$) spectrum of 5ad**

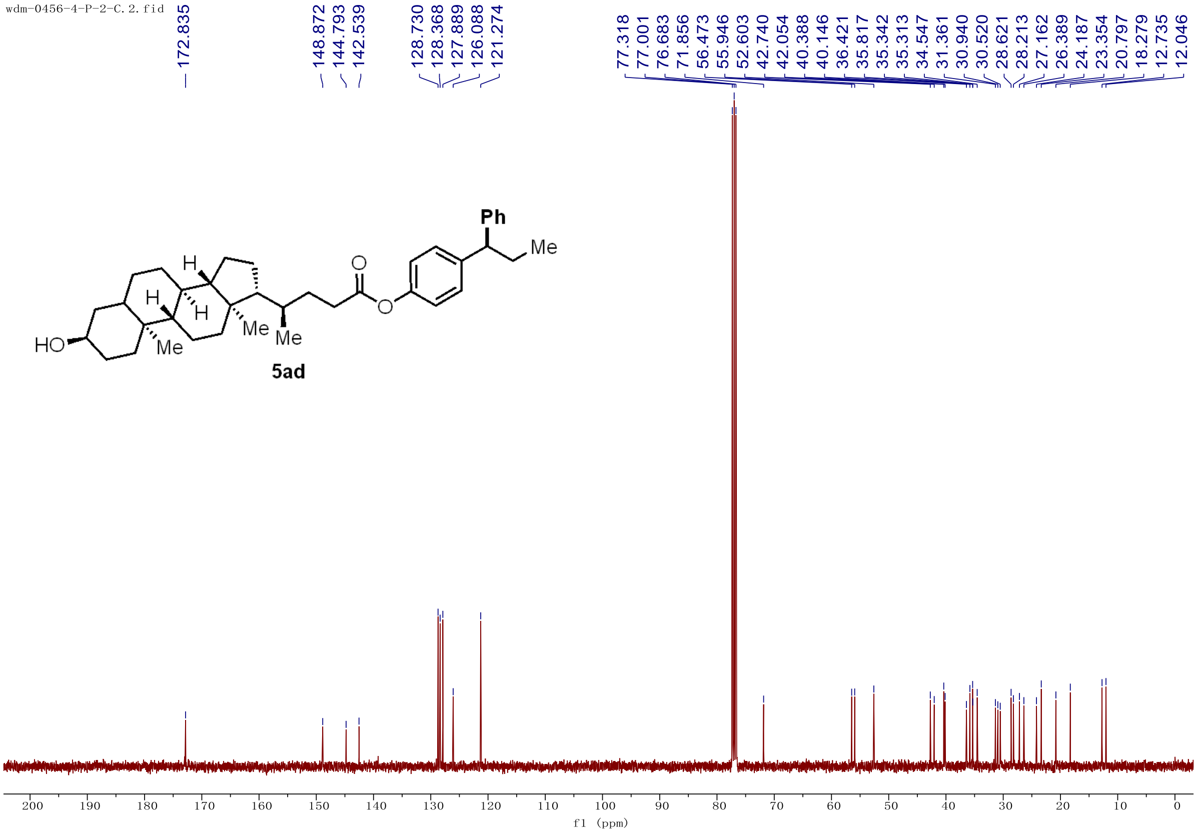

Supplementary Figure 170. ^{13}**C NMR (100 MHz, CDCl$_3$) spectrum of 5ad**

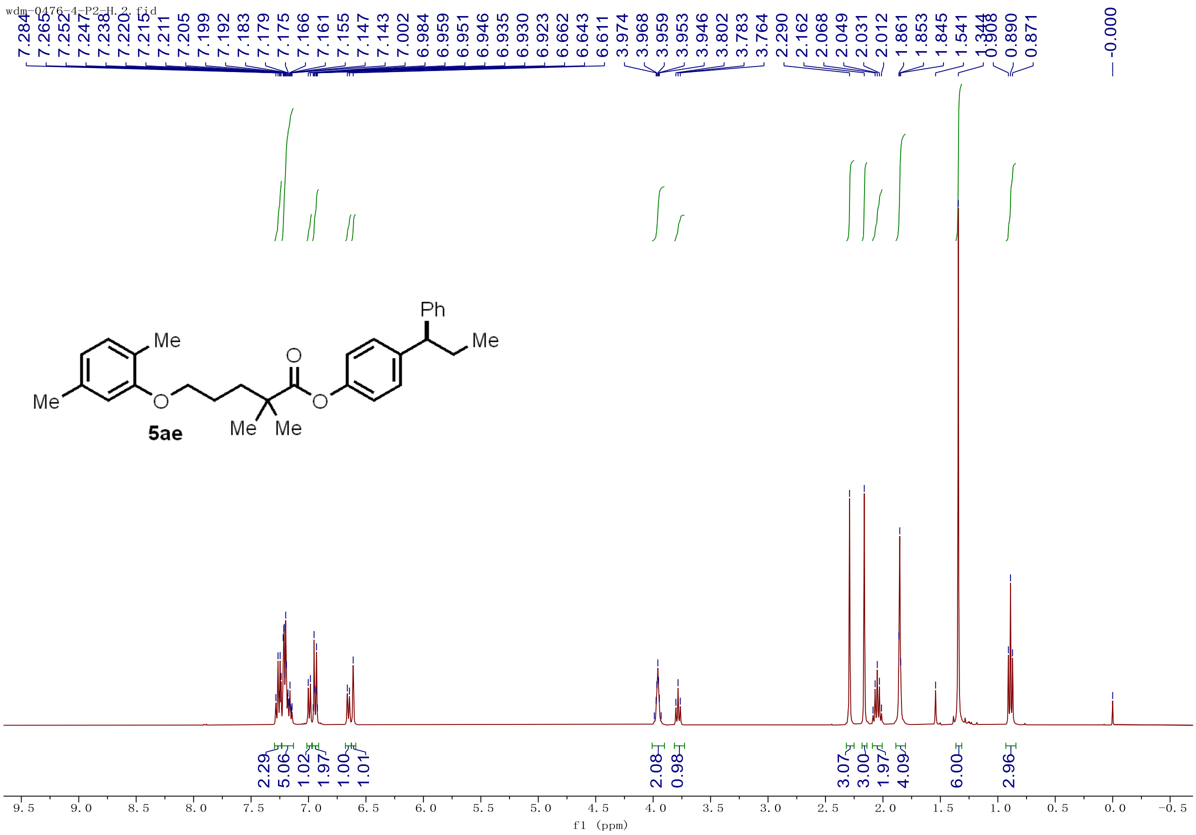

Supplementary Figure 171. ^{1}**H NMR (400 MHz,** $CDCl_3$**) spectrum of 5ae**

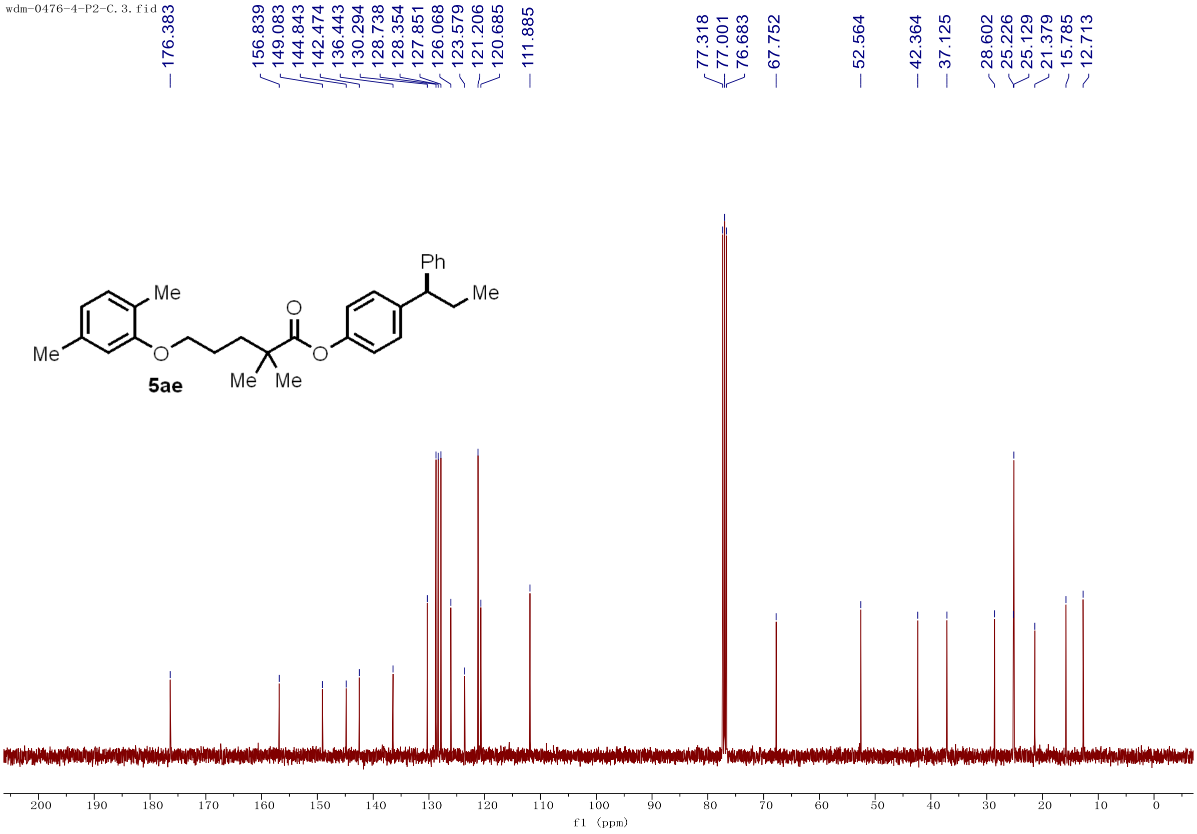

Supplementary Figure 172. ^{13}**C NMR (100 MHz,** $CDCl_3$**) spectrum of 5ae**

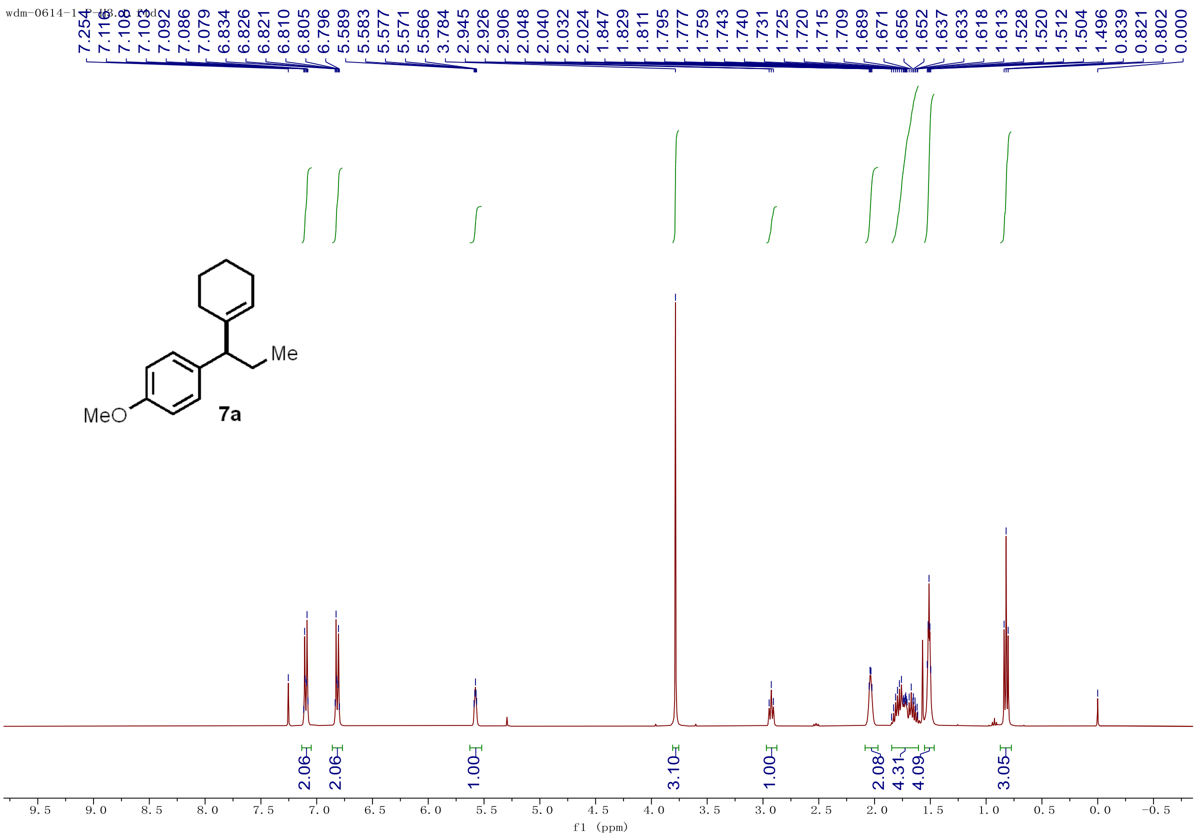

Supplementary Figure 173. ^{1}H NMR (400 MHz, $CDCl_3$) spectrum of 7a

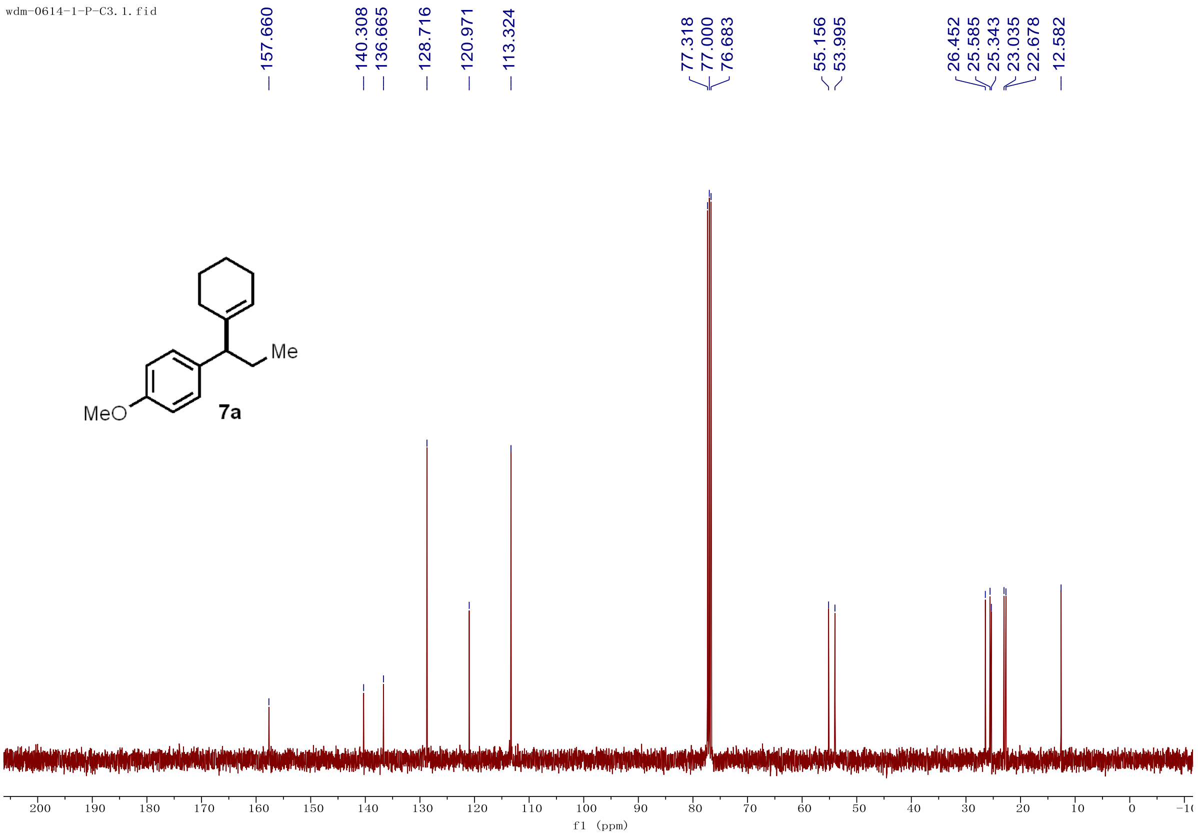

Supplementary Figure 174. ^{13}C NMR (100 MHz, $CDCl_3$) spectrum of 7a

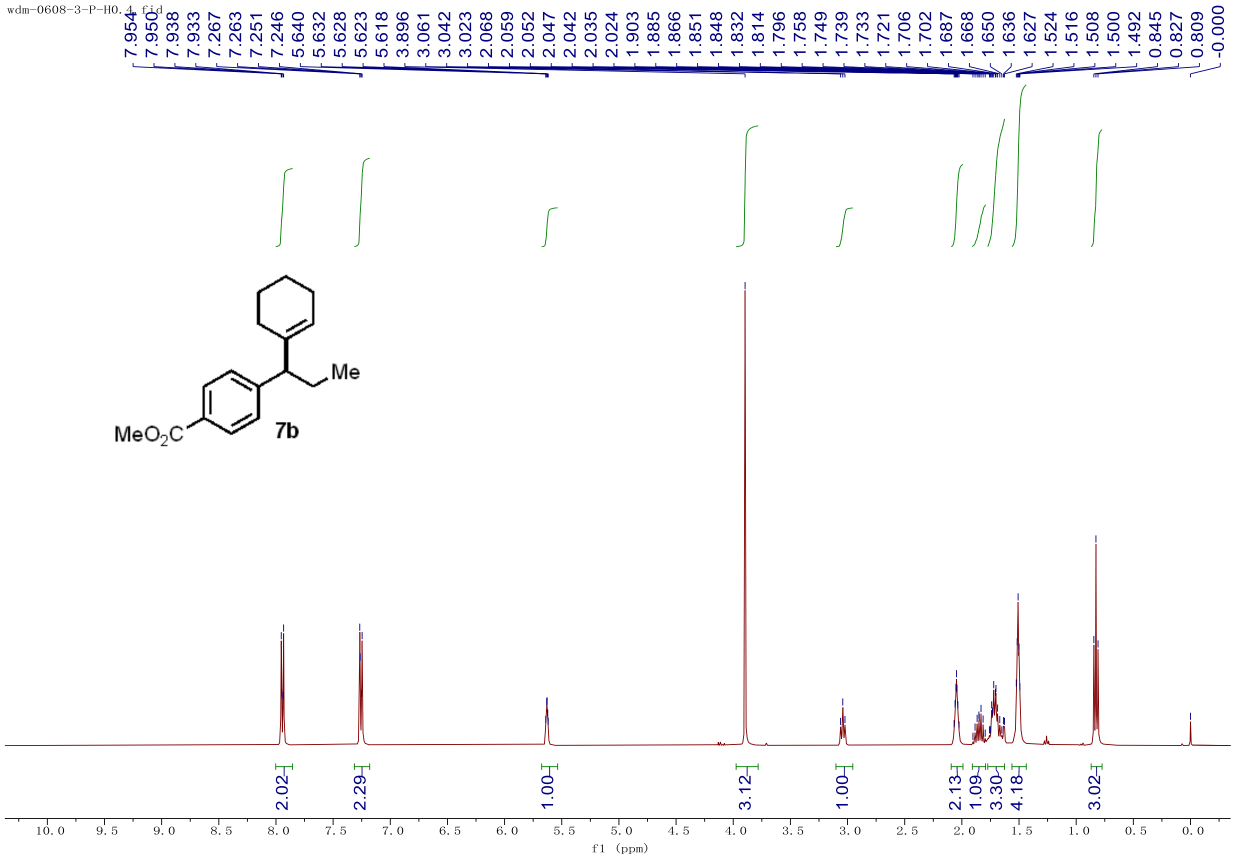

Supplementary Figure 175. ^{1}**H NMR (400 MHz, CDCl₃) spectrum of 7b**

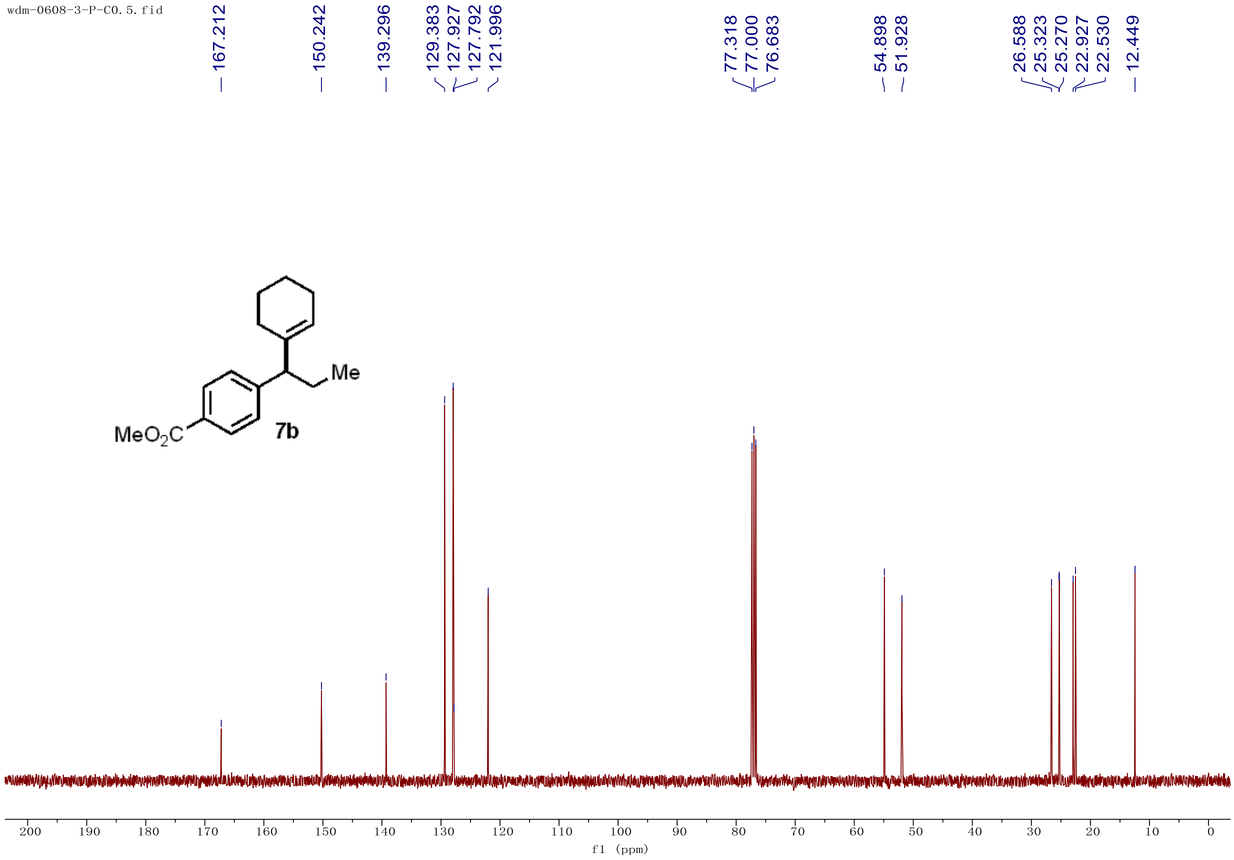

Supplementary Figure 176. ^{13}**C NMR (100 MHz, CDCl₃) spectrum of 7b**

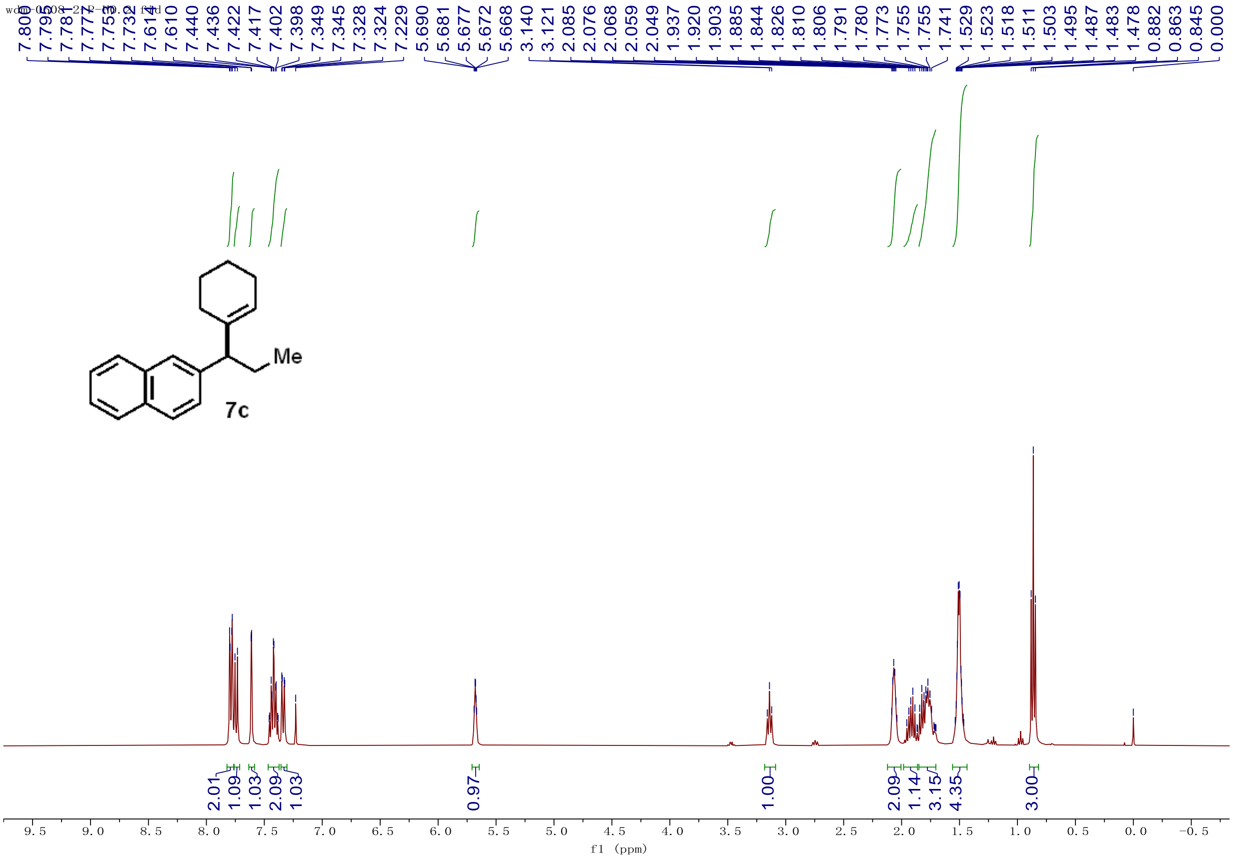

Supplementary Figure 177. ^{1}**H NMR (400 MHz, CDCl**$_3$**) spectrum of 7c**

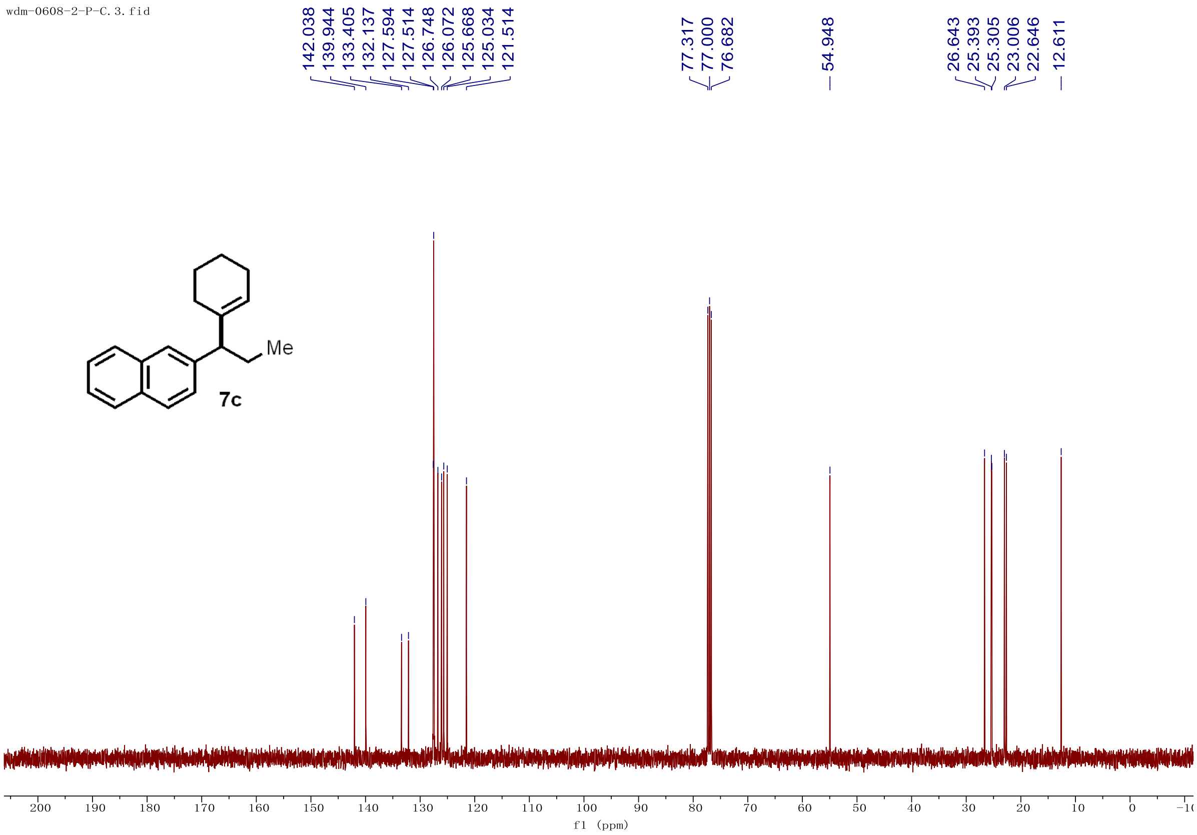

Supplementary Figure 178. ^{13}**C NMR (100 MHz, CDCl**$_3$**) spectrum of 7c**

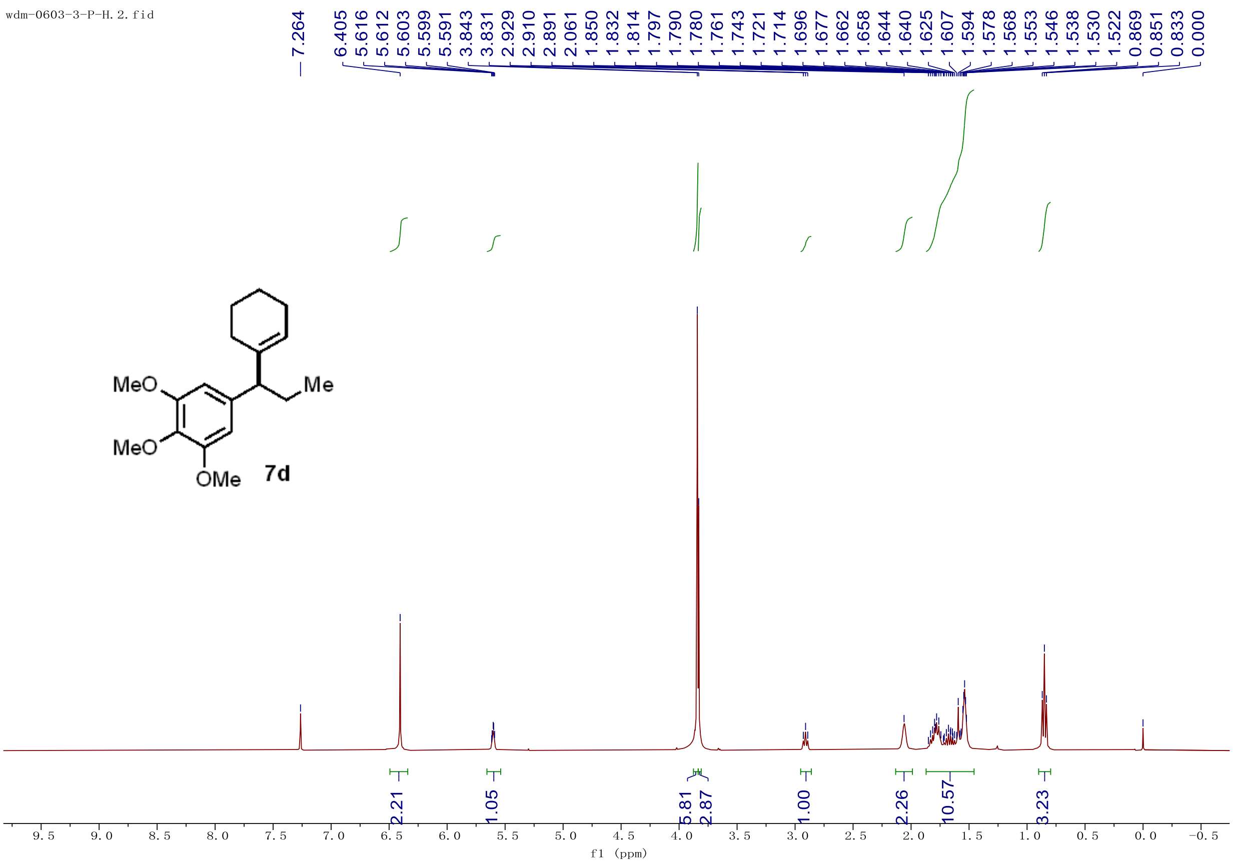

Supplementary Figure 179. ^{1}H NMR (400 MHz, $CDCl_3$) spectrum of 7d

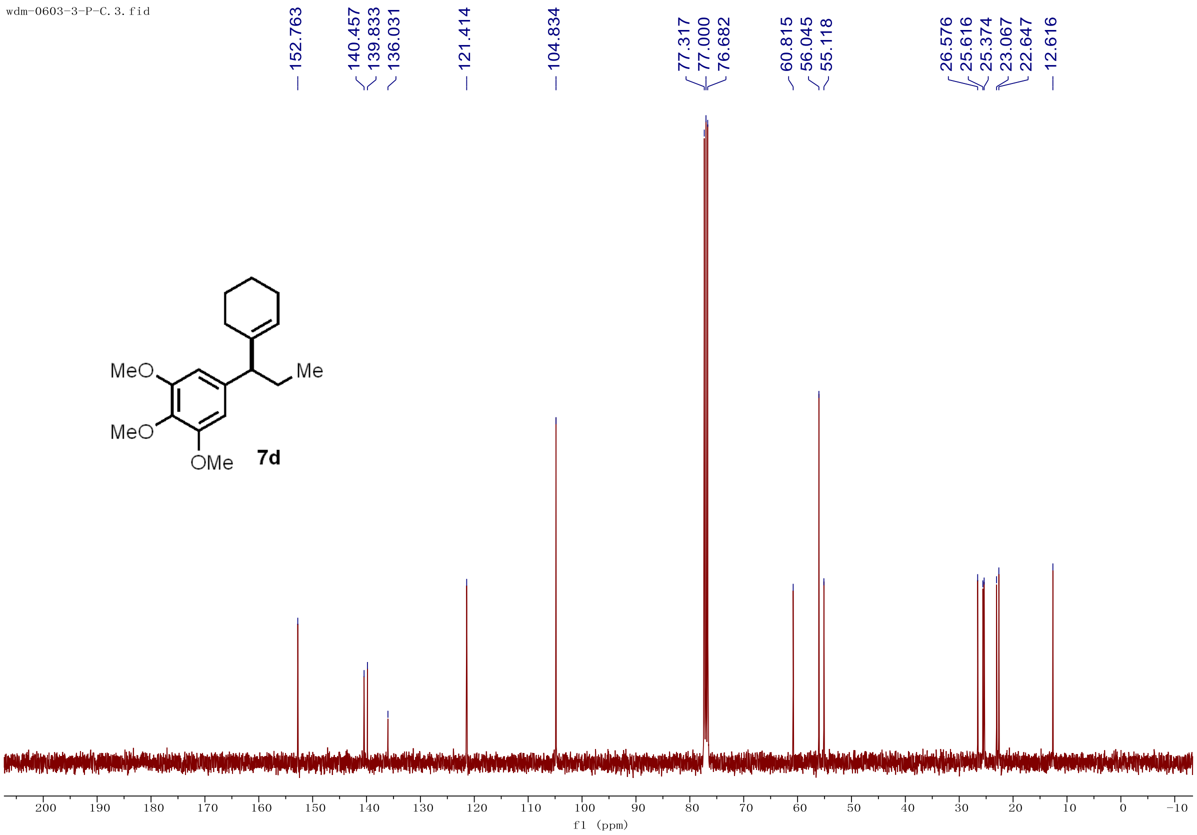

Supplementary Figure 180. ^{13}C NMR (100 MHz, $CDCl_3$) spectrum of 7d

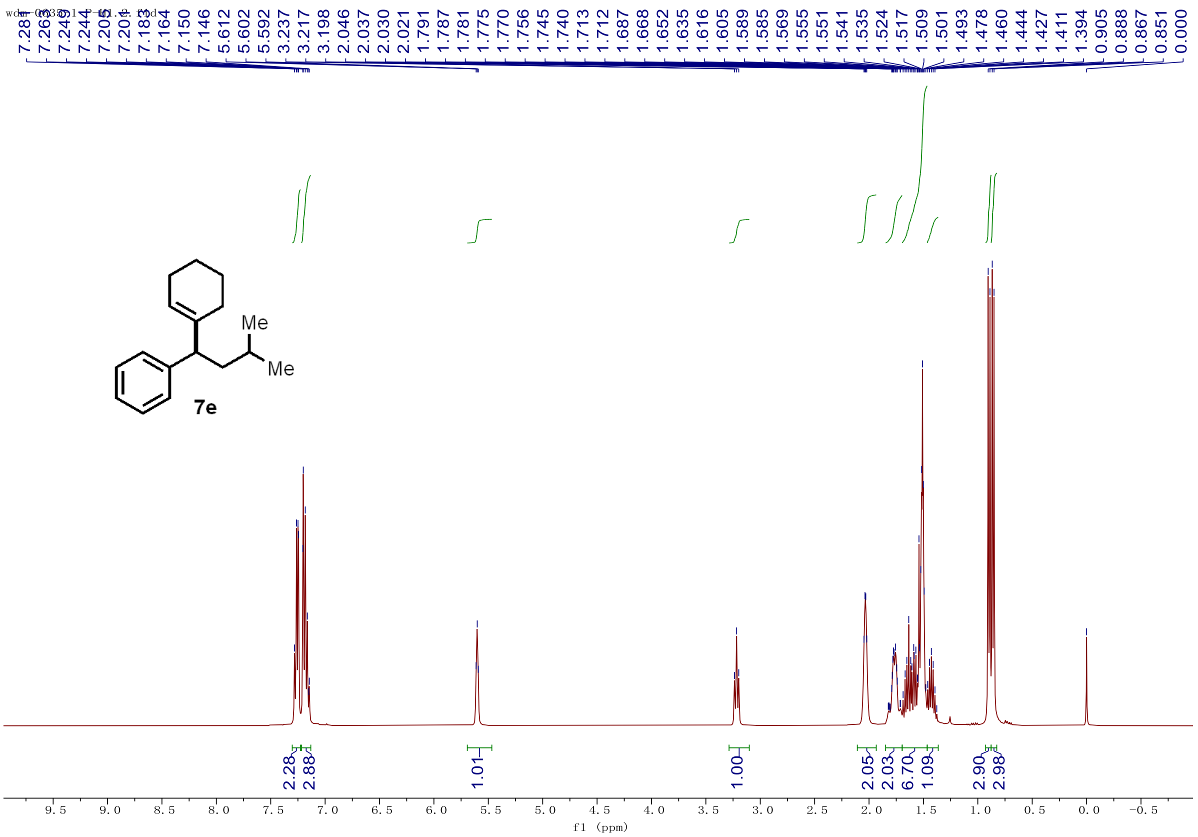

Supplementary Figure 181. ^{1}H NMR (400 MHz, $CDCl_3$) spectrum of 7e

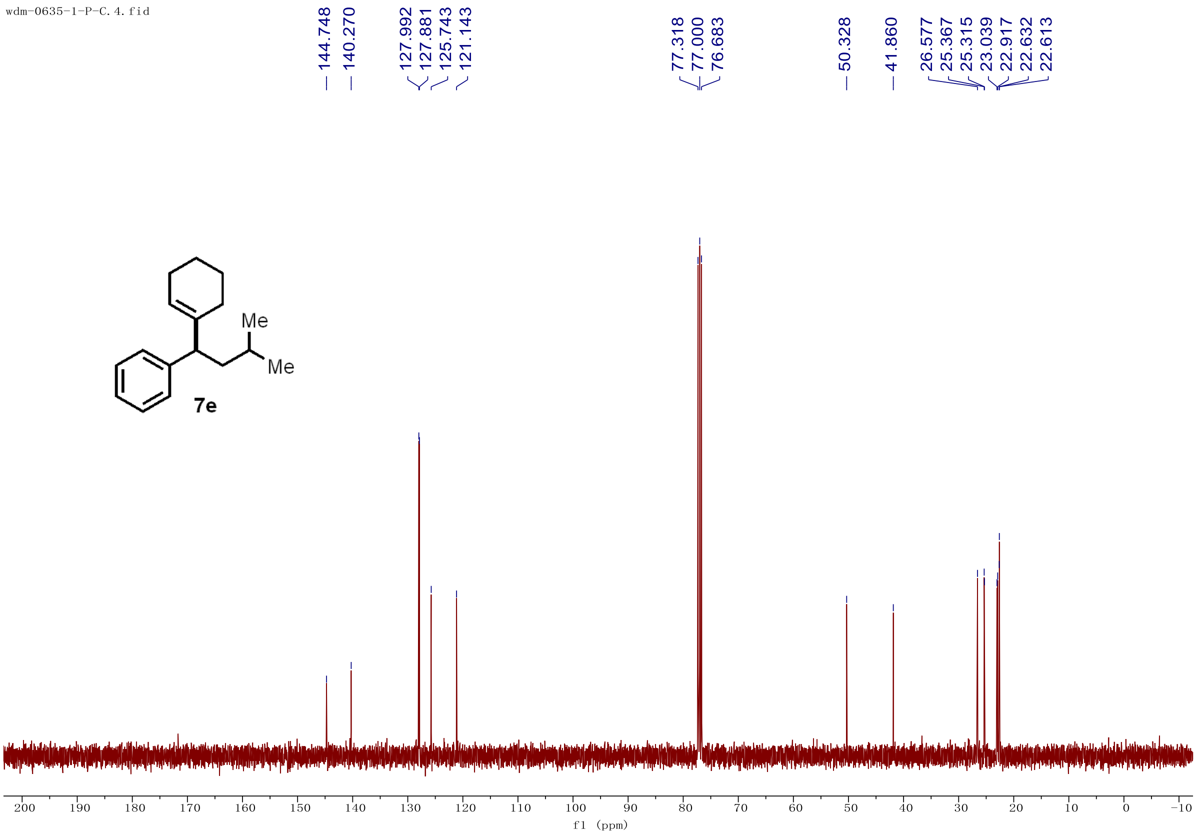

Supplementary Figure 182. ^{13}C NMR (100 MHz, $CDCl_3$) spectrum of 7e

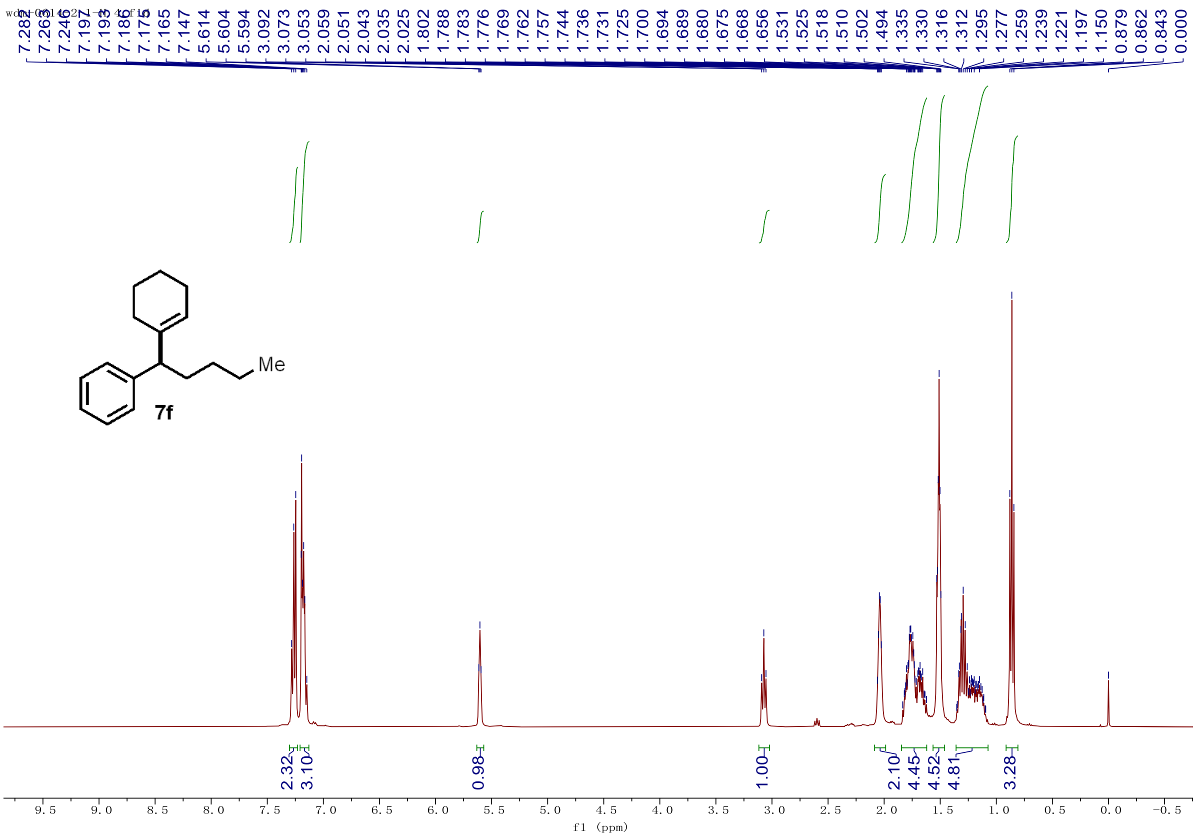

Supplementary Figure 183. ^{1}H NMR (400 MHz, $CDCl_3$) spectrum of 7f

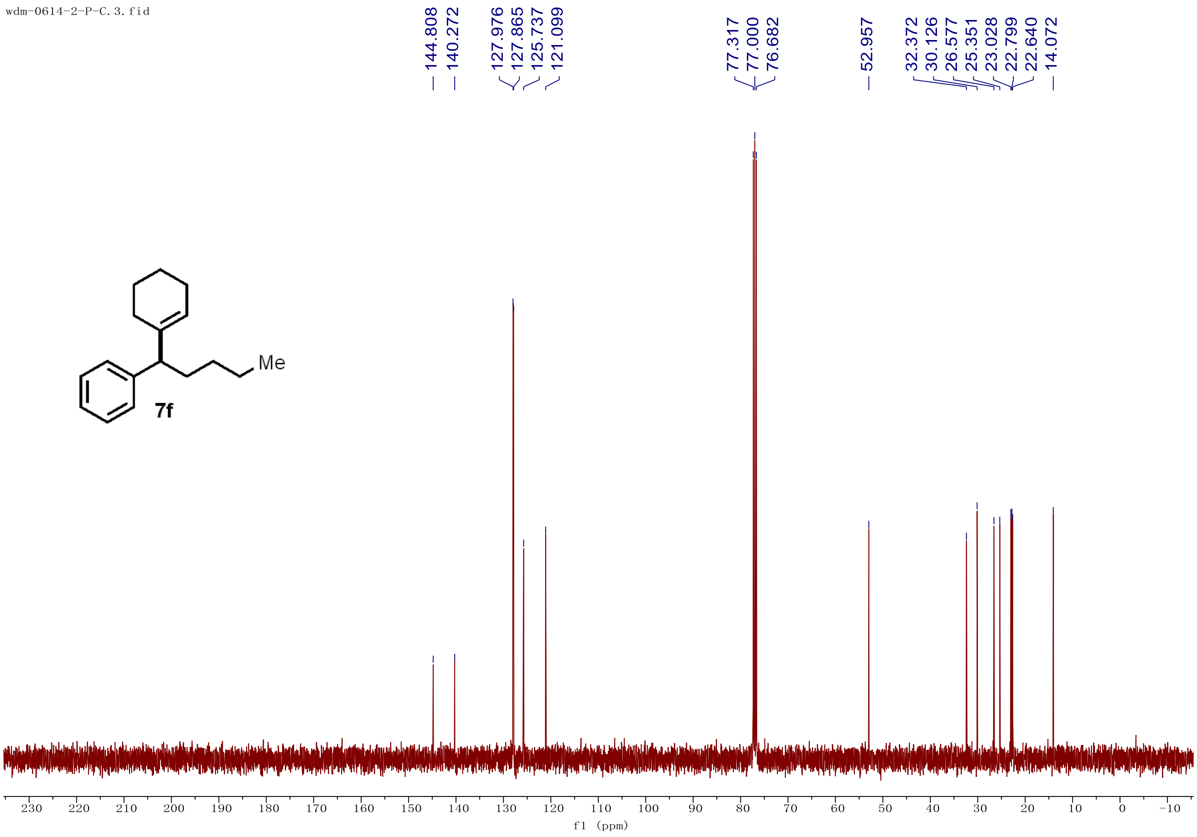

Supplementary Figure 184. ^{13}C NMR (100 MHz, $CDCl_3$) spectrum of 7f

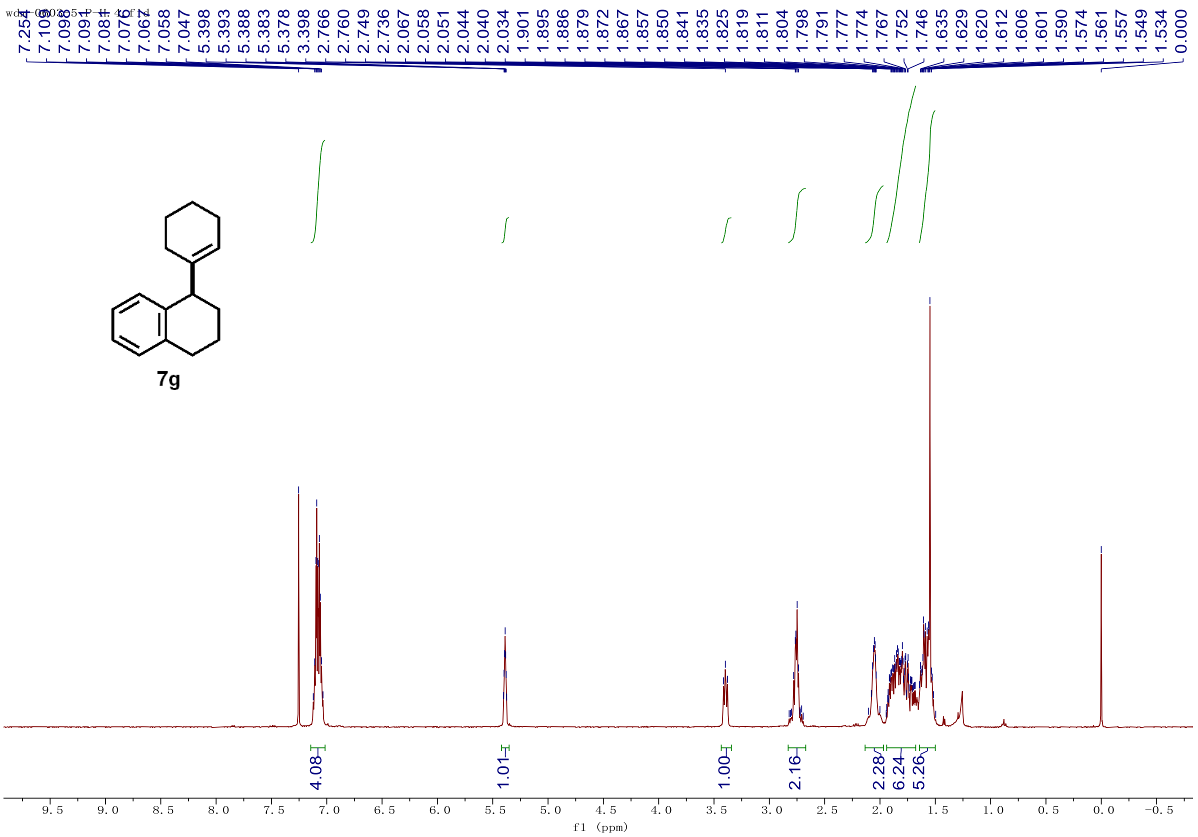

Supplementary Figure 185. ^{1}H NMR (400 MHz, $CDCl_3$) spectrum of 7g

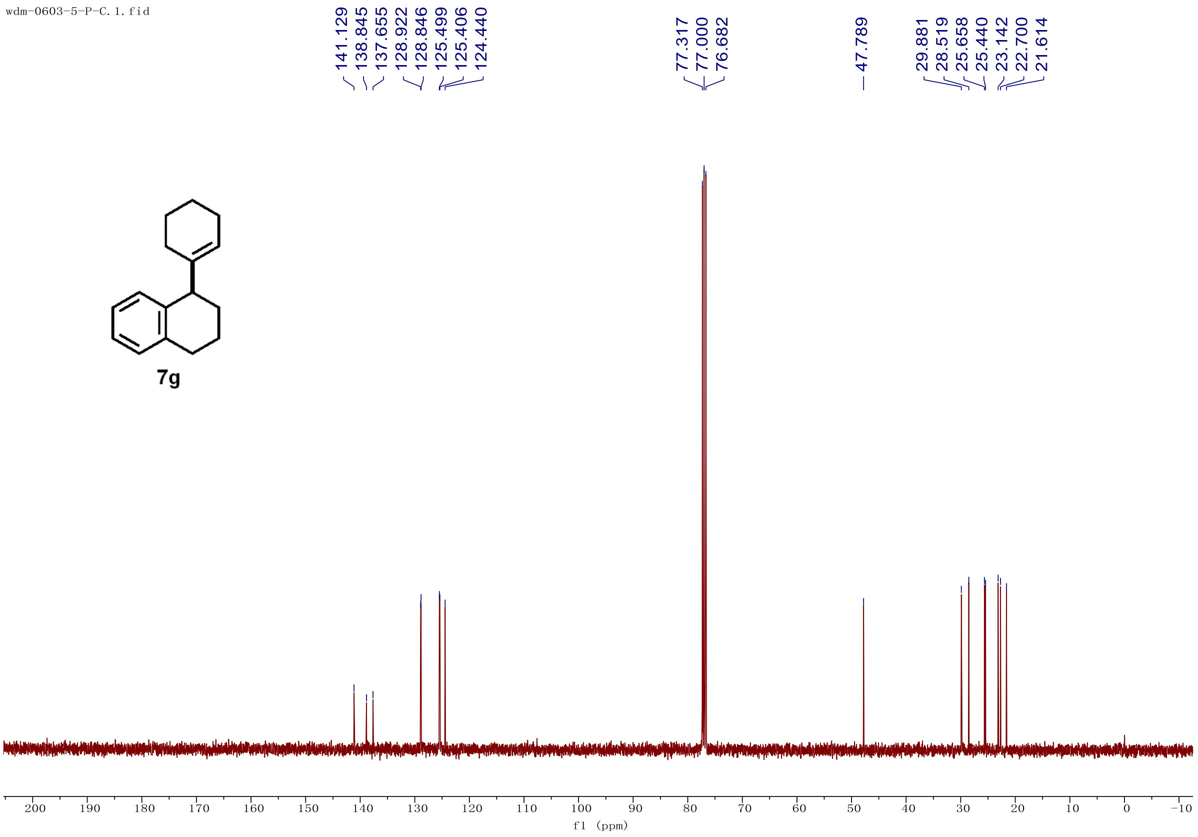

Supplementary Figure 186. ^{13}C NMR (100 MHz, $CDCl_3$) spectrum of 7g

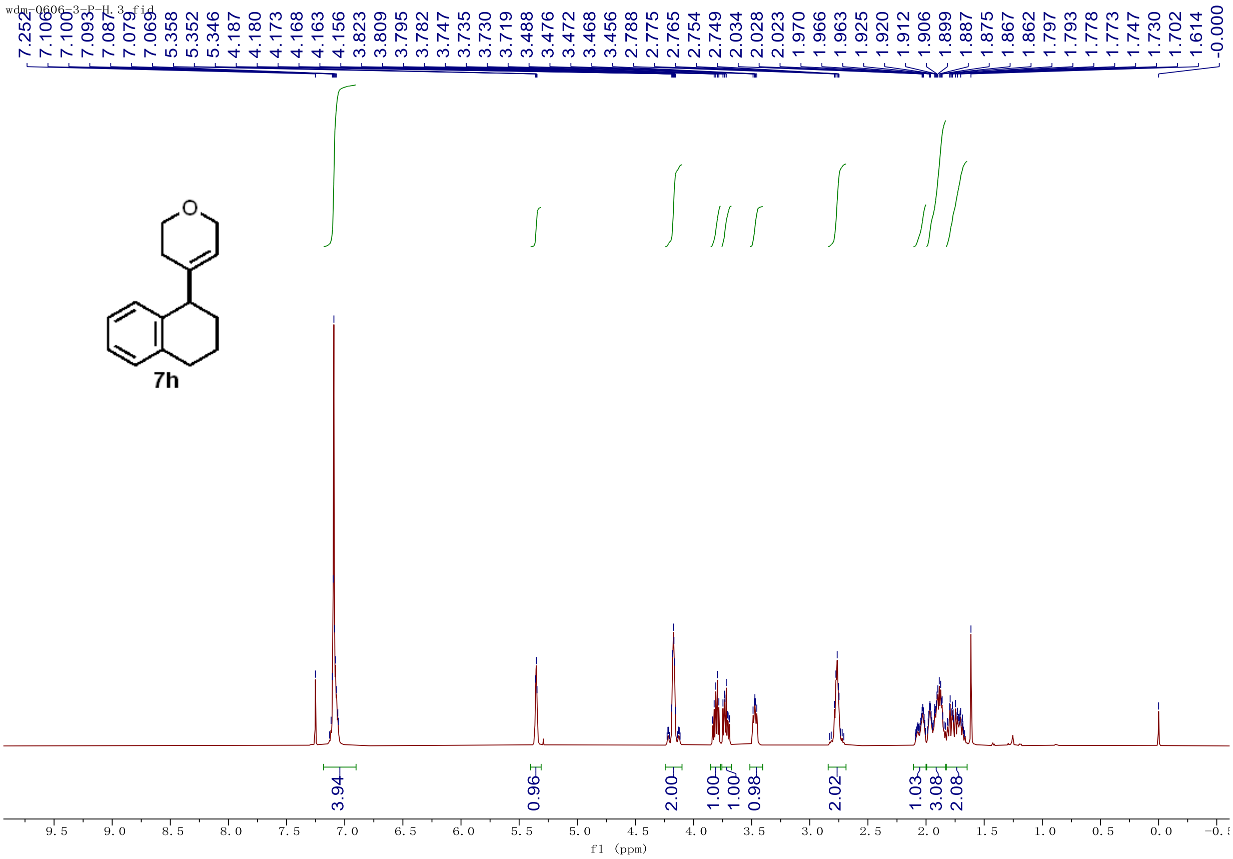

Supplementary Figure 187. ¹H NMR (400 MHz, CDCl₃) spectrum of 7h

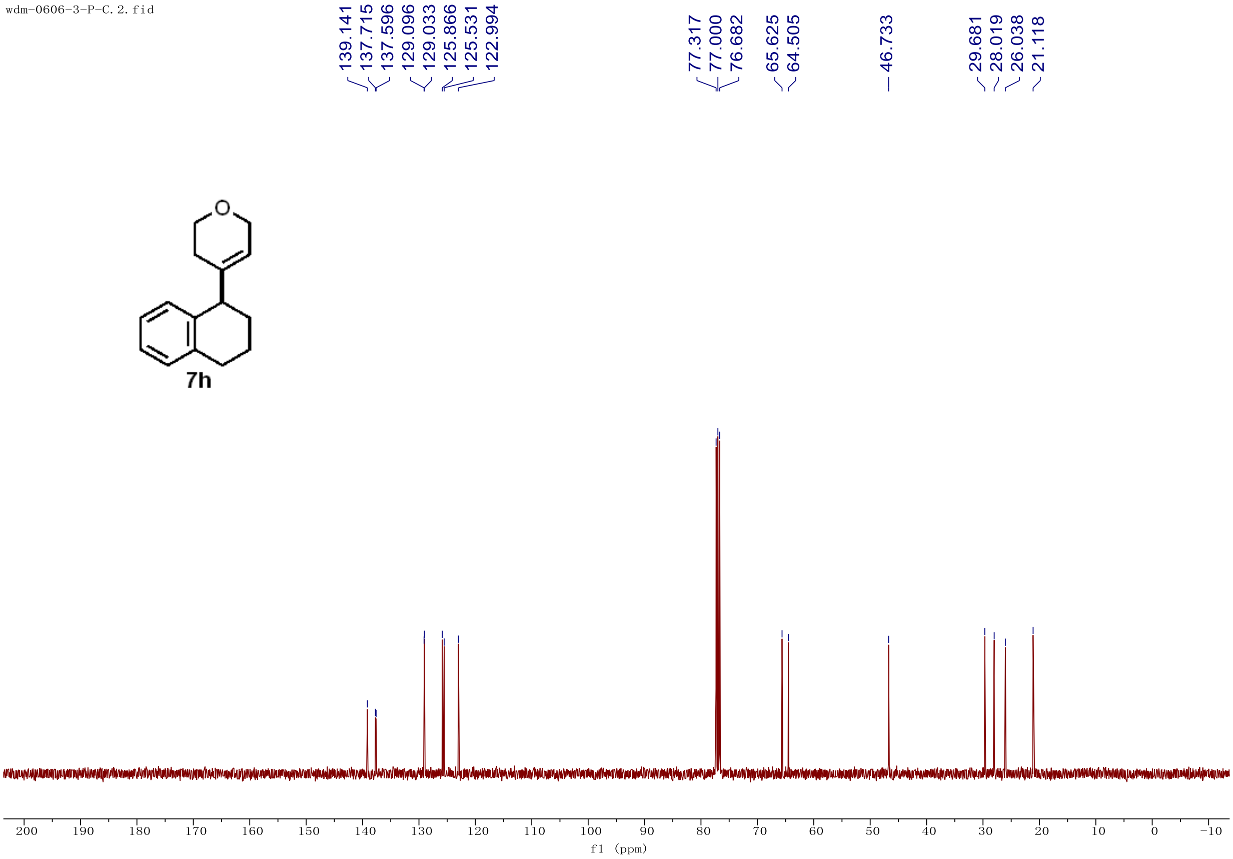

Supplementary Figure 188. ¹³C NMR (100 MHz, CDCl₃) spectrum of 7h

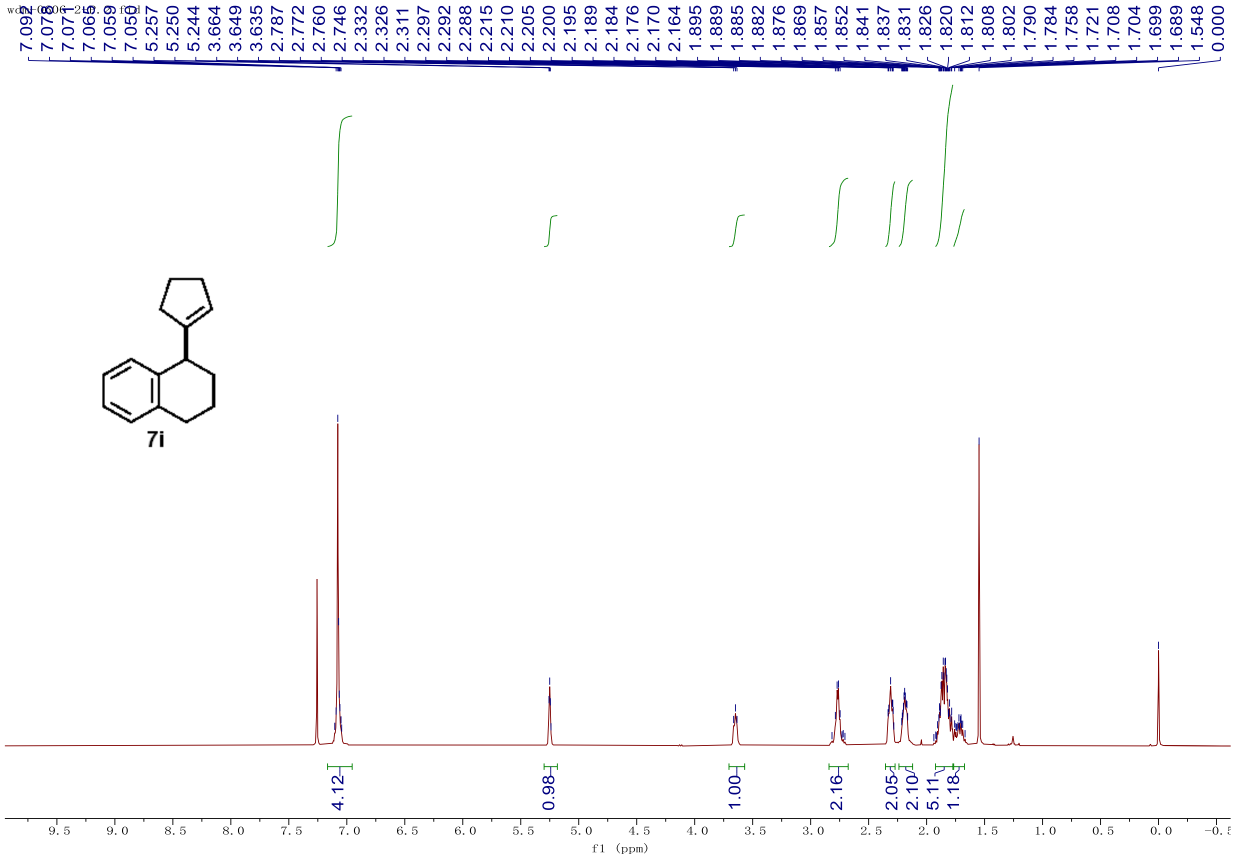

Supplementary Figure 189. ^{1}H **NMR (400 MHz, CDCl₃) spectrum of 7i**

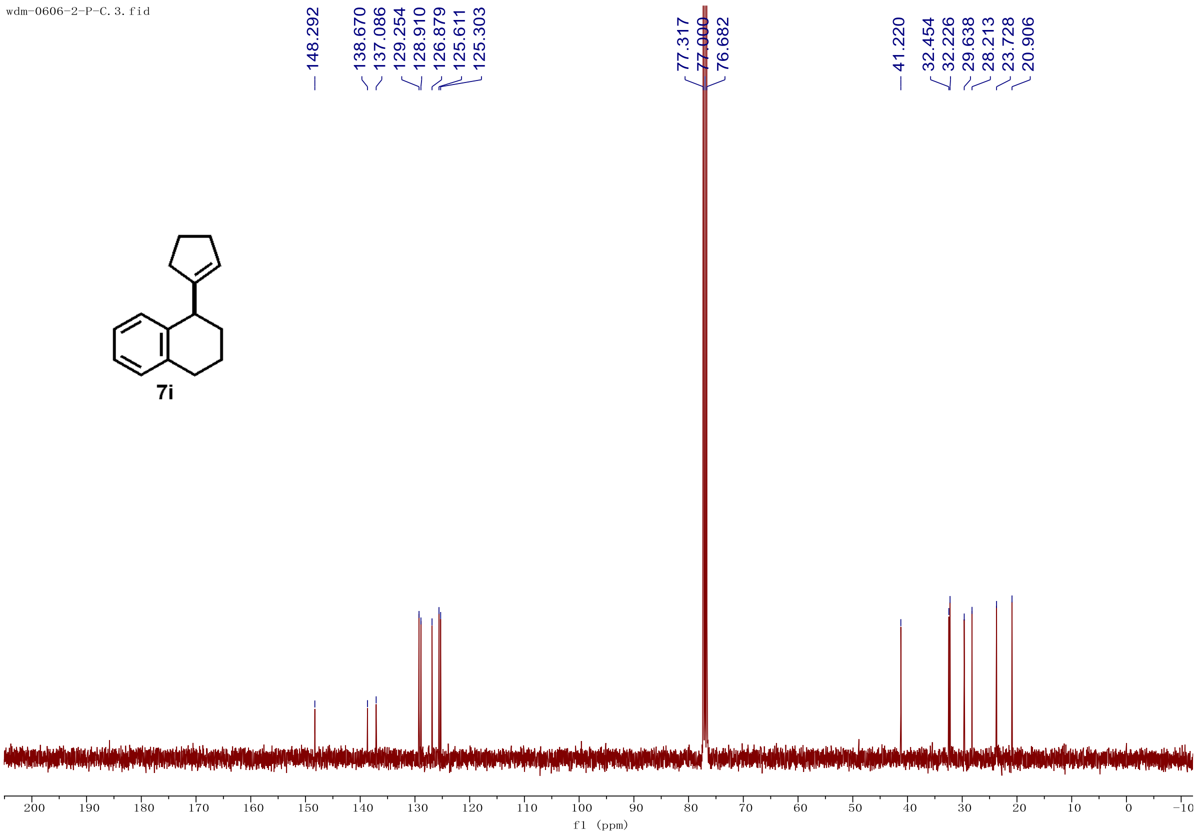

Supplementary Figure 190. ^{13}C **NMR (100 MHz, CDCl₃) spectrum of 7i**

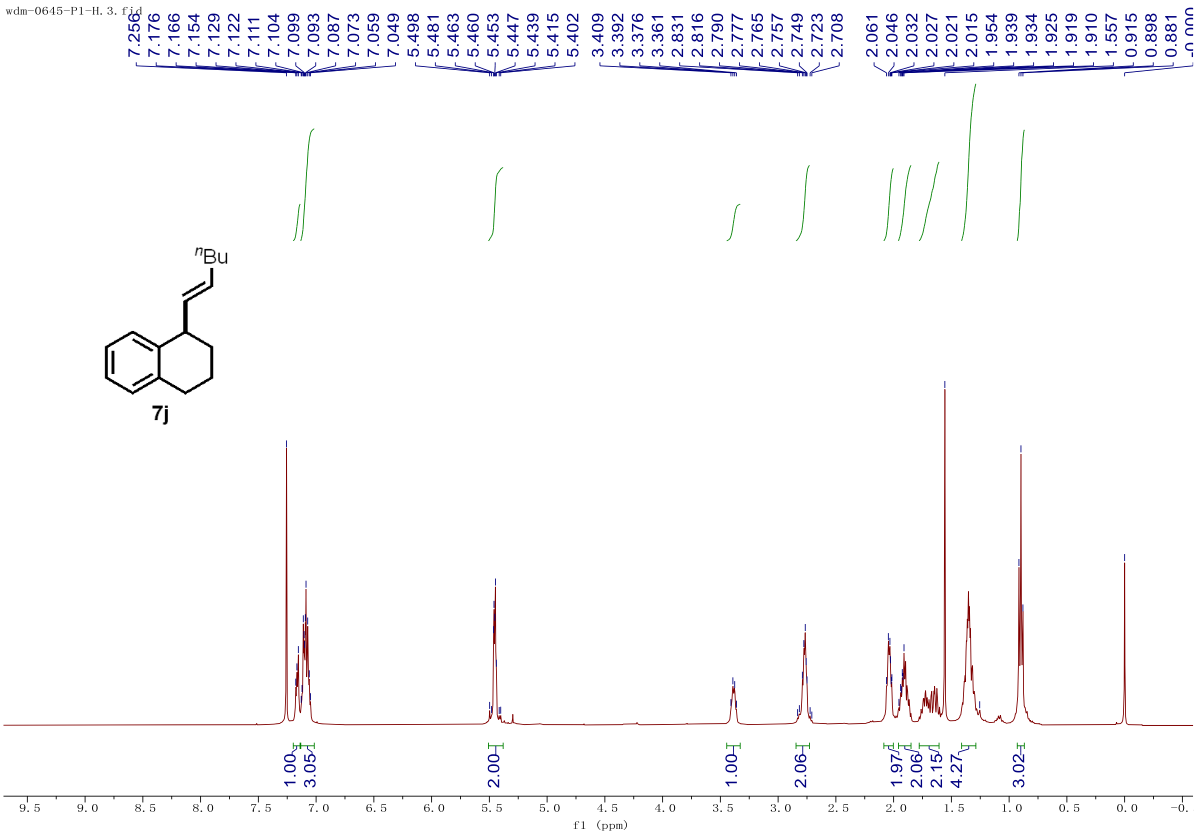

Supplementary Figure 191. ^{1}H NMR (400 MHz, $CDCl_3$) spectrum of **7j**

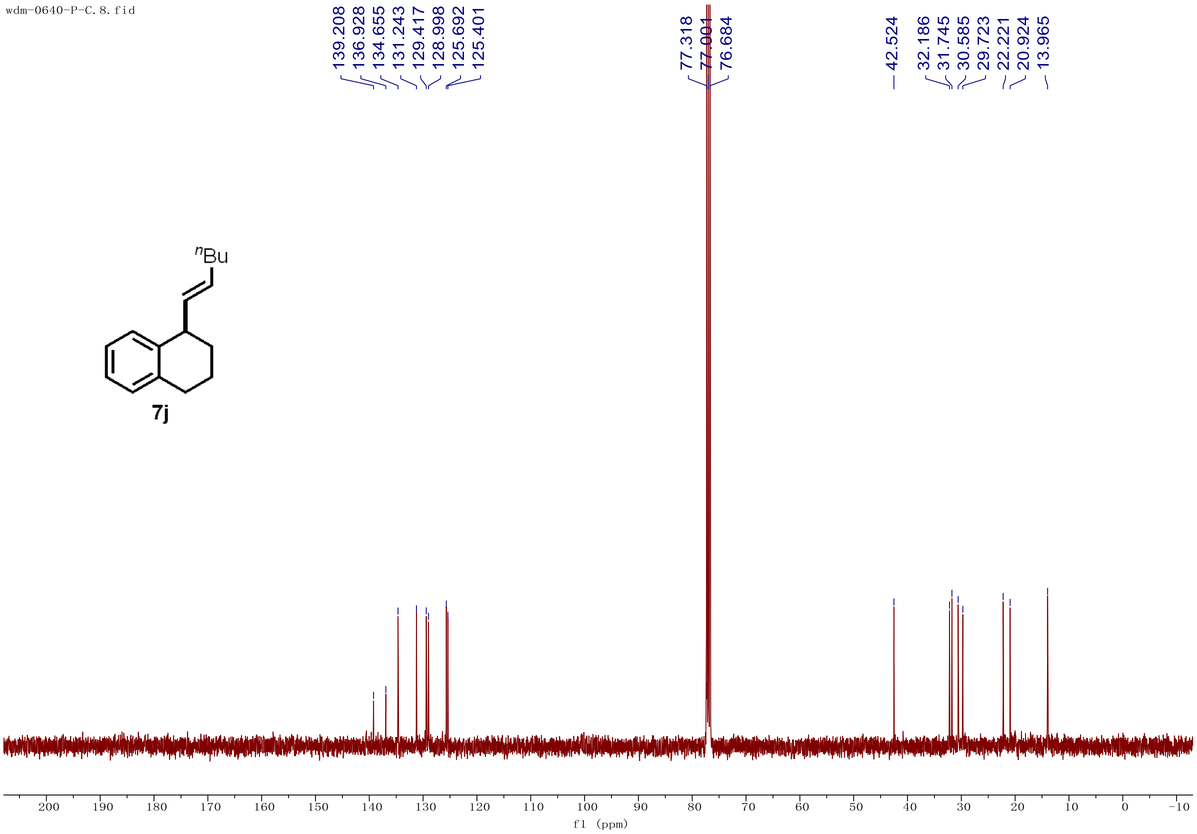

Supplementary Figure 192. ^{13}C NMR (100 MHz, $CDCl_3$) spectrum of **7j**

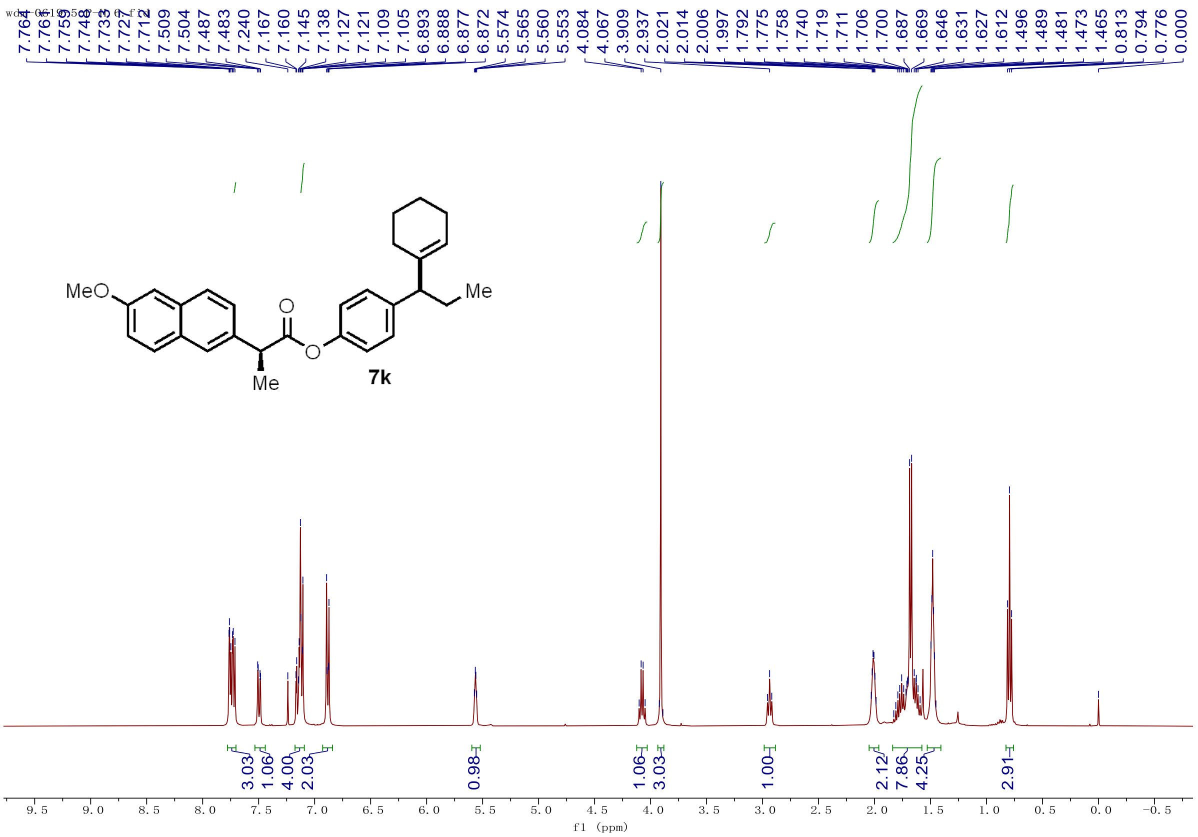

Supplementary Figure 193. ^{1}H NMR (400 MHz, CDCl$_3$) spectrum of 7k

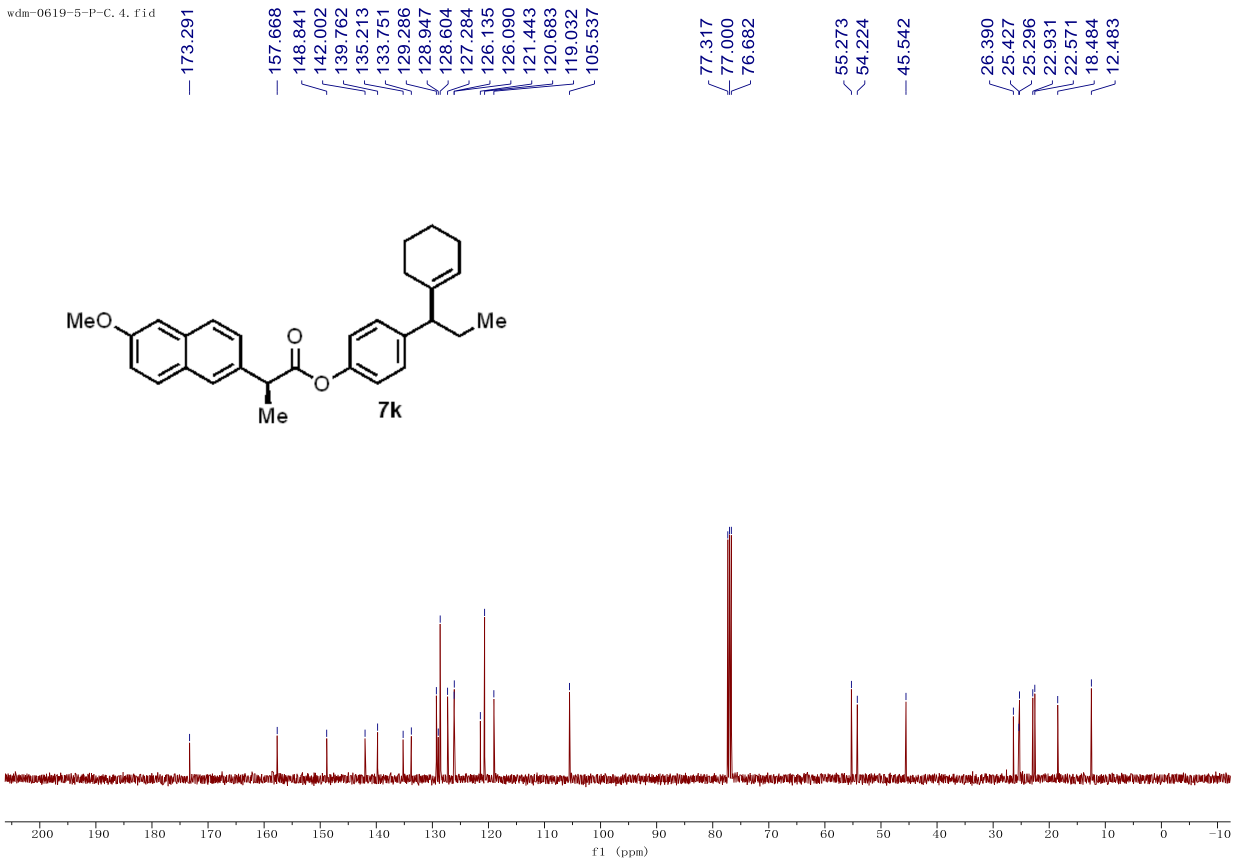

Supplementary Figure 194. ^{13}C NMR (100 MHz, CDCl$_3$) spectrum of 7k

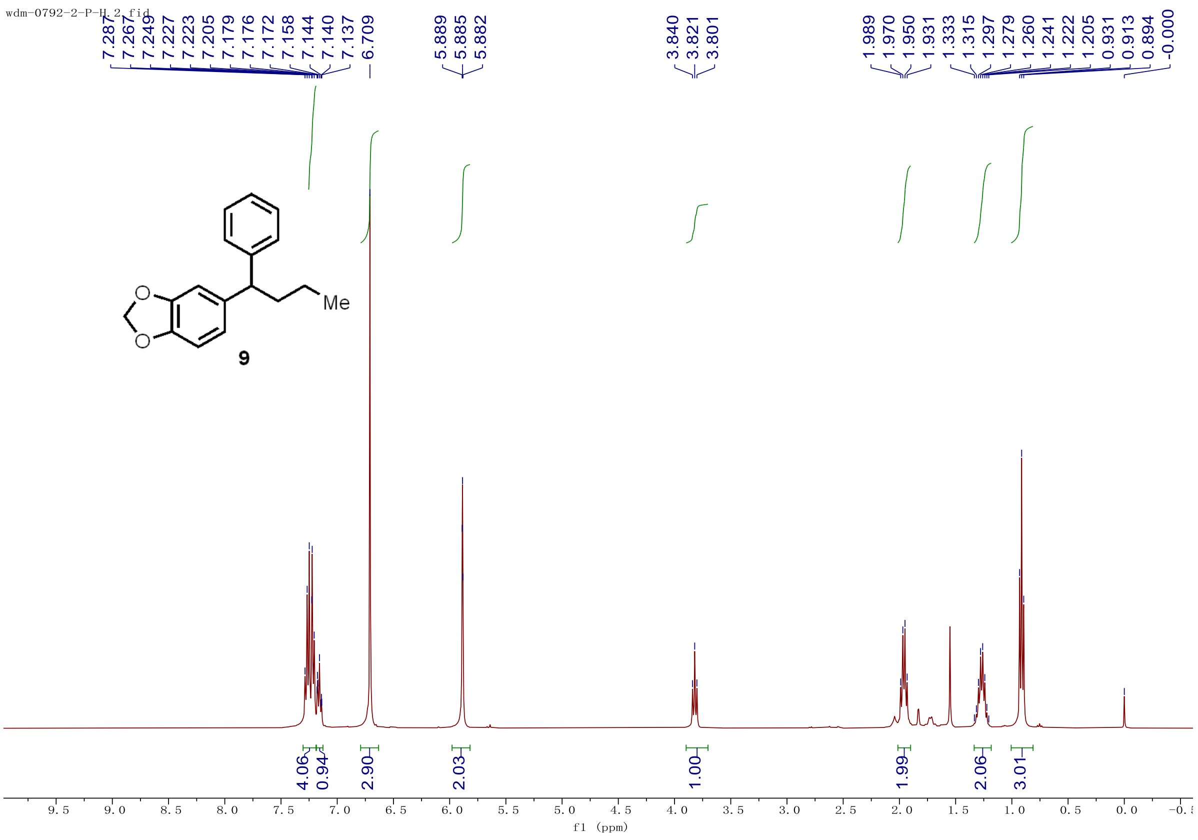

Supplementary Figure 195. ^{1}H NMR (400 MHz, $CDCl_3$) spectrum of 9

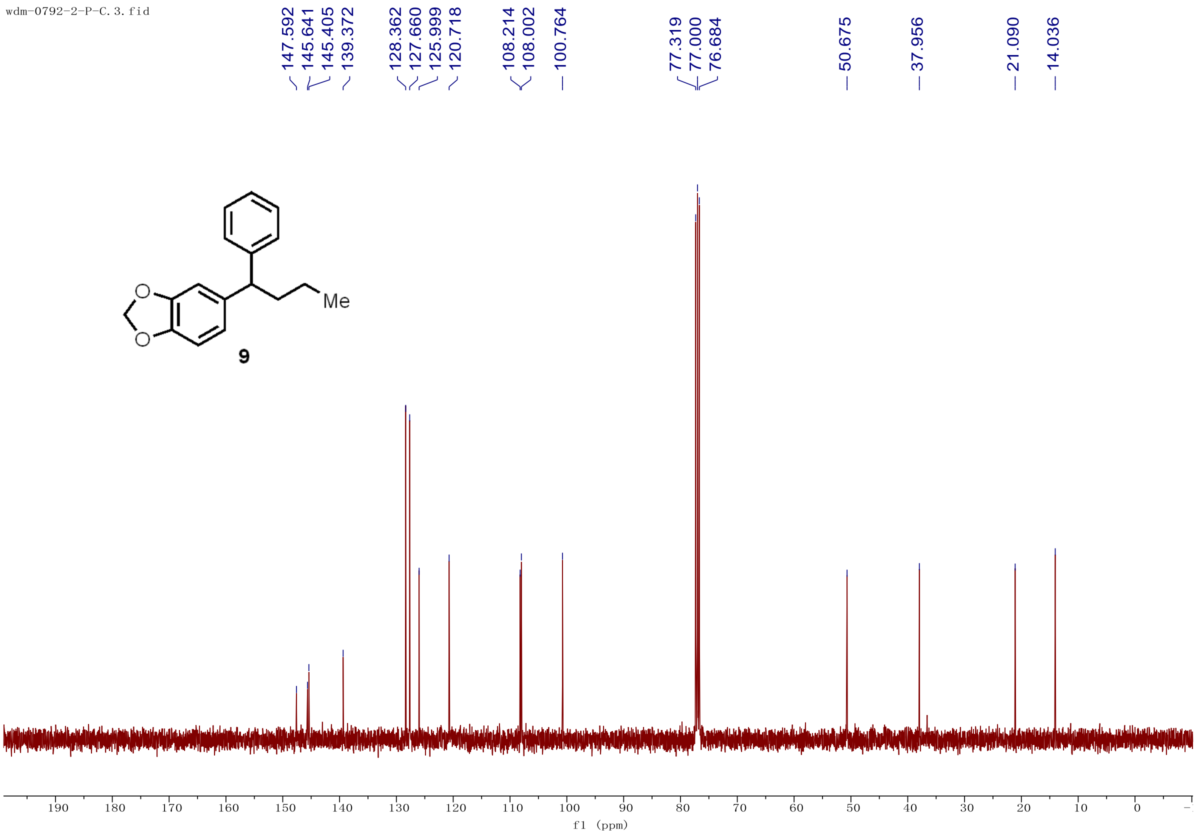

Supplementary Figure 196. ^{13}C NMR (100 MHz, $CDCl_3$) spectrum of 9
